# Development of a high-throughput strategy for discovery of potent analogues of antibiotic lysocin E

**DOI:** 10.1038/s41467-019-10754-4

**Published:** 2019-07-05

**Authors:** Hiroaki Itoh, Kotaro Tokumoto, Takuya Kaji, Atmika Paudel, Suresh Panthee, Hiroshi Hamamoto, Kazuhisa Sekimizu, Masayuki Inoue

**Affiliations:** 1 Graduate School of Pharmaceutical Sciences, The University of Tokyo, 7-3-1 Hongo, Bunkyo-ku, Tokyo, 113-0033 Japan; 2 Teikyo University Institute of Medical Mycology, 359 Otsuka, Hachioji, Tokyo, 192-0395 Japan

**Keywords:** Combinatorial libraries, Natural product synthesis, Solid-phase synthesis, Drug discovery and development

## Abstract

Lysocin E, a 37-membered natural depsipeptide, induces rapid bacteriolysis in methicillin-resistant *Staphylococcus aureus* via a unique menaquinone-dependent mechanism, presenting a promising therapeutic lead. Despite the great medical importance, exploring the potential utility of its derivatives as new platform structures for antibiotic development has remained a significant challenge. Here, we report a high-throughput strategy that enabled the preparation of thousands of analogues of lysocin E and large-scale structure-activity relationship analyses. We integrate 26-step total synthesis of 2401 cyclic peptides, tandem mass spectrometry-sequencing, and two microscale activity assays to identify 23 candidate compounds. Re-synthesis of these candidates shows that 11 of them (**A1**–**A11**) exhibit antimicrobial activity superior or comparable to that of lysocin E, and that lysocin E and **A1**–**A11** share l-Leu-6 and l-Ile-11. Therefore, the present strategy allows us to efficiently decipher biologically crucial residues and identify potentially useful agents for the treatment of infectious diseases.

## Abstract (web summary)

The depsipeptide Lysocin E has antibacterial activity against methicillin-resistant *Staphylococcus aureus*. Here, the authors developed a high-throughput one-bead-on-compound method for the synthesis and screening lysocin E derivatives, with several hits being more active than the parent compound.

## Introduction

As the emergence of multidrug-resistant bacteria poses a serious threat to public health around the world^1–3^, as in the case with hospital-acquired methicillin-resistant *Staphylococcus aureus* (MRSA), there is an urgent need for the discovery and development of effective antibiotics with new modes of action against the resistant strains^4,5^. Natural products have been essential sources of new drugs for infectious diseases^6–9^. While having evolutionarily selected structures, natural products themselves do not necessarily provide optimal properties as therapeutic agents. To identify desirable drug candidates with enhanced antibacterial activity derived from a natural product, structure-activity relationship (SAR) studies are typically required through syntheses of a wide variety of analogues based on its structural framework^10–13^. Total synthesis offers the most flexible and powerful approach to generating diverse structures and therefore addresses major challenges in the SAR analysis, where structurally complex natural products are often difficult to isolate in large quantities or to chemically modify^14^. However, one-by-one multi-step synthesis of many analogues is extremely time-consuming and impractical. Consequently, the potential of natural products has not been fully explored due to lack of efficient and robust synthetic methods for analogue generation. A highly efficient strategy for preparing and evaluating the natural product-based compounds for the development of pharmaceuticals is therefore in high demand.

**Fig. 1:**
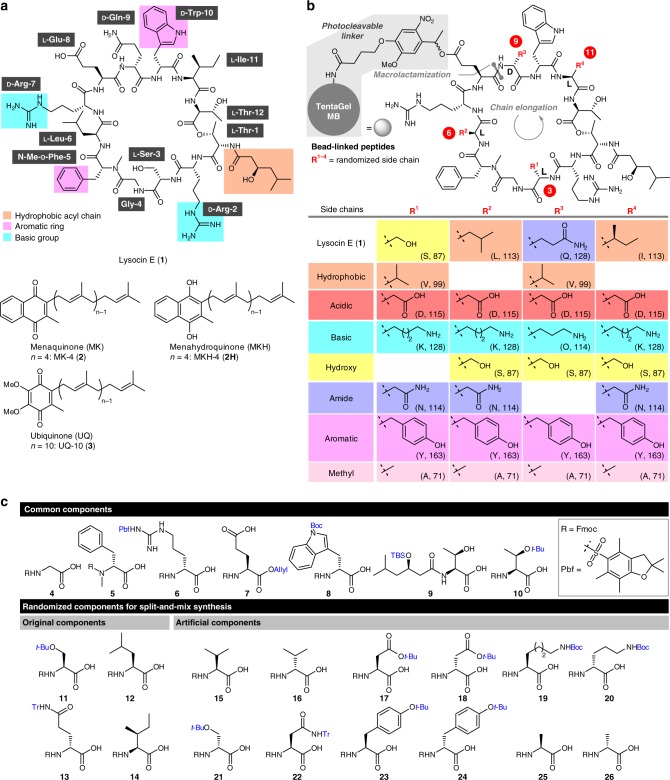
Chemical structures of compounds used in this study. **a** Structures of lysocin E (**1**), menaquinone-4 (MK-4, **2**), menahydroquinone-4 (MKH-4, **2 H**), and ubiquinone-10 (UQ-10, **3**). Side-chain structures of **1** important for the potent antimicrobial activity are highlighted in orange (hydrophobic acyl chain), magenta (aromatic ring), and cyan (basic group). **b** Structure of the **1**-based one-bead-one-compound (OBOC) library comprising the bead-linked peptides. R^1-4^ denote randomized side chains at residues-3, -6, -9, and -11 by split-and-mix synthesis. Table: Structures and properties of side chains at residues-3, -6, -9, and -11 of the bead-linked lysocin E analogues. One-letter codes of the amino acids and molecular weight of the residues are displayed in parentheses. The configurations of residues-3, -6, -9, and -11, sites for the bead-linking and macrolactamization, and direction of the chain elongation are also indicated. Hydrophobic, acidic, basic, hydroxy, primary amide, aromatic, and methyl groups are highlighted in orange, red, cyan, yellow, purple, magenta, and pink, respectively. TentaGel MB TentaGel Macrobeads. **c** Component amino acids for construction of the **1**-based OBOC library. Protective groups to be removed on a solid support are indicated in blue. Boc *t*-butoxycarbonyl; Fmoc 9-fluorenymethoxycarbonyl; Pbf 2,2,4,6,7-pentamethyldihydrobenzofuran-5-sulfonyl; TBS *tert*-butyldimethylsilyl; *t*-Bu *tert*-butyl; Tr triphenylmethyl

Lysocin E (**1**, Fig. 1a) was isolated from *Lysobacter* sp. as a new antibiotic and structurally determined to be a 37-membered depsipeptide (molecular weight = 1618 Da) with an N-methylated amide and ester linkages^15^. The macrocyclic core of 12 residues contains four different non-proteinogenic d-amino acids (d-Arg-2/7, N-Me-d-Phe-5, d-Gln-9, d-Trp-10) and is appended with (*R*)-3-hydroxy-5-methylhexanamide. It displays potent activity against MRSA through rapid bacteriolysis with a minimum inhibitory concentration (MIC) of 4 μg/mL and a superior therapeutic effect in *S. aureus*-infected mice (ED_50_ = 0.5 mg/kg) to that of vancomycin (ED_50_ = 5.8 mg/kg)^15^. Our previous studies revealed that **1** exerts its activity by a mechanism distinct from any other antibiotics. It disrupts bacterial membrane through selective interaction with menaquinone (MK), which is essential for the respiration process driven by conversion between MK and its reduced form, menahydroquinone (MKH), in the cell membrane^16,17^. In vitro, **1** forms a 1:1 complex (K_D_ = 4.5 μM) with menaquinone-4 (MK-4, **2**), but has no affinity toward ubiquinone-10 (UQ-10, **3**). Since ubiquinone is a coenzyme in the mammalian respiratory chain in mitochondria, the complexation selectivity of **1** is likely responsible for its selective toxicity against the bacteria. Recently, Walker et al. reported that **1** also interacts with lipid II^18^, a precursor for bacterial cell wall synthesis. The potent bactericidal activity and the novel mode of action make naturally occurring **1** a promising lead compound for the development of new antibiotics against multidrug-resistant bacteria.

The unique biological property of **1** prompted us to launch chemical and biological studies of **1**. In 2015, we achieved a full solid-phase total synthesis of **1**^19^. By taking advantage of the established synthetic strategy, we subsequently prepared 15 analogues with different side-chain structures^20^. This SAR study led us to dissect the key structural features (highlighted in color in Fig. 1a) of **1**: (1) the cationic functionalities of d-Arg-2 and -7 for binding to the anionic polar heads of the membrane, (2) the hydrophobic acyl group at l-Thr-1 for partition into the membrane, and (3) the indole ring of d-Trp-10 and the phenyl ring of N-Me-d-Phe-5 for aromatic-aromatic interaction with MK^21^. However, none of the 15 analogues was more potent than the parent natural product **1**, exemplifying the formidable challenge in searching for more active analogues through one-by-one total synthesis.

Here we report a high throughput platform for constructing and screening 2401 lysocin E analogues that had not been readily available en masse through total synthesis of individual compounds. It permits us to discover 18 hit compounds (**A1**–**A18**) with a hit rate of 0.7%, of which 11 compounds (**A1**–**A11**) are revealed to have antimicrobial activity more potent than or comparable to that of the parent natural product **1**, as well as high MK-dependent membrane lytic activity.

## Results

### Plan for construction of lysocin E-based OBOC library

To find molecules with more potent antimicrobial activity, we envisioned to synthesize a much larger number of natural product analogues (>1000 molecules) than that used in conventional SAR studies of natural products^22–24^. To achieve high-throughput synthesis and evaluation of thousands of cyclic peptides, we adopted the one-bead-one-compound (OBOC) strategy (Fig. 1b)^25,26^. Important features of this strategy are (i) the split-and-mix approach can readily diversify the structures, (ii) each bead displays a unique chemical entity, (iii) the chemical structure on a bead can be determined by tandem mass spectrometry (MS/MS)^27^, and (iv) both on-bead and in-solution assays can be used. Whereas the OBOC approach has provided a powerful tool for constructing a library of small peptides, it has not been applied to complex natural products except for in a few reports^28,29^. Nonetheless, we assumed that it would be possible to construct a **1**-based OBOC library because of the high efficiency of our synthetic route to **1** (Fig. 1c)^19,20^. The full solid-phase synthesis of **1** was achieved in 8.0% total yield via stepwise elongation from the Wang resin-linked **7** using **4**–**6** and **8**–**14**, subsequent macrolactamization, and simultaneous acid-promoted removal of the protective groups (*t*-Bu, Tr, Boc, TBS, Pbf^30^) and the Wang resin.

In designing the OBOC library of **1** with high hit rate, the sites of the structural alterations were selected so that the perturbation in the biologically important substructures and the overall synthetic efficiency would be minimized (Fig. 1a). First, the C_α_-stereogenic centers of the 11 residues and the C_α_-methylene of Gly-4 were retained due to their potential influence on the bioactive three-dimensional conformation. Second, (*R*)-3-hydroxy-5-methylhexanamide, d-Arg-2/7, N-Me-d-Phe-5, and d-Trp-10 were maintained, because their substitutions decreased the activities^20^. Third, l-Glu-8, and l-Thr-1/12 were not substituted. While l-Glu-8 is essential as an anchor to a solid support, a change in l-Thr-1/12 was presumed to affect the yield of the solid-phase esterification, which is generally more sensitive to the surrounding structures than an amidation. These considerations led us to determine l-Ser-3, l-Leu-6, d-Gln-9, and l-Ile-11 as the substitution sites for the library construction.

As there was no prior knowledge on the structural requirements for residues-3, -6, -9, and -11 for improving activity, the side chains of substituting residues were diversified in terms of their physicochemical properties (Fig. 1b). Hydrophobic (orange), acidic (red), basic (cyan), hydroxy (yellow), primary amide (purple), and aromatic (magenta) groups were selected for this purpose. As a chemical version of alanine-scanning mutagenesis^31^, substitution with the methyl group (pink) was also included. To achieve unambiguous structure determination of the compound on each bead by MS/MS analysis, each residue-n (*n* = 3, 6, 9, 11) of the sequence must be identified with a unique molecular weight. As shown in Fig. 1b, the mass units of the side chains at residue-n (R^1-4^) thus all differed. It should be noted that d-lysine was to be used as a basic substitution except for d-ornithine at residue-9, because d-glutamine (Mw = 128) as residue-9 of **1**, has the same mass number as d-lysine (Mw = 128). Thus, 12 amino acid components **15**–**26** were necessary for the library in addition to the 11 original components **4**–**14** contained in lysocin E (Fig. 1c).

**Fig. 2:**
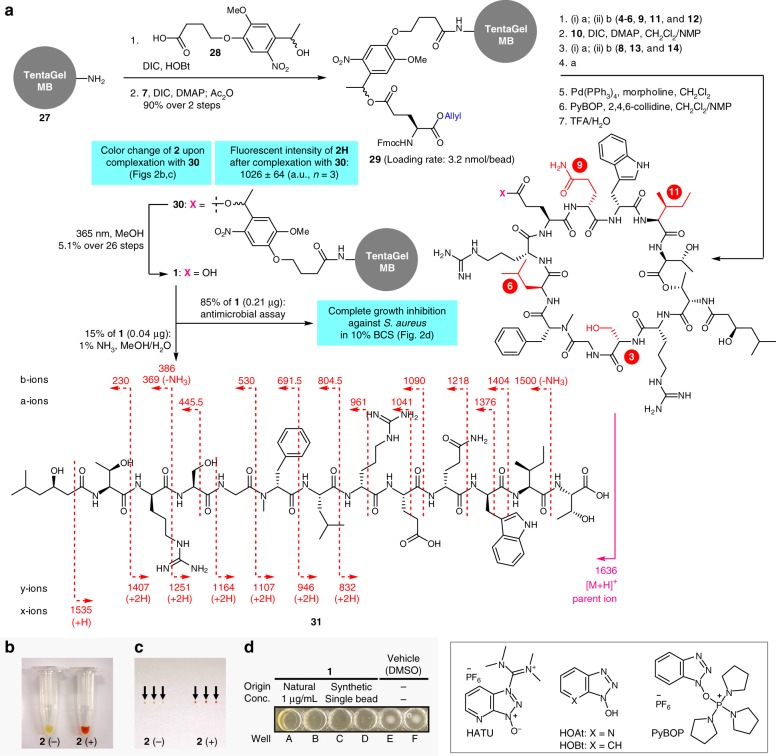
Synthesis and evaluation of bead-linked lysocin E. **a** Synthesis of bead-linked lysocin E **30** and establishment of the OBOC protocols. The fragmentation pattern of hydrolyzed **31** on MALDI-MS/MS is also shown. Reagents and conditions: a 20% piperidine/NMP, b HATU, HOAt, *i*-Pr_2_NEt, NMP, 40 °C (rt for **9**). a.u. arbitrary unit, DIC *N*,*N*’-diisopropylcarbodiimide, DMAP *N*,*N*-dimethyl-4-aminopyridine, HATU *O*-(7-aza-1*H*-benzotriazol-1-yl)-*N*,*N*,*N*’,*N*’-tetramethyluronium hexafluorophosphate, HOAt 1-hydroxy-7-azabenzotriazole; HOBt 1-hydroxybenzotriazole; NMP *N*-methyl-2-pyrrolidone; PyBOP benzotriazol-1-yloxy)tripyrrolidinophosphonium hexafluorophosphate; TFA trifluoroacetic acid. **b** Color of **30** before (**2** (−)) and after (**2** (+)) incubation with **2**. **c** Color of the 3 beads of **2** (−) and **2** (+). Each bead is indicated by an arrow. **d** Growth inhibition of *Staphylococcus aureus* Smith ATCC 13709 caused by natural (0.04 μg per well, wells A and B) and 85% of single bead-derived **1** (wells C and D) in 10% BCS-doped Mueller Hinton Broth (40 μL) as growth medium. DMSO was used as vehicle control (wells E and F). Evaluation of the growth inhibitory activity was conducted as duplicate experiments. BCS bovine calf serum

Randomization of the 4 positions with the 7 amino acid residues would generate the library comprising 2401 (=7^4^) peptides. For the split-and-mix synthesis of the bead-linked peptides, TentaGel^32^ beads were employed as a solid support (Fig. 1b). The polyethyleneglycol (PEG) chains on TentaGel beads would facilitate efficient peptide elongation due to their high swelling property in organic solvents^33,34^, and reduce non-specific binding of hydrophobic molecules such as MK-4 (**2**). Furthermore, a high loading capacity (3.5 nmol per 0.3 mm bead) would provide sufficient sample quantity for the post-synthesis analyses. To maximize the coverage of this library without losing operational efficiency, the 3-fold number of beads (approximately 7510 beads) was to be included (expected coverage of the peptides = 95%)^35^.

An *o*-nitroveratryl linker^36,37^ was chosen on the basis of acid-stability and photocleavable property. The bead-linked peptides can be obtained upon acidic global deprotection, which are readily amenable for the on-bead complexation activity with **2**. Simple light-irradiation would liberate the peptides, which can be subjected to MS/MS sequencing and to the in-solution analysis of their antimicrobial activity.

### Synthesis and evaluation of bead-linked lysocin E

Since one bead contains only less than 1 μg of a peptide, small-scale procedures should be devised prior to construction of the **1**-based OBOC library. Hence, we first aimed to synthesize the parent **1** using TentaGel beads to validate the feasibility of our OBOC strategy.

Preparation of bead-linked **1** started with the conjugation of photocleavable linker **28** with the amine of TentaGel beads **27** (Fig. 2a). The secondary alcohol of the linker was then condensed with the carboxylic acid of Fmoc-Glu-OAllyl **7** to afford **29** in 90% yield over 2 steps (3.2 nmol per bead). The thus-obtained beads were submitted to the Fmoc-based solid-phase peptide synthesis. Amide coupling was conducted at 40 °C under microwave-assisted conditions in NMP to facilitate the synthesis^38,39^. The linear dodecapeptide was elongated from **29** by 7 cycles of piperidine-promoted N_α_-deprotection and HOAt/HATU^40^-mediated amidation (**4**–**6**, **9**, **11**, and **12**), DIC/DMAP-promoted esterification (**10**), and then 3 cycles of N_α_-deprotection and amidation (**8**, **13**, and **14**). The Fmoc group of the N-terminus and the allyl group of the C-terminus of the product were then removed by treatment with piperidine, and subsequently with Pd(PPh_3_)_4_ and morpholine to provide the macrolactam precursor^41^. On-bead cyclization^42^ was then effected using PyBOP/2,4,6-collidine^43^, leading to the 37-membered macrolactam. Finally, treatment of the macrolactam with aqueous TFA detached the side-chain protective groups to furnish the bead-linked lysocin E **30**. Despite its stability under acidic conditions, the linker of **30** was smoothly cleaved by dispersing in MeOH and irradiating ultra-violet light (365 nm), delivering the crude **1**. The overall yield from **29** to **1** over 26 steps was calculated to be 5.1% by MS and ultra-high-performance liquid chromatography (UHPLC) analyses (Supplementary Fig. 1), demonstrating that the efficiency of the procedure was comparable to that of the previous total synthesis (8.0%). Consequently, one bead after the synthesis contained 0.25 μg of **1**.

Having optimized the synthesis of **1** for the OBOC format, we then needed to address the challenges in its structure determination by using only one bead. Sequencing of cyclic peptides is generally problematic, however, because of their complex fragmentation patterns caused by ring-opening at multiple positions^44^. Therefore, to simplify the analysis, the macrocycle of **1** was linearized by exploiting the single ester linkage between l-Thr-1 and -12. Specifically, **1** was treated with 1% NH_3_ in MeOH/H_2_O to generate the linear seco acid **31**. Matrix-assisted laser desorption/ionization (MALDI) MS/MS spectrometry of **31** afforded high-quality fragment ions, matching to the amino-acid sequence of **1**. Because of its high sensitivity, the MS/MS sequencing analysis required only 15% of **1** on one bead (0.04 μg).

Next, the feasibility of the on-bead complexation assay was assessed using bead-linked lysocin E **30** and MK-4 (**2**). Our initial assay based on the finding that **1** and **2** form a red precipitate upon complexation (Supplementary Fig. 2) was not robust^45^, because the magnitude of the red color was not proportional to the amount of captured **2** (Fig. 2b, c). We therefore developed a fluorescence-based method for quantifying **2** by reducing its naphthoquinone moiety to fluorescent hydroquinone **2H**. One bead of **30** was incubated with **2** in MeOH, washed by MeOH in a microplate well, and then the bead-bound **2** was eluted under more forcing hydrophobic conditions (*n*-BuOH at 50 °C). The eluate containing **2** was treated with NaBH_4_ in *n*-BuOH to produce **2H**^46^. The fluorescence intensity of **2H** was evaluated to determine the quantity of **2** bound to one bead of **30**. The method was found with a small margin of error (1026 ± 64 a.u.), indicating its high accuracy (Fig. 2a and Supplementary Fig. 3). Importantly, the structure of **1** remained intact during the complexation assay as judged by UHPLC and mass analyses.

Finally, a microscale antimicrobial assay was developed to evaluate the activity of **1** in solution phase. Since MS/MS-sequencing consumed 15% of the single-bead-derived **1**, only 0.21 μg of **1** corresponding to remaining 85% of the sample could be applied to the assay. The original MIC value (2–4 μg/mL) of **1** against *S. aureus* was, however, too high to reliably detect its activity in a submicrogram scale. Since **1** has potent therapeutic effects in *S. aureus*-infected mice (ED_50_ = 0.5 mg/kg), we assumed that mimicking the in vivo conditions would lower the MIC value of **1**. Screening of the additives indeed revealed that bovine calf serum (BCS) dramatically enhanced the antimicrobial activity of **1** (Supplementary Table 1), although the factors responsible for this remarkable effect remain to be identified. Specifically, the MIC values of **1** against methicillin-susceptible *S. aureus* (MSSA1) and *S. aureus* Smith ATCC 13709 were decreased by 64-fold (0.0625 μg/mL) by adding 10% BCS to the growth medium. By applying these conditions (Fig. 2d), both natural product **1** (0.04 μg, wells A and B) and single-bead-derived **1** (corresponding to 85% of the cleaved peptide sample, wells C and D) were shown to completely inhibit the growth of *S. aureus*, whereas no growth inhibition was observed in the control wells (E and F). Taken together, these established experimental protocols enabled synthesis, structural determination, and functional evaluation by using **1** derived from a single bead.

### Construction of the lysocin E-based OBOC library

**Fig. 3:**
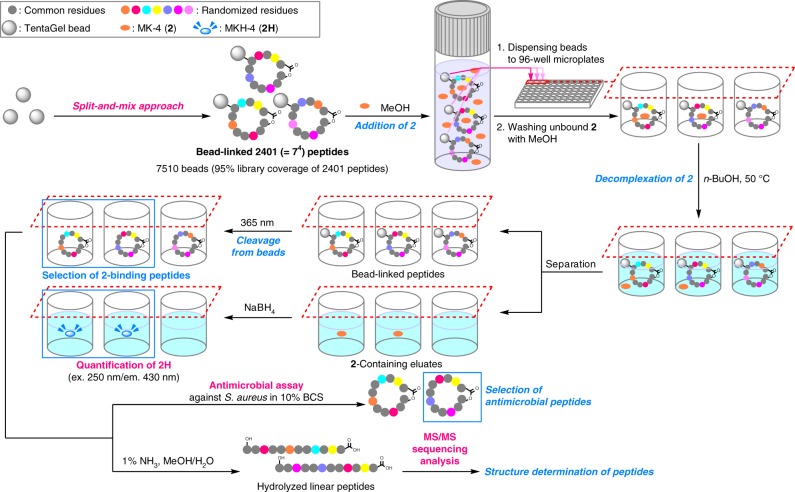
Overview of the OBOC strategy. A high throughput platform for constructing and screening 2401 lysocin E analogues is shown

Having successfully completed the pilot study using **1**, the high throughput scheme for the full-scale OBOC library was planned as shown in Fig. 3. First, the bead-linked 2401 peptides are to be generated by parallel solid-phase syntheses using the split-and-mix approach. Second, **2**-complexation activity is assessed by a fluorescence-based assay for all the beads in a parallel format in the microplate wells. Third, upon photocleavage, 15% aliquot of the single-bead derived peptide is linearized to determine its sequence by MS/MS analysis. Fourth, the remaining 85% aliquot is evaluated for the antimicrobial activity. To facilitate an efficient screening process, only the positive compounds in the second step were to be used for the third and fourth steps. We envisaged that the combined use of on-bead and in-solution assay systems would lead to the rapid discovery of biologically active analogues of **1** that share the **2**-dependent mechanism of action.

**Fig. 4:**
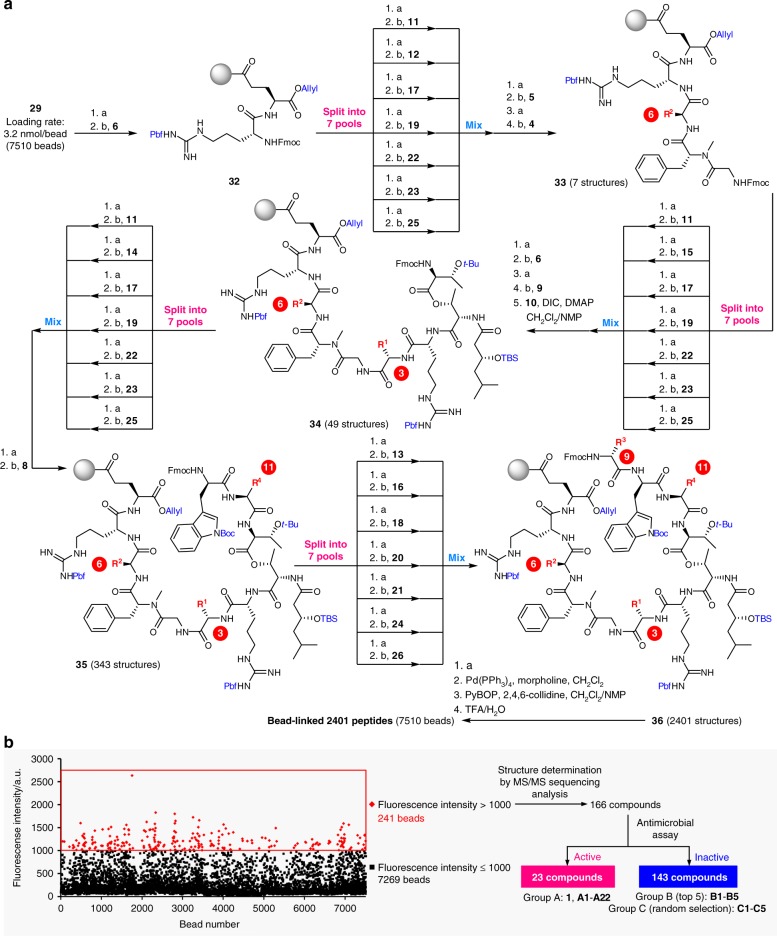
Synthesis and screening of the OBOC library. **a** Synthesis of **1**-based OBOC library comprising 2401 bead-linked peptides. Reagents and conditions: a 20% piperidine/NMP, b HATU, HOAt, *i*-Pr_2_NEt, NMP, 40 °C (rt for **9**). **b** Schematic diagram of selection of the compounds belonging to groups A–C. A dot plot of fluorescence intensity against bead number (1–7510) in the **2**-complexation assay is also displayed. Fluorescence intensity corresponds to the relative amount of **2H**. Beads exhibiting a fluorescence intensity >1000 a.u. are plotted as red dots. Source data are provided as a Source Data file

The OBOC library comprising 2401 peptides was constructed by applying split-and-mix methods at residues-3, -6, -9, and -11 (Fig. 4a). The reagents and conditions for amidation, esterification, macrocyclization, and deprotection were the same as those established in Fig. 2a, while 12 components **15**–**26** were additionally employed for the randomization. Thus, **7**-conjugated **29** consisting of 7510 beads was transformed into N_α_-Fmoc protected dipeptide **32** in 2 steps. In the first randomization step at residue-6, the beads of **32** were divided into 7 pools, which were separately deprotected and condensed with the original (**12**) and 6 different Fmoc amino acids (**11**, **17**, **19**, **22**, **23**, and **25**). Then, all the beads were mixed again and subjected to stepwise chain extension using **5** and **4** to afford pentapeptide **33** containing 7 structures. The second split-and-mix synthesis at residue-3 using the 7 components (**11**, **15**, **17**, **19**, **22**, **23**, and **25**) was followed by one-by-one N_α_-deprotection/condensation of **6**, **9**, and **10**, giving rise to nonapeptide **34** (49 structures). Further attachment of the 3 amino acid residues included randomization at residues-11 and -9 and simple extension at residue-10, leading to linear dodecapeptide **36** (2401 structures). Finally, **36** was collectively converted into the bead-linked 2401 cyclic peptides via sequential base-promoted removal of the Fmoc group, palladium-mediated removal of the allyl group, 37-membered ring cyclization, and on-bead global deprotection. The overall yields of the peptides were assumed to be approximately 2%–12% (0.1–0.6 μg of a peptide per bead) according to the yield of **1** in Fig. 2a and the yields of the re-synthesized compounds in Table 1, although calculation of the precise yields was not feasible at this stage.

**Table 1:** Structures and activities of lysocin E and 32 analogues

*Note*: Compound numbers, one-letter codes of the amino acids at residues-3, -6, -9, and -11, overall yields from Wang resin-linked **7**, color-gradient heat map showing the antimicrobial and membrane-disrupting activities, and selectivities of **2**/**3**

*S. haemolyticus* *Staphylococcus haemolyticus* JCM2416, *B. cereus* *Bacillus cereus* JCM20037, *B. subtilis* *Bacillus subtilis* JCM2499, *L. monocytogenes* *Listeria monocytogenes* 10403S, *LUV* large unilamellar vesicle, *PC* egg yolk phosphatidylcholine, *PG* egg yolk phosphatidylglycerol

^a^MIC values (μg/mL) were determined by the microdilution method

^b^EC_50_ values (nM) were determined by the liposomal membrane disruption assay. Average values of the 3 independent experiments are displayed

^c^PC/PG/**2** = LUVs containing PC and PG (50:50) with 1.25 mol% **2**

^d^PC/PG/**3** = LUVs containing PC and PG (50:50) with 1.25 mol% **3**

^e^PC/PG = LUVs containing PC and PG (50:50)

^f^Selectivity indexes were calculated as EC_50_ against PC/PG/**3** divided by EC_50_ against PC/PG/**2**

^g^Appearance frequency in the one-bead antimicrobial assay was 1 unless otherwise noted

^h^Appearance frequency in the one-bead antimicrobial assay was 2

^i^Appearance frequency in the one-bead antimicrobial assay was 3

^j^MIC value in the presence of 10% BCS

### MK-4–complexation assay and MS/MS-sequencing

An on-bead MK-4 (**2**)–complexation assay using the 7510 beads revealed significantly varied activities (Fig. 4b). Based on the fluorescence intensity of 1026 ± 64 a.u. for **30** carrying the parent lysocin E structure, we defined the 241 beads exhibiting fluorescence intensities of >1000 a.u. (Fig. 4b, red dots) as the hit beads. Approximately top 3% of the entire beads was selected for the subsequent experiments.

Next, the structures of the 241 bead-linked peptides were determined. After releasing the cyclic peptides from the beads by photocleavage, the 15% fraction of each peptide was chemoselectively hydrolyzed to produce the linear peptide. MALDI-MS/MS analysis of 241 linear dodecapeptides showed that 237 afforded clear parent ions and fragmentation patterns, permitting us to unambiguously establish their structures (Supplementary Tables 2–4). As a result, 166 unique structures were identified. Importantly, one of the 166 compounds turned out to be the parent **1**, demonstrating the reliability of the split-and-mix synthesis of thousands of peptides and the validity of the **2**-complexation assay for selecting the active molecules from the mixture.

Finally, the 166 positive compounds identified in the on-bead assay were evaluated in an in-solution antimicrobial assay. We found that 23 compounds including **1** completely suppressed the growth of *S. aureus* Smith ATCC 13709 in 10% BCS-supplemented medium (Supplementary Fig. 4 and Supplementary Tables 2–4). For further analyses, we designated 22 compounds that were positive in the two assays as **A1**–**A22** (group A), and 10 peptides positive in the first assay and negative in the second assay as **B1**–**B5** (group B) and **C1**–**C5** (group C). **B1**–**B5** correspond to the top 5 compounds in the **2**-complexation assay and **C1**–**C5** were randomly selected from the remaining compounds.

### Antimicrobial spectra and membrane-disrupting activity

To validate the OBOC strategy and evaluate the detailed biological activities, the 32 analogues of **1** (**A1**–**A22**, **B1**–**B5**, and **C1**–**C5**) were individually re-synthesized in several milligram scales according to the original solid-phase synthetic route to **1**^19,20^. The crude materials were purified by HPLC to generate **A1**–**A22**, **B1**–**B5**, and **C1**–**C5** in 1.8–12% total yield over 25 steps (Supplementary Tables 5 and 6). The total syntheses of the 32 structurally complex analogues in consistent overall yields (5.6 ± 3.0%) corroborated the robustness and generality of the full-solid phase synthetic route.

The antimicrobial activities of the fully synthetic 32 analogues were evaluated and compared with those of **1** (Table 1). The MIC values were determined by the 2-fold serial dilution procedure against 6 strains of Gram-positive bacteria. In addition, effects of the peptides against MSSA1 were evaluated in the absence or presence of BCS. The compounds in each group A, B, or C are listed in descending order of the activity against MSSA1 in the presence of 10% BCS. When the compounds displayed the same MIC values toward the BCS-supplemented MSSA1, the values for the BCS-unsupplemented MSSA1 were used for their ordering.

Most significantly, the MIC values of the 11 analogues **A1**–**A11** against MSSA1 were found to be 0.015–0.0625 μg/mL upon addition of 10% BCS to the growth medium, thus having activities more potent than or equipotent to that of **1** (0.0625 μg/mL). Among them, the 3 analogues, **A1**, **A2**, and **A3**, showed 4-fold higher antimicrobial activities than the parent natural product **1**. Similarly to **1** (0.25 μg/mL), **A1**–**A3** also displayed high activities against BCS-supplemented MRSA4 (0.25, 0.25, and 0.125 μg/mL, respectively), suggesting their potential utility in the treatment of MRSA infections. In addition, the 7 compounds **A12**–**A18** retained strong antimicrobial effects although somewhat weaker than **1**. On the other hand, the remaining 4 peptides, **A19**–**A22**, in group A have MIC values comparable to or higher than 6.7 ± 3.7 μg/mL, which was the upper threshold of the one-bead antimicrobial assay calculated from the molecular weights and the overall yields of the resynthesized 33 peptides. These false-positive compounds could be selected because of active impurities generated during the split-and-mix synthesis. Consistent with the high-throughput in-solution antimicrobial assay (Fig. 4b), none of the compounds in groups B (**B1**–**B5**) and C (**C1**–**C5**) had the low MIC values. The results that 83% of 23 peptides in group A and 0% of 10 peptides in groups B and C were active therefore validated our screening system for the discovery of multiple active analogues.

We also assessed the antimicrobial activity of the 33 compounds in groups A–C against the 6 Gram-positive bacteria in the absence of BCS. Natural product **1** and 18 analogues **A1**–**A18** were active against all the strains including MRSA4, whereas **A19**–**A22**, **B1**–**B5**, and **C1**–**C5** had only weak or negligible effects on the bacteria. Intriguingly, while BCS enhanced the activities of **A1**–**A14** toward MSSA1 by 16 to 533 times, the additive only increased the activities of **A15**–**A18** by 2–4 fold. The strain selectivity of **A1**–**A18** was found to be similar. They are more active toward *S. haemolyticus* and *B. cereus*, and less active toward *L. monocytogenes*. **A15** and **A17** were slightly different from the other peptides, however, having only weak effects on *L. monocytogenes* (MIC = 64 μg/mL), and on *B. cereus* and *B. subtilis* (MIC = 16 μg/mL), respectively. These results demonstrated that structural alternations of **1** are effective in attenuating the strain selectivity.

To confirm that the synthetic analogues share the mode of action of **1**, we quantified the MK-4 (**2**)-dependent membrane lytic activity of the 33 synthetic peptides as EC_50_ values by employing liposomes as bacterial cell membrane models^47^. Large unilamellar vesicles (LUVs) comprising egg yolk phosphatidylcholine (PC)/egg yolk phosphatidylglycerol (PG) (50:50 ratio) were prepared to mimic the negatively charged surface of bacterial membranes^48^. The PC/PG LUVs were doped with 1.25 mol% of MK-4 (**2**)^49,50^, or with 1.25 mol% of UQ-10 (**3**) to assess the selectivity toward **2** over **3**. The EC_50_ values of **1** for disrupting PC/PG/**2**, PC/PG/**3**, and PC/PG LUVs were determined to be 19.5 nM, 92.0 nM, and 559 nM, respectively (Table 1), confirming the **2**-dependent lysis by **1**. The selectivity index of **1** toward **2** over **3** was calculated to be 4.7 (=92.0/19.5). Remarkably, 25 of the 32 analogues (**A1**–**A20**, **B1**, **B3**, and **C1**–**C3**) had detectable EC_50_ values for the PC/PG/**2** LUVs and selectivity indexes greater than 2.7. Importantly, the 11 most potent antimicrobial compounds **A1**–**A11** displayed membrane-disrupting activities against the PC/PG/**2** LUVs (EC_50_ = 10.7–38.8 nM) comparable to that of **1** (19.5 nM). Thus, it is expected that **1** and **A1**–**A11** share the same **2**-dependent mechanism of action. It is also noteworthy that the selectivity indexes of **A7** (19.7), **A14** (>19.7), and **A16** (20.6) were higher than that of **1** (4.7), demonstrating that the **2**-selectivity can be enhanced by structural alteration of **1**.

**Fig. 5:**
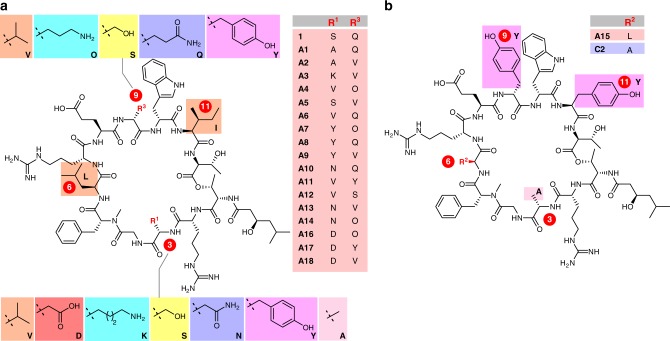
Structures of lysocin E and 19 analogues. **a** Structures of **1** and its antimicrobial analogues **A1**–**A14** and **A16**–**A18**. **b** Structures of antimicrobial analogue **A15** and non-antimicrobial analogue **C2**

These data together allowed us to gather valuable information on the structural requirements for the antimicrobial and **2**-selective membrane-disrupting activities (Fig. 5a). The most remarkable finding is that leucine (L) at residues-6 and isoleucine (I) at residue-11 are both shared among the parent **1**, all the 11 potent analogues **A1**–**A11** and the less active **A12**–**A18** except for **A15**. Thus, these residues are most likely to be essential for the potent antimicrobial functions. As leucine and isoleucine are hydrophobic and bulky, their side chains can interact with hydrophobic tails of bacterial membrane lipids or **2**, and/or organize the bioactive conformation of the peptides^51^. In contrast to the conserved residues-6 and -11, residues-3 and -9 are significantly varied, indicating the less stringent requirement for these substructures for the activity. **A1**–**A3** with the highest antimicrobial activities possess alanine (A) or lysine (K) at residue-3 and valine (V) or glutamine (Q) at residue-9. For **A7**, **A14**, and **A16** with the highest **2**-selectivity, residue-9 is substituted with ornithine (O), implying that ornithine may play specific roles to augment the activity. It is interesting to note that the only difference between the most potent analogue **A1** and **1** is the presence or absence of the hydroxy group at residue-3. **A15** is different from **1** at residues-3 (alanine), -9 (tyrosine), and -11 (tyrosine) (Fig. 5b), suggesting this unique sequence is responsible for insensitivity to the BCS-addition and different bacterial strain selectivity. Since changing the leucine (L) at residue-6 of **A15** to alanine (A) gives inactive analogue **C2**, it confirms the significance of l-Leu-6 for the activity. Overall, the present strategy proved to be effective for deciphering the crucial structural factors for enhancing and modulating the functions of **1**.

## Discussion

We discovered 18 antimicrobial analogues **A1**–**A18** of complex peptide natural product **1** by developing a high throughput method that integrates 26-step solid-phase total synthesis of 2401 cyclic peptides, MS/MS-sequencing, and two different microscale activity assays. Our design strategy for the peptide library involved three criteria. Four amino acid residues of **1** (residues-3, -6, -9, and -11) were chosen as the site of randomization based on the previous SAR data and the synthetic efficiency. The 7 amino-acid components were employed to substitute the sites of randomization in order to explore diverse structures in the library with varying physicochemical properties and to represent unique molecular weight for unambiguous structure determination by MS/MS. The split-and-mix synthesis was achieved in a submicrogram scale to generated the 2401 cyclic peptides on TentaGel beads with a photocleavable linker. The on-bead complexation assay with **2** and MS/MS-sequencing of photocleaved and linearized peptides identified 166 distinct sequences. Based on their in-solution assay against MSSA1 in the presence of BCS, they were further classified into antimicrobial (**1** and **A1**–**A22**, 23 compounds) and non-antimicrobial groups (143 compounds). To validate the results obtained by the OBOC-based high throughput approach, active analogues **A1**–**A22**, and inactive analogues **B1**–**B5** (top 5 of the first assay) and **C1**–**C5** (random extraction) were all re-synthesized in a milligram scale. Among them, 18 compounds, **A1**–**A18**, proved to be active against MSSA1 under the BCS-supplemented conditions, demonstrating the high fidelity and high hit-rate (0.7%) of the developed screening system. Remarkably, **A1**–**A3** had 4-fold higher and comparable effects toward MSSA1 and MRSA4, respectively, indicating their potential superiority to **1** as antibiotic agents. More active **A1**–**A3** and comparably active **A4**–**A11** also exhibited antimicrobial effects against 6 Gram-positive bacteria with selectivity profiles similar to that of **1** and share the **2**-dependent membrane-disrupting functions. The key common structural elements for **A1**–**A18** except for **A15** were l-leucine at residue-6 and l-isoleucine at residue-11, revealing, for the first time, the significance of these hydrophobic amino acids. This SAR studies on a high number of lysocin E analogues provided large data sets that were previously unavailable, and led us to identify potentially superior agents for the treatment of infectious diseases caused by Gram-positive bacteria, including MRSA.

Improving the activities of architecturally complex natural products is extremely challenging often due to lack of SAR information and synthetic accessibility. Thus, the discovery of the more potent synthetic analogues **A1**–**A3**, in particular, highlights the advantage of the present OBOC approach over the conventional one-by-one synthesis or rational design approach. As an OBOC library is extremely useful for rapid exploration of the biologically relevant chemical space around natural products, the overall methodology developed, here will be widely applicable as a promising strategy for extracting key structural features and identifying more biologically active analogues of natural products beyond this study.

## Methods

### General remarks

All reactions sensitive to air and/or moisture were carried out under argon (Ar) atmosphere in dry solvents, unless otherwise noted. CH_2_Cl_2_, DMF, and Et_2_O were purified by a Glass Contour solvent dispensing system (Nikko Hansen). All other reagents were used as supplied unless otherwise stated. Solid-phase peptide synthesis (SPPS) was performed on a microwave-assisted peptide synthesizer MWS-1000 (EYELA) using a sealed reaction vessel, a reaction temperature of which was monitored by an internal temperature probe. Optical rotations were recorded on a P-2200 polarimeter (JASCO). Infrared (IR) spectra were recorded on an FT/IR-4100 spectrometer (JASCO) as a thin film on a CaF_2_. ^1^H and ^13^C NMR spectra were recorded on an ECX 500 (500 MHz for ^1^H NMR) spectrometer, an ECZ 500 R (500 MHz for ^1^H NMR) spectrometer (JEOL), or an Avance III HD 800 MHz equipped with CryoProbe (800 MHz for ^1^H NMR, 200 MHz for ^13^C NMR) (Bruker). Chemical shifts are denoted in *δ* (ppm) relative to residual solvent peaks as internal standard (DMSO-*d*_6_, ^1^H *δ* 2.50, ^13^C *δ* 39.5). HRMS spectra were recorded on a MicrOTOFII (Bruker Daltonics) electrospray ionization time-of-flight (ESI-TOF) mass spectrometer. UV absorbance was measured on a UV-1800 UV-VIS spectrophotometer (Shimadzu). High performance liquid chromatography (HPLC) experiments were performed on a HPLC system equipped with a PU-2089 Plus intelligent pump or a HPLC system equipped with a PU-2086 Plus intelligent pump (JASCO). Ultrahigh-performance liquid chromatography (UHPLC) experiments were performed on a X-LC system (JASCO).

### Experimental data

For MS/MS spectra of compounds **A1**–**A22**, **B1**–**B5**, and **C1**–**C5**, see Supplementary Figs. 10–42. For ^1^H, ^13^C NMR, ^1^H–^1^H DQF-COSY, ^1^H–^1^H TOCSY, ^1^H–^1^H NOESY, ^1^H–^13^C HMBC, and ^1^H–^13^C HSQC of **A1**–**A3**, see Supplementary Figs. 43–54. For chemical shifts of compounds **A1**–**A3**, see Supplementary Tables 5 and 6. For ^1^H NMR spectra of compounds **A4**–**A22**, **B1**–**B5**, and **C1**–**C5**, see Supplementary Figs. 55–69. For HPLC charts for purification of synthetic peptides, see Supplementary Figs. 70–134. For UHPLC charts for purified synthetic peptides, see Supplementary Figs. 135–172. For MS spectra of 241 bead-derived linear peptides, see Supplementary Figs. 173–213. For the experimental procedures and spectroscopic data of compounds, see Supplementary Methods.

### Reporting summary

Further information on research design is available in the Nature Research Reporting Summary linked to this article.

## Supplementary information

Supplementary Information

Reporting Summary

Source Data

## Data Availability

The data that support the findings of this study are available from the corresponding author upon reasonable request. The source data underlying Fig. 4b, and Supplementary Figs. 3, 7, 8, and 9 are provided as a Source Data file.
