# Supplementary material for: Development of a high-throughput strategy for discovery of potent analogues of antibiotic lysocin E
Source: Nat Commun. 2019 Jul 5;10:2992. doi: 10.1038/s41467-019-10754-4 (PMC6611794; doi:10.1038/s41467-019-10754-4)
Supplement: Supplementary file 1 — Supplementary Information [file 41467_2019_10754_MOESM1_ESM.pdf]

Supplementary Information

Development of a High-Throughput Strategy for Discovery of Potent Analogues of Antibiotic Lysocin E

Hiroaki Itoh[1†], Kotaro Tokumoto[1†], Takuya Kaji[1†], Atmika Paudel[2], Suresh Panthee[2], Hiroshi Hamamoto[2], Kazuhisa Sekimizu[2], and Masayuki Inoue*[1]

[1]Graduate School of Pharmaceutical Sciences, The University of Tokyo, 7-3-1 Hongo, Bunkyo-ku, Tokyo 113-0033, Japan.

[2]Teikyo University Institute of Medical Mycology, 359 Otsuka, Hachioji, Tokyo 192-0395, Japan.

[†]These authors contributed equally to this work.

*E-mail: inoue@mol.f.u-tokyo.ac.jp

Table of Contents

**Supplementary Figures**	3
**Supplementary Tables**	7
**Supplementary Methods**	14
**Supplementary References**	160

Supplementary Figures

a

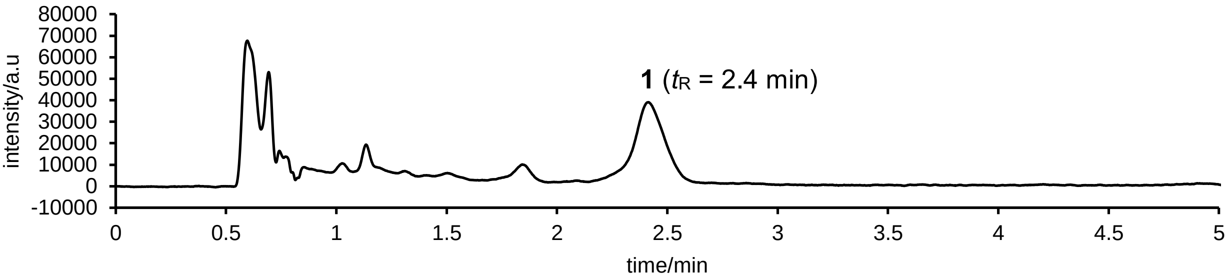

b

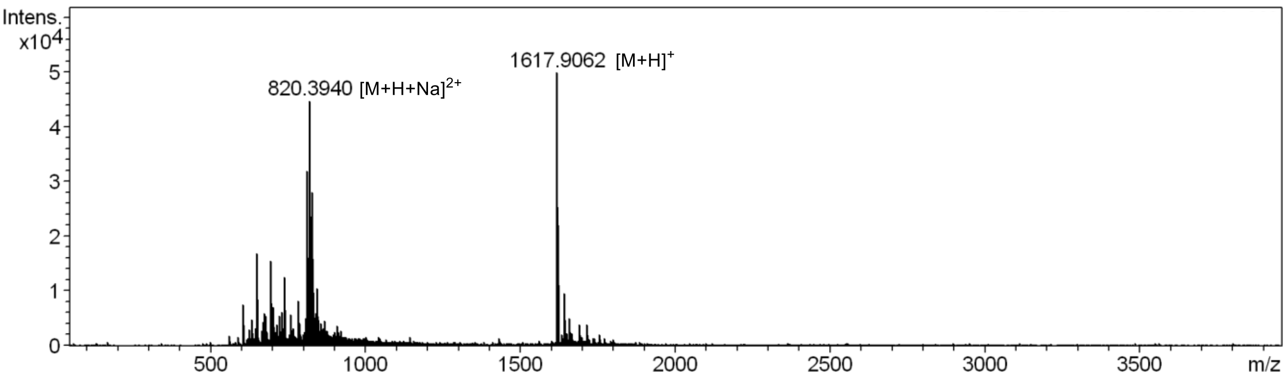

Supplementary Figure 1. UHPLC and MS data of bead-derived lysocin E. (a) UHPLC chart of bead-derived lysocin E (**1**). Column: Accucore C18 2.1 × 150 mm, eluent A: MeCN + 0.05% TFA, eluent B: H_2O + 0.05% TFA, A/B = 40/60, flow rate: 0.40 mL/min, detection: photodiode array detector 200−648 nm (UV chromatogram: 280 nm), temperature: 40 ºC. a.u. = arbitrary unit. (b) ESI-TOF MS spectrum of the crude bead-derived **1**.

a

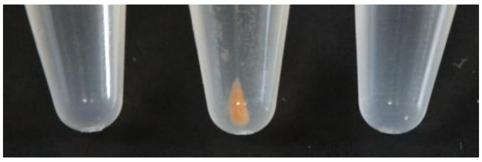

b

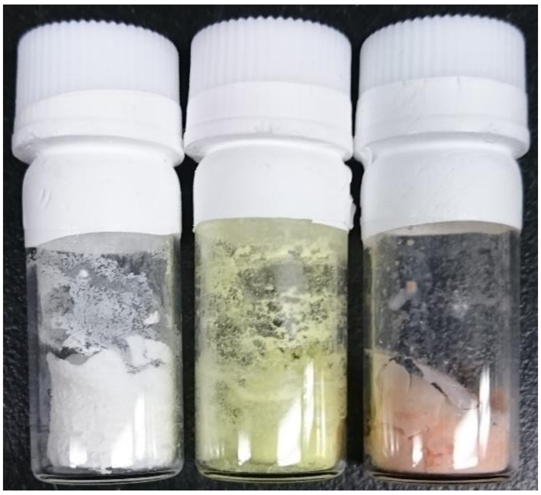

Supplementary Figure 2. Complexation of lysocin E with MK-4. (a) Red precipitation consisting of lysocin E (**1**) and MK-4 (**2**) in Müller Hinton Broth. Left: **2** (12.5 μg/mL), center: **1** (50 μg/mL) and **2** (12.5 μg/mL), right: **1** (50 μg/mL). (b) Color change upon the complexation of **1** with **2**. Left: **1**, center: **2**, right: mixture of **1** and **2** (1:1). All the samples were prepared by lyophilization from CH_3CN/H_2O.

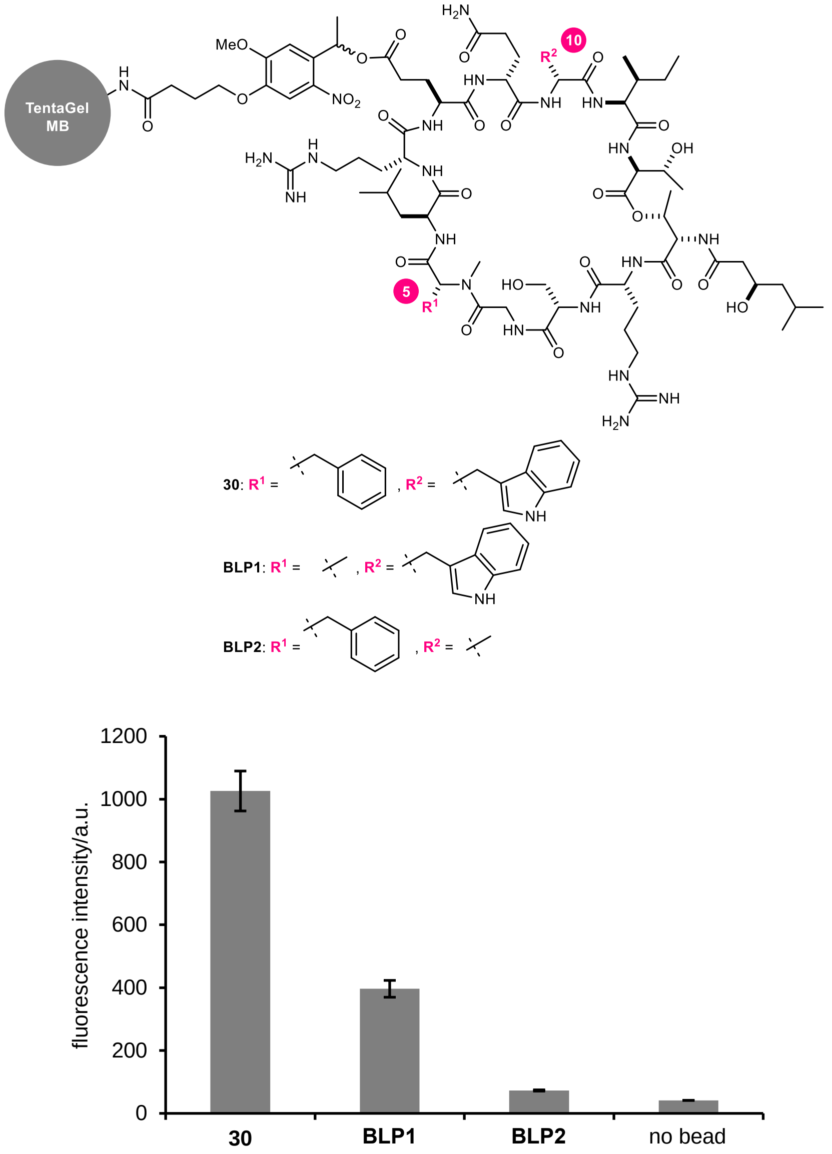

Supplementary Figure 3. Fluorescence intensity of MKH-4 eluted from one bead. no bead = background. The fluorescence intensities (arbitrary unit) are plotted as mean ± SD of three data points. The data showed the importance of the aromatic residues for the complexation with MK-4 (**2**). Source data are provided as a Source Data file.

a

	1	2	3	4	5	6	7	8	9	10	11	12
A	-	-	-	-	-	-	-	-	-	-	-	+
B	-	-	-	-	-	-	-	-	-	-	-	+
C	-	-	-	-	-	-	-	-	-	-	-	+
D	-	-	-	-	-	-	-	-	-	-	++	+
E	-	-	-	+	-	-	-	+	-	-	-	-
F	+	-	-	-	-	-	-	-	-	-	-	-
G	-	-	-	-	-	-	-	-	-	-	+	-
H	-	-	-	-	-	-	+	-	-	-	-	-

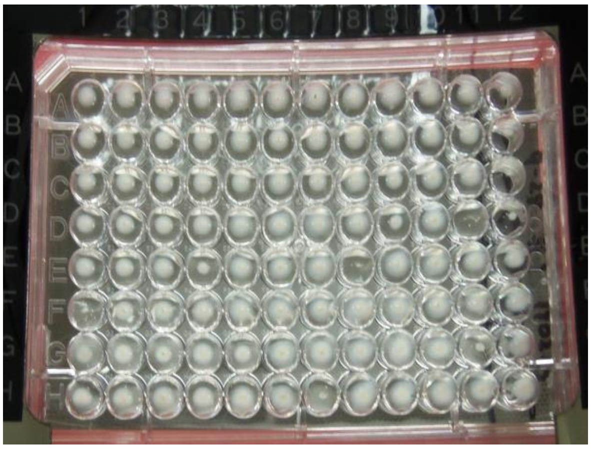

b

	1	2	3	4	5	6	7	8	9	10	11	12
A	-	-	-	-	+	-	-	-	++	-	+	+
B	-	-	++	-	-	-	-	-	-	+	-	++
C	-	-	-	++	-	-	-	-	-	-	-	+
D	-	-	-	-	-	-	-	++	++	-	-	+
E	-	-	-	-	-	-	-	-	-	+	-	-
F	-	+	-	-	-	+	-	-	-	-	-	-
G	-	-	-	-	-	-	-	+	-	++	+	-
H	-	-	-	-	-	-	-	+	+	-	-	-

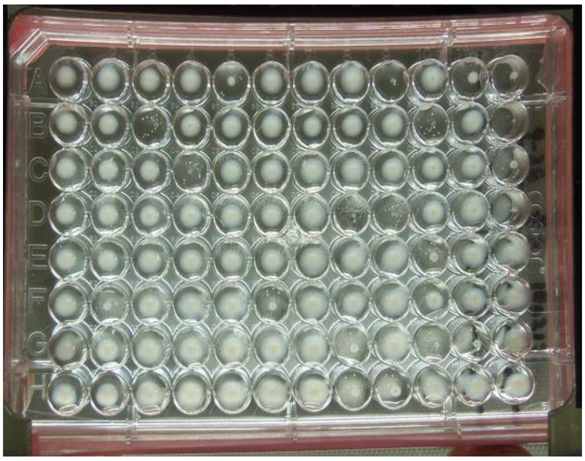

c

	1	2	3	4	5	6	7	8	9	10	11	12
A	-	-	-	-	-	-	-	-	-	+		
B	-	-	-	-	-	-	++	-	-	+		
C	++	-	-	-	-	-	+	-	-	+		
D	+	-	-	++	-	-	-	-	-	++		
E	-	-	-	-	-	-	-	-	-	-		
F	-	-	-	-	-	-	++	-	-	-		
G	-	-	-	-	-	-	+	-	-	-		
H	-	-	+	++	-	-	-	-	-	-		

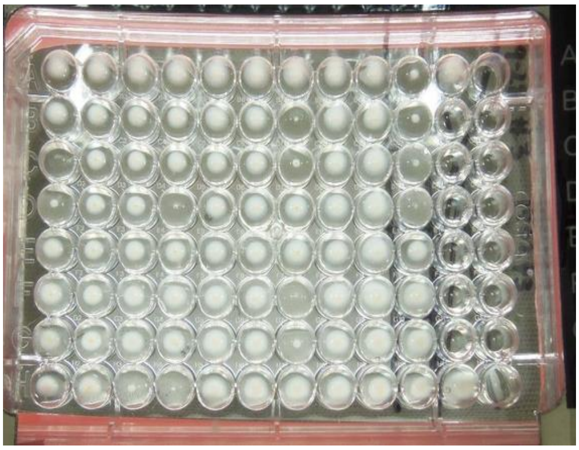

- bead-derived peptide from library
- **1** (0.04 μg)
- **1** (cleaved from one bead of **30**)
- vehicle control (DMSO)
- none

Supplementary Figure 4. Antimicrobial activities of the 241 one-bead-derived peptides. The activities against *S. aureus* Smith ATCC 13709 were evaluated. (a) plate 1. (b) plate 2. (c) plate 3. ++: inhibition, +: partial inhibition, -: no inhibition.

Supplementary Tables

Supplementary Table 1. Enhancement of antimicrobial activity in the presence of 10% BCS

strains	MIC (µg/mL)[a]	
	BCS free	10% BCS (+)
Staphylococcus aureus MSSA1	2.0	0.0625
Staphylococcus aureus Smith ATCC 13709	2.0	0.0625
Staphylococcus aureus RN4220	4.0	0.125

[a]MIC values (µg/mL) were determined by the microdilution method.

Supplementary Table 2. One-bead-derived peptides of the plate 1 in Supplementary Figure 4a[a]

location			residue number				activity	
plate	line	column	3	6	9	11	fluorescence intensity of **2H** (a.u.)	antimicrobial activity
1	A	1	Y	S	A	K	2632.20	-
1	B	1	D	D	Q	Y	1596.58	-
1	C	1	A	L	O	A	1556.97	-
1	D	1	S	Y	A	A	1511.06	-
1	E	1	D	L	O	Y	1471.73	-
1	F	1	N	L	O	I	1470.92	+
1	G	1	V	L	V	Y	1462.14	-
1	H	1	A	L	Q	S	1458.38	-
1	A	2	D	A	D	Y	1445.22	-
1	B	2	Y	Y	Q	I	1434.22	-
1	C	2	S	D	D	I	1364.16	-
1	D	2	D	Y	Q	I	1351.77	-
1	E	2	D	L	O	A	1349.41	-
1	F	2	D	L	V	A	1320.28	-
1	G	2	K	L	Y	D	1315.58	-
1	H	2	D	D	D	S	1299.90	-
1	A	3	D	N	O	D	1293.89	-
1	B	3	D	L	S	A	1293.71	-
1	C	3	ND				1267.94	-
1	D	3	D	L	Y	S	1236.20	-
1	E	3	Y	Y	V	I	1233.44	-
1	F	3	S	L	Y	N	1220.61	-
1	G	3	V	L	V	Y	1213.90	-
1	H	3	V	Y	V	I	1201.71	-
1	A	4	V	L	V	Y	1195.40	-
1	B	4	Y	A	S	I	1189.05	-
1	C	4	D	L	S	Y	1185.06	-
1	D	4	D	L	V	A	1178.76	-
1	E	4	D	L	V	I	1177.94	+
1	F	4	D	L	Y	Y	1173.25	-
1	G	4	A	Y	D	I	1172.65	-
1	H	4	S	L	D	S	1172.60	-
1	A	5	S	Y	Q	Y	1170.65	-
1	B	5	S	S	V	I	1168.39	-
1	C	5	D	D	V	Y	1161.82	-
1	D	5	Y	Y	Q	I	1159.71	-
1	E	5	Y	Y	D	S	1151.29	-
1	F	5	D	A	Q	I	1149.49	-
1	G	5	V	L	O	A	1144.18	-
1	H	5	Y	L	V	Y	1141.61	-
1	A	6	A	D	D	Y	1141.41	-
1	B	6	D	S	Y	I	1140.47	-
1	C	6	D	L	D	A	1127.49	-
1	D	6	V	L	S	Y	1118.24	-

location			residue number				activity	
plate	line	column	3	6	9	11	fluorescence intensity of **2H** (a.u.)	antimicrobial activity
1	E	6	D	Y	A	Y	1117.16	-
1	F	6	N	D	Q	I	1116.52	-
1	G	6	V	L	A	D	1116.32	-
1	H	6	A	L	Q	N	1106.06	-
1	A	7	S	L	Q	A	1105.68	-
1	B	7	D	S	V	I	1105.32	-
1	C	7	D	A	Y	I	1104.53	-
1	D	7	V	Y	Q	I	1101.96	-
1	E	7	D	L	Y	N	1097.32	-
1	F	7	N	L	O	N	1093.36	-
1	G	7	D	L	V	Y	1081.13	-
1	H	7	N	L	Q	I	1074.12	+
1	A	8	V	L	Y	S	1073.12	-
1	B	8	S	S	Y	S	1061.00	-
1	C	8	Y	A	Q	I	1054.47	-
1	D	8	D	Y	V	I	1053.52	-
1	E	8	S	L	V	I	1051.42	+
1	F	8	Y	L	D	N	1050.24	-
1	G	8	Y	D	Q	I	1049.20	-
1	H	8	V	L	A	A	1045.10	-
1	A	9	K	Y	Q	I	1040.86	-
1	B	9	D	Y	Q	A	1035.57	-
1	C	9	V	A	O	I	1027.02	-
1	D	9	D	L	Q	I	1025.79	-
1	E	9	D	A	O	Y	1014.70	-
1	F	9	D	K	V	Y	1011.67	-
1	G	9	D	L	Y	D	1825.82	-
1	H	9	D	L	O	Y	1798.69	-
1	A	10	D	L	V	A	1723.17	-
1	B	10	Y	L	D	N	1659.43	-
1	C	10	Y	A	D	I	1644.29	-
1	D	10	D	L	S	Y	1622.17	-
1	E	10	D	L	A	S	1590.33	-
1	F	10	Y	Y	O	I	1578.11	-
1	G	10	A	Y	V	I	1508.12	-
1	H	10	D	A	V	I	1488.78	-
1	A	11	A	D	Q	I	1462.30	-
1	B	11	S	L	Q	S	1455.14	-
1	C	11	V	Y	V	I	1449.55	-
1	D	11	K	L	V	I	1439.45	++
1	E	11	S	A	Y	A	1425.54	-
1	F	11	D	Y	Y	A	1421.23	-
1	G	11	D	L	O	I	1414.45	+
1	H	11	D	D	V	N	1396.80	-

[a]The structures of the residues-3, -6, -9, and -11 are displayed as one-letter codes of the amino acids. Fluorescence intensity of MKH-4 (**2H**) in menaquinone complexation assay and antimicrobial activity are also listed. ++: inhibition, +: partial inhibition, -: no inhibition, ND = The structure was not determined due to the low intensity of the MS spectrum.

Supplementary Table 3. One-bead-derived peptides of the plate 2 in Supplementary Figure 4b[a]

location			residue number				activity	
plate	line	column	3	6	9	11	fluorescence intensity of **2H** (a.u.)	antimicrobial activity
2	A	1	A	Y	S	I	1372.55	-
2	B	1	D	D	V	Y	1368.46	-
2	C	1	Y	L	D	S	1342.18	-
2	D	1	Y	A	Q	I	1339.68	-
2	E	1	D	A	V	I	1321.70	-
2	F	1	ND				1311.54	-
2	G	1	D	D	A	S	1303.04	-
2	H	1	D	L	V	I	1302.73	-
2	A	2	Y	Y	Y	Y	1285.36	-
2	B	2	Y	L	A	A	1281.70	-
2	C	2	V	L	V	K	1276.24	-
2	D	2	S	L	O	Y	1273.94	-
2	E	2	A	L	Q	A	1271.37	-
2	F	2	A	L	Q	I	1237.12	+
2	G	2	D	D	Q	I	1230.93	-
2	H	2	A	D	Y	I	1221.12	-
2	A	3	D	S	V	I	1216.90	-
2	B	3	V	L	O	I	1209.94	++
2	C	3	Y	L	V	N	1208.97	-
2	D	3	A	D	D	I	1208.67	-
2	E	3	D	A	D	I	1207.35	-
2	F	3	K	L	D	D	1204.49	-
2	G	3	D	A	A	D	1192.95	-
2	H	3	Y	L	V	N	1185.83	-
2	A	4	D	D	V	I	1184.74	-
2	B	4	S	L	Y	D	1174.96	-
2	C	4	A	L	V	I	1152.24	++
2	D	4	Y	D	V	I	1150.09	-
2	E	4	D	Y	Y	I	1132.50	-
2	F	4	D	L	V	D	1125.03	-
2	G	4	S	D	Q	I	1122.33	-
2	H	4	D	L	Q	N	1121.63	-
2	A	5	D	L	S	I	1111.46	+
2	B	5	ND				1108.62	-
2	C	5	D	A	D	I	1102.51	-
2	D	5	D	D	V	I	1098.20	-
2	E	5	S	Y	V	I	1095.54	-
2	F	5	D	L	D	A	1095.52	-
2	G	5	D	Y	Q	I	1090.46	-
2	H	5	D	L	A	K	1086.60	-
2	A	6	D	L	O	N	1086.35	-
2	B	6	N	L	A	Y	1078.71	-
2	C	6	S	D	D	I	1076.24	-
2	D	6	D	L	O	Y	1076.00	-

location			residue number				activity	
plate	line	column	3	6	9	11	fluorescence intensity of **2H** (a.u.)	antimicrobial activity
2	E	6	V	L	Q	Y	1075.93	-
2	F	6	V	L	S	I	1062.93	+
2	G	6	V	L	O	Y	1058.87	-
2	H	6	D	L	O	N	1046.77	-
2	A	7	S	Y	S	I	1044.46	-
2	B	7	Y	L	V	Y	1040.64	-
2	C	7	ND				1034.86	-
2	D	7	A	Y	V	I	1027.72	-
2	E	7	A	L	Q	N	1018.63	-
2	F	7	D	A	Y	I	1017.95	-
2	G	7	S	L	Y	D	1016.84	-
2	H	7	D	L	V	I	1012.63	-
2	A	8	D	Y	V	Y	1005.96	-
2	B	8	Y	Y	S	I	1005.43	-
2	C	8	D	L	V	Y	1564.46	-
2	D	8	D	L	V	Y	1488.30	++
2	E	8	S	Y	V	I	1482.25	-
2	F	8	D	L	V	I	1425.64	-
2	G	8	D	L	Y	I	1396.02	+
2	H	8	K	L	V	I	1379.53	+
2	A	9	S	L	Q	I	1369.78	++
2	B	9	D	L	D	Y	1345.74	-
2	C	9	Y	L	D	D	1290.34	-
2	D	9	N	L	Q	I	1282.12	++
2	E	9	D	Y	V	I	1269.51	-
2	F	9	D	A	D	I	1256.95	-
2	G	9	S	L	Q	S	1223.00	-
2	H	9	N	L	Q	I	1219.94	+
2	A	10	N	Y	Y	Y	1216.99	-
2	B	10	Y	L	V	I	1212.44	+
2	C	10	Y	L	V	D	1209.50	-
2	D	10	D	Y	Y	I	1205.90	-
2	E	10	A	N	V	K	1199.78	+
2	F	10	V	L	D	I	1163.48	-
2	G	10	V	L	Y	I	1146.74	++
2	H	10	D	A	Y	I	1146.74	-
2	A	11	A	L	Y	Y	1125.25	+
2	B	11	D	L	O	A	1116.86	-
2	C	11	Y	L	D	D	1105.77	-
2	D	11	S	Y	Y	I	1105.44	-
2	E	11	D	L	Q	S	1102.86	-
2	F	11	N	L	A	Y	1095.23	-
2	G	11	V	L	Q	I	1073.64	+
2	H	11	D	L	D	S	1059.05	-

[a]The structures of the residues-3, -6, -9, and -11 are displayed as one-letter codes of the amino acids. Fluorescence intensity of MKH-4 (**2H**) in menaquinone complexation assay and antimicrobial activity are also listed. ++: inhibition, +: partial inhibition, -: no inhibition, ND = The structure was not determined due to the low intensity of the MS spectrum.

Supplementary Table 4. One-bead-derived peptides of the plate 3 in Supplementary Figure 4c[a]

location			residue number				activity	
plate	line	column	3	6	9	11	fluorescence intensity of **2H** (a.u.)	antimicrobial activity
3	A	1	Y	Y	V	I	1056.92	-
3	B	1	Y	L	Q	Y	1049.95	-
3	C	1	D	L	V	I	1048.91	++
3	D	1	Y	L	O	I	1035.66	+
3	E	1	A	L	A	Y	1034.30	-
3	F	1	V	L	S	Y	1022.53	-
3	G	1	V	A	S	I	1746.55	-
3	H	1	D	Y	Y	A	1700.89	-
3	A	2	D	L	D	Y	1695.50	-
3	B	2	S	N	V	I	1693.66	-
3	C	2	D	D	V	A	1588.77	-
3	D	2	S	L	V	N	1561.06	-
3	E	2	D	A	V	I	1510.62	-
3	F	2	Y	L	A	A	1453.19	-
3	G	2	V	N	V	I	1373.59	-
3	H	2	Y	L	Q	Y	1351.17	-
3	A	3	A	L	V	D	1340.18	-
3	B	3	D	L	A	D	1339.60	-
3	C	3	Y	A	D	I	1335.16	-
3	D	3	D	Y	D	I	1331.59	-
3	E	3	V	S	Y	Y	1330.47	-
3	F	3	D	L	S	I	1328.51	-
3	G	3	A	A	Y	Y	1278.46	-
3	H	3	Y	L	Q	I	1276.04	+
3	A	4	S	S	V	I	1274.21	-
3	B	4	A	D	Y	I	1265.73	-
3	C	4	D	Y	Y	I	1262.82	-
3	D	4	Y	L	V	I	1246.57	++
3	E	4	D	L	Q	S	1209.56	-
3	F	4	Y	Y	O	I	1209.45	-
3	G	4	D	Y	V	Y	1209.43	-
3	H	4	N	L	V	I	1207.23	++
3	A	5	V	A	V	D	1197.28	-
3	B	5	D	A	V	Y	1195.02	-
3	C	5	Y	Y	D	I	1186.32	-
3	D	5	V	Y	Q	I	1185.64	-
3	E	5	A	L	Q	S	1173.75	-
3	F	5	Y	Y	V	I	1173.03	-
3	G	5	V	A	S	I	1171.62	-
3	H	5	V	A	Q	A	1156.57	-
3	A	6	A	A	V	I	1155.05	-
3	B	6	V	Y	O	I	1154.51	-
3	C	6	S	A	S	I	1151.61	-
3	D	6	D	D	Q	I	1145.16	-
3	E	6	Y	L	O	Y	1140.16	-
3	F	6	Y	A	D	S	1136.07	-
3	G	6	A	D	D	I	1131.04	-
3	H	6	S	A	S	D	1127.96	-
3	A	7	V	L	O	N	1127.04	-
3	B	7	Y	L	O	I	1123.94	++
3	C	7	Y	L	S	I	1111.58	+
3	D	7	V	K	O	I	1099.22	-
3	E	7	A	L	S	Y	1096.47	-
3	F	7	V	L	Y	I	1094.22	++
3	G	7	S	L	V	I	1087.21	+
3	H	7	S	D	S	I	1075.31	-
3	A	8	V	A	S	A	1065.39	-
3	B	8	A	Y	V	Y	1060.70	-
3	C	8	A	L	V	D	1054.38	-
3	D	8	D	A	D	I	1043.60	-
3	E	8	D	L	V	A	1031.88	-
3	F	8	A	Y	A	I	1027.34	-
3	G	8	S	A	O	I	1024.78	-
3	H	8	V	L	D	D	1009.80	-
3	A	9	Y	A	V	I	1007.32	-

[a]The structures of the residues-3, -6, -9, and -11 are displayed as one-letter codes of the amino acids. Fluorescence intensity of MKH-4 (**2H**) in menaquinone complexation assay and antimicrobial activity are also listed. ++: inhibition, +: partial inhibition, -: no inhibition.

Supplementary Table 5. Comparison of ^{1}H NMR chemical shifts of lysocin E and **A1–A3**[a]

residue	position	**1**	**A1**[b]	**A2**[b]	**A3**[b]
3-OH-5-Me-	H2	2.32	2.30	2.29	2.29
hexanoic acid	H3	3.86	3.85	3.84	3.84
	H4	1.09 1.30	1.11 1.27	1.07 1.23	1.07 1.22
	H5	1.69	1.69	1.67	1.66
	H6	0.79	0.79	0.75	0.75
	H7	0.79	0.79	0.74	0.75
L-Thr-1	NHα	7.90	7.89	8.02	8.04
	Hα	4.89	4.91	5.00	4.99
	Hβ	4.97	4.91	4.81	4.82
	Hγ	1.07	1.07	1.11	1.09
D-Arg-2	NHα	7.60	7.58	7.54	7.53
	Hα	4.60	4.66	4.70	4.69
	Hβ	1.70	1.70	1.70	1.68
	Hγ	1.47	1.49	1.48	1.49
	Hδ	3.11	3.14	3.13	3.14
	NHδ	7.44	7.59	7.60	7.59
L-Ser-3 (**1**)	NHα	8.66	8.72	8.85	8.82
L-Ala-3 (**A1** and **A2**)	Hα	4.96	4.96	5.03	4.86
L-Lys-3 (**A3**)	Hβ	3.63	1.29	1.30	1.63 1.71
	Hγ				1.36
	Hδ				1.58
	Hε				2.76
	NHε				7.83
Gly-4	NHα	8.30	8.27	8.30	8.30
	Hα	3.87 3.89	3.88	3.86 3.91	3.81 3.91
N-Me-D-Phe-5	NMeα	2.56	2.54	2.51	2.51
	H2	4.28	4.25	4.16	4.21
	H3	3.03 3.29	3.03 3.30	3.03 3.30	3.00 3.29
	H5, H9	7.18	7.18	7.17	7.16
	H6, H8	7.28	7.27	7.29	7.28
	H7	7.21	7.21	7.22	7.22
L-Leu-6	NHα	7.71	7.75	7.74	7.72
	Hα	4.23	4.24	4.25	4.26
	Hβ	1.34 1.50	1.37 1.53	1.34 1.55	1.32 1.53
	Hγ	1.70	1.49	1.47	1.44
	Hδ	0.71	0.72	0.71	0.70
	Hδ'	0.70	0.71	0.67	0.66

residue	position	**1**	**A1**[b]	**A2**[b]	**A3**[b]
D-Arg-7	NHα	8.28	8.40	8.60	8.71
	Hα	4.39	4.66	4.72	4.71
	Hβ	1.66	1.70	1.64 1.67	1.62 1.68
	Hγ	1.57	1.49	1.53	1.49
	Hδ	3.13	3.14	3.14	3.13
	NHδ	7.48	7.59	7.54	7.64
L-Glu-8	NHα	8.37	8.32	8.47	8.52
	Hα	4.52	4.52	4.98	4.98
	Hβ	1.83	1.81 1.83	1.78 1.89	1.76 1.88
	Hγ	2.10	2.12	2.20	2.19
D-Gln-9 (**1** and **A1**)	NHα	8.35	8.46	8.29	8.23
D-Val-9 (**A2** and **A3**)	Hα	4.83	4.84	4.39	4.37
	Hβ	1.73 1.86	1.77 1.87	1.89	1.88
	Hγ	2.20	2.19	0.86	0.86
	Hγ'			0.86	0.85
	NHδ	6.78 7.30	6.77 7.29		
D-Trp-10	NHα	8.05	8.73	8.77	8.76
	H2	4.62	5.16	5.28	5.26
	H3	2.97 3.11	2.95 3.11	2.94 3.12	2.93 3.11
	H5	7.12	7.12	7.11	7.12
	NH_{indole}	10.56	10.56	10.45	10.47
	H7	7.48	7.46	7.42	7.42
	H8	6.92	6.92	6.91	6.92
	H9	6.99	6.99	6.97	6.98
	H10	7.30	7.30	7.29	7.29
L-Ile-11	NHα	7.96	7.94	7.86	7.83
	Hα	4.12	4.14	4.18	4.17
	Hβ	1.48	1.50	1.42	1.40
	Hγ (CH_3)	0.62	0.66	0.67	0.66
	Hγ' (CH_2)	0.91 1.29	0.82 1.17	0.81 1.19	0.79 1.16
	Hδ	0.64	0.65	0.64	0.63
L-Thr-12	NHα	7.30	7.32	7.18	7.17
	Hα	4.22	4.20	4.28	4.31
	Hβ	4.05	4.05	4.12	4.12
	Hγ	0.98	0.99	1.00	1.00

[a]The spectra of lysocin E (**1**, 500 MHz, ref. 15), **A1** (39 mM, 800 MHz), **A2** (22 mM, 800 MHz), and **A3** (21 mM, 800 MHz) were obtained in DMSO-d_6 at 50 °C. The spectra of **A1**, **A2**, and **A3** were recorded on Bruker Avance III HD equipped with CryoProbe. [b]Multiple conformers were observed. Chemical shifts of the major conformers were shown.

Supplementary Table 6. Comparison of ^{13}C NMR chemical shifts of lysocin E and **A1–A3**[a]

1	A1	A2	A3[b]
10.6	10.5	10.4	10.4
14.9	14.9	14.8	14.8
16.4	16.3	16.2	16.3
19.8	18.7	18.3	18.3
20.9	19.8	18.7	18.7
21.7	20.8	18.8	19.8
22.7	21.7	19.8	20.8
23.1	22.7	20.8	21.6
23.8	23.1	21.6	22.2
24.1	23.8	22.7	22.7
24.2	24.1	23.0	23.0
24.6	24.2	23.7	23.7
24.6	24.5	24.1	24.0
28.5	24.7	24.3	24.3
28.8	28.4	24.3	24.5
29.6	28.6	24.7	24.8
29.9	29.3	28.8	26.5
29.9	30.0	29.0	28.7
30.3	30.0	29.9	28.9[c]
31.3	30.0	29.9	29.8
33.8	31.3	30.6	30.0
36.0	33.8	31.6	30.6
36.1	36.1	33.7	31.6[c]
40.2	36.1	36.5	31.8
40.3	39.3[c]	36.5	33.8
40.3	40.2	39.1[c]	36.5
42.3	40.3	40.2	38.5
43.8	42.0	40.3	39.3
46.1	43.7	42.0	40.1
51.0	46.0	43.6	40.2
51.3	47.6	45.9	41.9[c]
51.5	51.1	47.6	43.6
51.8	51.2	51.1	45.9
51.8	51.4	51.1	51.0
52.5	51.4	51.3	51.0
54.5	51.7	51.3	51.2
55.4	51.8	51.6	51.2
57.9	52.4	51.6	51.6
58.1	55.4	55.4	51.6
58.5	57.8	57.3	52.0
62.1	58.2	57.7	55.5
65.5	65.5	57.8	57.3
66.4	66.4	65.4	57.6
71.7	71.5	66.5	57.8
109.2	109.2	72.1[c]	65.4
111.1	111.0	109.3	66.5
118.0	118.0	111.0	72.0[c]
118.1	118.1	117.9	109.3
120.7	120.7	118.1	111.0
123.0	122.9	120.7	117.9
126.2	126.1	122.7	118.1
127.2	127.2	126.1	120.7
128.2	128.1	127.2	122.7
128.2	128.1	128.2	126.1
129.0	129.0	128.2	127.3
129.0	129.0	129.0	128.1
135.9	135.9	129.0	128.1
138.4	138.4	135.8	128.9
156.7	156.8	138.5	128.9
156.7	156.8	156.8	135.8
168.0	167.9	156.8	138.4
169.0	168.9	167.7	156.8
169.1	169.1	169.0	156.8
169.7	169.4	169.1	167.8
169.9	170.3	169.3	169.0
170.4	170.3	170.1	169.0
170.5	170.4	170.1 or 170.3	169.3
170.8	170.7	170.3	170.3
170.9	171.0	170.3	170.3
171.1	171.3	170.3	170.3
171.6	171.5	171.2	170.3
172.0	172.0	171.5	170.5
172.6	172.2	172.0	171.1
173.6	173.4	172.3	171.4
174.0	173.9	173.5	171.5
			172.0
			173.5

[a]The spectra of lysocin E (**1**, 125 MHz, ref. 15), **A1** (39 mM, 200 MHz), **A2** (22 mM, 200 MHz), and **A3** (21 mM, 200 MHz) were obtained in DMSO-d_6 at 50 °C. The spectra of **A1**, **A2**, and **A3** were recorded on Bruker Avance III HD equipped with CryoProbe. [b]Chemical shift of NMeα of N-Me-D-Phe-5 was not determined due to the broaden peak. [c]Chemical shifts were determined from the ^{1}H–^{13}C HSQC spectrum.

Supplementary Table 7. EC_{50} values of liposomal membrane disruption assay

compounds	EC_{50} (nM)[a]		
	PC/PG/**2**	PC/PG/**3**	PC/PG
**1**	19.5 ± 13.3	92.0 ± 64.6	559 ± 118
**A1**	12.2 ± 11.9	92.7 ± 31.3	975 ± 195
**A2**	18.0 ± 13.3	93.4 ± 38.0	714 ± 188
**A3**	10.7 ± 3.5	103 ± 85	822 ± 234
**A4**	30.3 ± 16.6	215 ± 69	1170 ± 60
**A5**	15.5 ± 11.4	159 ± 166	547 ± 172
**A6**	16.1 ± 6.5	98.9 ± 33.5	851 ± 320
**A7**	22.4 ± 6.7	441 ± 136	1120 ± 200
**A8**	11.5 ± 5.1	120 ± 21	1380 ± 120
**A9**	20.4 ± 16.9	94.7 ± 43.5	>1500
**A10**	38.8 ± 20.0	384 ± 166	>1500
**A11**	19.4 ± 11.4	55.3 ± 8.8	651 ± 299
**A12**	46.4 ± 28.8	284 ± 221	>1500
**A13**	19.3 ± 20.1	124 ± 71	>1500
**A14**	76.3 ± 43.1	>1500	>1500
**A15**	35.4 ± 24.6	268 ± 102	1010 ± 210
**A16**	40.3 ± 27.1	832 ± 243	>1500
**A17**	253 ± 160	>1500	>1500
**A18**	381 ± 183	>1500	>1500
**A19**	29.0 ± 8.9	480 ± 27	>1500
**A20**	548 ± 197	>1500	>1500
**A21**	1080 ± 460	>1500	>1500
**A22**	>1500	>1500	>1500
**B1**	102 ± 61	809 ± 181	>1500
**B2**	>1500	>1500	>1500
**B3**	354 ± 80	>1500	>1500
**B4**	>1500	>1500	>1500
**B5**	>1500	>1500	>1500
**C1**	52.4 ± 13.2	703 ± 145	>1500
**C2**	155 ± 49	>1500	>1500
**C3**	155 ± 49	>1500	>1500
**C4**	>1500	>1500	>1500
**C5**	>1500	>1500	>1500

[a]The values are displayed as mean ± SD of three independent experiments. Source data are provided as a Source Data file.

Supplementary Methods

General remarks. All reactions sensitive to air and/or moisture were carried out under argon (Ar) atmosphere in dry solvents, unless otherwise noted. CH_2Cl_2, DMF, and Et_2O were purified by a Glass Contour solvent dispensing system (Nikko Hansen). All other reagents were used as supplied unless otherwise stated. Solid-phase peptide synthesis (SPPS) was performed on a microwave-assisted peptide synthesizer MWS-1000 (EYELA) using a sealed reaction vessel, a reaction temperature of which was monitored by an internal temperature probe. Optical rotations were recorded on a P-2200 polarimeter (JASCO). Infrared (IR) spectra were recorded on an FT/IR-4100 spectrometer (JASCO) as a thin film on a CaF_2. ^1H and ^{13}C NMR spectra were recorded on an ECX 500 (500 MHz for ^1H NMR) spectrometer, an ECZ 500R (500 MHz for ^1H NMR) spectrometer (JEOL), or an Avance III HD 800 MHz equipped with CryoProbe (800 MHz for ^1H NMR, 200 MHz for ^{13}C NMR) (Bruker). Chemical shifts are denoted in δ (ppm) relative to residual solvent peaks as internal standard (DMSO-d_6, ^1H δ 2.50, ^{13}C δ 39.5). HRMS spectra were recorded on a MicrOTOFII (Bruker Daltonics) electrospray ionization time-of-flight (ESI-TOF) mass spectrometer. UV absorbance was measured on a UV-1800 UV-VIS spectrophotometer (Shimadzu). High performance liquid chromatography (HPLC) experiments were performed on a HPLC system equipped with a PU-2089 Plus intelligent pump or a HPLC system equipped with a PU-2086 Plus intelligent pump (JASCO). Ultrahigh-performance liquid chromatography (UHPLC) experiments were performed on a X-LC system (JASCO).

Preparation of 29. The TentaGel Macrobeads (**27**, 26−28 μmol, 0.25 mol/g, 65550 beads/g) in 5 mL LibraTube (Hipep Laboratories) were washed with DMF (1.00 mL × 3). To the beads in the LibraTube were added a solution of hydroxylethyl photolinker (**28**, 3 eq) in DMF (500 μL), *N,N'*-diisopropylcarbodiimide (3 eq), and 1-hydroxybenzotriazole (HOBt, 3 eq) at room temperature. After being stirred at room temperature for 2 h, the mixture was filtered and washed with DMF (1.00 mL × 3) to give the photolinker-bound resin.

To the above photolinker-bound resin in the LibraTube was added a solution of Fmoc-L-Glu-OAllyl (**7**, 5 eq) in DMF (1.00 mL), *N,N'*-diisopropylcarbodiimide (5 eq), and 4-(*N,N*-dimethylamino)pyridine (0.5 eq) at room temperature. After being stirred at room temperature for 12 h, the reaction mixture was filtered, and washed with DMF (1.00 mL × 3) and CH_2Cl_2 (1.00 mL × 3) to give the preloaded beads.

To the above preloaded beads was added Ac_2O/CH_2Cl_2 (1/3, 1.00 mL) at room temperature for the capping of remaining hydroxy groups. After being stirred at room temperature for 30 min, the reaction mixture was filtered, washed with CH_2Cl_2 (1.00 mL × 3), MeOH (1.00 mL × 3), and Et_2O (1.00 mL × 3), and dried under vacuum to give resin **29**.

Determination of the loading rate. Fmoc-protected resin was treated with piperidine/DMF (1/4, 100 μL) at room temperature for 30 min. The reaction mixture was diluted with DMF (2.90 mL). UV absorption at 301 nm of the resultant mixture was measured. The background absorbance was canceled by subtracting the control absorbance obtained from a solution of piperidine/DMF (1/4). The loading rate (*x* mmol/g) was determined by the following Supplementary Equation 1, where *a* is the weight of Fmoc-protected resin (mg), and *b* is absorbance at 301 nm.

$$x = (10000 \times b) / (7800 \times a) \tag{1}$$

Procedures for split-and-mix synthesis. Bead-linked 2401 peptides were prepared on a peptide synthesizer. Standard operation was shown as follows:

Step 1: The solid supported N_α-Fmoc peptide (*a* μmol, $a < 50$) was split into seven 5 mL LibraTubes with CH_2Cl_2 and dried under vacuum.

Step 2: The solid supported N_α-Fmoc peptide was deprotected with piperidine/NMP (1/4, room temperature, 10 min).

Step 3: The resin in 5 mL LibraTube was washed with NMP (2 mL, 30 sec × 5).

Step 4: An amino acid (4.0 eq) in a vial was activated by a solution of *O*-(7-azabenzotriazole-1-yl)-*N,N,N',N'*-tetramethyluronium hexafluorophosphate (HATU, 4.0 eq, 0.45 M)/1-hydroxy-7-azabenzotriazole (HOAt, 4.0 eq, 0.45 M) in NMP. To the solution of activated amino acid was added a solution of *i*-Pr_2NEt (8.0 eq, 2.0 M) in NMP. The resultant mixture was transferred to the reaction vessel. The vial was washed with NMP (0.0072 × *a* mL). The solution was transferred to the reaction vessel.

Step 5: The activated amino acid was coupled with the peptide on the resin (40 °C, 100 W; 20 min) and the reaction vessel containing the resin was washed with NMP (2 mL, 30 sec × 5).

Step 6: Seven batches of resin were mixed in one LibraTube with CH_2Cl_2.

Steps 1−6 were conducted for the condensation of the residues-3, -6, -9, and -11.

Steps 2−5 were conducted for the condensation of the residues-2, -4, -5, -7, and -10.

The coupling of **9** was conducted at room temperature for 40 min.

On-bead esterification. To the bead-linked heptapeptide (*a* μmol, *a* < 50) in 5 mL LibraTube was added a solution of *N*,*N*'-diisopropylcarbodiimide (8.0 eq) and **10** (8.0 eq) in NMP/CH_2Cl_2 (1/9, 500 μL). To the mixture was added *N*,*N*-dimethyl-4-aminopyridine (1.0 eq). After being stirred at room temperature for 3 h, the reaction mixture was washed with CH_2Cl_2 (1.00 mL × 5) and NMP (1.00 mL × 5) to give the bead-linked depsipeptide, which was used in the next reaction without further purification.

On-bead macrolactamization and deprotection. To the bead-linked depsipeptide **36** or **S5** in LibraTube was added a solution of $Pd(PPh_3)_4$ (0.25 eq) and morpholine (24 eq) in CH_2Cl_2 (500 μL). After being stirred at room temperature for 30 min, the reaction mixture was washed with CH_2Cl_2 (1.00 mL × 5) to give the bead-linked peptide.

To the above bead-linked peptide in LibraTube was added a solution of PyBOP (5.0 eq) and 2,4,6-collidine (10 eq) in NMP/CH_2Cl_2 (1/9, 500 μL). After being stirred at room temperature for 12–14 h, the reaction mixture was washed with CH_2Cl_2 (1.00 mL × 3), NMP (1.00 mL × 3), and CH_2Cl_2 (1.00 mL × 3), and dried under vacuum for 1 h to give bead-linked macrolactam.

To the above bead-linked macrolactam was added TFA/H_2O (19/1, 2.00 mL). After being stirred at room temperature for 1 h, the reaction mixture was filtered, washed with TFA/H_2O (19/1, 1.00 mL × 3), MeOH (1.00 mL × 3), and Et_2O (1.00 mL × 3), and dried under vacuum to give the bead-linked lysocin E analogue.

Bead-linked peptide 30. Compound **29** (37.4 mg, 8.30 μmol, loading rate 0.222 mmol/g) was subjected to the above microwave-assisted standard SPPS protocol, on-bead esterification, on-bead macrolactamization, and deprotection to give **30** (40.0 mg).

Bead-linked peptide BLP1. Compound **29** (39.2 mg, 8.70 μmol, loading rate 0.222 mmol/g) was subjected to the above microwave-assisted standard SPPS protocol, on-bead esterification, on-bead macrolactamization, and deprotection to give **BLP1** (42.9 mg, Supplementary Figure 3).

Bead-linked peptide BLP2. Compound **29** (41.7 mg, 9.26 μmol, loading rate 0.222 mmol/g) was subjected to the above microwave-assisted standard SPPS protocol, on-bead esterification, on-bead macrolactamization, and deprotection to give **BLP2** (47.6 mg, Supplementary Figure 3).

Determination of overall yield of lysocin E. Beads **29** (loading rate 0.174 mmol/g) was subjected to the above microwave-assisted standard SPPS protocol, on-bead esterification, on-bead macrolactamization, and deprotection to give bead-linked peptide **30**.

To the above bead-linked peptide **30** in a 96-well PCR plate (652270, Greiner bio-one, 1 bead/well, 96 beads) was added MeOH (30 µL/well). The microplate was irradiated with UV light (λ = 365 nm) using handy UV lamp (AS ONE, SLUV-6, 6 W) at room temperature for 2 h by shifting the position of the lamp by two lines every 30 min (Supplementary Figure 5). The resultant solution was concentrated under a stream of Ar at room temperature. To the crude peptides in the plate was added DMSO (30 µL/well). The plate was capped with 8-cap strips (373250, Greiner bio-one), and sonicated at room temperature for 10 min. The resultant mixture in the plate was centrifuged at 3500 × *g* at room temperature for 10 sec. The 8-cap strips were carefully removed from the plate. The supernatants were collected. The wells were washed with DMSO (10 µL/well × 2), and then the supernatants were collected. The combined supernatants were dried under vacuum to give the crude **1**. The crude **1** was dissolved in MeOH (50 µL), and filtered through 0.20 µm PTFE filter. The filtrate was analyzed by reversed-phase UHPLC (column: Accucore C18 2.1 × 150 mm, eluent A: MeCN + 0.05% TFA, eluent B: H_2O + 0.05% TFA, A/B = 40/60 flow rate: 0.40 mL/min, detection: photodiode array detector 200−648 nm (UV chromatogram: 280 nm), temperature: 40 ºC, t_R = 2.4 min, Supplementary Figure 1). The peak area of **1** (UV 280 nm) was calculated using ChromNAV (JASCO) to compare with the peak area of purified **1** as a reference compound. The overall yield of **1** from **29** was determined as 5.1% over 26 steps.

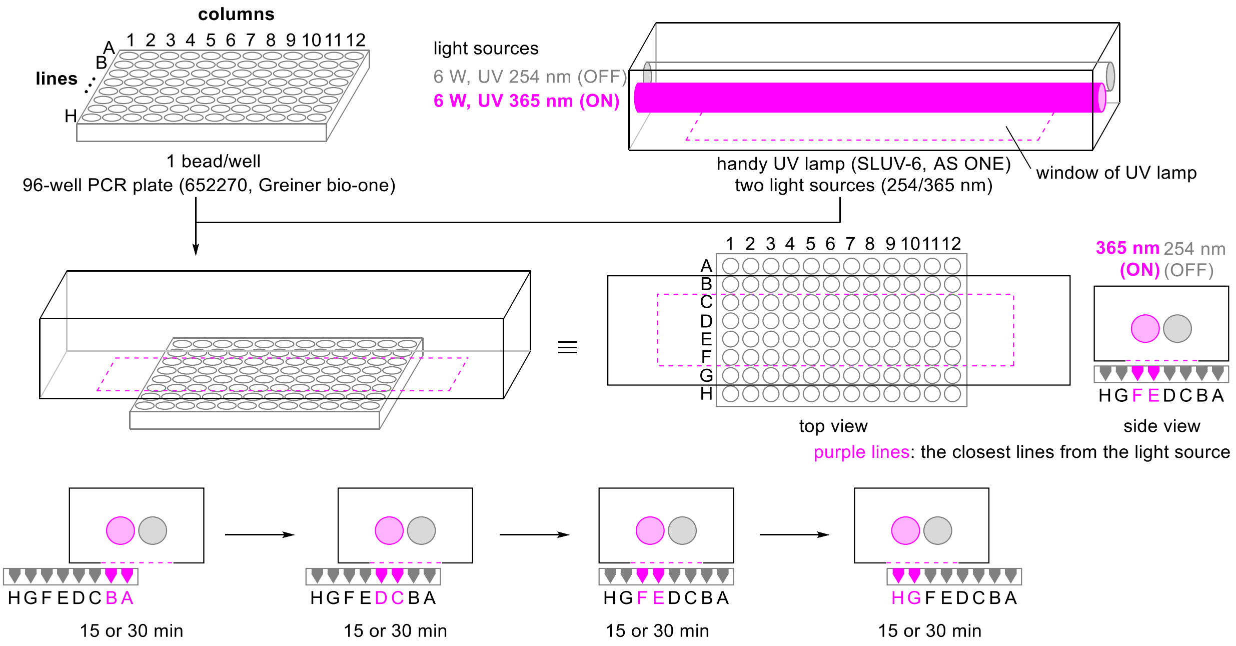

Supplementary Figure 5. Schematic diagram of procedure for cleavage of peptides. The closest lines from the light source (365 nm) are indicated in purple.

Construction of lysocin E-based OBOC library. Beads **29** (131 mg, 24.9 μmol, loading rate 0.190 mmol/g) was subjected to microwave-assisted split-and-mix synthesis protocol, on-bead esterification, on-bead macrolactamization, and deprotection to give bead-linked 2401 peptides (160 mg).

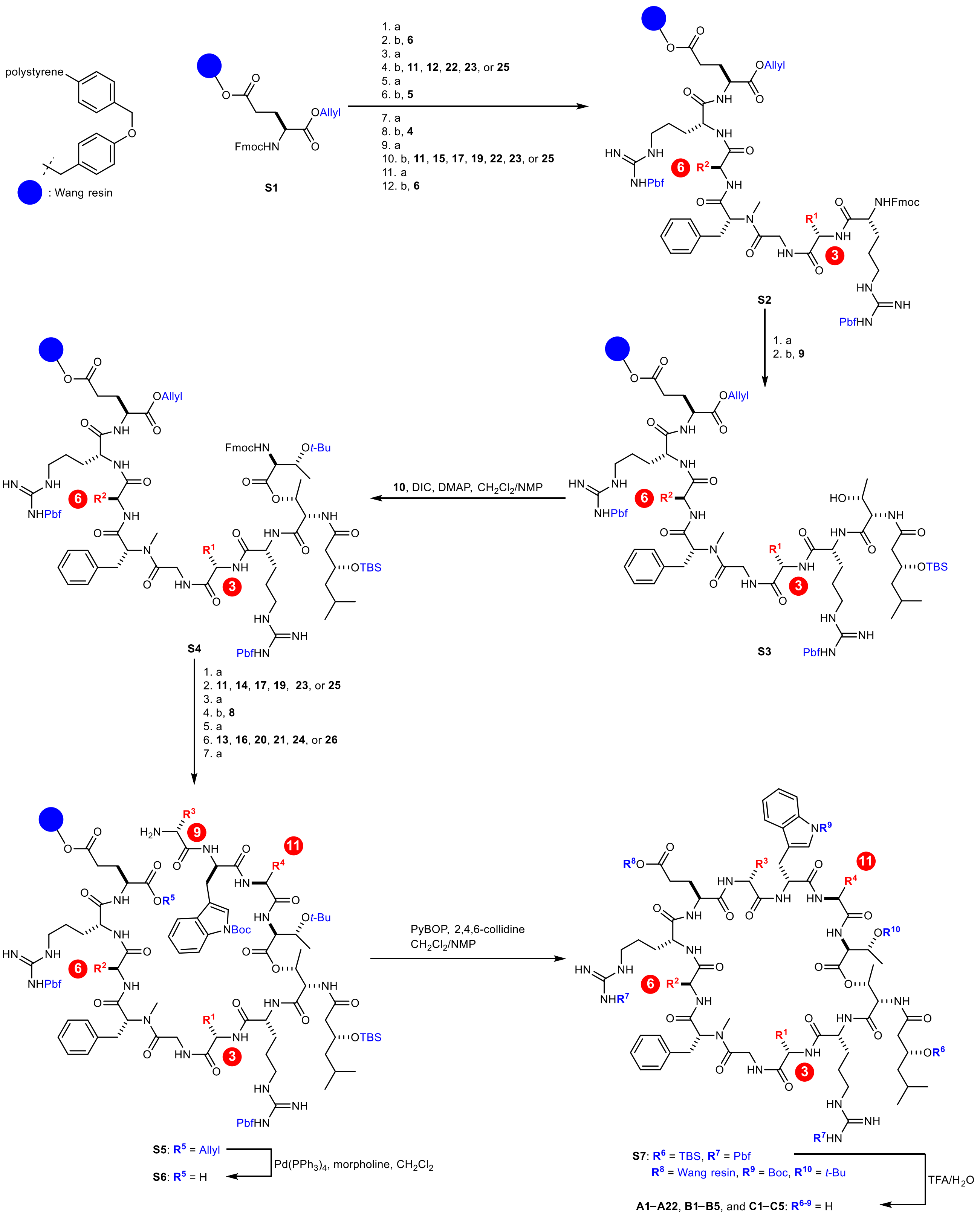

Supplementary Figure 6. Syntheses of peptides. Reagents and conditions: (a) 20% piperidine/NMP, (b) HATU, HOAt, *i*-Pr_2NEt, NMP, 40 °C (room temperature for **9**).

Procedures for solid-phase peptide synthesis (SPPS). Peptides **1**, **A1**−**A22**, **B1**−**B5**, and **C1**−**C5** were prepared on a peptide synthesizer. Standard operation was shown as follows:

Step 1: The solid supported N_α-Fmoc peptide was deprotected with piperidine/NMP (1/4, room temperature, 10 min).

Step 2: The resin in reaction vessel [20 mL LibraTube (Hipep Laboratories, for ≥ 50 μmol of **S1**) or 5 mL LibraTube (for < 50 μmol of **S1**)] was washed with NMP [5 mL (for ≥ 50 μmol of **S1**) or 2 mL (for < 50 μmol of **S1**), 40 sec × 6].

Step 3: An amino acid (4.0 eq) in a vial was activated by a solution of *O*-(7-azabenzotriazole-1-yl)-*N,N,N',N'*-tetramethyluronium hexafluorophosphate (HATU, 4.0 eq, 0.45 M)/1-hydroxy-7-azabenzotriazole (HOAt, 4.0 eq, 0.45 M) in NMP. To the solution of activated amino acid was added a solution of *i*-Pr_2NEt (8.0 eq, 2.0 M) in NMP. The resultant mixture was transferred to the reaction vessel. The vial was washed with NMP (0.0072 × *a* mL) for *a* μmol of **S1**. The solution was transferred to the reaction vessel.

Step 4: The activated amino acid was coupled with the peptide on the resin (40 °C, 200 W; 20 min) and the reaction vessel containing the resin was washed with NMP [5 mL (for ≥ 50 μmol of **S1**) or 2 mL (for < 50 μmol of **S1**), 40 sec × 6].

Steps 1–4 were repeated and amino acids were condensed on the solid support.

Syntheses of lysocin E analogues. Preloaded resin Fmoc-L-Glu(Wang resin)-OAllyl (**S1,** *a* μmol) was purchased from Novabiochem. Resin **S1** in the reaction vessel [20 mL LibraTube (for *a* ≥ 50) or 5 mL LibraTube (for *a* < 50)] was washed with CH_2Cl_2 [5 mL (for *a* ≥ 50) or 2 mL (for *a* < 50), 40 sec × 3] and NMP [5 mL (for *a* ≥ 50) or 2 mL (for *a* < 50), 40 sec × 6]. The resin was subjected to 6 cycles (**6**, **X2**, **5**, **4**, **X1**, and **6**) of the microwave-assisted SPPS protocol to give peptide **S2**, where **X2** is one of **11**, **12**, **17**, **22**, **23**, and **25**, and **X1** is one of **11**, **15**, **17**, **19**, **22**, **23**, and **25**.

The resin-bound peptide **S2** was subjected to the microwave-assisted SPPS protocol (the coupling of **9** was conducted at room temperature for 40 min). The reaction mixture was washed with NMP/CH_2Cl_2 [1/9, 5 mL (for *a* ≥ 50) or 2 mL (for *a* < 50), 40 sec × 6] to give peptide **S3**.

To **10** (8.0 eq) in a vial was added a solution of *N,N'*-diisopropylcarbodiimide (8.0 eq, 0.57 M) in NMP/CH_2Cl_2 (1/9). The resultant mixture was transferred to the resin-bound peptide **S3** in the reaction vessel [20 mL LibraTube (for *a* ≥ 50) or 5 mL LibraTube (for *a* < 50)]. The vial was washed with NMP/CH_2Cl_2 (1/9, 0.0047 × *a* mL). The resultant solution was transferred to the reaction vessel. The reaction mixture was stirred at room temperature for 5 min, and then a solution of 4-(*N,N*-dimethylamino)pyridine (1.0 eq, 0.82 M) in NMP/CH_2Cl_2 (1/9) was added. After being stirred at room temperature for 2 h, the resultant mixture was washed with NMP/CH_2Cl_2 [1/9, 5 mL (for *a* ≥ 50) or 2 mL (for *a* < 50), 40 sec × 6]. This coupling reaction was repeated (× 2). The reaction mixture was washed with CH_2Cl_2 [5 mL (for *a* ≥ 50) or 2 mL (for *a* < 50), 40 sec × 6] and NMP [5 mL (for *a* ≥ 50) or 2 mL (for *a* < 50), 40 sec × 6] to give peptide **S4**.

The resin-bound peptide **S4** was subjected to 3 cycles (**X4**, **8**, and **X3**) of the microwave-assisted SPPS

protocol (**X3** is one of **11**, **14**, **17**, **19**, **23**, and **25**, and **X4** is one of **13**, **16**, **18**, **20**, **21**, **24**, and **26**). The N_α-Fmoc group of the resultant peptide was removed by steps 1 and 2 of the above microwave-assisted SPPS protocol. The reaction mixture was washed with CH_2Cl_2 [5 mL (for $a \geq 50$) or 2 mL (for $a < 50$), 40 sec × 6] to give peptide **S5**.

To the resin-bound peptide **S5** in the reaction vessel [20 mL LibraTube (for $a \geq 50$) or 5 mL LibraTube (for $a < 50$)] was added a solution of $Pd(PPh_3)_4$ (0.25 eq, 0.013 M) and morpholine (24 eq, 2.6 M) in CH_2Cl_2. After being stirred at room temperature for 30 min, the reaction mixture was washed with CH_2Cl_2 [5 mL (for $a \geq 50$) or 2 mL (for $a < 50$), 40 sec × 6], NMP [5 mL (for $a \geq 50$) or 2 mL (for $a < 50$), 40 sec × 6], and NMP/CH_2Cl_2 [1/9, 5 mL (for $a \geq 50$) or 2 mL (for $a < 50$), 40 sec × 6] to give peptide **S6**.

To the resin-bound peptide **S6** in the reaction vessel [20 mL LibraTube (for $a \geq 50$) or 5 mL LibraTube (for $a < 50$)] were added a solution of PyBOP (5.0 eq, 0.33 M) in NMP/CH_2Cl_2 (1/9) and 2,4,6-collidine (10 eq). After being stirred at room temperature for 11 h, the reaction mixture was washed with CH_2Cl_2 [5 mL (for $a \geq 50$) or 2 mL (for $a < 50$), 40 sec × 6], NMP [5 mL (for $a \geq 50$) or 2 mL (for $a < 50$), 40 sec × 6], and CH_2Cl_2 [5 mL (for $a \geq 50$) or 2 mL (for $a < 50$), 40 sec × 6] and dried under vacuum to give peptide **S7**. For the resin-bound peptide **S7** in 20 mL LibraTube, the resin-bound peptide was split into three 5 mL LibraTubes.

To the resin-bound peptide **S7** in 5 mL LibraTube was added TFA/H_2O (19/1, 2.0 mL). After being stirred at room temperature for 1 h, the reaction mixture was filtered, and washed with TFA/H_2O (19/1, 1.0 mL, 40 sec × 6). The combined filtrates were stirred for 2 h. The resultant mixture was concentrated to give the crude peptide. To the crude peptide was added Et_2O (10 mL). The resultant mixture was sonicated at room temperature for 1 min, and the organic layer was removed. The same procedure was repeated (× 2). The residue was dried under vacuum to give the crude peptide. The crude peptide was dissolved in MeCN containing 0.05% TFA/H_2O containing 0.05% TFA [25/75, 8.0 mL (for each batch of $a \geq 50$) or 5.0 mL (for $a < 50$)]. The solution was charged on InertSep Slim C18-B [840 mg (for each batch of $a \geq 50$) or 360 mg (for $a < 50$)]. The column was washed with MeCN containing 0.05% TFA/H_2O containing 0.05% TFA [25/75, 8.0 mL (for each batch of $a \geq 50$) or 5.0 mL (for $a < 50$)] to remove impurities. The crude peptide was eluted with MeCN containing 0.05% TFA/H_2O containing 0.05% TFA [60/40, 16 mL (for each batch of $a \geq 50$) or 10 mL (for $a < 50$)], and lyophilized. The residue was purified by reversed-phase HPLC to give the **1** analogue.

Peptide A1. Fmoc-L-Glu(Wang resin)-OAllyl (**S1**, loading rate: 0.45 mmol/g) was subjected to the general procedures described above using **25** as **X1**, **12** as **X2**, **13** as **X3**, and **14** as **X4**. The crude **A1** was purified by 1st reversed-phase HPLC (column: Inertsil C8-3 20 × 250 mm, eluent A: MeOH + 0.05% TFA, eluent B: H_2O + 0.05% TFA, linear gradient A/B = 60/40 to 85/15 over 50 min, flow rate: 5.0 mL/min, detection: photodiode array detector 199–651 nm) and 2nd reversed-phase HPLC (column: Inertsil C8-3 20 × 250 mm, eluent A: MeCN + 0.05% TFA, eluent B: H_2O + 0.05% TFA, linear gradient A/B = 37.5/62.5 to 45/55 over 30 min, then 45/55 over 10 min, flow rate: 5.0 mL/min, detection: photodiode array detector 199–651 nm) to give **A1** (t_R = 29.6–31.4 min, 18.7 mg, 10.9 μmol, 10% over 25 steps): white solid; $[\alpha]_D^{19}$ = +22.2° (c = 0.800, MeOH); IR (film) ν 1135, 1183, 1202, 1436, 1538, 1627, 1668, 2362, 2957, 3272 cm^{-1}; ^1H NMR (800 MHz, DMSO-d_6), see Supplementary Table 5; ^{13}C NMR (200 MHz, DMSO-d_6), see Supplementary Table 6; HRMS

(ESI) calcd for $C_{75}H_{117}N_{20}O_{19}$ $[M+H]^+$ 1601.8798, found 1601.8795.

Peptide A2. Fmoc-L-Glu(Wang resin)-OAllyl (**S1**, loading rate: 0.45 mmol/g) was subjected to the general procedures described above using **25** as **X1**, **12** as **X2**, **16** as **X3**, and **14** as **X4**. The crude **A2** was purified by 1st reversed-phase HPLC (column: Inertsil C8-3 20 × 250 mm, eluent A: MeOH + 0.05% TFA, eluent B: H_2O + 0.05% TFA, linear gradient A/B = 60/40 to 85/15 over 50 min, flow rate: 5.0 mL/min, detection: photodiode array detector 199–651 nm) and 2nd reversed-phase HPLC (column: Inertsil C8-3 20 × 250 mm, eluent A: MeCN + 0.05% TFA, eluent B: H_2O + 0.05% TFA, linear gradient A/B = 37.5/62.5 to 45/55 over 30 min, then 45/55 over 10 min, flow rate: 5.0 mL/min, detection: photodiode array detector 199–651 nm) to give **A2** (t_R = 35.7–37.1 min, 12.4 mg, 7.37 μmol, 6.9% over 25 steps): white solid; $[\alpha]_D^{23}$ = +26.2° (c = 0.427, MeOH); IR (film) ν 1139, 1203, 1454, 1540, 1627, 1678, 2338, 2360, 2959, 3276, 3625, 3730 cm^{-1}; ^1H NMR (800 MHz, DMSO-d_6), see Supplementary Table 5; ^{13}C NMR (200 MHz, DMSO-d_6), see Supplementary Table 6; HRMS (ESI) calcd for $C_{75}H_{118}N_{19}O_{18}$ $[M+H]^+$ 1572.8897, found 1572.8927.

Peptide A3. Fmoc-L-Glu(Wang resin)-OAllyl (**S1**, loading rate: 0.45 mmol/g) was subjected to the general procedures described above using **19** as **X1**, **12** as **X2**, **16** as **X3**, and **14** as **X4**. The crude **A3** was purified by 1st reversed-phase HPLC (column: Inertsil C8-3 20 × 250 mm, eluent A: MeOH + 0.05% TFA, eluent B: H_2O + 0.05% TFA, linear gradient A/B = 60/40 to 85/15 over 50 min, flow rate: 5.0 mL/min, detection: photodiode array detector 199–651 nm) and 2nd reversed-phase HPLC (column: Inertsil C8-3 20 × 250 mm, eluent A: MeCN + 0.05% TFA, eluent B: H_2O + 0.05% TFA, A/B = 37.5/62.5 over 50 min, flow rate: 5.0 mL/min, detection: photodiode array detector 199–651 nm) to give **A3** (t_R = 27.6–29.9 min, 13.8 mg, 7.43 μmol, 6.9% over 25 steps): white solid; $[\alpha]_D^{24}$ = +25.0° (c = 0.447, MeOH); IR (film) ν 1141, 1200, 1465, 1515, 1629, 1677, 1740, 2305, 3274, 3742 cm^{-1}; ^1H NMR (800 MHz, DMSO-d_6), see Supplementary Table 5; ^{13}C NMR (200 MHz, DMSO-d_6), see Supplementary Table 6; HRMS (ESI) calcd for $C_{78}H_{125}N_{20}O_{18}$ $[M+H]^+$ 1629.9475, found 1629.9477.

Peptide A4. Fmoc-L-Glu(Wang resin)-OAllyl (**S1**, loading rate: 0.746 mmol/g) was subjected to the general procedures described above using **15** as **X1**, **12** as **X2**, **20** as **X3**, and **14** as **X4**. The crude **A4** was purified by 1st reversed-phase HPLC (column: Inertsil ODS-4 10 × 250 mm, eluent A: MeCN + 0.05% TFA, eluent B: H_2O + 0.05% TFA, linear gradient A/B = 35/65 to 50/50 over 25 min, then 50/50 over 5 min, flow rate: 3.0 mL/min, detection: UV 280 nm) and 2nd reversed-phase HPLC (column: Inertsil ODS-4 10 × 250 mm, eluent A: MeOH + 0.05% TFA, eluent B: H_2O + 0.05% TFA, linear gradient A/B = 70/30 to 90/10 over 40 min, flow rate: 2.0 mL/min, detection: UV 220 nm) to give **A4** (t_R = 14.1–16.0 min, 1.22 mg, 0.705 μmol, 4.1% over 25 steps): white solid; HRMS (ESI) calcd for $C_{77}H_{123}N_{20}O_{18}$ $[M+H]^+$ 1615.9319, found 1615.9277.

Peptide A5. Fmoc-L-Glu(Wang resin)-OAllyl (**S1**, loading rate: 0.45 mmol/g) was subjected to the general procedures described above using **11** as **X1**, **12** as **X2**, **16** as **X3**, and **14** as **X4**. The crude **A5** was purified by 1st reversed-phase HPLC (column: Inertsil C8-3 10 × 250 mm, eluent A: MeCN + 0.05% TFA, eluent B:

H_2O + 0.05% TFA, linear gradient A/B = 25/75 to 50/50 over 50 min, flow rate: 3.0 mL/min, detection: photodiode array detector 199–651 nm) and 2nd reversed-phase HPLC (column: Inertsil C8-3 10 × 250 mm, eluent A: MeOH + 0.05% TFA, eluent B: H_2O + 0.05% TFA, linear gradient A/B = 45/55 to 75/25 over 60 min, flow rate: 3.0 mL/min, detection: photodiode array detector 199–651 nm) to give **A5** (t_R = 55.5–56.8 min, 0.684 mg, 0.402 μmol, 1.8% over 25 steps): white solid; HRMS (ESI) calcd for $C_{75}H_{118}N_{19}O_{19}$ $[M+H]^+$ 1588.8846, found 1588.8895.

Peptide A6. Fmoc-L-Glu(Wang resin)-OAllyl (**S1**, loading rate: 0.45 mmol/g) was subjected to the general procedures described above using **15** as **X1**, **12** as **X2**, **13** as **X3**, and **14** as **X4**. The crude **A6** was purified by 1st reversed-phase HPLC (column: Inertsil C8-3 10 × 250 mm, eluent A: MeCN + 0.05% TFA, eluent B: H_2O + 0.05% TFA, linear gradient A/B = 25/75 to 50/50 over 50 min, flow rate: 3.0 mL/min, detection: photodiode array detector 199–651 nm) and 2nd reversed-phase HPLC (column: Inertsil C8-3 10 × 250 mm, eluent A: MeOH + 0.05% TFA, eluent B: H_2O + 0.05% TFA, linear gradient A/B = 45/55 to 75/25 over 60 min, flow rate: 3.0 mL/min, detection: photodiode array detector 199–651 nm) to give **A6** (t_R = 54.3–55.8 min, 3.71 mg, 2.13 μmol, 9.8% over 25 steps): white solid; HRMS (ESI) calcd for $C_{77}H_{121}N_{20}O_{19}$ $[M+H]^+$ 1629.9111, found 1629.9111.

Peptide A7. Fmoc-L-Glu(Wang resin)-OAllyl (**S1**, loading rate: 0.45 mmol/g) was subjected to the general procedures described above using **23** as **X1**, **12** as **X2**, **20** as **X3**, and **14** as **X4**. The crude **A7** was purified by 1st reversed-phase HPLC (column: Inertsil C8-3 10 × 250 mm, eluent A: MeCN + 0.05% TFA, eluent B: H_2O + 0.05% TFA, linear gradient A/B = 25/75 to 50/50 over 50 min, flow rate: 3.0 mL/min, detection: photodiode array detector 199–651 nm) and 2nd reversed-phase HPLC (column: Inertsil C8-3 10 × 250 mm, eluent A: MeOH + 0.05% TFA, eluent B: H_2O + 0.05% TFA, linear gradient A/B = 45/55 to 75/25 over 60 min, flow rate: 3.0 mL/min, detection: photodiode array detector 199–651 nm) to give **A7** (t_R = 51.0–52.8 min, 3.53 mg, 1.85 μmol, 8.8% over 25 steps): white solid; HRMS (ESI) calcd for $C_{81}H_{123}N_{20}O_{19}$ $[M+H]^+$ 1679.9268, found 1679.9276.

Peptide A8. Fmoc-L-Glu(Wang resin)-OAllyl (**S1**, loading rate: 0.45 mmol/g) was subjected to the general procedures described above using **23** as **X1**, **12** as **X2**, **13** as **X3**, and **14** as **X4**. The crude **A8** was purified by 1st reversed-phase HPLC (column: Inertsil C8-3 10 × 250 mm, eluent A: MeCN + 0.05% TFA, eluent B: H_2O + 0.05% TFA, linear gradient A/B = 25/75 to 49/51 over 48 min, flow rate: 3.0 mL/min, detection: photodiode array detector 199–651 nm) and 2nd reversed-phase HPLC (column: Inertsil C8-3 10 × 250 mm, eluent A: MeOH + 0.05% TFA, eluent B: H_2O + 0.05% TFA, linear gradient A/B = 45/55 to 75/25 over 60 min, flow rate: 3.0 mL/min, detection: photodiode array detector 199–651 nm) to give **A8** (t_R = 55.7–57.1 min, 2.27 mg, 1.26 μmol, 5.6% over 25 steps): white solid; HRMS (ESI) calcd for $C_{81}H_{121}N_{20}O_{20}$ $[M+H]^+$ 1693.9061, found 1693.9086.

Peptide A9. Fmoc-L-Glu(Wang resin)-OAllyl (**S1**, loading rate: 0.746 mmol/g) was subjected to the general procedures described above using **23** as **X1**, **12** as **X2**, **16** as **X3**, and **14** as **X4**. The crude **A9** was purified

by 1st reversed-phase HPLC (column: Inertsil ODS-4 4.6 × 250 mm, eluent A: MeCN + 0.05% TFA, eluent B: H_2O + 0.05% TFA, linear gradient A/B = 35/65 to 50/50 over 20 min, then 50/50 over 15 min, flow rate: 1.0 mL/min, detection: UV 280 nm) and 2nd reversed-phase HPLC (column: Inertsil ODS-4 10 × 250 mm, eluent A: MeOH + 0.05% TFA, eluent B: H_2O + 0.05% TFA, linear gradient A/B = 70/30 to 90/10 over 40 min, flow rate: 2.0 mL/min, detection: UV 220 nm) to give **A9** (t_R = 17.4–20.1 min, 1.25 mg, 0.703 μmol, 3.8% over 25 steps): white solid; HRMS (ESI) calcd for $C_{81}H_{122}N_{19}O_{19}$ $[M+H]^+$ 1664.9159, found 1664.9207.

Peptide A10. Fmoc-L-Glu(Wang resin)-OAllyl (**S1**, loading rate: 0.746 mmol/g) was subjected to the general procedures described above using **22** as **X1**, **12** as **X2**, **13** as **X3**, and **14** as **X4**. The crude **A10** was purified by 1st reversed-phase HPLC (column: Inertsil ODS-4 4.6 × 250 mm, eluent A: MeCN + 0.05% TFA, eluent B: H_2O + 0.05% TFA, linear gradient A/B = 35/65 to 50/50 over 20 min, then 50/50 over 15 min, flow rate: 1.0 mL/min, detection: UV 280 nm) and 2nd reversed-phase HPLC (column: Inertsil ODS-4 10 × 250 mm, eluent A: MeOH + 0.05% TFA, eluent B: H_2O + 0.05% TFA, linear gradient A/B = 70/30 to 90/10 over 40 min, flow rate: 2.0 mL/min, detection: UV 220 nm) to give **A10** (t_R = 15.6–18.3 min, 1.18 mg, 0.671 μmol, 3.7% over 25 steps): white solid; HRMS (ESI) calcd for $C_{76}H_{118}N_{21}O_{20}$ $[M+H]^+$ 1644.8857, found 1644.8871.

Peptide A11. Fmoc-L-Glu(Wang resin)-OAllyl (**S1**, loading rate: 0.45 mmol/g) was subjected to the general procedures described above using **15** as **X1**, **12** as **X2**, **24** as **X3**, and **14** as **X4**. The crude **A11** was purified by 1st reversed-phase HPLC (column: Inertsil C8-3 10 × 250 mm, eluent A: MeOH + 0.05% TFA, eluent B: H_2O + 0.05% TFA, linear gradient A/B = 55/45 to 80/20 over 50 min, flow rate: 3.0 mL/min, detection: photodiode array detector 199–651 nm) and 2nd reversed-phase HPLC (column: Inertsil C8-3 10 × 250 mm, eluent A: MeOH/MeCN (2/1) + 0.05% TFA, eluent B: H_2O + 0.05% TFA, linear gradient A/B = 40/60 to 75/25 over 50 min, flow rate: 3.0 mL/min, detection: photodiode array detector 199–651 nm) to give **A11** (t_R = 27.3-28.0 min, 3.35 mg, 1.88 μmol, 8.3% over 25 steps): white solid; HRMS (ESI) calcd for $C_{81}H_{122}N_{19}O_{19}$ $[M+H]^+$ 1664.9159, found 1664.9145.

Peptide A12. Fmoc-L-Glu(Wang resin)-OAllyl (**S1**, loading rate: 0.746 mmol/g) was subjected to the general procedures described above using **15** as **X1**, **12** as **X2**, **21** as **X3**, and **14** as **X4**. The crude **A12** was purified by 1st reversed-phase HPLC (column: Inertsil ODS-4 10 × 250 mm, eluent A: MeCN + 0.05% TFA, eluent B: H_2O + 0.05% TFA, linear gradient A/B = 35/65 to 50/50 over 25 min, then 50/50 over 30 min, flow rate: 3.0 mL/min, detection: UV 280 nm) and 2nd reversed-phase HPLC (column: Inertsil ODS-4 10 × 250 mm, eluent A: MeOH + 0.05% TFA, eluent B: H_2O + 0.05% TFA, linear gradient A/B = 70/30 to 85/15 over 30 min, flow rate: 2.0 mL/min, detection: UV 220 nm) to give **A12** (t_R = 17.4–18.9 min, 0.944 mg, 0.554 μmol, 3.4% over 25 steps): white solid; HRMS (ESI) calcd for $C_{75}H_{118}N_{19}O_{19}$ $[M+H]^+$ 1588.8846, found 1588.8873.

Peptide A13. Fmoc-L-Glu(Wang resin)-OAllyl (**S1**, loading rate: 0.45 mmol/g) was subjected to the general procedures described above using **22** as **X1**, **12** as **X2**, **16** as **X3**, and **14** as **X4**. The crude **A13** was purified

by 1st reversed-phase HPLC (column: Inertsil C8-3 10 × 250 mm, eluent A: MeOH + 0.05% TFA, eluent B: H_2O + 0.05% TFA, linear gradient A/B = 55/45 to 80/20 over 50 min, flow rate: 3.0 mL/min, detection: photodiode array detector 199–651 nm) and 2nd reversed-phase HPLC (column: Inertsil C8-3 10 × 250 mm, eluent A: MeCN + 0.05% TFA, eluent B: H_2O + 0.05% TFA, linear gradient A/B = 30/70 to 50/50 over 40 min, flow rate: 3.0 mL/min, detection: photodiode array detector 199–651 nm) to give **A13** (t_R = 32.9–33.9 min, 2.55 mg, 1.47 μmol, 6.1% over 25 steps): white solid; HRMS (ESI) calcd for $C_{76}H_{119}N_{20}O_{19}$ $[M+H]^+$ 1615.8955, found 1615.8967.

Peptide A14. Fmoc-L-Glu(Wang resin)-OAllyl (**S1**, loading rate: 0.746 mmol/g) was subjected to the general procedures described above using **22** as **X1**, **12** as **X2**, **20** as **X3**, and **14** as **X4**. The crude **A14** was purified by 1st reversed-phase HPLC column: Inertsil ODS-4 10 × 250 mm, eluent A: MeCN + 0.05% TFA, eluent B: H_2O + 0.05% TFA, linear gradient A/B = 35/65 to 50/50 over 25 min, then 50/50 over 30 min, flow rate: 3.0 mL/min, detection: UV 280 nm) and 2nd reversed-phase HPLC (column: Inertsil ODS-4 10 × 250 mm, eluent A: MeOH + 0.05% TFA, eluent B: H_2O + 0.05% TFA, linear gradient A/B = 70/30 to 79/21 over 18 min, flow rate: 2.0 mL/min, detection: UV 220 nm) to give **A14** (t_R = 13.2–14.9 min, 0.939 mg, 0.538 μmol, 3.6% over 25 steps): white solid; HRMS (ESI) calcd for $C_{76}H_{120}N_{21}O_{19}$ $[M+H]^+$ 1630.9064, found 1630.9112.

Peptide A15. Fmoc-L-Glu(Wang resin)-OAllyl (**S1**, loading rate: 0.746 mmol/g) was subjected to the general procedures described above using **25** as **X1**, **12** as **X2**, **24** as **X3**, and **23** as **X4**. The crude **A15** was purified by 1st reversed-phase HPLC (column: Inertsil ODS-4 10 × 250 mm, eluent A: MeCN + 0.05% TFA, eluent B: H_2O + 0.05% TFA, linear gradient A/B = 35/65 to 50/50 over 20 min, then 50/50 over 10 min, flow rate: 3.0 mL/min, detection: UV 280 nm) and 2nd reversed-phase HPLC (column: Inertsil ODS-4 10 × 250 mm, eluent A: MeOH + 0.05% TFA, eluent B: H_2O + 0.05% TFA, linear gradient A/B = 70/30 to 85/15 over 30 min, flow rate: 2.0 mL/min, detection: UV 220 nm) to give **A15** (t_R = 11.0–12.8 min, 1.54 mg, 0.855 μmol, 5.8% over 25 steps): white solid; HRMS (ESI) calcd for $C_{82}H_{116}N_{19}O_{20}$ $[M+H]^+$ 1686.8639, found 1686.8662.

Peptide A16. Fmoc-L-Glu(Wang resin)-OAllyl (**S1**, loading rate: 0.746 mmol/g) was subjected to the general procedures described above using **17** as **X1**, **12** as **X2**, **20** as **X3**, and **14** as **X4**. The crude **A16** was purified by 1st reversed-phase HPLC (column: Inertsil ODS-4 10 × 250 mm, eluent A: MeCN + 0.05% TFA, eluent B: H_2O + 0.05% TFA, linear gradient A/B = 35/65 to 50/50 over 25 min, then 50/50 over 5 min, flow rate: 3.0 mL/min, detection: UV 280 nm) and 2nd reversed-phase HPLC (column: Inertsil ODS-4 10 × 250 mm, eluent A: MeOH + 0.05% TFA, eluent B: H_2O + 0.05% TFA, linear gradient A/B = 70/30 to 85/15 over 30 min, flow rate: 2.0 mL/min, detection: UV 220 nm) to give **A16** (t_R = 13.2–15.5 min, 1.58 mg, 0.905 μmol, 5.9% over 25 steps): white solid; HRMS (ESI) calcd for $C_{76}H_{119}N_{20}O_{20}$ $[M+H]^+$ 1631.8904, found 1631.8889.

Peptide A17. Fmoc-L-Glu(Wang resin)-OAllyl (**S1**, loading rate: 0.45 mmol/g) was subjected to the general procedures described above using **17** as **X1**, **12** as **X2**, **24** as **X3**, and **14** as **X4**. The crude **A17** was purified

by 1st reversed-phase HPLC (column: Inertsil C8-3 10 × 250 mm, eluent A: MeOH + 0.05% TFA, eluent B: H_2O + 0.05% TFA, linear gradient A/B = 55/45 to 80/20 over 50 min, flow rate: 3.0 mL/min, detection: photodiode array detector 199–651 nm) and 2nd reversed-phase HPLC (column: Inertsil C8-3 10 × 250 mm, eluent A: MeCN + 0.05% TFA, eluent B: H_2O + 0.05% TFA, linear gradient A/B = 30/70 to 55/45 over 50 min, flow rate: 3.0 mL/min, detection: photodiode array detector 199–651 nm) to give **A17** (t_R = 34.1–35.2 min, 3.13 mg, 1.86 μmol, 9.3% over 25 steps): white solid; HRMS (ESI) calcd for $C_{80}H_{118}N_{19}O_{21}$ $[M+H]^+$ 1680.8744, found 1680.8766.

Peptide A18. Fmoc-L-Glu(Wang resin)-OAllyl (**S1**, loading rate: 0.746 mmol/g) was subjected to the general procedures described above using **17** as **X1**, **12** as **X2**, **16** as **X3**, and **14** as **X4**. The crude **A18** was purified by 1st reversed-phase HPLC (column: Inertsil ODS-4 4.6 × 250 mm, eluent A: MeCN + 0.05% TFA, eluent B: H_2O + 0.05% TFA, linear gradient A/B = 35/65 to 50/50 over 20 min, then 50/50 over 15 min, flow rate: 1.0 mL/min, detection: UV 280 nm) and 2nd reversed-phase HPLC (column: Inertsil ODS-4 10 × 250 mm, eluent A: MeOH + 0.05% TFA, eluent B: H_2O + 0.05% TFA, linear gradient A/B = 70/30 to 87.5/12.5 over 35 min, flow rate: 2.0 mL/min, detection: UV 220 nm) to give **A18** (t_R = 23.2–25.3 min, 0.821 mg, 0.474 μmol, 2.6% over 25 steps): white solid; HRMS (ESI) calcd for $C_{76}H_{118}N_{19}O_{20}$ $[M+H]^+$ 1616.8795, found 1616.8811.

Peptide A19. Fmoc-L-Glu(Wang resin)-OAllyl (**S1**, loading rate: 0.45 mmol/g) was subjected to the general procedures described above using **23** as **X1**, **12** as **X2**, **11** as **X3**, and **14** as **X4**. The crude **A19** was purified by 1st reversed-phase HPLC (column: Inertsil C8-3 10 × 250 mm, eluent A: MeCN + 0.05% TFA, eluent B: H_2O + 0.05% TFA, linear gradient A/B = 32.5/67.5 to 57.5/42.5 over 50 min, flow rate: 3.0 mL/min, detection: photodiode array detector 199–651 nm) and 2nd reversed-phase HPLC (column: Inertsil C8-3 10 × 250 mm, eluent A: MeOH + 0.05% TFA, eluent B: H_2O + 0.05% TFA, A/B = 67.5/32.5 over 25 min, flow rate: 3.0 mL/min, detection: photodiode array detector 199–651 nm) to give **A19** (t_R = 12.5–14.5 min, 4.31 mg, 2.44 μmol, 12% over 25 steps): white solid; HRMS (ESI) calcd for $C_{79}H_{118}N_{19}O_{20}$ $[M+H]^+$ 1652.8795, found 1652.8807.

Peptide A20. Fmoc-L-Glu(Wang resin)-OAllyl (**S1**, loading rate: 0.45 mmol/g) was subjected to the general procedures described above using **17** as **X1**, **12** as **X2**, **21** as **X3**, and **14** as **X4**. The crude **A20** was purified by 1st reversed-phase HPLC (column: Inertsil C8-3 10 × 250 mm, eluent A: MeCN + 0.05% TFA, eluent B: H_2O + 0.05% TFA, linear gradient A/B = 30/70 to 55/45 over 50 min, flow rate: 3.0 mL/min, detection: photodiode array detector 199–651 nm) and 2nd reversed-phase HPLC (column: Inertsil C8-3 10 × 250 mm, eluent A: MeOH + 0.05% TFA, eluent B: H_2O + 0.05% TFA, linear gradient A/B = 60/40 to 80/20 over 35 min, flow rate: 3.0 mL/min, detection: photodiode array detector 199–651 nm) to give **A20** (t_R = 20.3–21.6 min, 2.04 mg, 1.27 μmol, 5.1% over 25 steps): white solid; HRMS (ESI) calcd for $C_{74}H_{114}N_{19}O_{21}$ $[M+H]^+$ 1604.8431, found 1604.8409.

Peptide A21. Fmoc-L-Glu(Wang resin)-OAllyl (**S1**, loading rate: 0.746 mmol/g) was subjected to the general procedures described above using **17** as **X1**, **12** as **X2**, **16** as **X3**, and **23** as **X4**. The crude **A21** was purified by 1st reversed-phase HPLC (column: Inertsil ODS-4 10 × 250 mm, eluent A: MeCN + 0.05% TFA, eluent B: H_2O + 0.05% TFA, A/B = 35/65 over 5 min, then linear gradient A/B = 35/65 to 60/40 over 25 min, flow rate: 3.0 mL/min, detection: UV 280 nm), 2nd reversed-phase HPLC (column: Inertsil ODS-4 10 × 250 mm, eluent A: MeOH + 0.05% TFA, eluent B: H_2O + 0.05% TFA, linear gradient A/B = 65/35 to 70/30 over 25 min, then 70/30 over 5 min, flow rate: 3.0 mL/min, detection: photodiode array detector 199–651 nm), and 3rd reversed-phase HPLC (column: Inertsil ODS-4 10 × 250 mm, eluent A: MeOH + 0.05% TFA, eluent B: H_2O + 0.05% TFA, linear gradient A/B = 70/30 to 87.5/12.5 over 35 min, flow rate: 2.0 mL/min, detection: UV 220 nm) to give **A21** (t_R = 17.8–19.4 min, 0.969 mg, 0.544 μmol, 3.2% over 25 steps): white solid; HRMS (ESI) calcd for $C_{79}H_{116}N_{19}O_{21}$ $[M+H]^+$ 1666.8588, found 1666.8624.

Peptide A22. Fmoc-L-Glu(Wang resin)-OAllyl (**S1**, loading rate: 0.45 mmol/g) was subjected to the general procedures described above using **25** as **X1**, **22** as **X2**, **16** as **X3**, and **19** as **X4**. The crude **A22** was purified by 1st reversed-phase HPLC (column: Inertsil C8-3 10 × 250 mm, eluent A: MeOH + 0.05% TFA, eluent B: H_2O + 0.05% TFA, linear gradient A/B = 24/76 to 73/27 over 56 min, flow rate: 3.0 mL/min, detection: photodiode array detector 199–651 nm) and 2nd reversed-phase HPLC (column: Inertsil C8-3 10 × 250 mm, eluent A: MeCN + 0.05% TFA, eluent B: H_2O + 0.05% TFA, linear gradient A/B = 20/80 to 35/65 over 40 min, flow rate: 3.0 mL/min, detection: photodiode array detector 199–651 nm) to give **A22** (t_R = 24.2–26.0 min, 3.17 mg, 1.74 μmol, 8.4% over 25 steps): white solid; HRMS (ESI) calcd for $C_{73}H_{115}N_{21}O_{19}$ $[M+2H]^{2+}$ 794.9334, found 794.9346.

Peptide B1. Fmoc-L-Glu(Wang resin)-OAllyl (**S1**, loading rate: 0.45 mmol/g) was subjected to the general procedures described above using **15** as **X1**, **25** as **X2**, **21** as **X3**, and **14** as **X4**. The crude **B1** was purified by 1st reversed-phase HPLC (column: Inertsil C8-3 10 × 250 mm, eluent A: MeCN + 0.05% TFA, eluent B: H_2O + 0.05% TFA, linear gradient A/B = 30/70 to 55/45 over 50 min, flow rate: 3.0 mL/min, detection: photodiode array detector 199–651 nm) and 2nd reversed-phase HPLC (column: Inertsil C8-3 10 × 250 mm, eluent A: MeOH + 0.05% TFA, eluent B: H_2O + 0.05% TFA, linear gradient A/B = 45/55 to 70/30 over 50 min, flow rate: 3.0 mL/min, detection: photodiode array detector 199–651 nm) to give **B1** (t_R = 40.0–42.6 min, 2.24 mg, 1.35 μmol, 6.1% over 25 steps): white solid; HRMS (ESI) calcd for $C_{72}H_{113}N_{19}O_{19}$ $[M+2H]^{2+}$ 773.9225, found 773.9234.

Peptide B2. Fmoc-L-Glu(Wang resin)-OAllyl (**S1**, loading rate: 0.45 mmol/g) was subjected to the general procedures described above using **17** as **X1**, **12** as **X2**, **16** as **X3**, and **25** as **X4**. The crude **B2** was purified by 1st reversed-phase HPLC (column: Inertsil C8-3 10 × 250 mm, eluent A: MeOH + 0.05% TFA, eluent B: H_2O + 0.05% TFA, linear gradient A/B = 40/60 to 70/30 over 60 min, flow rate: 3.0 mL/min, detection: photodiode array detector 199–651 nm) and 2nd reversed-phase HPLC (column: Inertsil C8-3 10 × 250 mm, eluent A: MeCN + 0.05% TFA, eluent B: H_2O + 0.05% TFA, linear gradient A/B = 25/75 to 50/50 over 50 min,

flow rate: 3.0 mL/min, detection: photodiode array detector 199–651 nm) to give **B2** (t_R = 25.4–26.5 min, 3.35 mg, 2.13 μmol, 9.9% over 25 steps): white solid; HRMS (ESI) calcd for $C_{73}H_{112}N_{19}O_{20}$ $[M+H]^+$ 1574.8326, found 1574.8357.

Peptide B3. Fmoc-L-Glu(Wang resin)-OAllyl (**S1**, loading rate: 0.45 mmol/g) was subjected to the general procedures described above using **17** as **X1**, **12** as **X2**, **20** as **X3**, and **23** as **X4**. The crude **B3** was purified by 1st reversed-phase HPLC (column: Inertsil C8-3 10 × 250 mm, eluent A: MeOH + 0.05% TFA, eluent B: H_2O + 0.05% TFA, linear gradient A/B = 40/60 to 70/30 over 60 min, flow rate: 3.0 mL/min, detection: photodiode array detector 199–651 nm) and 2nd reversed-phase HPLC (column: Inertsil C8-3 10 × 250 mm, eluent A: MeCN + 0.05% TFA, eluent B: H_2O + 0.05% TFA, linear gradient A/B = 25/75 to 50/50 over 50 min, flow rate: 3.0 mL/min, detection: photodiode array detector 199–651 nm) to give **B5** (t_R = 30.8–32.2 min, 4.34 mg, 2.42 μmol, 11% over 25 steps): white solid; HRMS (ESI) calcd for $C_{79}H_{117}N_{20}O_{21}$ $[M+H]^+$ 1681.8697, found 1681.8727.

Peptide B4. Fmoc-L-Glu(Wang resin)-OAllyl (**S1**, loading rate: 0.45 mmol/g) was subjected to the general procedures described above using **23** as **X1**, **11** as **X2**, **26** as **X3**, and **19** as **X4**. The crude **B4** was purified by 1st reversed-phase HPLC (column: Inertsil C8-3 10 × 250 mm, eluent A: MeCN + 0.05% TFA, eluent B: H_2O + 0.05% TFA, linear gradient A/B = 25/75 to 35/65 over 40 min, flow rate: 3.0 mL/min, detection: photodiode array detector 199–651 nm) and 2nd reversed-phase HPLC (column: Inertsil C8-3 10 × 250 mm, eluent A: MeOH + 0.05% TFA, eluent B: H_2O + 0.05% TFA, linear gradient A/B = 45/55 to 62.5/57.5 over 50 min, flow rate: 3.0 mL/min, detection: photodiode array detector 199–651 nm) to give **B4** (t_R = 18.4–19.8 min, 0.743 mg, 0.401 μmol, 2.0% over 25 steps): white solid; HRMS (ESI) calcd for $C_{76}H_{114}N_{20}O_{20}$ $[M+2H]^{2+}$ 813.4254, found 813.4276.

Peptide B5. Fmoc-L-Glu(Wang resin)-OAllyl (**S1**, loading rate: 0.45 mmol/g) was subjected to the general procedures described above using **17** as **X1**, **12** as **X2**, **24** as **X3**, and **17** as **X4**. The crude **B5** was purified by 1st reversed-phase HPLC (column: Inertsil C8-3 10 × 250 mm, eluent A: MeCN + 0.05% TFA, eluent B: H_2O + 0.05% TFA, linear gradient A/B = 25/75 to 44/56 over 38 min, flow rate: 3.0 mL/min, detection: photodiode array detector 199–651 nm) and 2nd reversed-phase HPLC (column: Inertsil C8-3 10 × 250 mm, eluent A: MeOH + 0.05% TFA, eluent B: H_2O + 0.05% TFA, linear gradient A/B = 50/50 to 70/30 over 40 min, flow rate: 3.0 mL/min, detection: photodiode array detector 199–651 nm) to give **B5** (t_R = 27.3–28.0 min, 4.12 mg, 2.45 μmol, 12% over 25 steps): white solid; HRMS (ESI) calcd for $C_{78}H_{112}N_{19}O_{23}$ $[M+H]^+$ 1682.8173, found 1682.8175.

Peptide C1. Fmoc-L-Glu(Wang resin)-OAllyl (**S1**, loading rate: 0.45 mmol/g) was subjected to the general procedures described above using **15** as **X1**, **12** as **X2**, **24** as **X3**, and **11** as **X4**. The crude **C1** was purified by 1st reversed-phase HPLC (column: Inertsil C8-3 10 × 250 mm, eluent A: MeCN + 0.05% TFA, eluent B: H_2O + 0.05% TFA, linear gradient A/B = 25/75 to 50/50 over 50 min, flow rate: 3.0 mL/min, detection:

photodiode array detector 199–651 nm) and 2nd reversed-phase HPLC (column: Inertsil C8-3 10 × 250 mm, eluent A: MeOH + 0.05% TFA, eluent B: H_2O + 0.05% TFA, linear gradient A/B = 45/55 to 70/30 over 50 min, flow rate: 3.0 mL/min, detection: photodiode array detector 199–651 nm) to give **C1** (t_R = 38.2–39.6 min, 3.44 mg, 1.96 μmol, 8.8% over 25 steps): white solid; HRMS (ESI) calcd for $C_{78}H_{116}N_{19}O_{20}$ $[M+H]^+$ 1638.8639, found 1638.8627.

Peptide C2. Fmoc-L-Glu(Wang resin)-OAllyl (**S1**, loading rate: 0.45 mmol/g) was subjected to the general procedures described above using **25** as **X1**, **25** as **X2**, **24** as **X3**, and **23** as **X4**. The crude **C2** was purified by 1st reversed-phase HPLC (column: Inertsil C8-3 10 × 250 mm, eluent A: MeCN + 0.05% TFA, eluent B: H_2O + 0.05% TFA, linear gradient A/B = 30/70 to 55/45 over 50 min, flow rate: 3.0 mL/min, detection: photodiode array detector 199–651 nm) and 2nd reversed-phase HPLC (column: Inertsil C8-3 10 × 250 mm, eluent A: MeOH + 0.05% TFA, eluent B: H_2O + 0.05% TFA, linear gradient A/B = 60/40 to 80/20 over 35 min, flow rate: 3.0 mL/min, detection: photodiode array detector 199–651 nm) to give **C2** (t_R = 7.69–9.19 min, 1.48 mg, 0.843 μmol, 4.0% over 25 steps): white solid; HRMS (ESI) calcd for $C_{79}H_{110}N_{19}O_{20}$ $[M+H]^+$ 1644.8169, found 1644.8139.

Peptide C3. Fmoc-L-Glu(Wang resin)-OAllyl (**S1**, loading rate: 0.45 mmol/g) was subjected to the general procedures described above using **11** as **X1**, **25** as **X2**, **24** as **X3**, and **25** as **X4**. The crude **C3** was purified by 1st reversed-phase HPLC (column: Inertsil C8-3 10 × 250 mm, eluent A: MeCN + 0.05% TFA, eluent B: H_2O + 0.05% TFA, linear gradient A/B = 30/70 to 55/45 over 50 min, flow rate: 3.0 mL/min, detection: photodiode array detector 199–651 nm) and 2nd reversed-phase HPLC (column: Inertsil C8-3 10 × 250 mm, eluent A: MeOH + 0.05% TFA, eluent B: H_2O + 0.05% TFA, linear gradient A/B = 60/40 to 73.3/26.7 over 30 min, flow rate: 3.0 mL/min, detection: photodiode array detector 199–651 nm) to give **C3** (t_R = 7.51–8.82 min, 0.870 mg, 0.517 μmol, 2.5% over 25 steps): white solid; HRMS (ESI) calcd for $C_{73}H_{107}N_{19}O_{20}$ $[M+2H]^{2+}$ 784.8964, found 784.8957.

Peptide C4. Fmoc-L-Glu(Wang resin)-OAllyl (**S1**, loading rate: 0.45 mmol/g) was subjected to the general procedures described above using **17** as **X1**, **23** as **X2**, **13** as **X3**, and **14** as **X4**. The crude **C4** was purified by 1st reversed-phase HPLC (column: Inertsil C8-3 10 × 250 mm, eluent A: MeCN + 0.05% TFA, eluent B: H_2O + 0.05% TFA, linear gradient A/B = 25/75 to 47.5/52.5 over 45 min, flow rate: 3.0 mL/min, detection: photodiode array detector 199–651 nm) and 2nd reversed-phase HPLC (column: Inertsil C8-3 10 × 250 mm, eluent A: MeOH + 0.05% TFA, eluent B: H_2O + 0.05% TFA, linear gradient A/B = 45/55 to 70/30 over 50 min, flow rate: 3.0 mL/min, detection: photodiode array detector 199–651 nm) to give **C4** (t_R = 40.4–41.9 min, 1.62 mg, 0.955 μmol, 4.1% over 25 steps): white solid; HRMS (ESI) calcd for $C_{79}H_{115}N_{20}O_{22}$ $[M+H]^+$ 1695.8489, found 1695.8484.

Peptide C5. Fmoc-L-Glu(Wang resin)-OAllyl (**S1**, loading rate: 0.45 mmol/g) was subjected to the general procedures described above using **17** as **X1**, **23** as **X2**, **24** as **X3**, and **25** as **X4**. The crude **C5** was purified

by 1st reversed-phase HPLC (column: Inertsil C8-3 10 × 250 mm, eluent A: MeCN + 0.05% TFA, eluent B: H_2O + 0.05% TFA, linear gradient A/B = 25/75 to 50/50 over 50 min, flow rate: 3.0 mL/min, detection: photodiode array detector 199–651 nm) and 2nd reversed-phase HPLC (column: Inertsil C8-3 10 × 250 mm, eluent A: MeOH + 0.05% TFA, eluent B: H_2O + 0.05% TFA, linear gradient A/B = 45/55 to 70/30 over 50 min, flow rate: 3.0 mL/min, detection: photodiode array detector 199–651 nm) to give **C5** (t_R = 32.2–34.6 min, 1.54 mg, 0.913 μmol, 4.3% over 25 steps): white solid; HRMS (ESI) calcd for $C_{80}H_{110}N_{19}O_{22}$ $[M+H]^+$ 1688.8067, found 1688.8095.

General procedures for menaquinone complexation assay. To the bead-linked lysocin E analogues in LibraTube was added a solution of menaquinone-4 (**2**) in MeOH (10 mM, 1.0 mL/1000 beads). The mixture was incubated at room temperature for 12 h. A suspension of the beads in MeOH (200 beads/200 μL) was transferred to a 2.0 mL microtube.

Each bead in the microtube was transferred to each well of a 96-well filtration plate (278011, Thermo Fisher Scientific, 1 bead/well) with the solution of **2** (20 μL/bead) using a micropipette. To each well was added MeOH (400 μL), and the beads was filtered by gravity flow (× 3). The beads in the plate were dried under vacuum for 0.5–1 h. The dried beads were transferred to a 96-well PCR plate (652270, Greiner bio-one, 1 bead/well) with MeOH (20 μL/well) using a micropipette. The solution was removed under a stream of Ar at 40 ºC to give the **2**-bound bead-linked peptides.

To the **2**-bound bead-linked peptides in the plate was added *n*-BuOH (30 μL/well) at room temperature. The suspension was incubated at 50 ºC for 1 h. The resultant mixture in the plate was centrifuged at 3500 × *g* at room temperature for 10 sec. The supernatant in each well was transferred to the corresponding well of a black polystyrene flat-bottom 96-well plate (655086, Greiner bio-one). The solution was concentrated under a stream of Ar at 40 ºC to give the eluted **2**.

To the above eluted **2** in the plate was added *n*-BuOH (25 μL/well) at room temperature. The plate was vortexed at room temperature for 10 min. To the resultant solution was added a solution of $NaBH_4$ in *n*-BuOH (1.0 mM, 25 μL/well). The plate was vortexed at room temperature for 10 min. The fluorescence of menahydroquinone (**2H**, Ex. 250 nm/Em. 430 nm) of each well was measured at room temperature every 3 min (0, 3, and 6 min) on Gemini EM microplate reader (Molecular Devices). The three fluorescence intensities of each bead were averaged and plotted against the bead number.

Evaluation of menaquinone complexation activities of the selected bead-linked peptides. Bead-linked peptides **30**, **BLP1**, and **BLP2** were synthesized according to the above described procedures (loading rate: 0.222 mmol/g). The bead-linked peptides were subjected to the MK complexation assay described above. The obtained fluorescent intensities of **2H** (arbitrary unit) were 1026 ± 64 for **30**, 396 ± 27 for **BLP1**, 72.9 ± 2.0 for **BLP2**, and 41.6 ± 0.1 for background adsorption (no bead), respectively (Supplementary Figure 3).

Preparation of beads for cleavage reaction. After the menaquinone complexation assay, the beads were washed for the cleavage reaction as follows:

The beads in a 96-well PCR plate (1 bead/well) were transferred to a filtration plate (1 bead/well) with MeOH (50 μL/bead) by using a micropipette. To each well was added DMSO (400 μL), and the bead was filtered by gravity flow (× 3). To each well was added MeOH (400 μL), and the bead was filtered by gravity flow (× 2). The beads in the plate were dried under vacuum for 1 h. The dried beads were transferred to a 96-well PCR plate (1 bead/well) with MeOH (20 μL/bead) by using a micropipette. The suspension was dried under a stream of Ar at 40 ºC to give the dried beads, which were used in the cleavage reaction.

Cleavage of 2401 peptides from beads. To the above washed bead-linked peptides in the 96-well PCR plate (1 bead/well) was added MeOH (20 μL/well). The microplate was irradiated with UV light (λ = 365 nm) using a handy UV lamp (AS ONE, SLUV-6, 6 W) at room temperature for 1 h by shifting the lamp position by two lines every 15 min (Supplementary Figure 5). The resultant solution was concentrated under a stream of Ar at 40 ºC to give the crude lysocin E analogues (1 peptide/well).

Separation of peptides for antimicrobial activity assay and MS/MS sequencing analysis. To the crude peptides in the 96-well PCR plate was added MeOH (100 μL/well). The plate was capped with 8-cap strips, and sonicated at room temperature for 1 min. The resultant mixture in the plate was centrifuged at 3500 × *g* at room temperature for 10 sec. The 8-cap strips were carefully removed from the plate. After gentle pipetting of the solution (× 3), the solution of the crude peptide in MeOH (85 μL/well) was transferred to a 96-well round-bottom plate (3367, Corning). The solution in the round-bottom plate (85 μL/well) was dried a stream of Ar at 40 ºC to give the crude peptides, which were used for antimicrobial activity assay. The remaining solution (15 μL/well) in the PCR plate was dried under a stream of Ar at 40 ºC to give the crude peptides, which were used for MS/MS sequencing analysis.

MS/MS sequencing analysis. To the crude peptides in the 96-well PCR plates was added MeOH/H_2O (1/9, 20 μL/well) containing 1% NH_3. The mixtures were incubated at room temperature for 12–14 h. The reaction mixtures were concentrated under a stream of Ar at 40 ºC to give the hydrolyzed linear peptides.

The crude linear peptides were dissolved in a solution of α-cyano-4-hydroxycinnamic acid in MeCN/H_2O (7/3) containing 0.1% TFA (2.5 μL/well, 4.0 mg/mL) and a solution of diammonium hydrogen citrate in H_2O (0.0025 μL/well, 100 mg/mL). The resultant solution of peptides was subjected to the MS/MS sequencing analysis on TOF/TOF 5800 (AB Sciex).

Antimicrobial activity assay of bead-derived peptides. To the crude peptides in the 96-well round-bottom plate was added DMSO (1 μL/well). To the solution of peptides in each well was added a suspension of *S. aureus* Smith ATCC 13709 strain in 10% calf serum containing cation adjusted Müller Hinton Broth [40 μL, 2 $\times 10^5$ colony forming units (CFU), 21 g/L Müller Hinton Broth (Difco), 50 mg/L Ca^{2+} (adjusted with $CaCl_2 \cdot 2H_2O$), and 25 mg/L Mg^{2+} (adjusted with $MgCl_2 \cdot 6H_2O$)]. The plate was incubated at 37 ºC for 18 h, and the precipitation of the grown cells was examined.

Evaluation of antimicrobial spectra of peptides. The MIC assay was performed according to the Clinical and Laboratory Standards Institute protocols. The antimicrobial activities of the peptides were measured using the broth microdilution method.[1] Serial dilutions of peptides were prepared in cation adjusted Müller Hinton Broth. Each dilution (100 μL) was dispensed to each well of the round-bottom plate to obtain final concentrations ranging from 64 μg/mL to 0.0075 μg/mL. Bacteria were grown at 37ºC overnight on Tryptic Soy Broth or Müller Hinton Broth agar plates. Bacterial inoculum was prepared by direct suspension of the colonies in 0.9% saline, and adjusted to obtain OD_{625} 0.08–0.13 using a spectrophotometer UV-1280 (Shimadzu). The resultant suspension was diluted with cation adjusted Müller Hinton Broth (1/20). The resultant mixture (10 μL) was inoculated to each well of the plates (approximately 5×10^4 CFU/well), mixed, and incubated at 37 ºC for 18 h. The MIC value was determined as the minimum concentration that inhibited growth of bacteria. To evaluate the antimicrobial activities of peptides in the presence of serum, 10% bovine calf serum in cation adjusted Müller Hinton Broth was used.

Preparation of LUVs consisting of PC/PG. Carboxyfluorescein-encapsulated large unilamellar vesicles (LUVs) were prepared according to the thin-film hydration method, followed by extrusion through Nuclepore polycarbonate filters (GE Healthcare) mounted in the mini-extruder apparatus (Avanti Polar Lipids). A solution of egg yolk phosphatidylcholine (9.60 mg, 12.5 μmol) and egg yolk phosphatidylglycerol (9.78 mg, 12.5 μmol) in $CHCl_3$ (0.505 mL) was concentrated, and dried under vacuum to form a lipid thin film. The thin film was hydrated and suspended in carboxyfluorescein-containing buffer solution [0.500 mL, 5 mM 4-(2-hydroxyethyl)-1-piperazineethanesulfonic acid (HEPES), 20 mM carboxyfluorescein, pH 7.5] by vortexing and sonication. After five-times freeze-thaw cycles, the lipid suspension was extruded 19 times through a polycarbonate filter with 0.40 μm of pore size in the diameter. The external carboxyfluorescein-containing buffer was replaced with a carboxyfluorescein-free buffer solution [20 mM HEPES, 1 mM ethylenediaminetetraacetic acid (EDTA), pH 7.5] through size exclusion chromatography using disposable PD-10 column (GE Healthcare). Concentration of egg yolk phosphatidylcholine was determined by using Phospholipid C-Test Wako (Fujifilm Wako Pure Chemical). The final concentration of egg yolk phosphatidylcholine was adjusted to 4.0 μM with the carboxyfluorescein-free buffer solution, and the solution was used for membrane disruption assay.

Preparation of LUVs consisting of PC/PG/MK-4 or PC/PG/UQ-10. To a solution of egg yolk phosphatidylcholine (9.48 mg, 12.4 μmol) and egg yolk phosphatidylglycerol (9.66 mg, 12.4 μmol) in $CHCl_3$ (0.208 mL) was added a solution of MK-4 (**2**) or UQ-10 (**3**) (0.313 μmol) in $CHCl_3$ (21.8 μL). The solution was concentrated, and dried under vacuum to form a lipid thin film. The thin film was hydrated and suspended in carboxyfluorescein-containing buffer solution (0.500 mL, 5 mM HEPES, 20 mM carboxyfluorescein, pH 7.5) by vortexing and sonication. After five-times freeze-thaw cycles, the lipid suspension was extruded 19 times through a polycarbonate filter with 0.40 μm of pore size in the diameter. The external carboxyfluorescein-containing buffer was replaced with a carboxyfluorescein-free buffer solution (20 mM HEPES, 1 mM EDTA, pH 7.5) through size exclusion chromatography using disposable PD-10 column. Concentration of egg yolk phosphatidylcholine was determined by using Phospholipid C-Test Wako. The final concentration of egg yolk phosphatidylcholine was adjusted to 4.0 μM with the carboxyfluorescein-free buffer solution, and the solution was used for membrane disruption assay.

Membrane disruption assay. Peptides **1**, **A1**–**A22**, **B1**–**B5**, and **C1**–**C5** were diluted with MeOH/H_2O (4/6) to various concentrations as 5-fold serial dilutions. The solution of peptides (2 μL/well) and the carboxyfluorescein-encapsulated LUV suspension (200 μL/well) were mixed in black polystyrene flat-bottom 96-well plates. The final concentration of peptides ranged from 2970 nM to 0.950 nM. The plates were vortexed at room temperature for 30 min, and then fluorescence of carboxyfluorescein (Ex. 490 nm/Em. 517 nm) of each well was measured at room temperature on Gemini EM microplate reader. Trion X-100/H_2O (5/95, 2 μL), MeOH/H_2O (4/6, 2 μL), and the LUV suspension were mixed in each plate to determine 100% lysis. MeOH/H_2O (4/6, 2 μL) and the LUV suspension were mixed in each plate to determine 0% lysis. The membrane disruption activities of tested peptides (I) were normalized against 100% lysis by Triton X-100

(I_{max}) and 0% lysis by MeOH/H_2O (4/6) (I_0) as following, where I_x is the fluorescence intensity (Supplementary Equation 2).

$$I = 100 \times (I_x - I_0) / (I_{max} - I_0) \quad (2)$$

The membrane-disrupting activities were evaluated as half-maximal response (EC_{50}, nM) values by means of three replicates. Sigmoidal curve fittings were performed on R^2 with drc package (Supplementary Figs S7–S9).[3] Two-parameter logistic model was applied for the fitting.

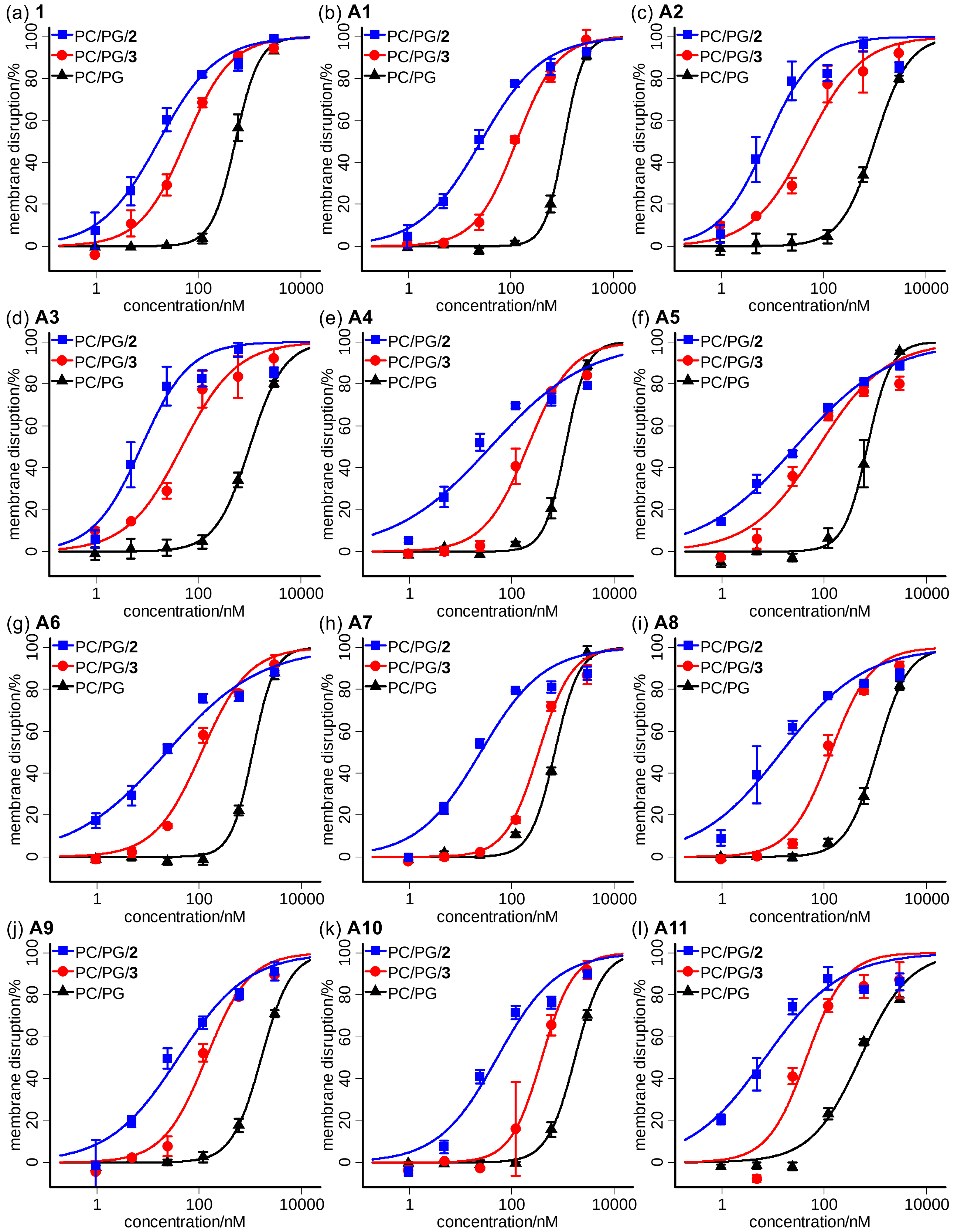

Supplementary Figure 7. Concentration-response curves for membrane disrupting activities of peptides. Representative curves of **1** and **A1**–**A11** against the three LUVs (PC/PG =50:50, PC/PG = 50:50 containing 1.25 mol% **2** or **3**) from three independent experiments were shown as mean ± SD. Source data are provided as a Source Data file.

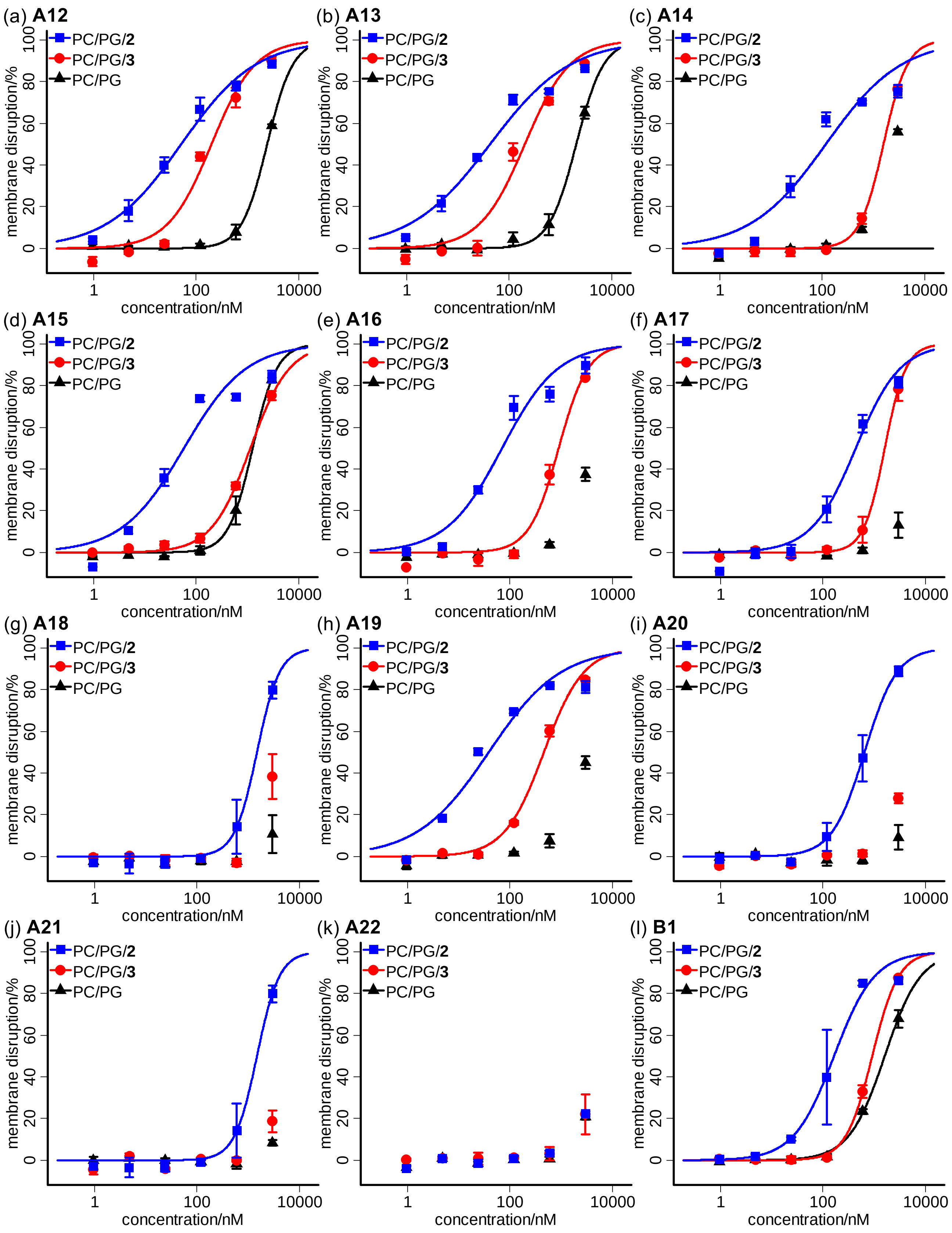

Supplementary Figure 8. Concentration-response curves for membrane disrupting activities of peptides. Representative curves of **A12**–**A22** and **B1** against the three LUVs (PC/PG =50:50, PC/PG = 50:50 containing 1.25 mol% **2** or **3**) from three independent experiments were shown as mean ± SD. Source data are provided as a Source Data file.

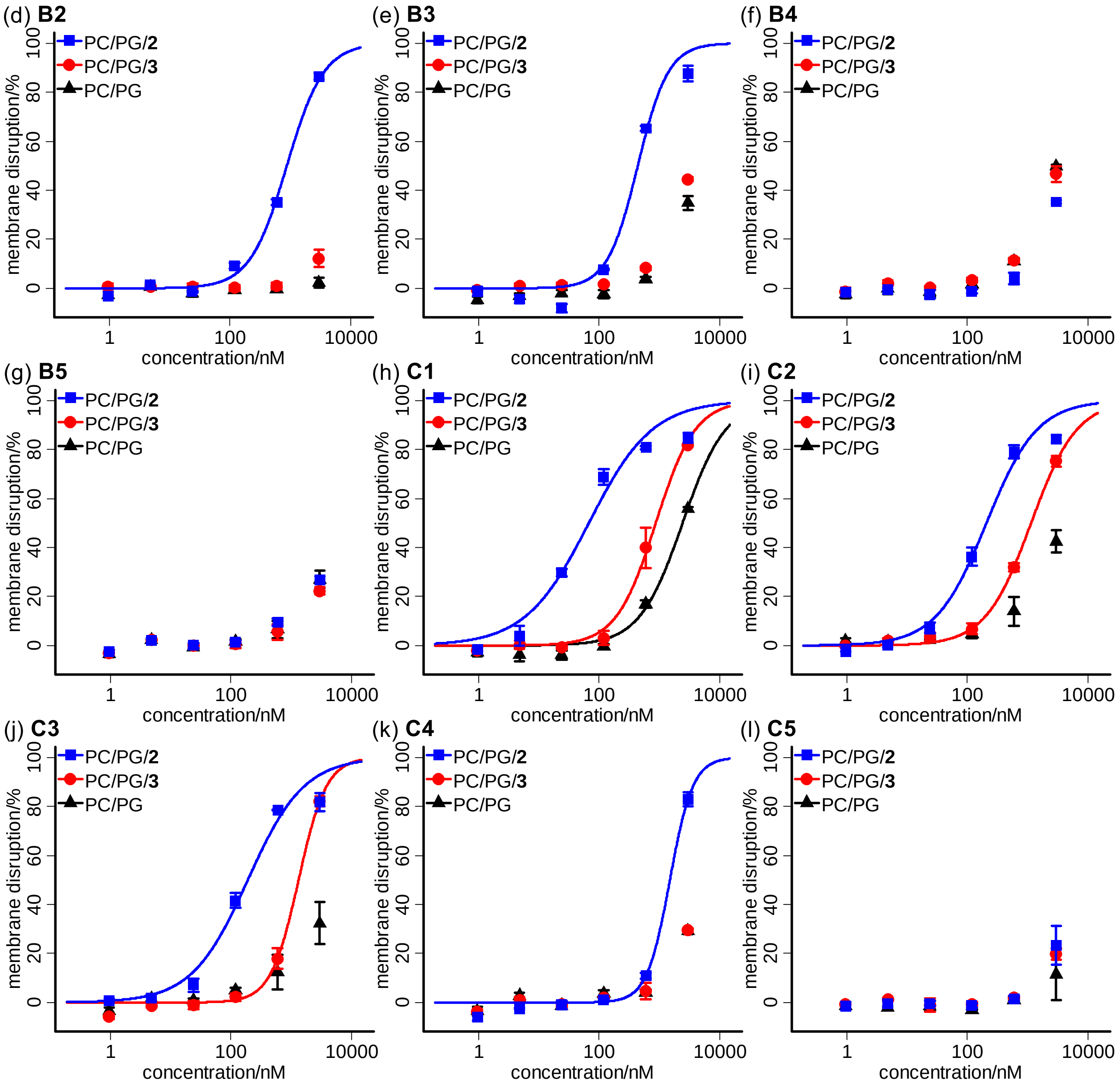

Supplementary Figure 9. Concentration-response curves for membrane disrupting activities of peptides. Representative curves of **B2**–**B5** and **C1**–**C5** against the three LUVs (PC/PG =50:50, PC/PG = 50:50 containing 1.25 mol% **2** or **3**) from three independent experiments were shown as mean ± SD. Source data are provided as a Source Data file.

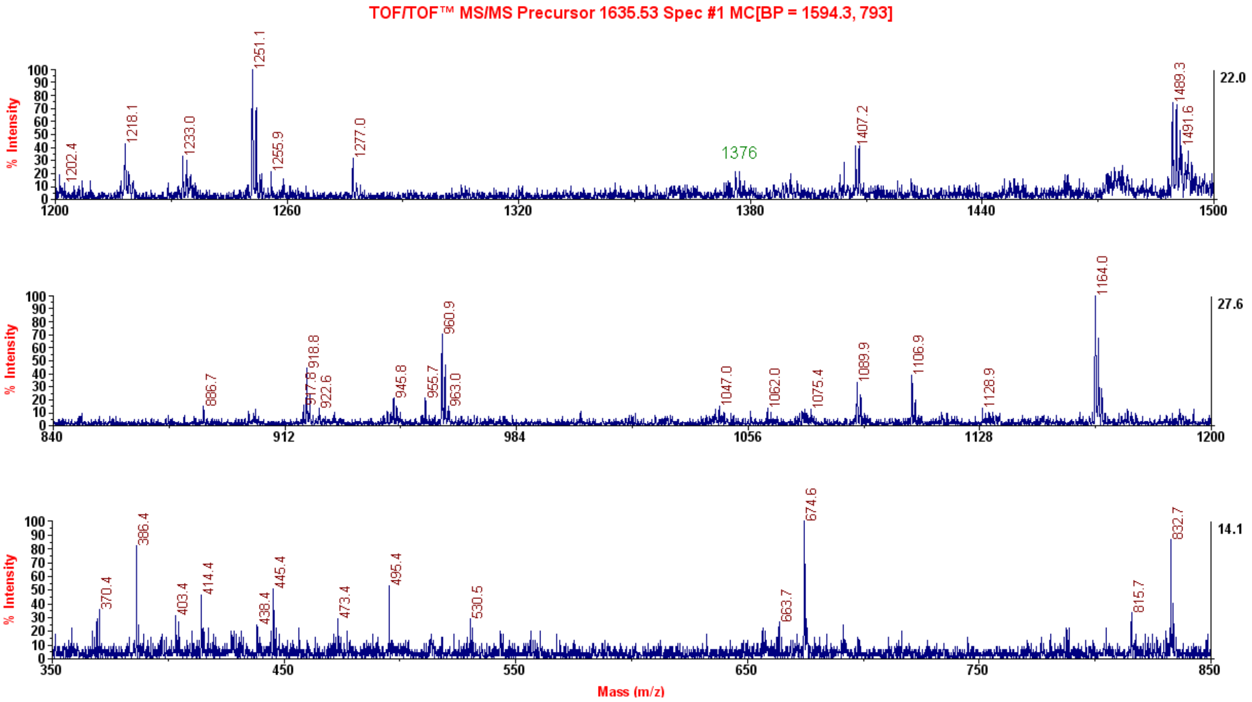

Supplementary Figure 10. MS/MS spectrum of **1**. Mass range: *m*/*z* 350–1500.

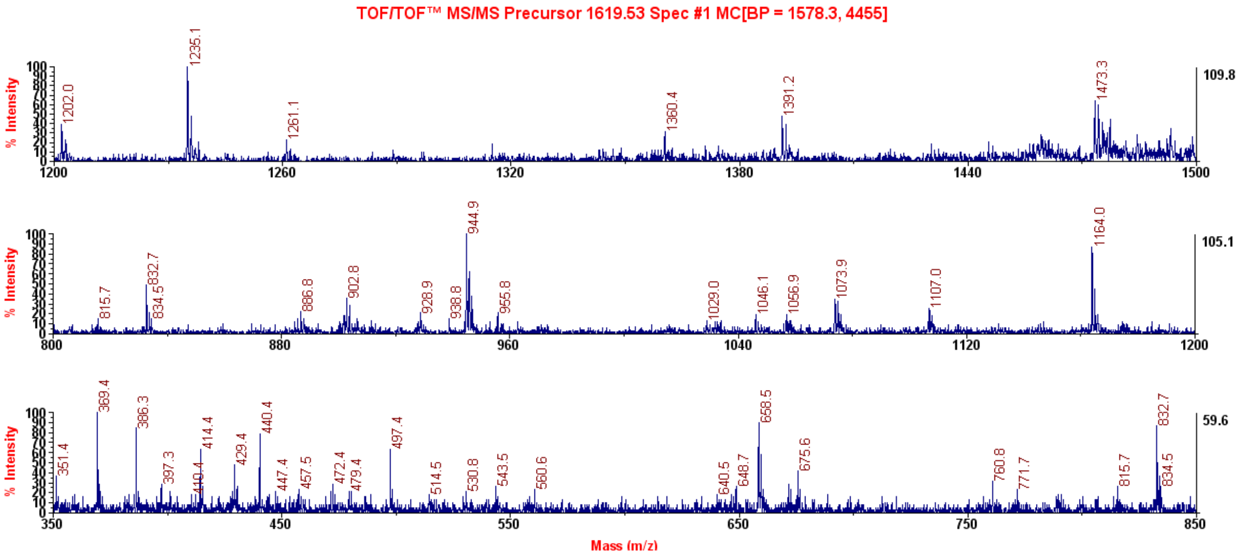

Supplementary Figure 11. MS/MS spectrum of **A1**. Mass range: *m*/*z* 350–1500.

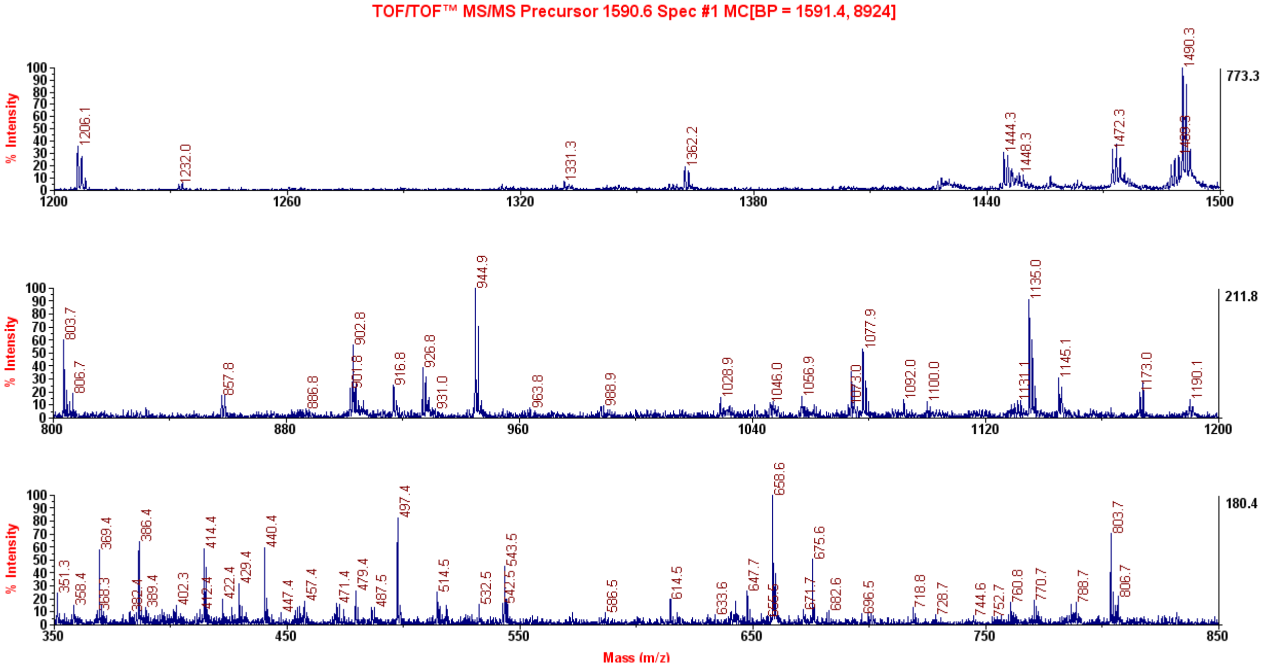

Supplementary Figure 12. MS/MS spectrum of **A2**. Mass range: *m*/*z* 350–1500.

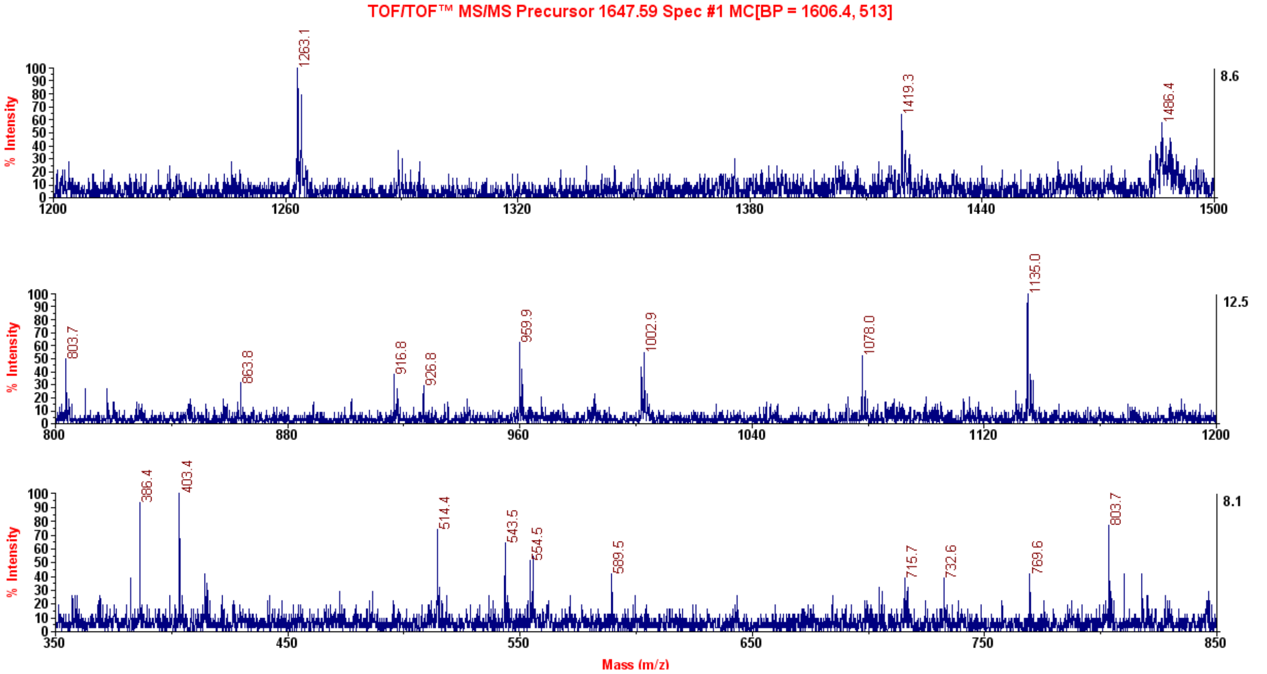

Supplementary Figure 13. MS/MS spectrum of **A3**. Mass range: *m*/*z* 350–1500.

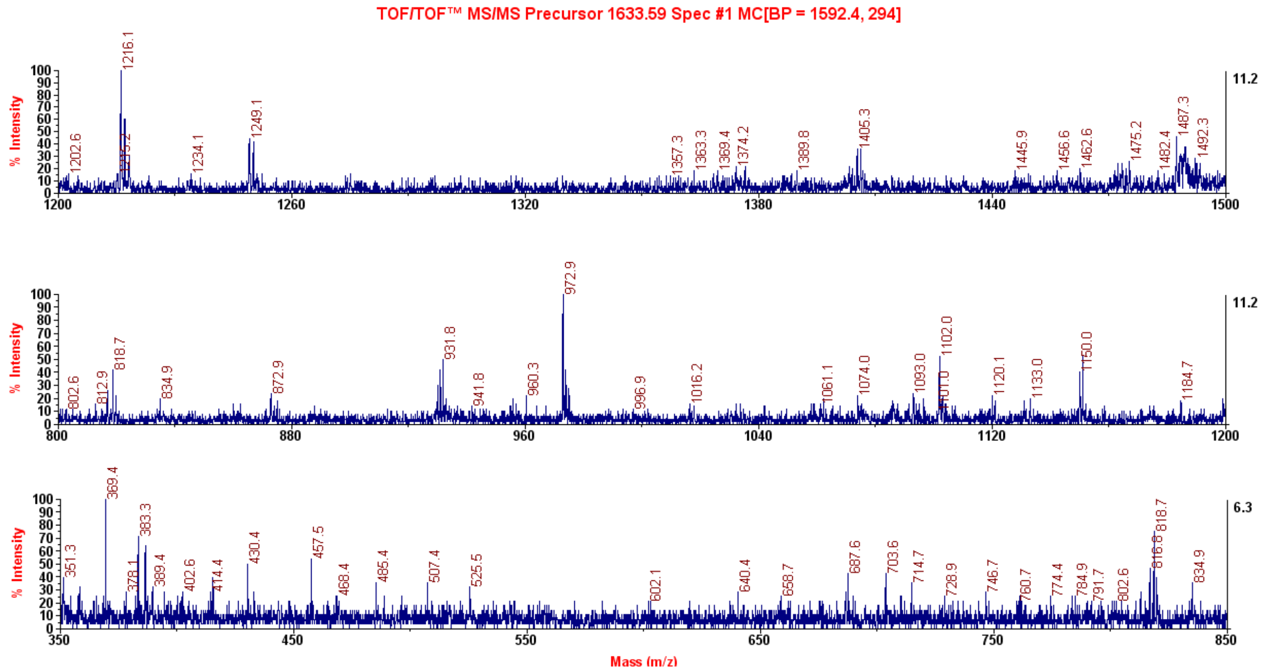

Supplementary Figure 14. MS/MS spectrum of **A4**. Mass range: *m*/*z* 350–1500.

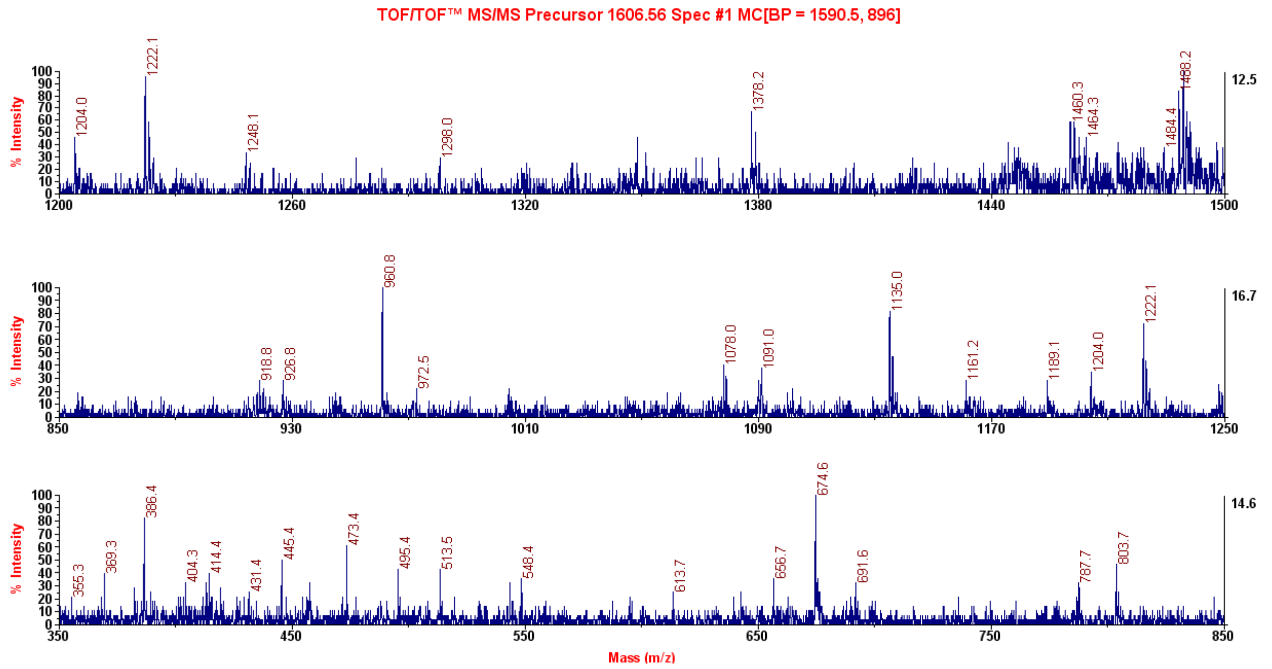

Supplementary Figure 15. MS/MS spectrum of **A5**. Mass range: *m*/*z* 350–1500.

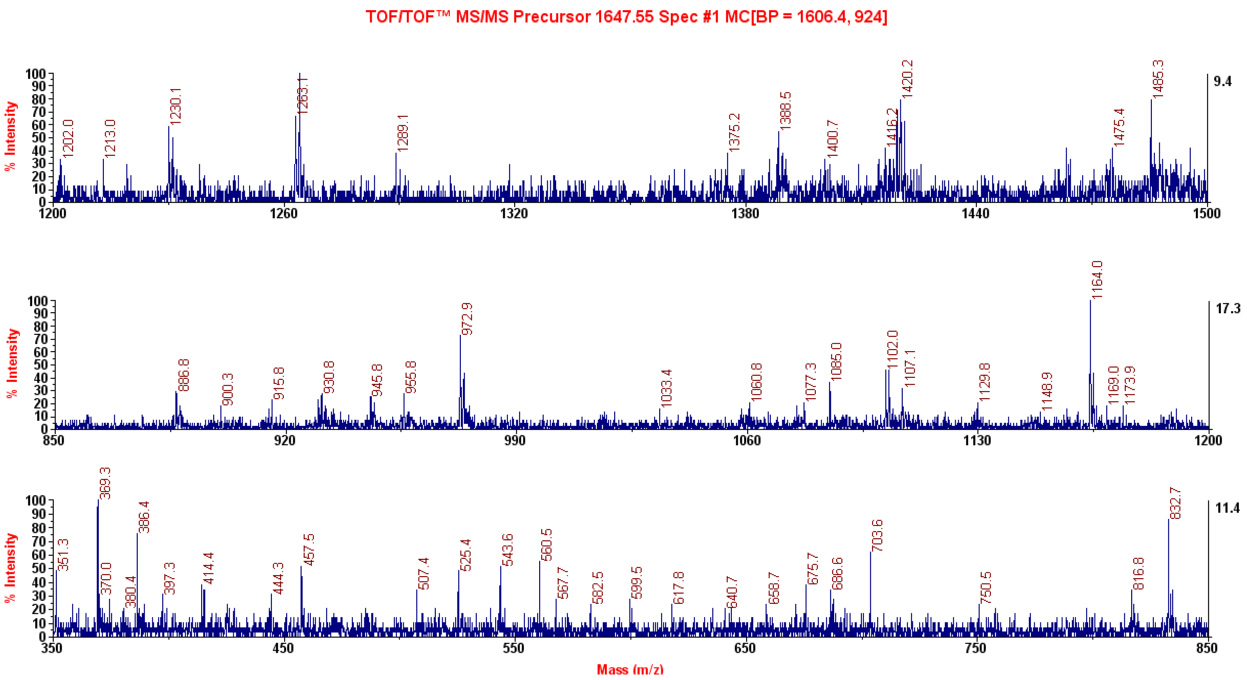

Supplementary Figure 16. MS/MS spectrum of **A6**. Mass range: *m*/*z* 350–1500.

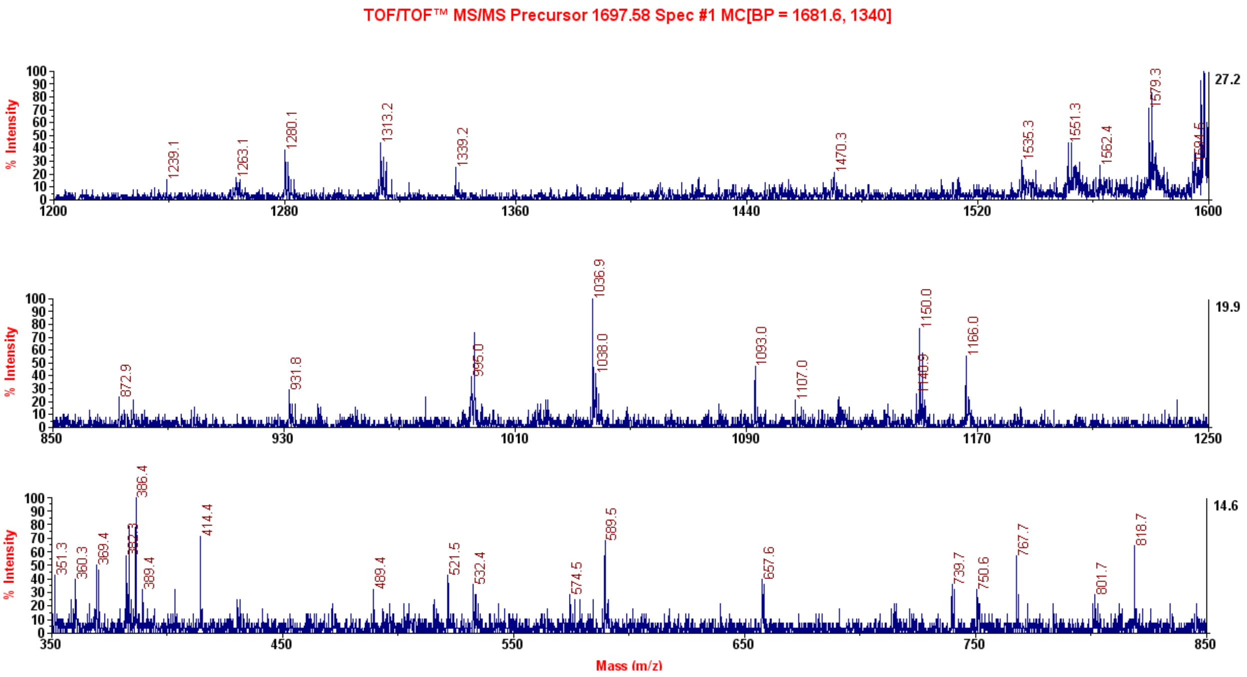

Supplementary Figure 17. MS/MS spectrum of **A7**. Mass range: *m*/*z* 350–1600.

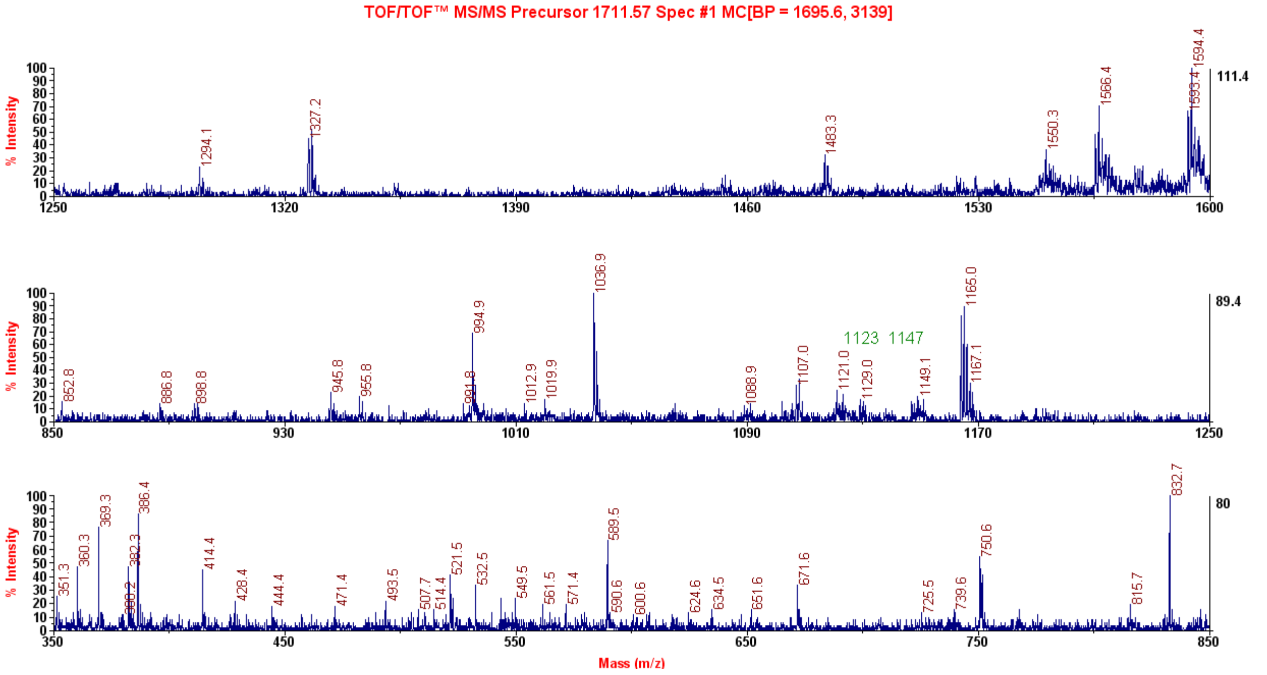

Supplementary Figure 18. MS/MS spectrum of **A8**. Mass range: *m*/*z* 350–1600.

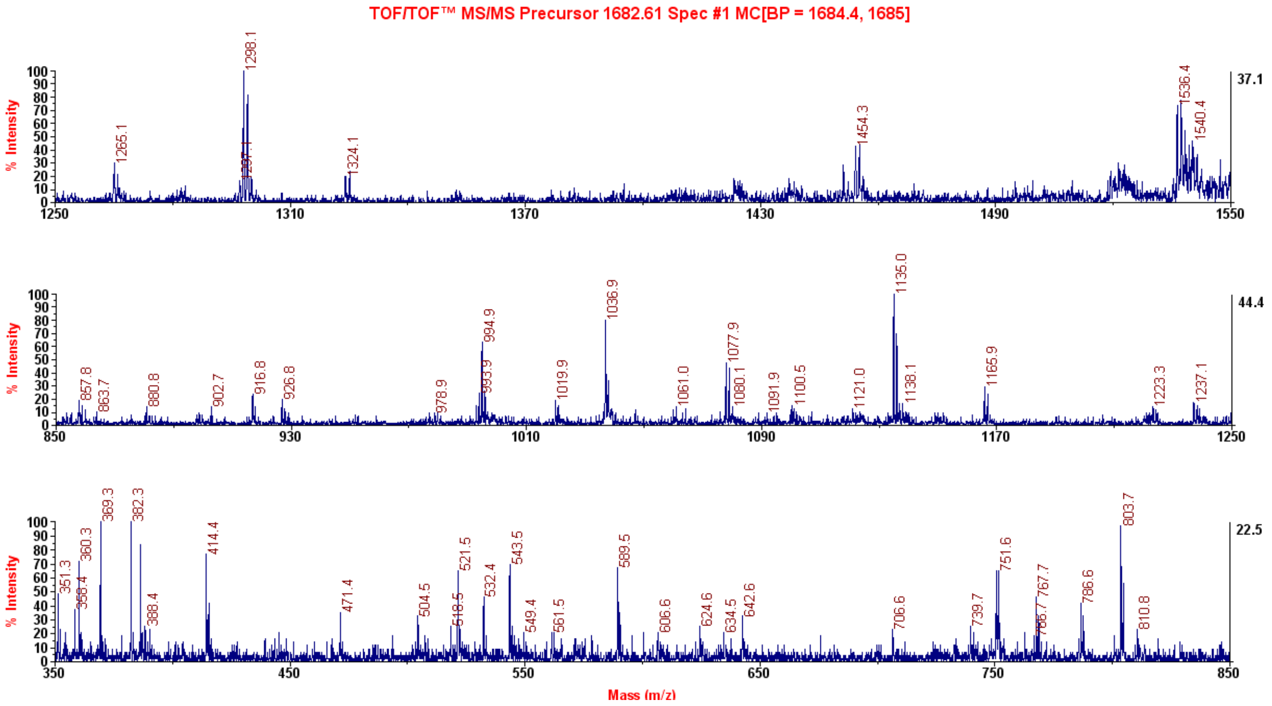

Supplementary Figure 19. MS/MS spectrum of **A9**. Mass range: *m*/*z* 350–1550.

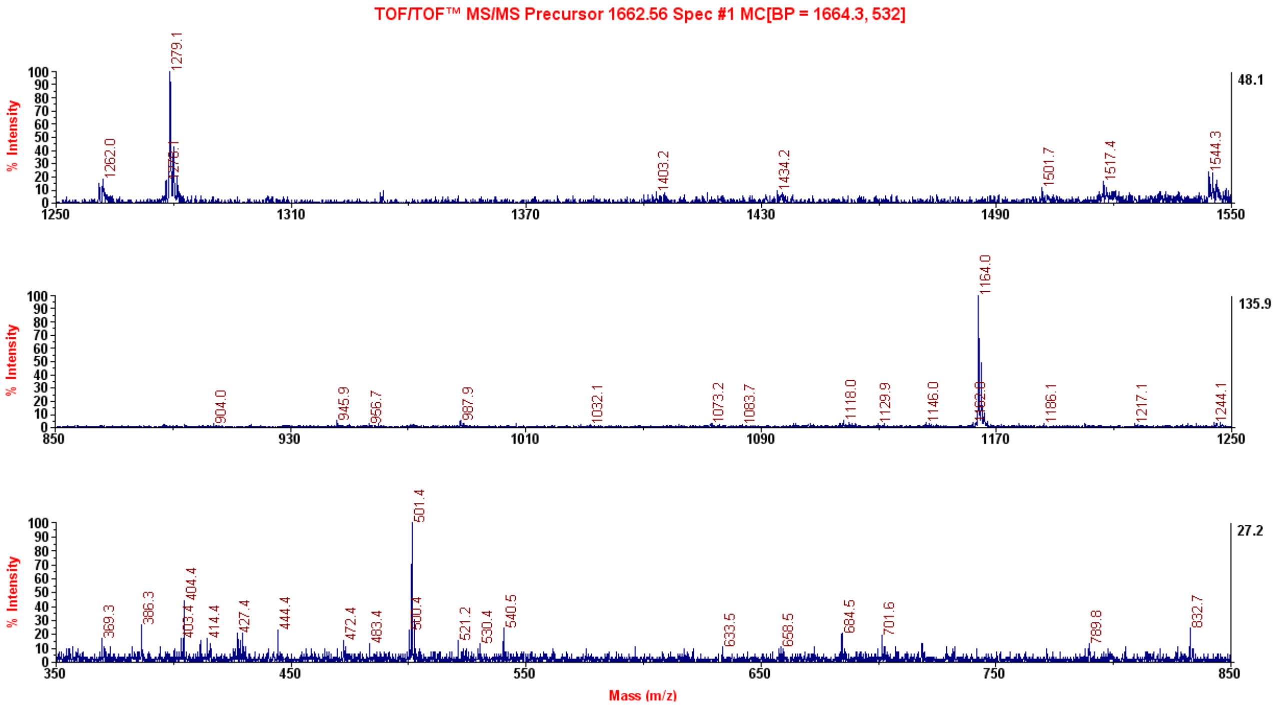

Supplementary Figure 20. MS/MS spectrum of **A10**. Mass range: *m*/*z* 350–1550.

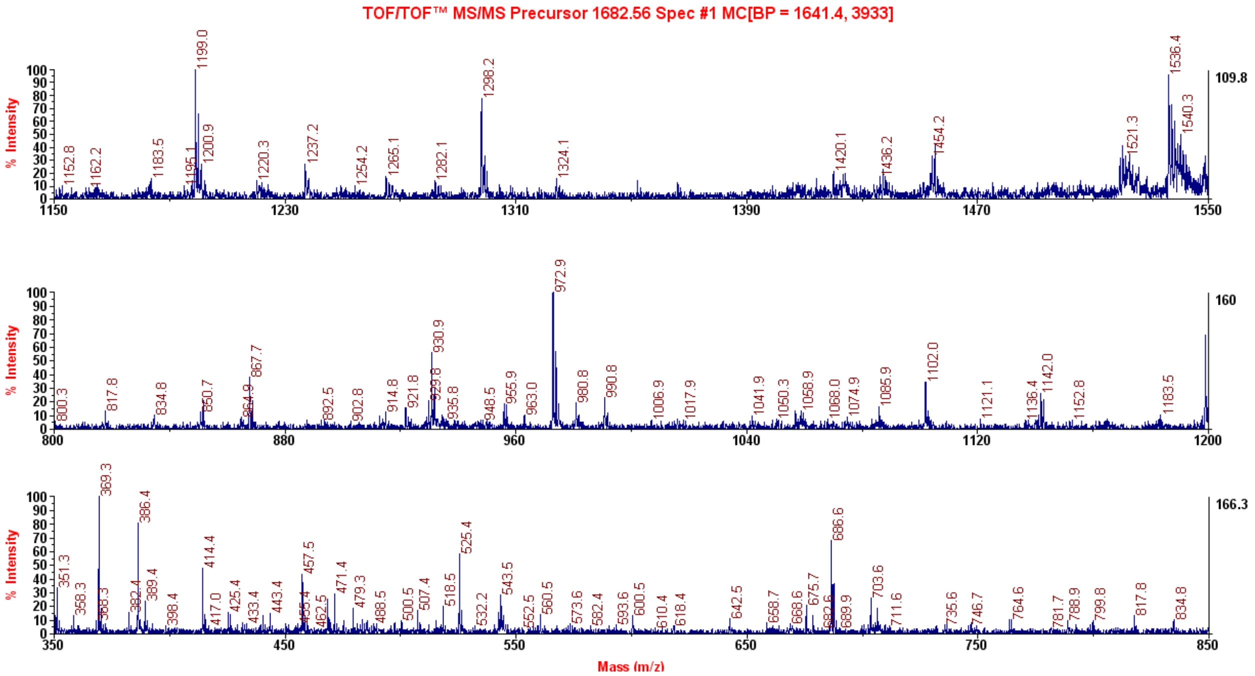

Supplementary Figure 21. MS/MS spectrum of **A11**. Mass range: *m*/*z* 350–1550.

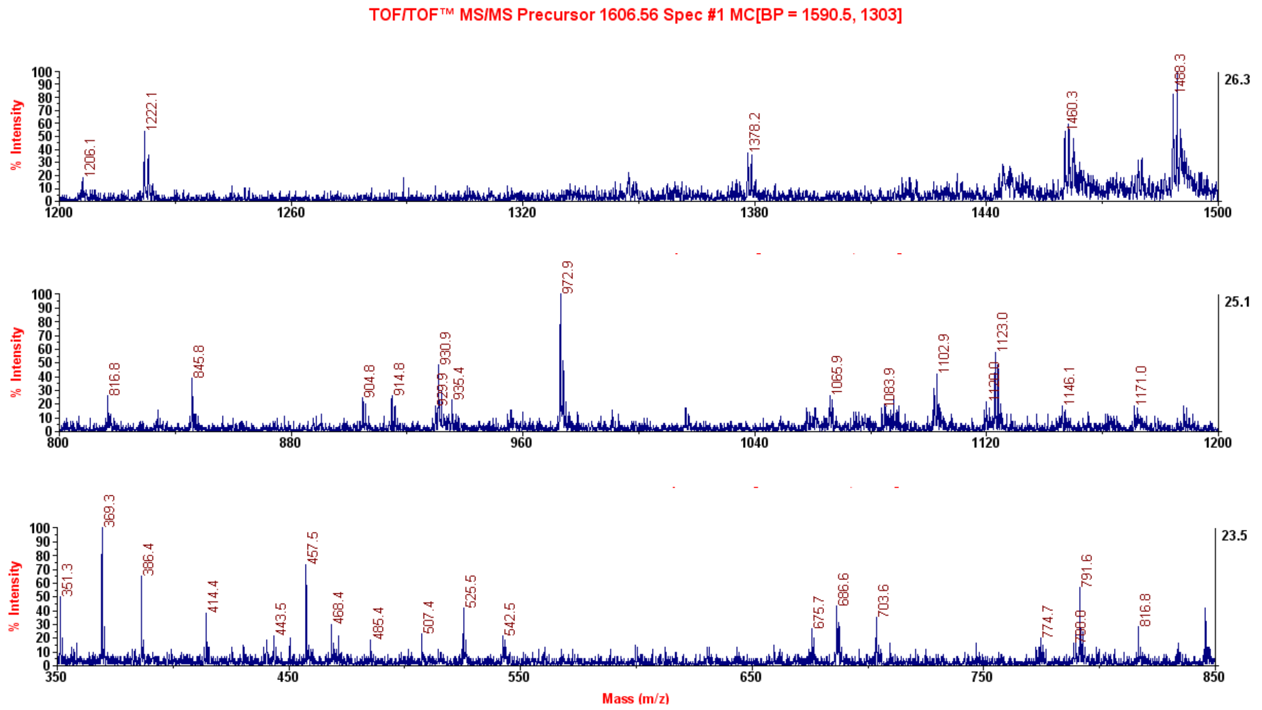

Supplementary Figure 22. MS/MS spectrum of **A12**. Mass range: *m*/*z* 350–1500.

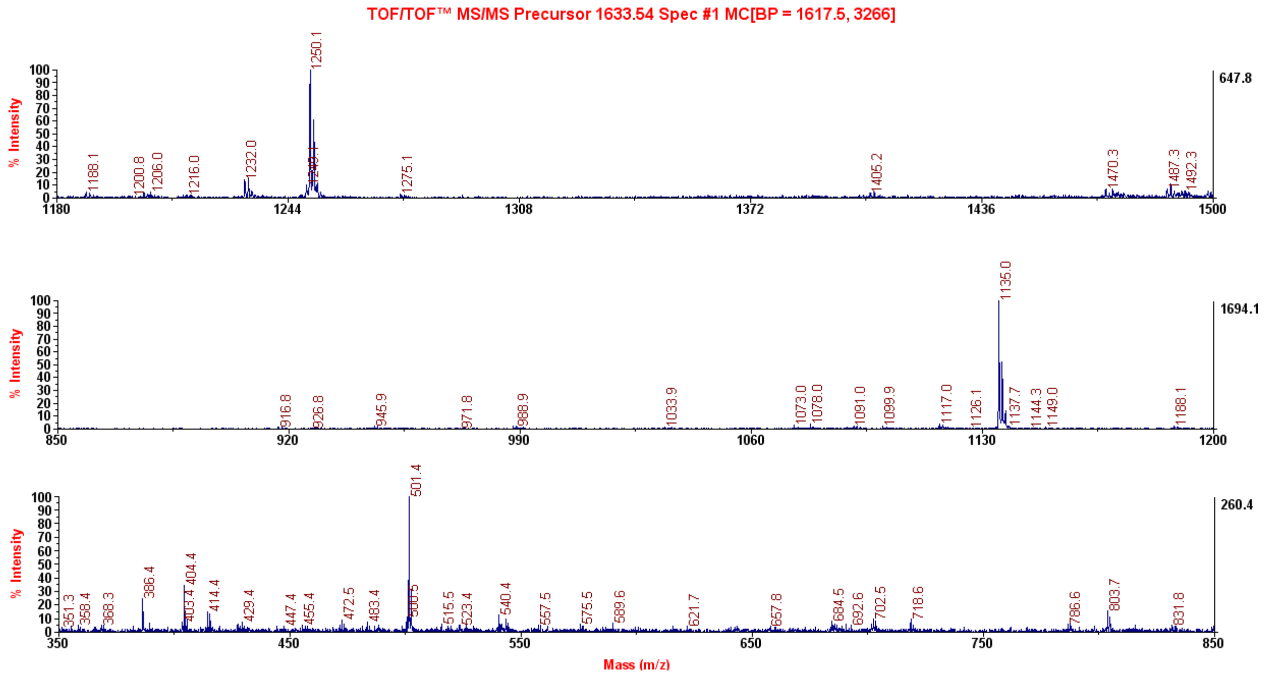

Supplementary Figure 23. MS/MS spectrum of **A13**. Mass range: *m*/*z* 350–1500.

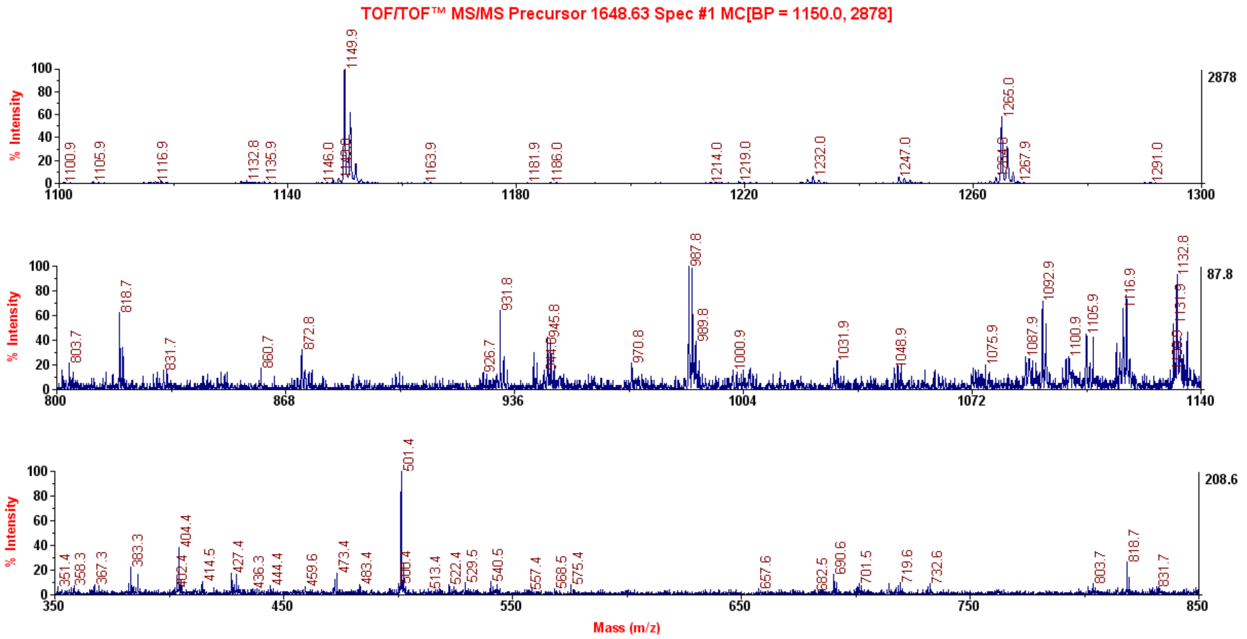

Supplementary Figure 24. MS/MS spectrum of **A14**. Mass range: *m*/*z* 350–1300.

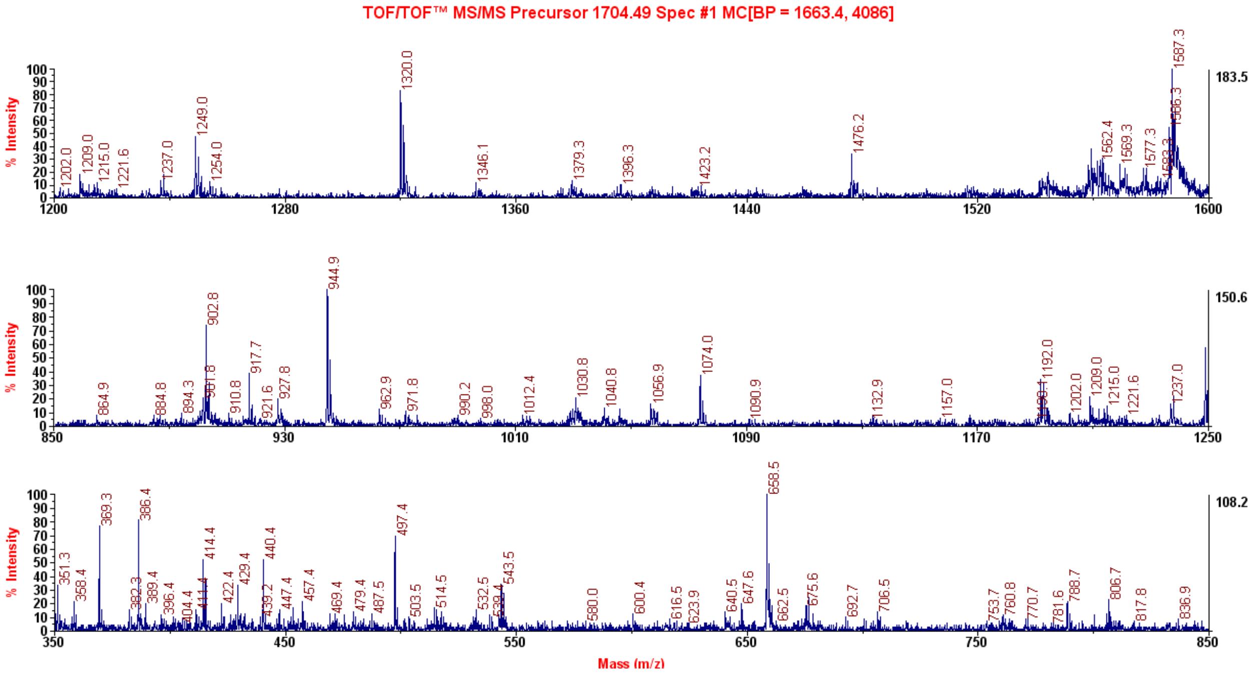

Supplementary Figure 25. MS/MS spectrum of **A15**. Mass range: *m*/*z* 350–1600.

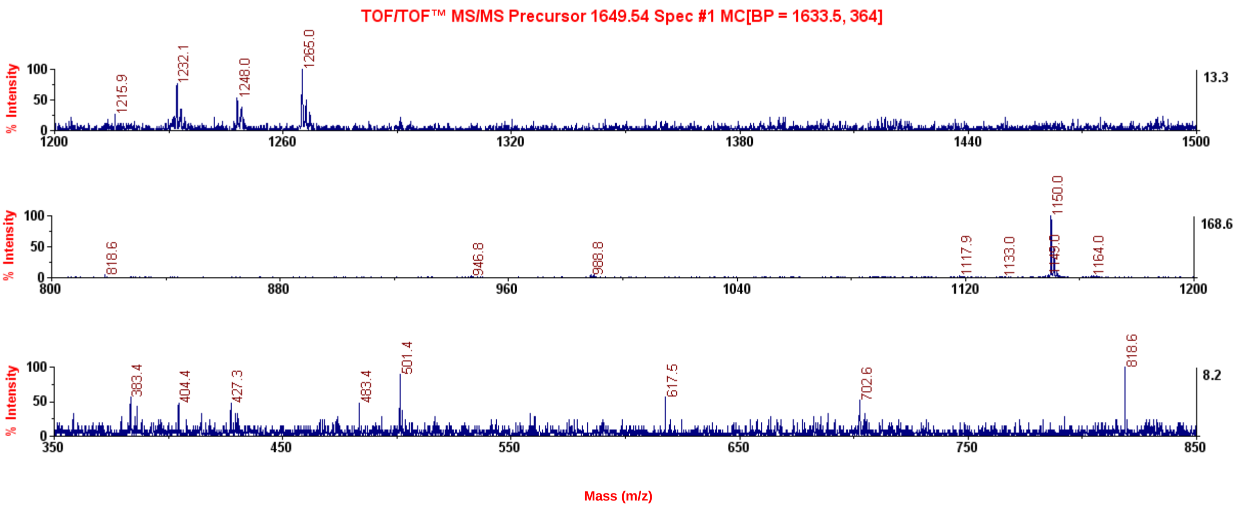

Supplementary Figure 26. MS/MS spectrum of **A16**. Mass range: *m*/*z* 350–1500.

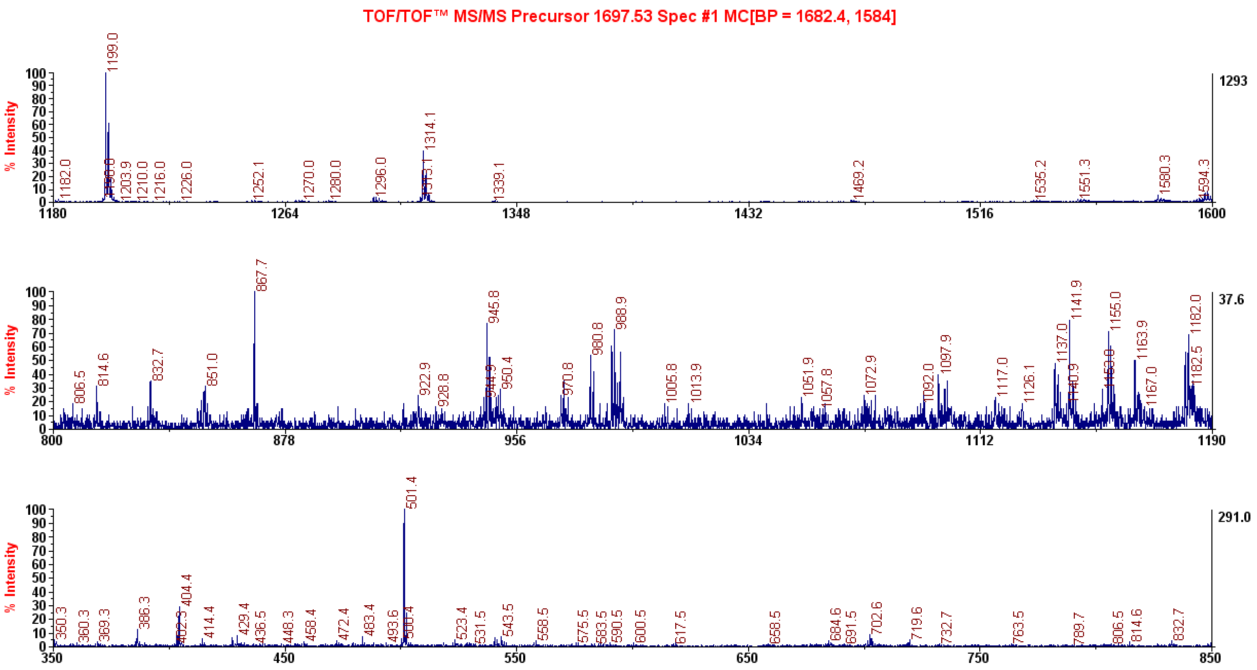

Supplementary Figure 27. MS/MS spectrum of **A17**. Mass range: *m*/*z* 350–1600.

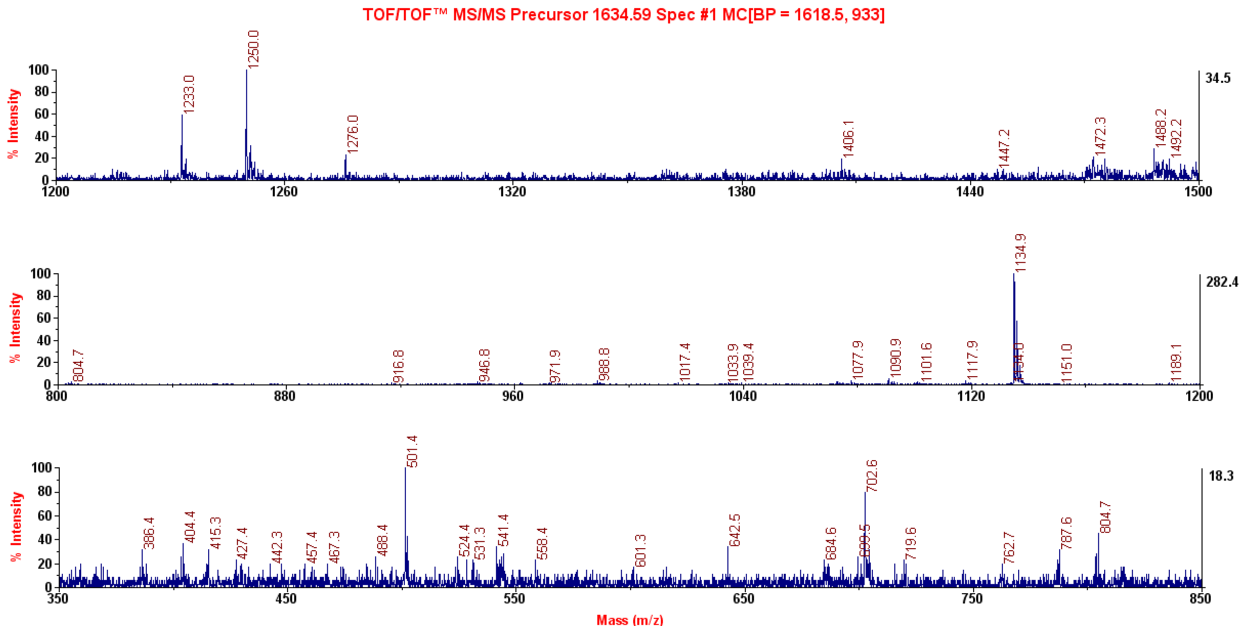

Supplementary Figure 28. MS/MS spectrum of **A18**. Mass range: *m*/*z* 350–1500.

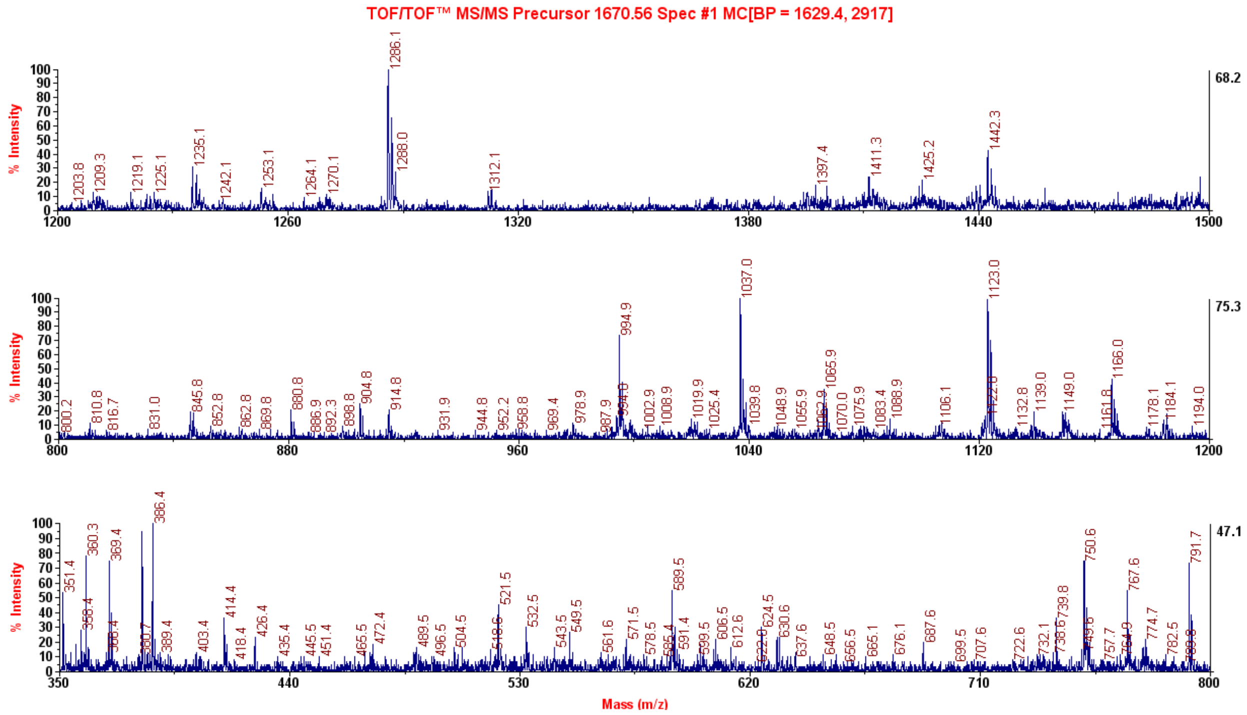

Supplementary Figure 29. MS/MS spectrum of **A19**. Mass range: *m*/*z* 350–1500.

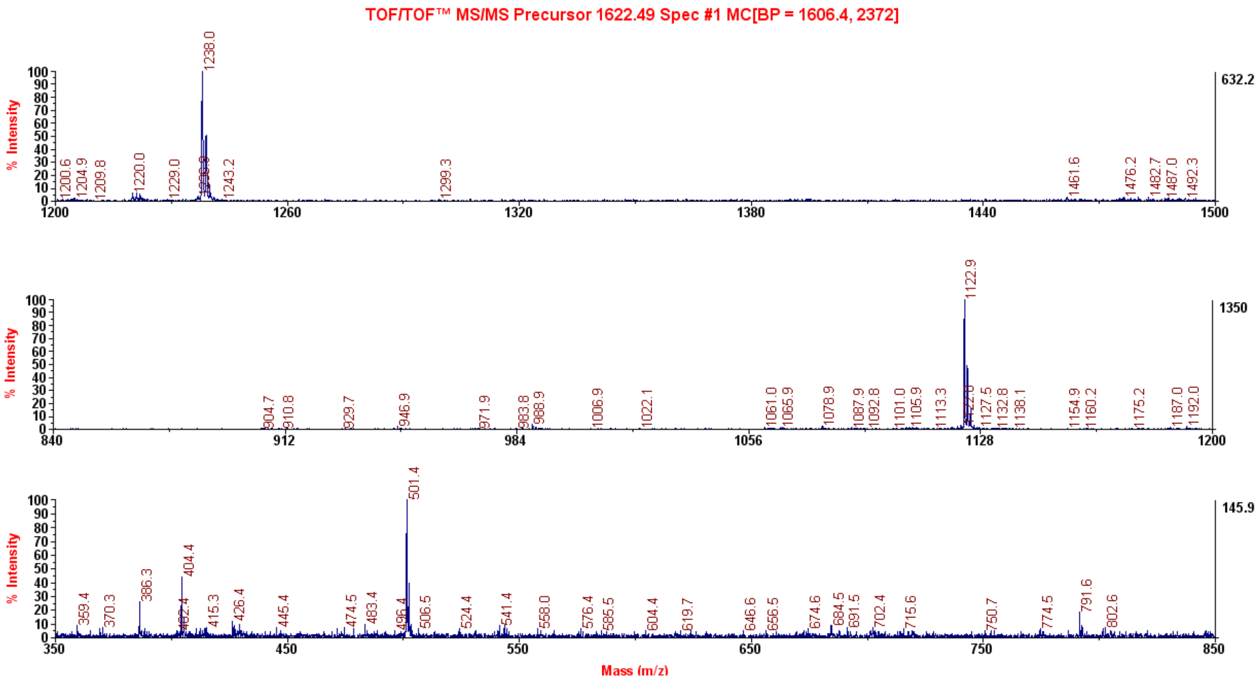

Supplementary Figure 30. MS/MS spectrum of **A20**. Mass range: *m*/*z* 350–1500.

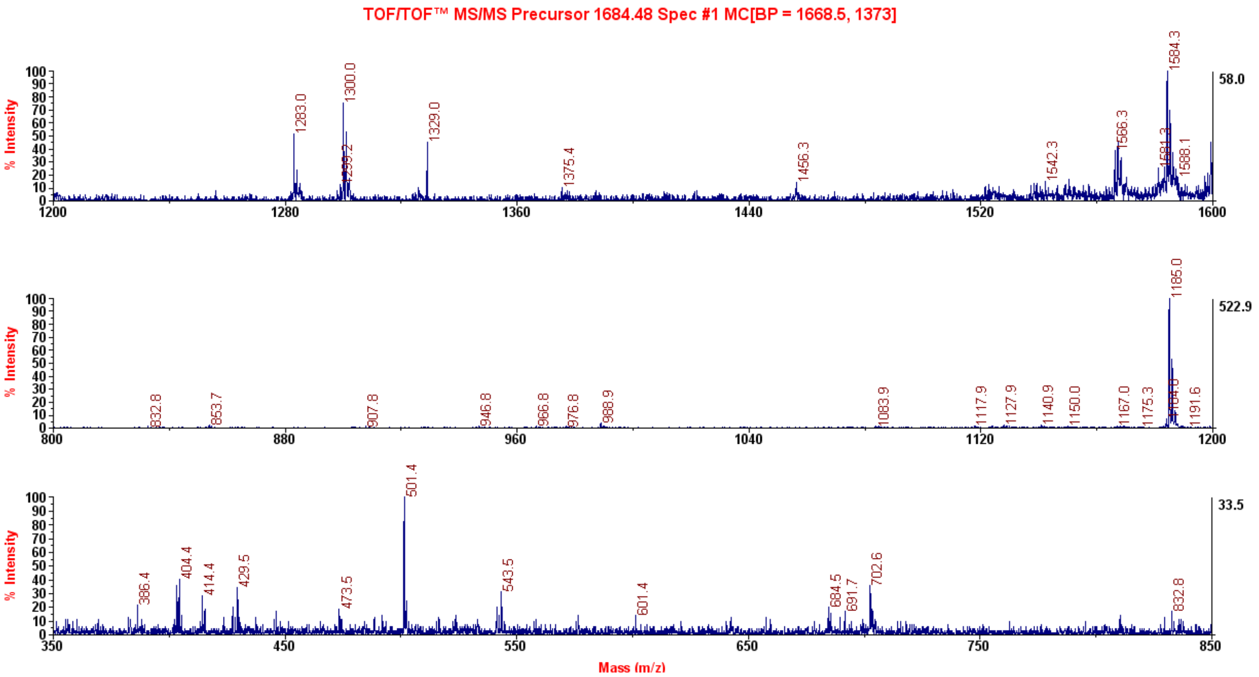

Supplementary Figure 31. MS/MS spectrum of **A21**. Mass range: *m*/*z* 350–1600.

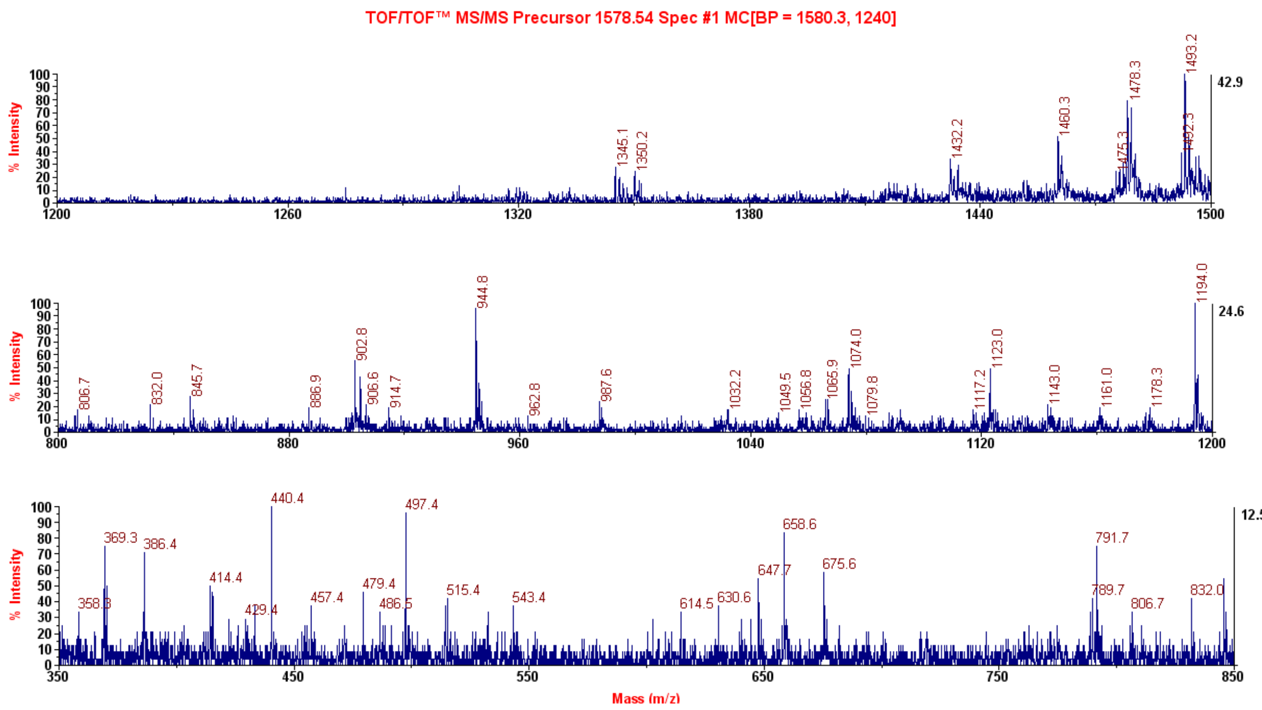

Supplementary Figure 32. MS/MS spectrum of **A22**. Mass range: *m*/*z* 350–1500.

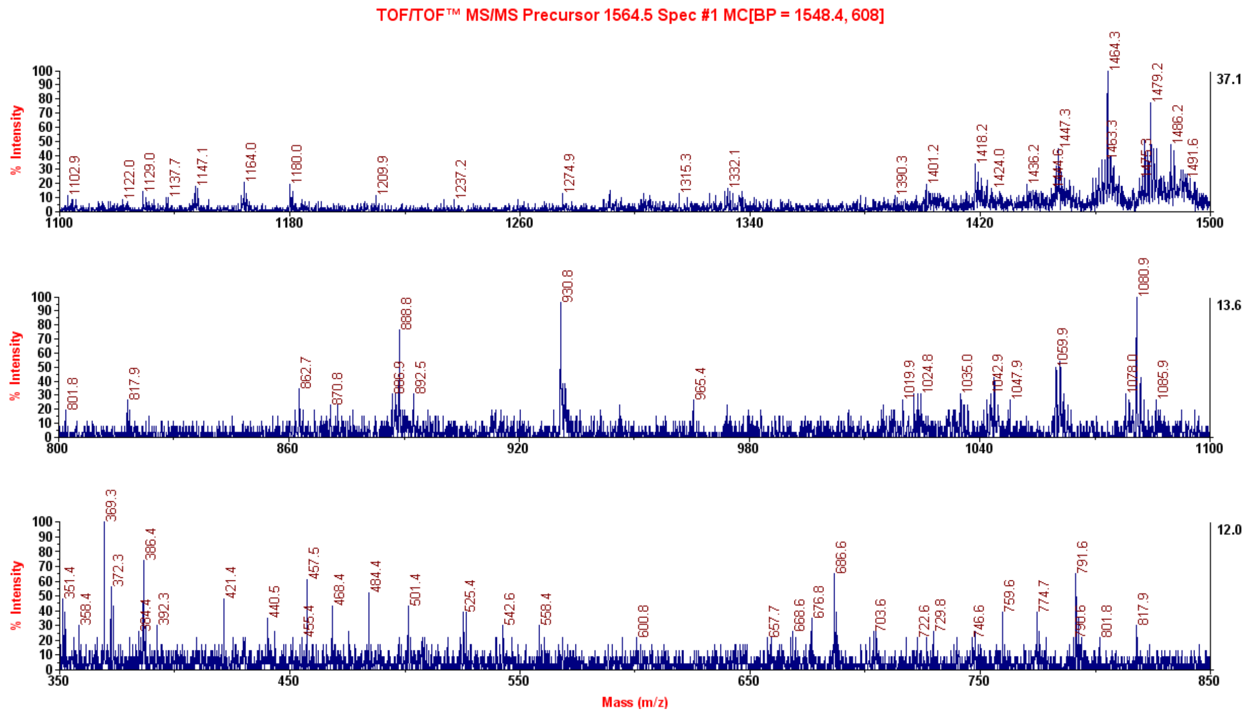

Supplementary Figure 33. MS/MS spectrum of **B1**. Mass range: *m*/*z* 350–1500.

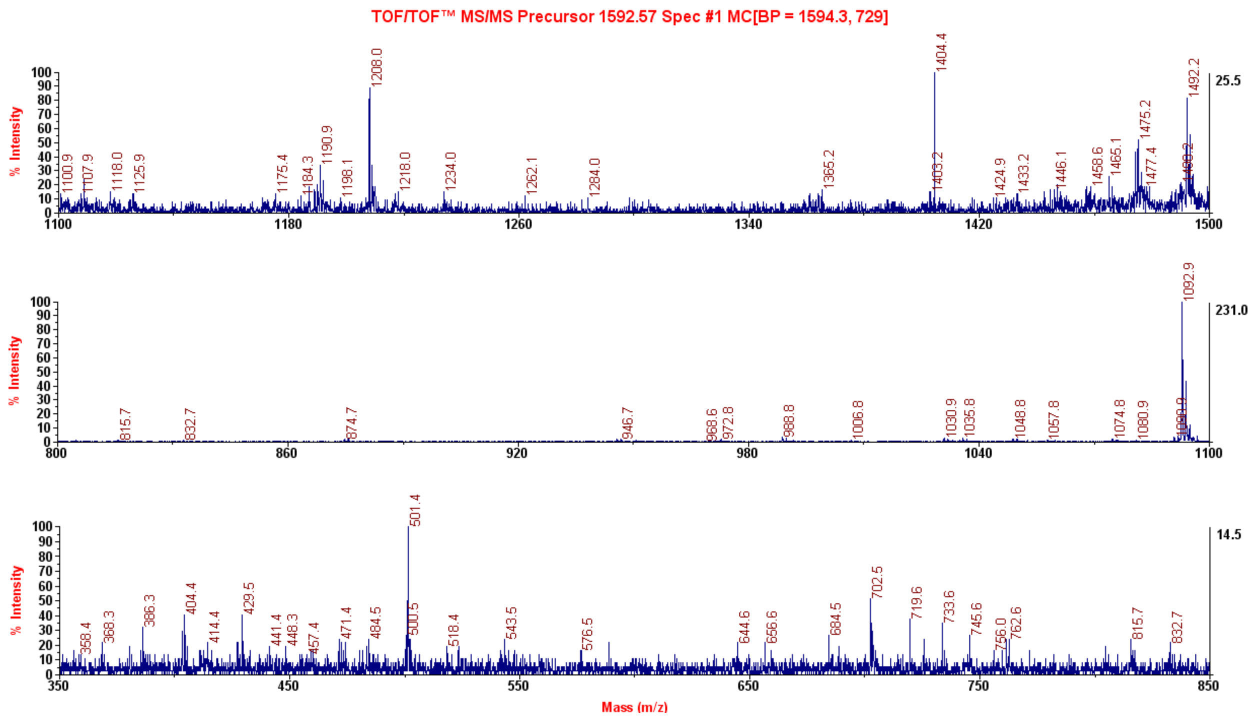

Supplementary Figure 34. MS/MS spectrum of **B2**. Mass range: *m*/*z* 350–1500.

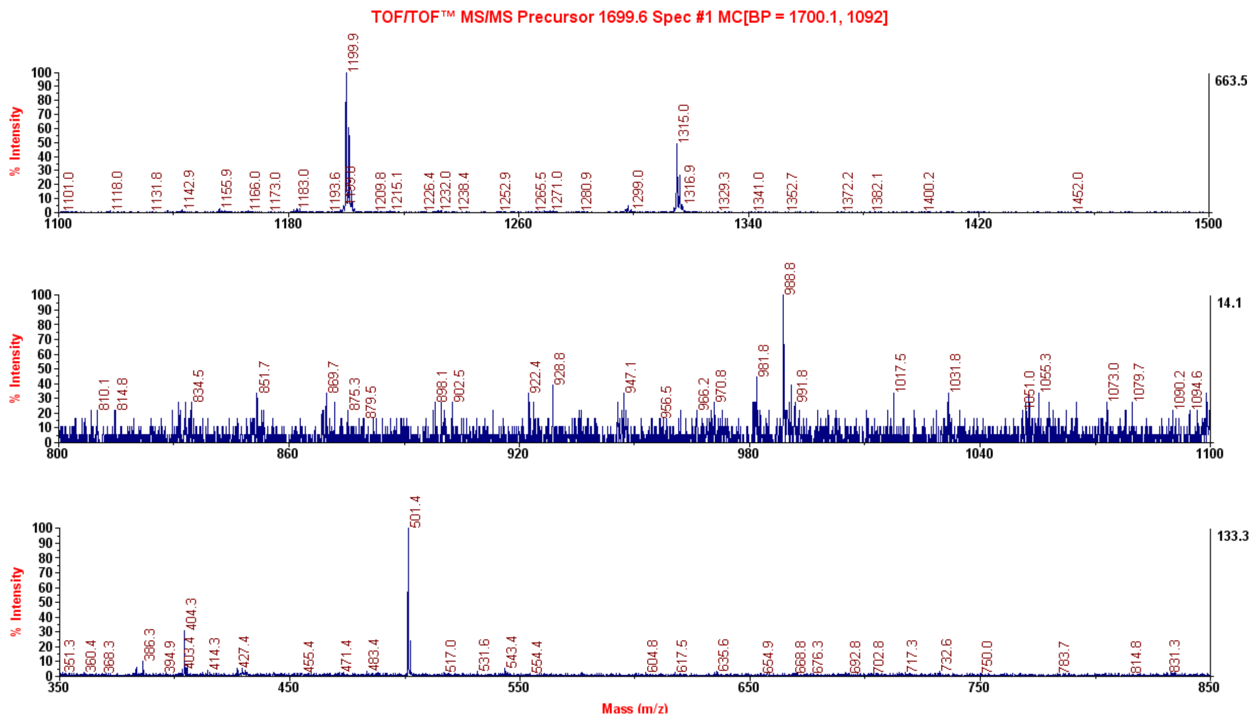

Supplementary Figure 35. MS/MS spectrum of **B3**. Mass range: *m*/*z* 350–1500.

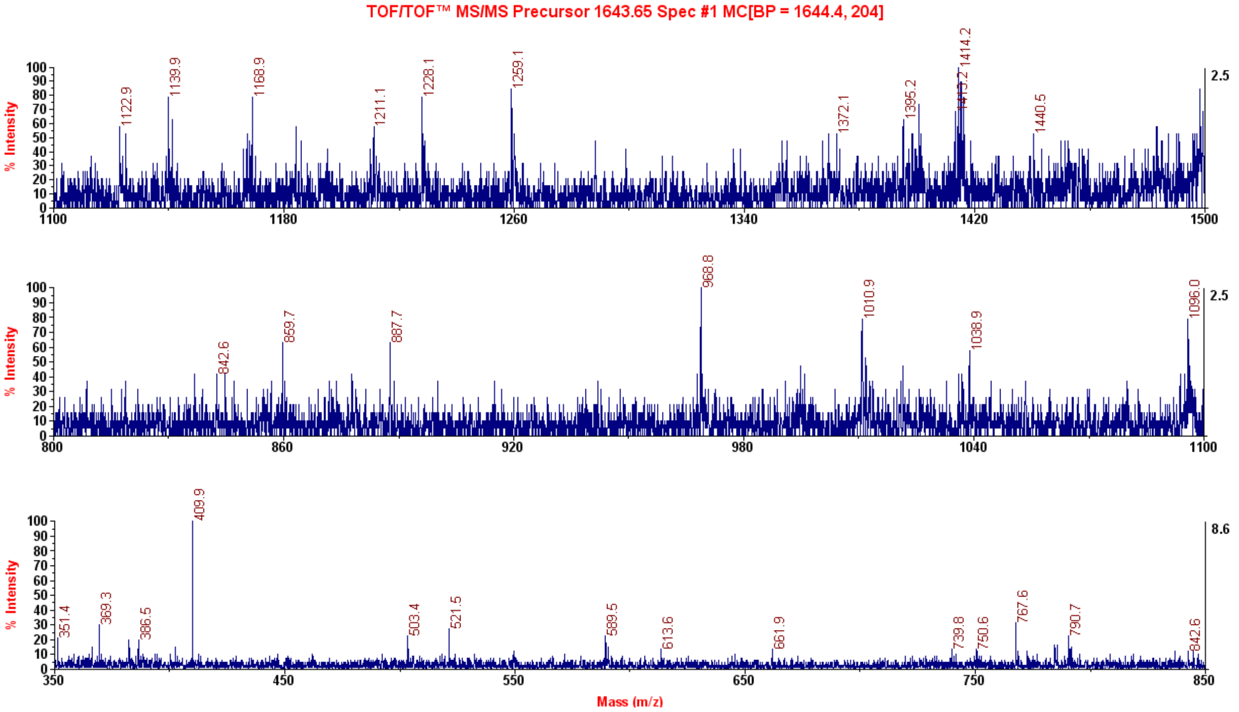

Supplementary Figure 36. MS/MS spectrum of **B4**. Mass range: *m*/*z* 350–1500.

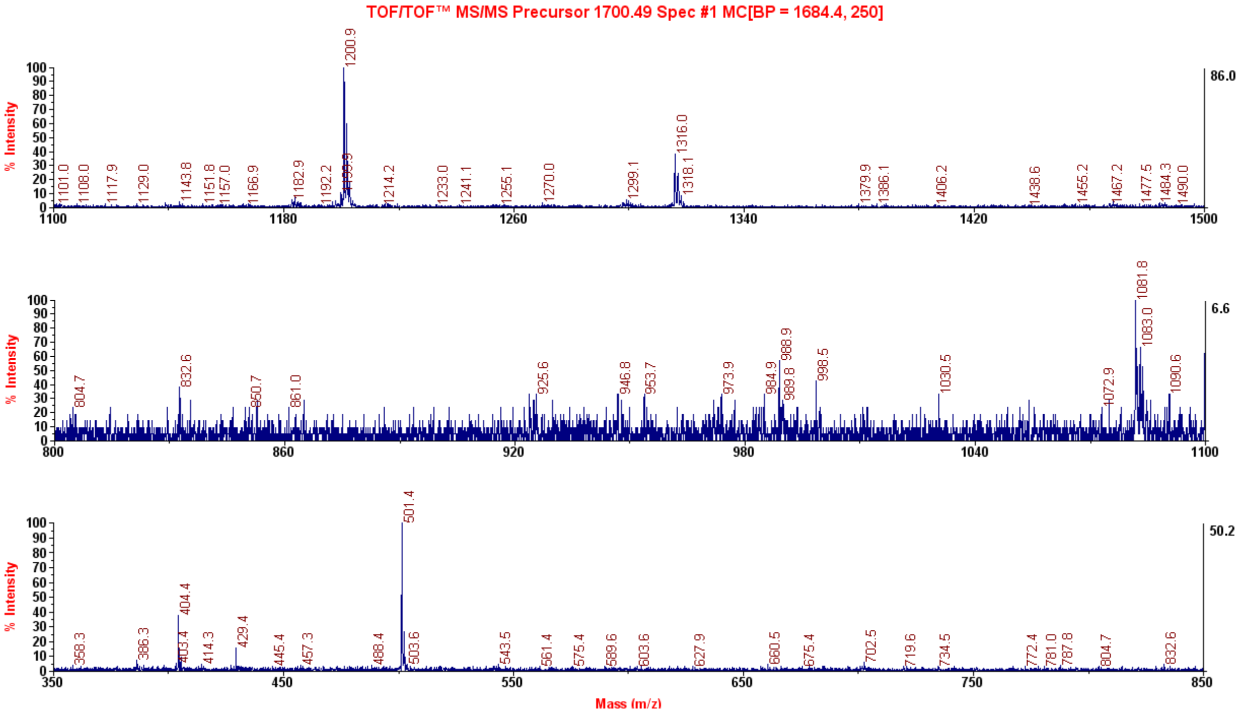

Supplementary Figure 37. MS/MS spectrum of **B5**. Mass range: *m*/*z* 350–1500.

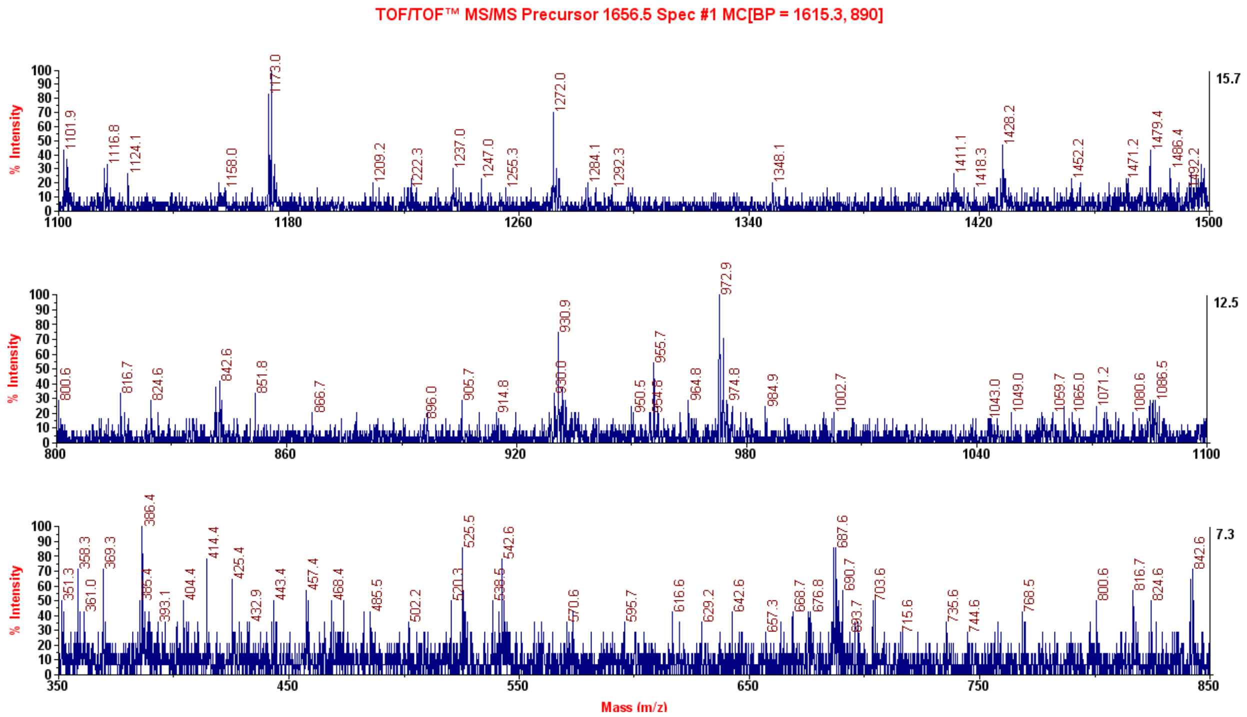

Supplementary Figure 38. MS/MS spectrum of **C1**. Mass range: *m*/*z* 350–1500.

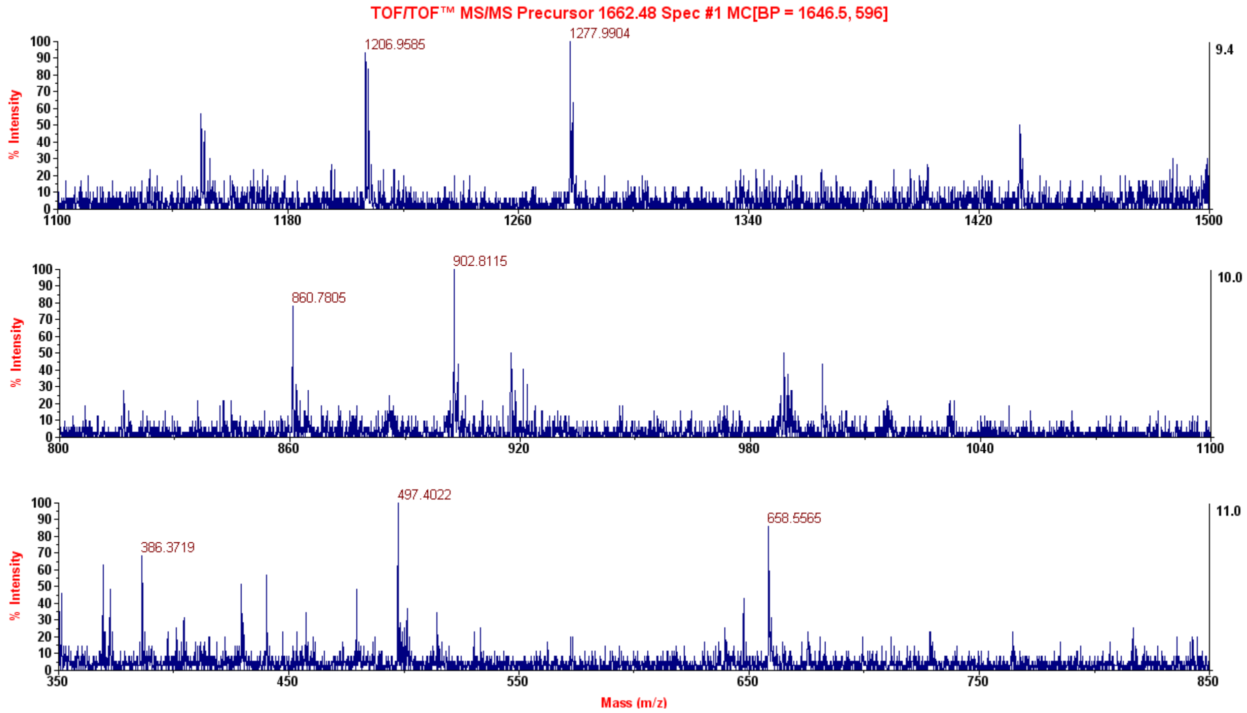

Supplementary Figure 39. MS/MS spectrum of **C2**. Mass range: *m*/*z* 350–1500.

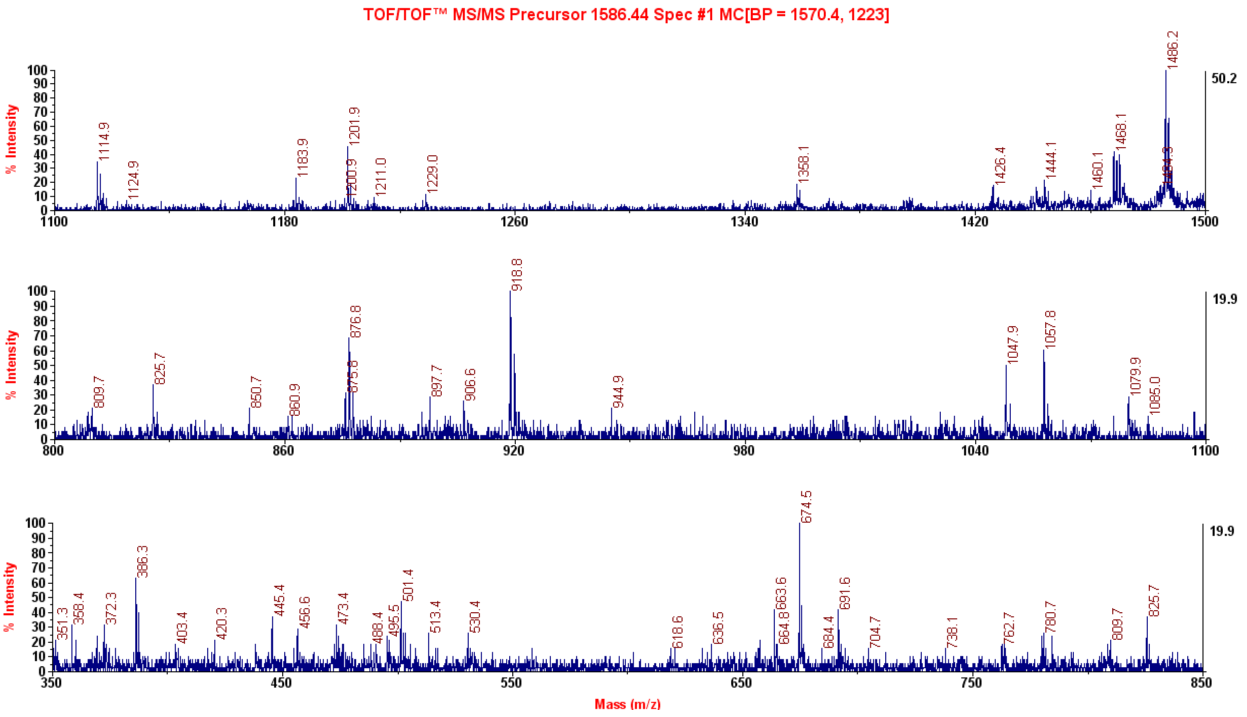

Supplementary Figure 40. MS/MS spectrum of **C3**. Mass range: *m*/*z* 350–1500.

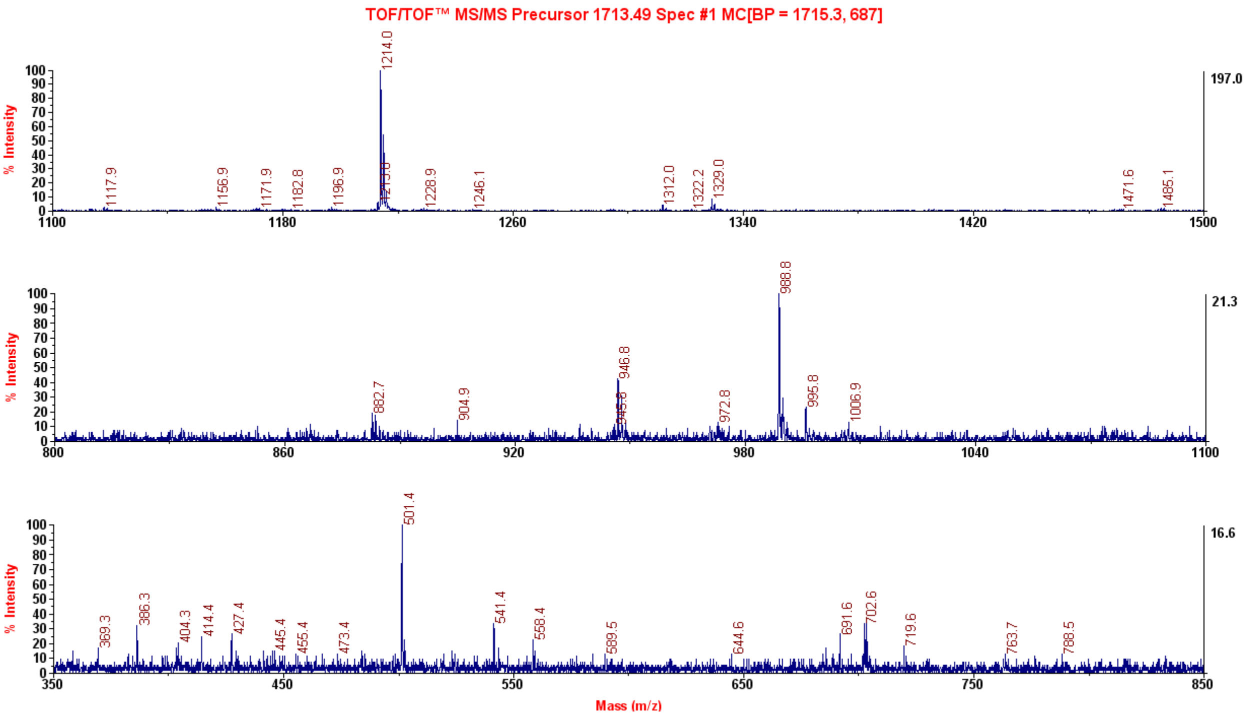

Supplementary Figure 41. MS/MS spectrum of **C4**. Mass range: *m*/*z* 350–1500.

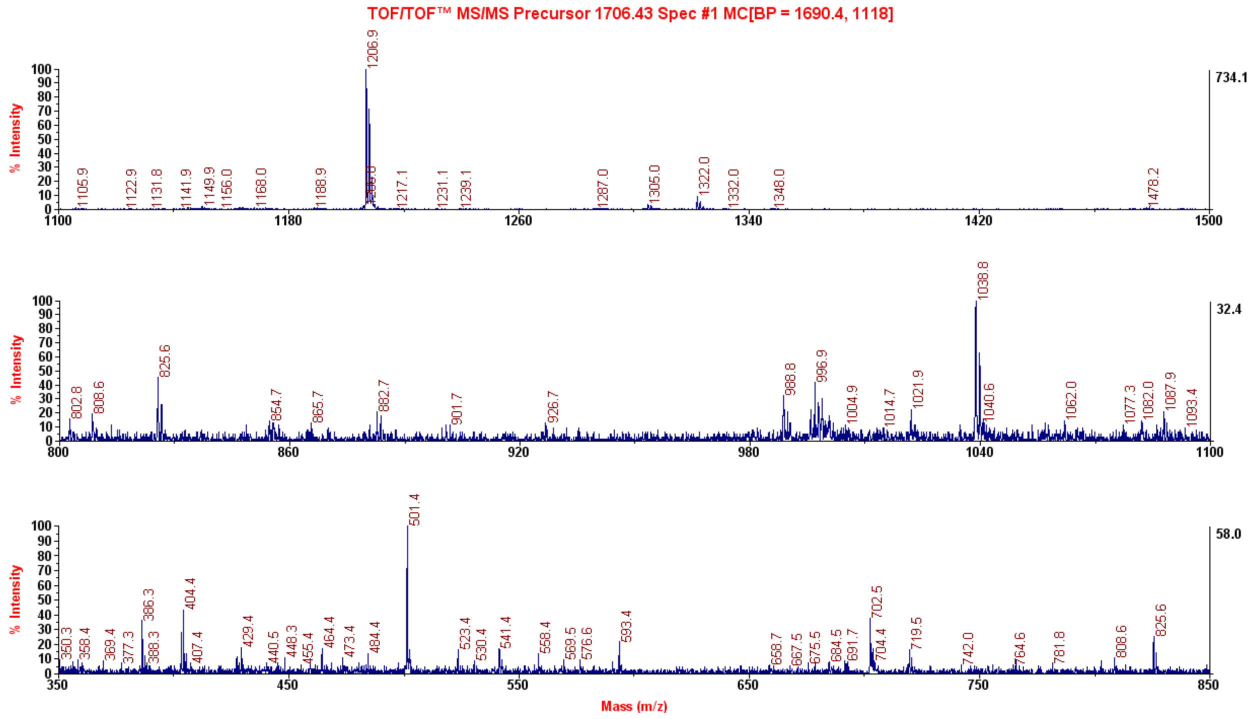

Supplementary Figure 42. MS/MS spectrum of **C5**. Mass range: *m*/*z* 350–1500.

^{1}H NMR (800 MHz, DMSO-d_6, 50 °C)
peptide A1

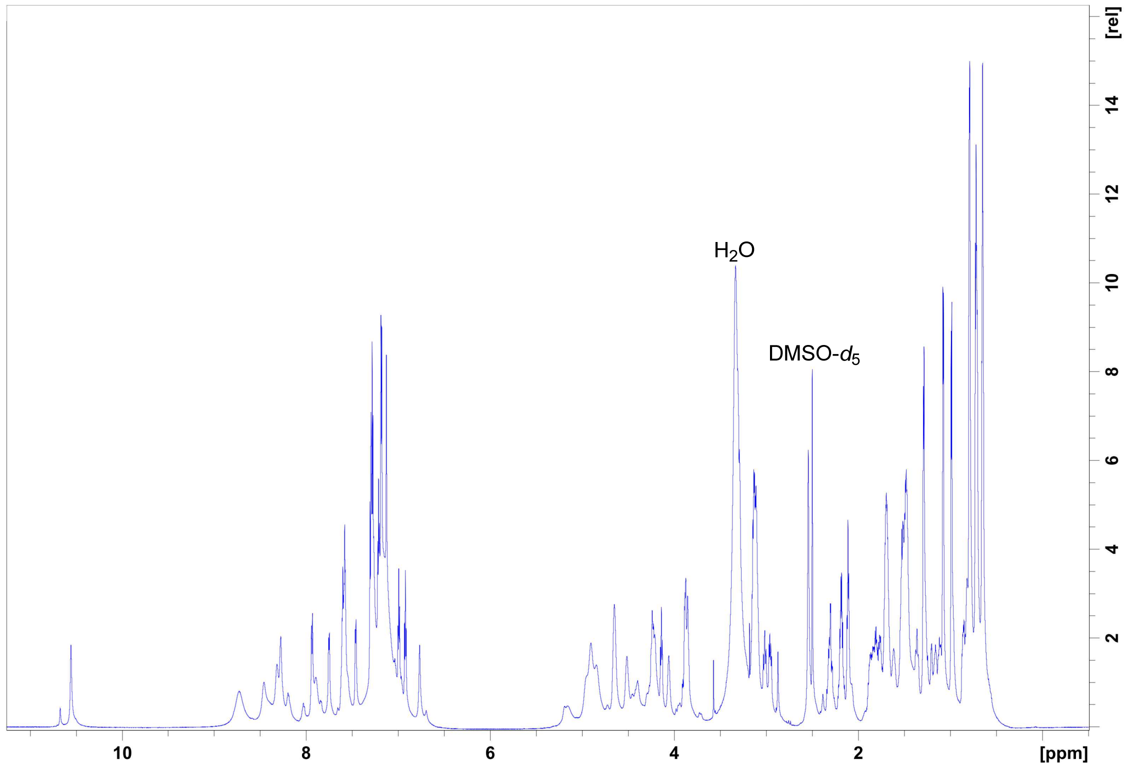

^{13}C NMR (200 MHz, DMSO-d_6, 50 °C)
peptide A1

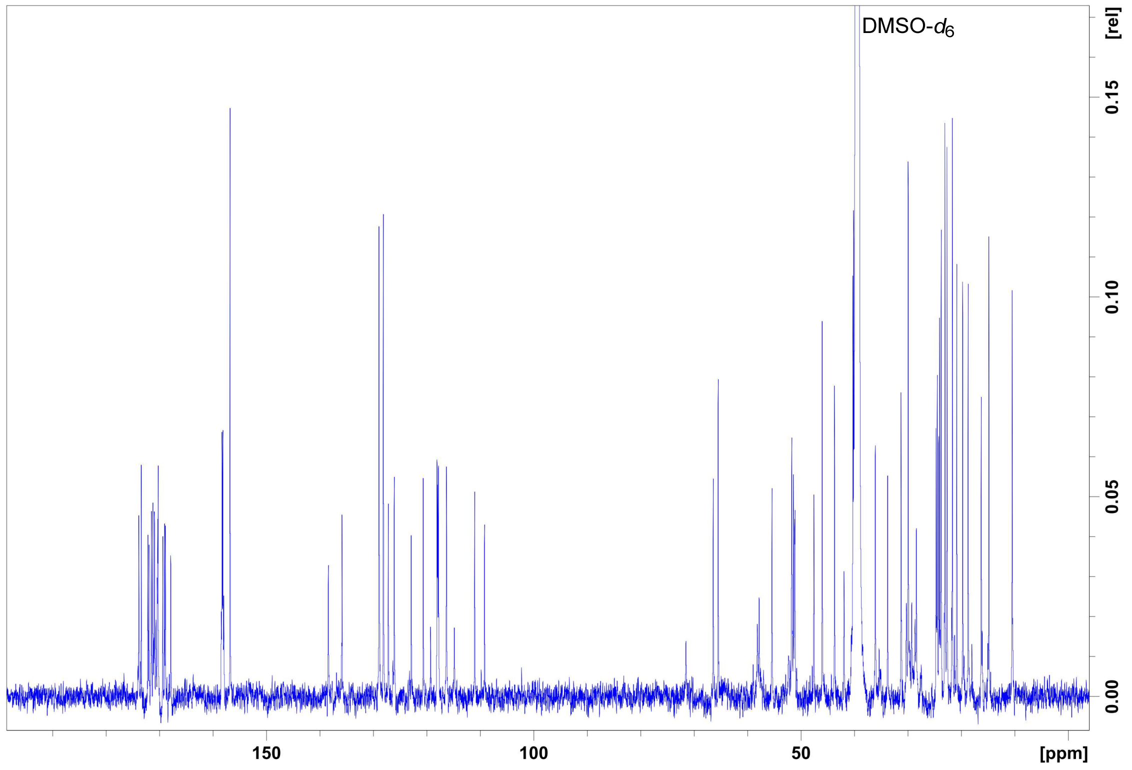

Supplementary Figure 43. ^{1}H and ^{13}C NMR spectra of **A1**. The spectra were obtained in DMSO-d_6 at 50 °C.

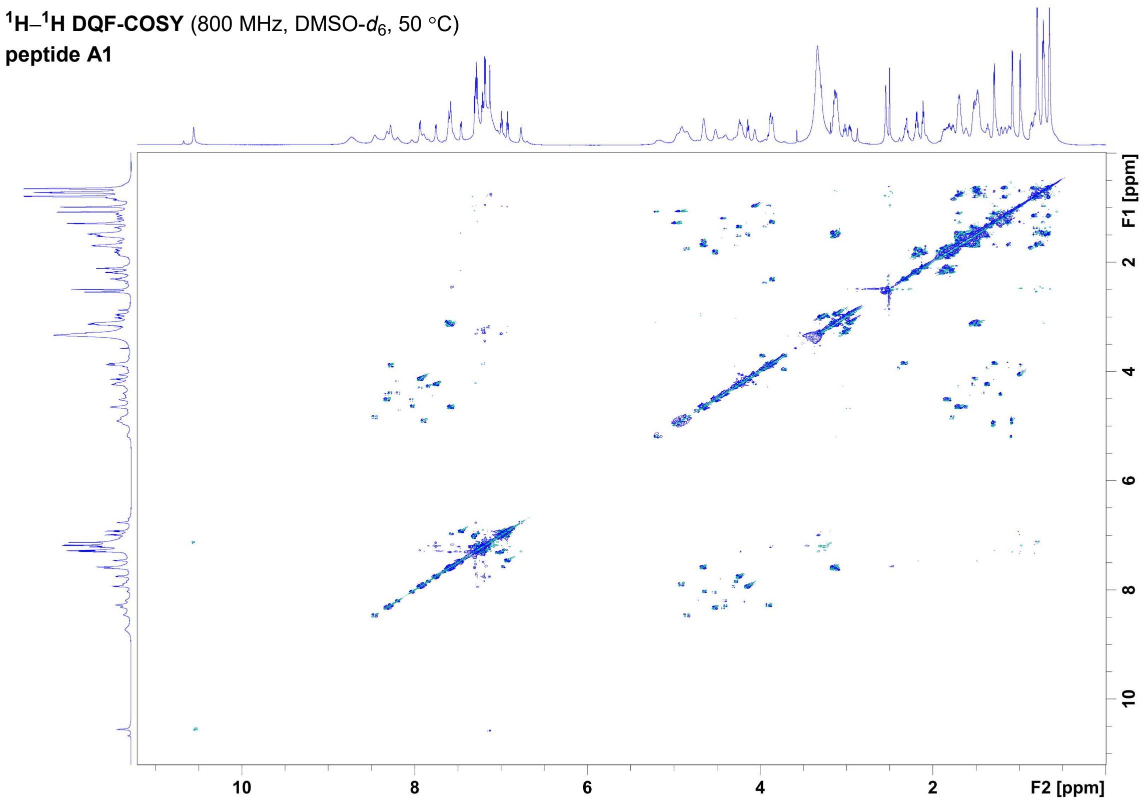

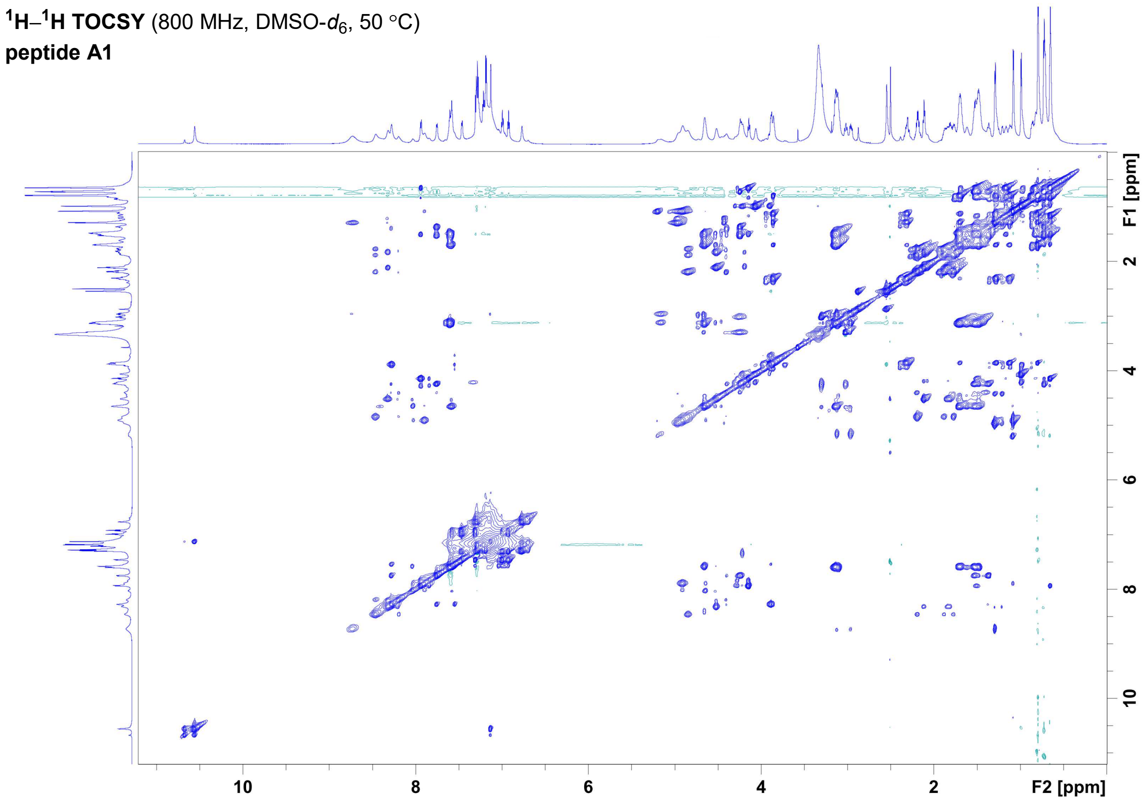

Supplementary Figure 44. ^{1}H–^{1}H DQF-COSY and ^{1}H–^{1}H TOCSY spectra of **A1**. The spectra were obtained in DMSO-d_6 at 50 °C.

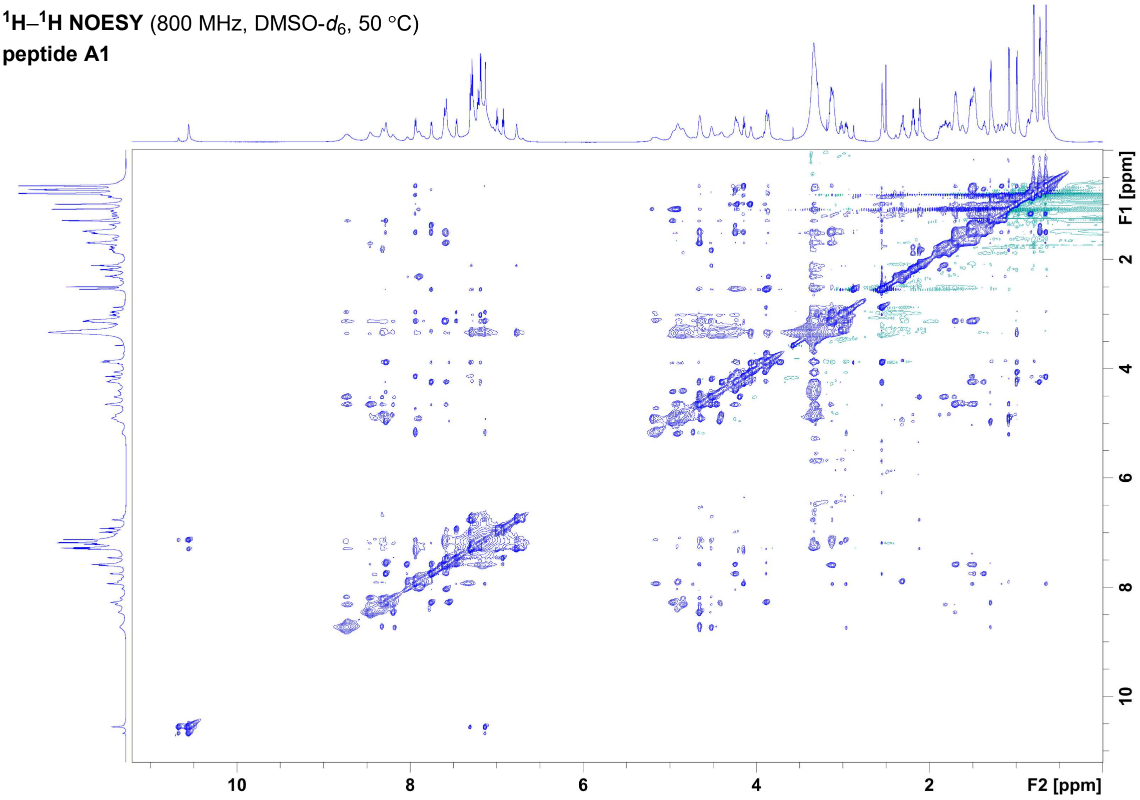

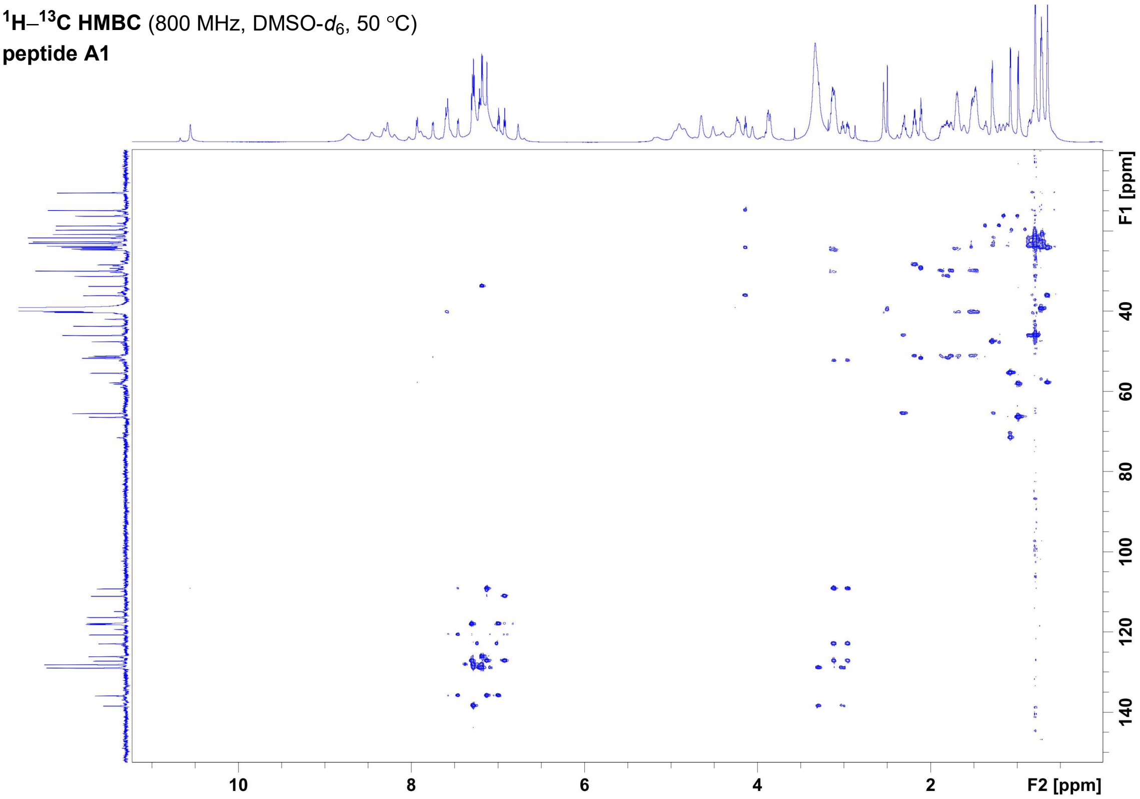

Supplementary Figure 45. ^{1}H–^{1}H NOESY and ^{1}H–^{13}C HMBC spectra of **A1**. The spectra were obtained in DMSO-d_6 at 50 °C.

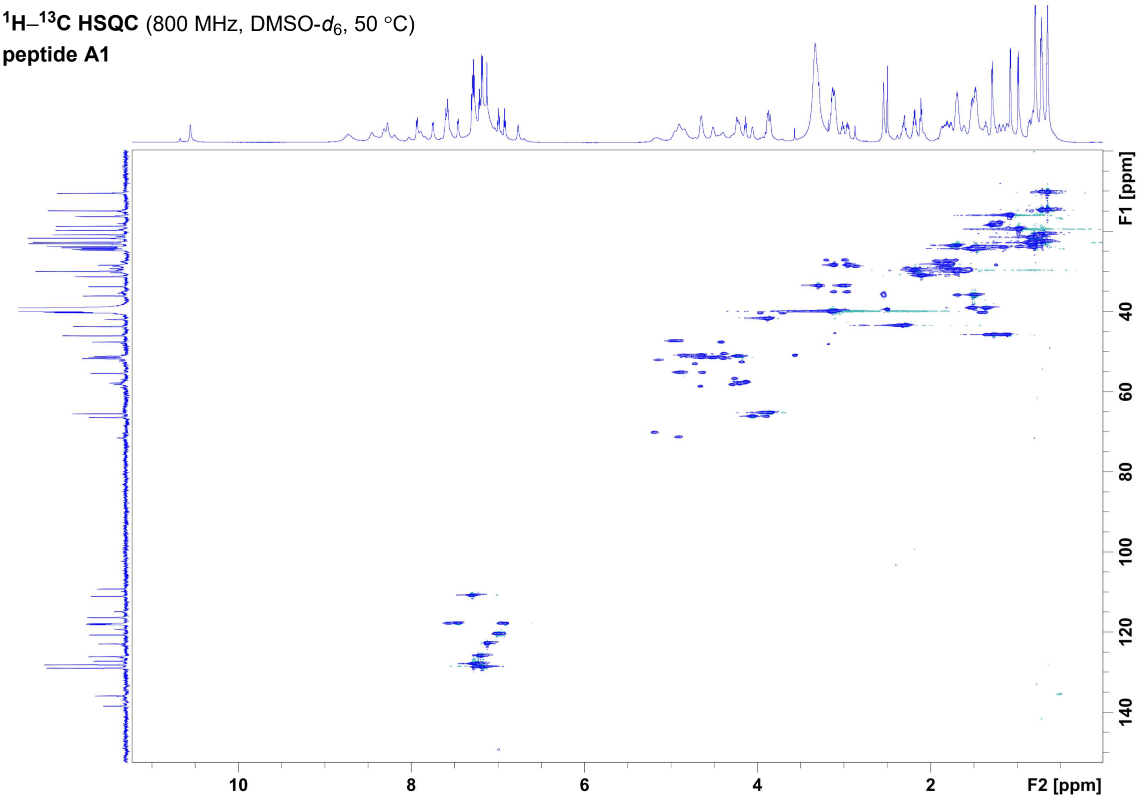

Supplementary Figure 46. ^{1}H–^{13}C HSQC spectrum of **A1**. The spectrum was obtained in DMSO-d_6 at 50 °C.

¹H NMR (800 MHz, DMSO-d_6, 50 °C)
peptide A2

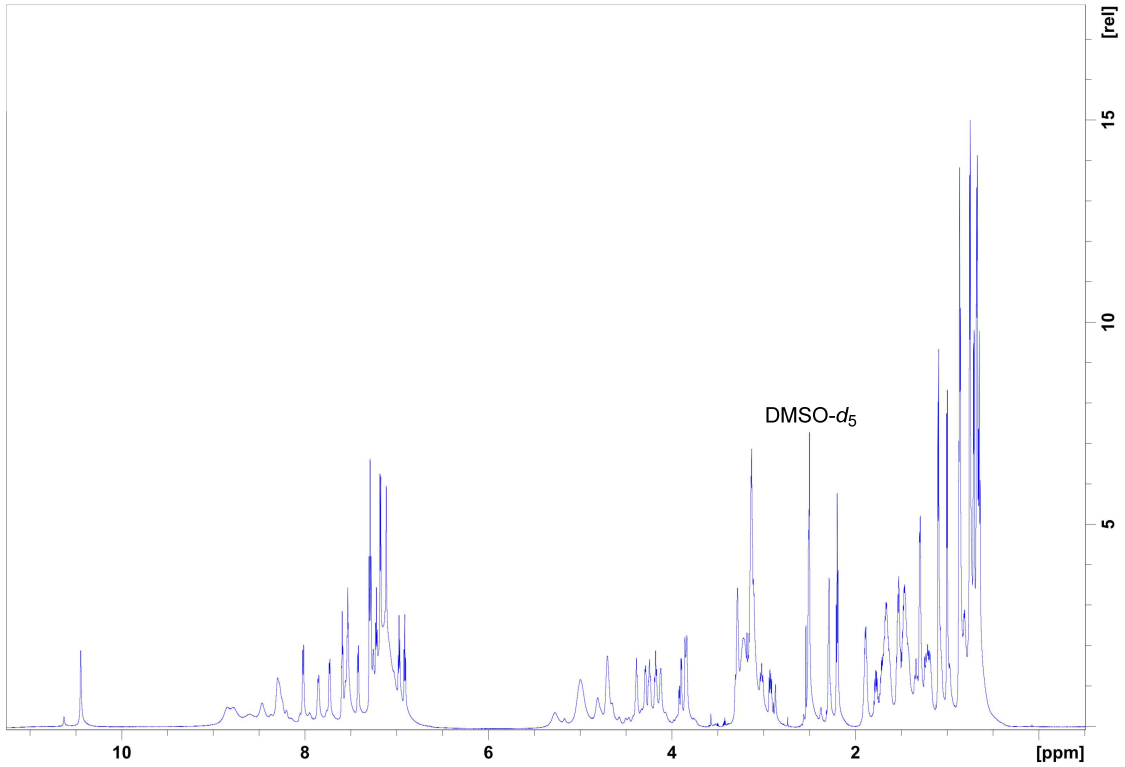

¹³C NMR (200 MHz, DMSO-d_6, 50 °C)
peptide A2

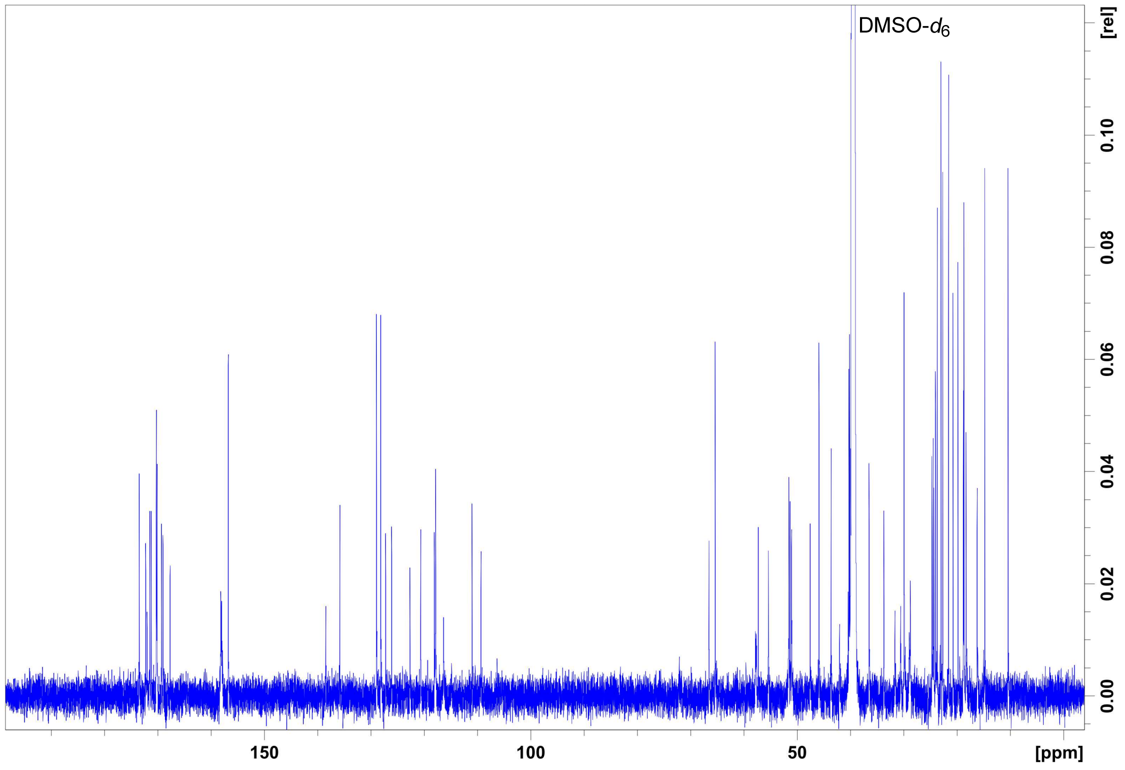

Supplementary Figure 47. ^{1}H and ^{13}C NMR spectra of **A2**. The spectra were obtained in DMSO-d_6 at 50 °C.

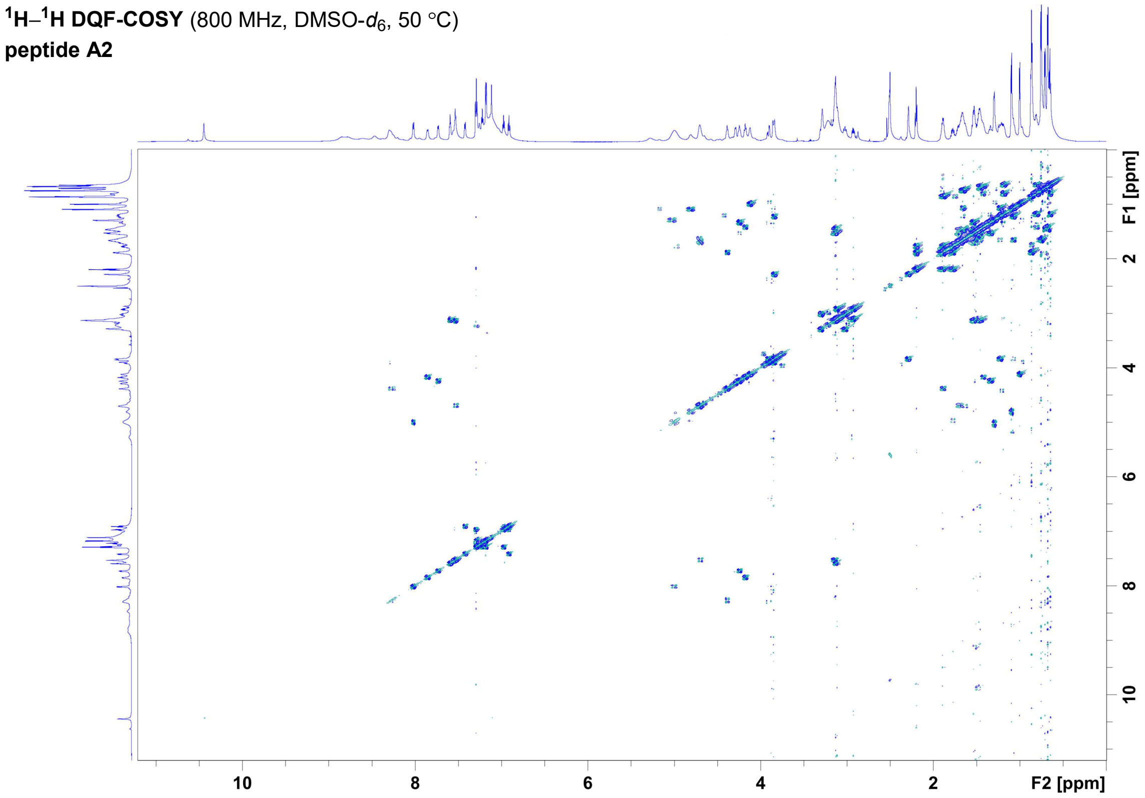

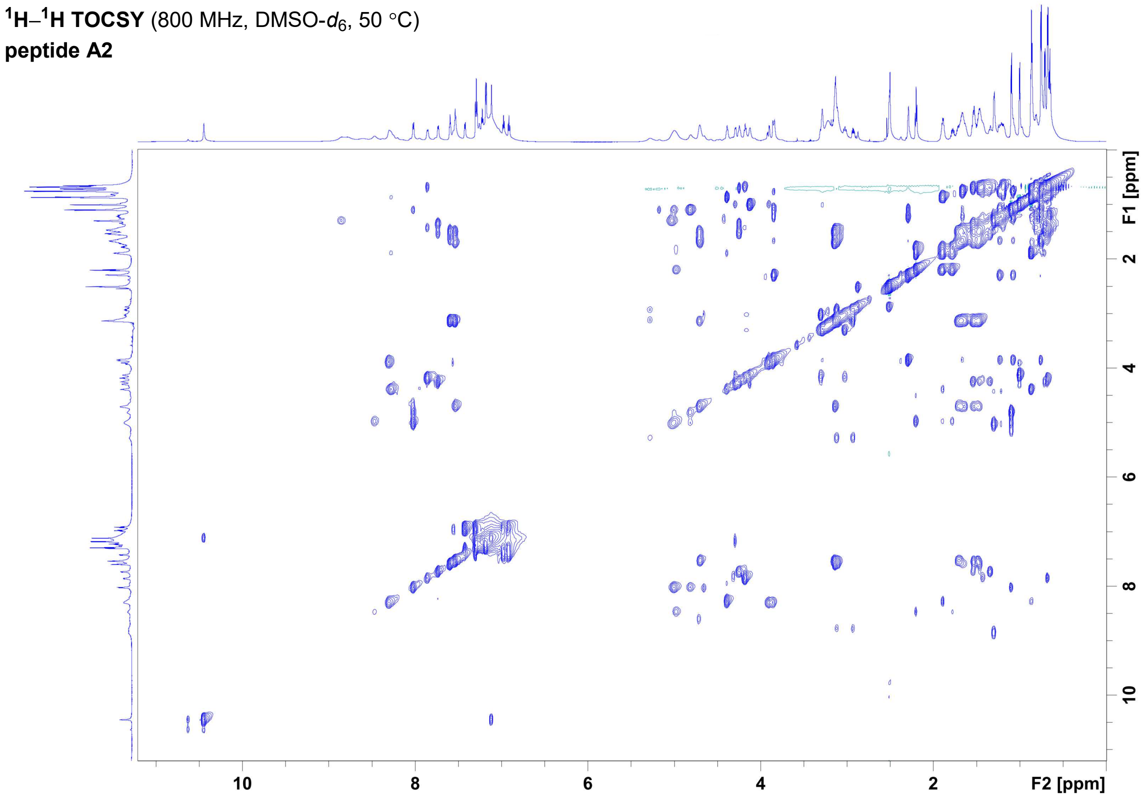

Supplementary Figure 48. ^{1}H–^{1}H DQF-COSY and ^{1}H–^{1}H TOCSY spectra of **A2**. The spectra were obtained in DMSO-d_6 at 50 °C.

^{1}H–^{1}H NOESY (800 MHz, DMSO-d_6, 50 °C)
peptide A2

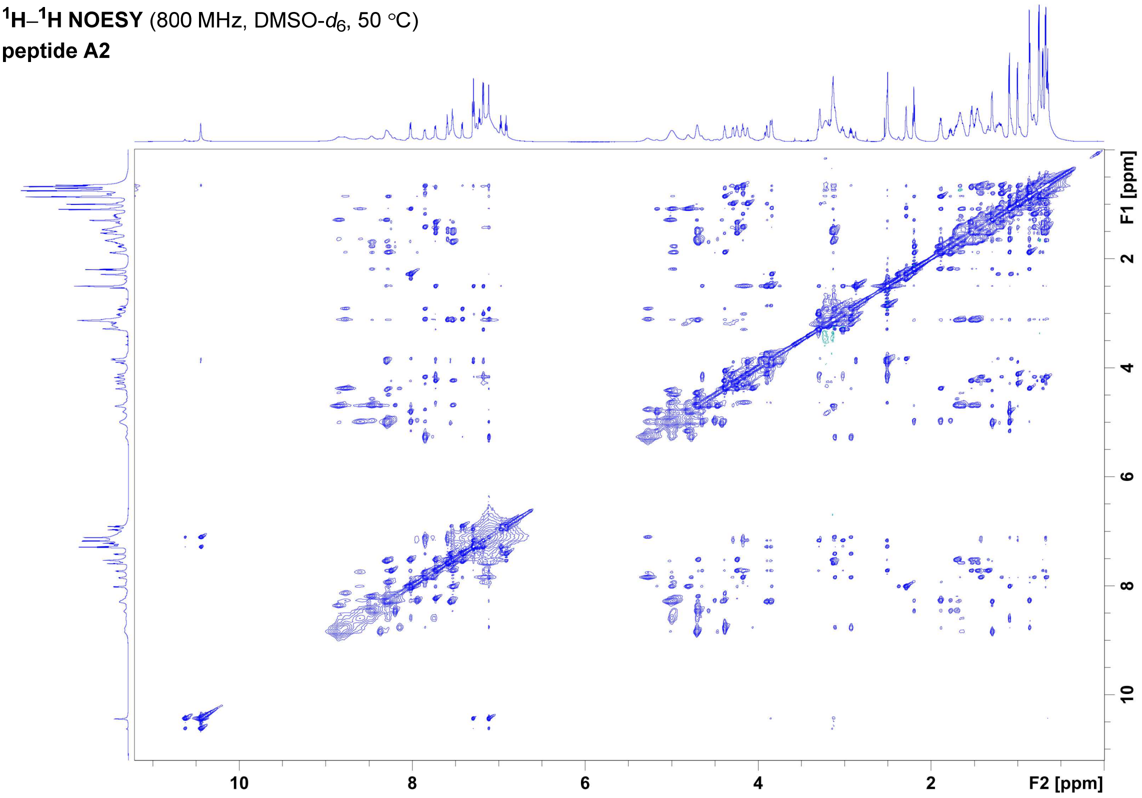

^{1}H–^{13}C HMBC (800 MHz, DMSO-d_6, 50 °C)
peptide A2

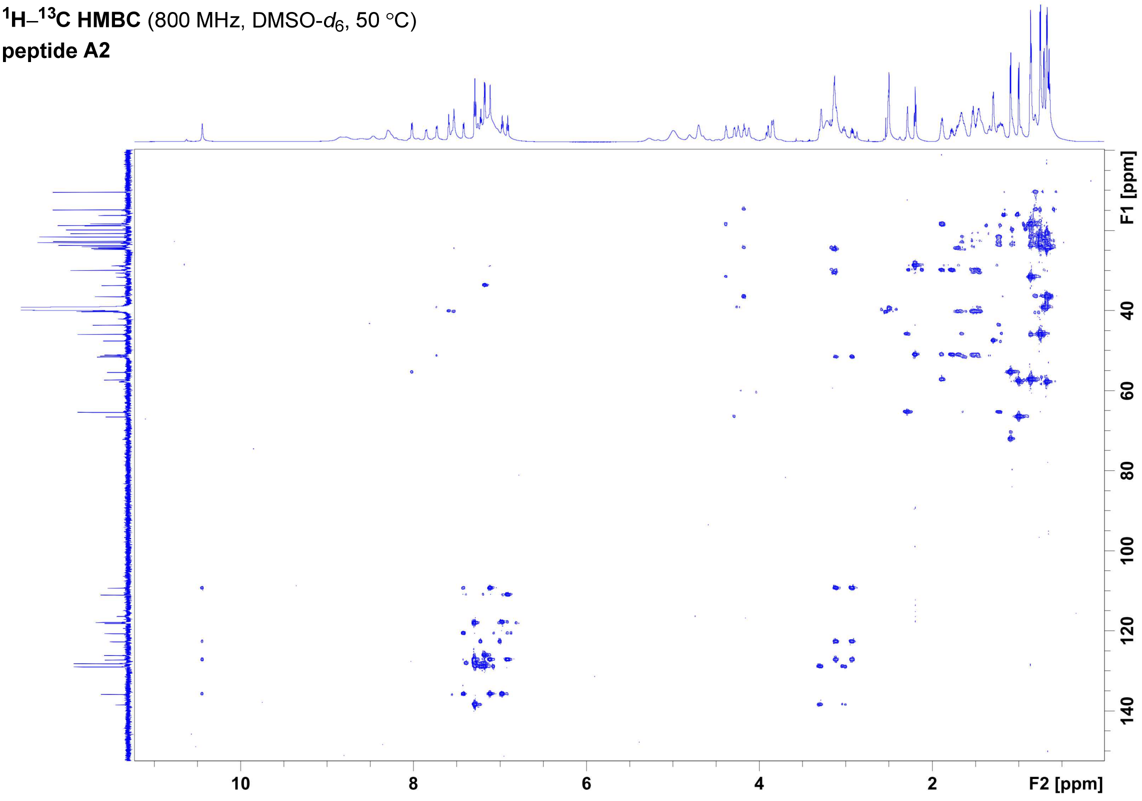

Supplementary Figure 49. ^{1}H–^{1}H NOESY and ^{1}H–^{13}C HMBC spectra of **A2**. The spectra were obtained in DMSO-d_6 at 50 °C.

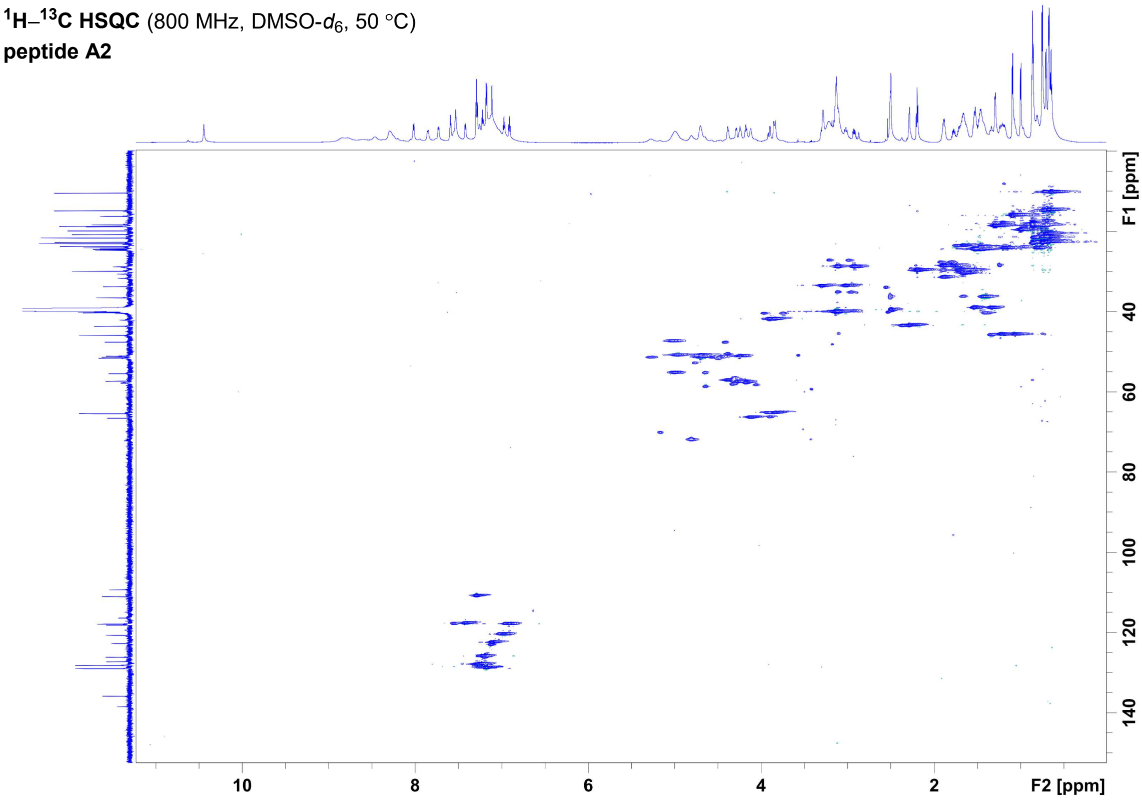

Supplementary Figure 50. ^{1}H–^{13}C HSQC spectrum of **A2**. The spectrum was obtained in DMSO-d_6 at 50 °C.

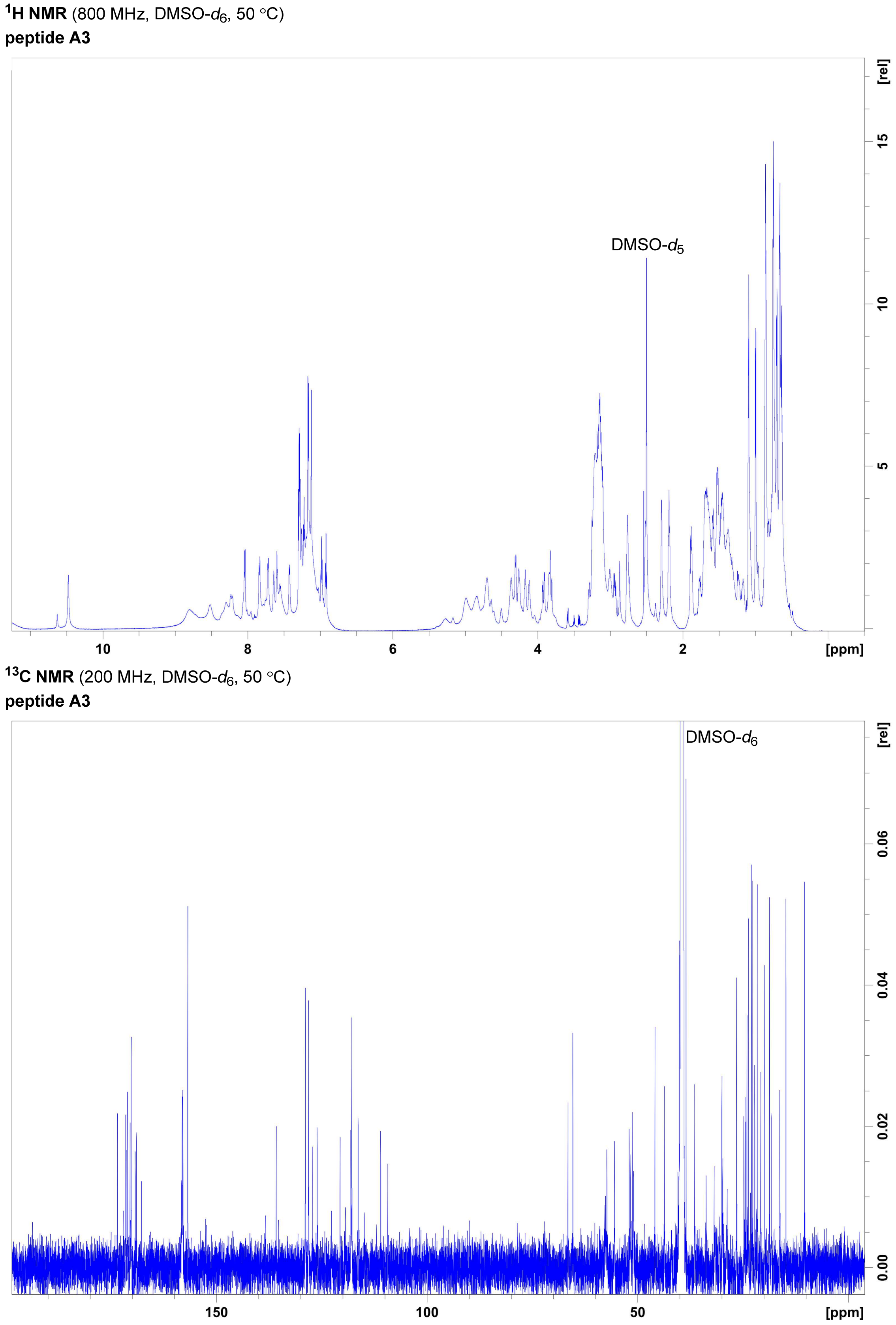

Supplementary Figure 51. ^{1}H and ^{13}C NMR spectra of **A3**. The spectra were obtained in DMSO-d_6 at 50 °C.

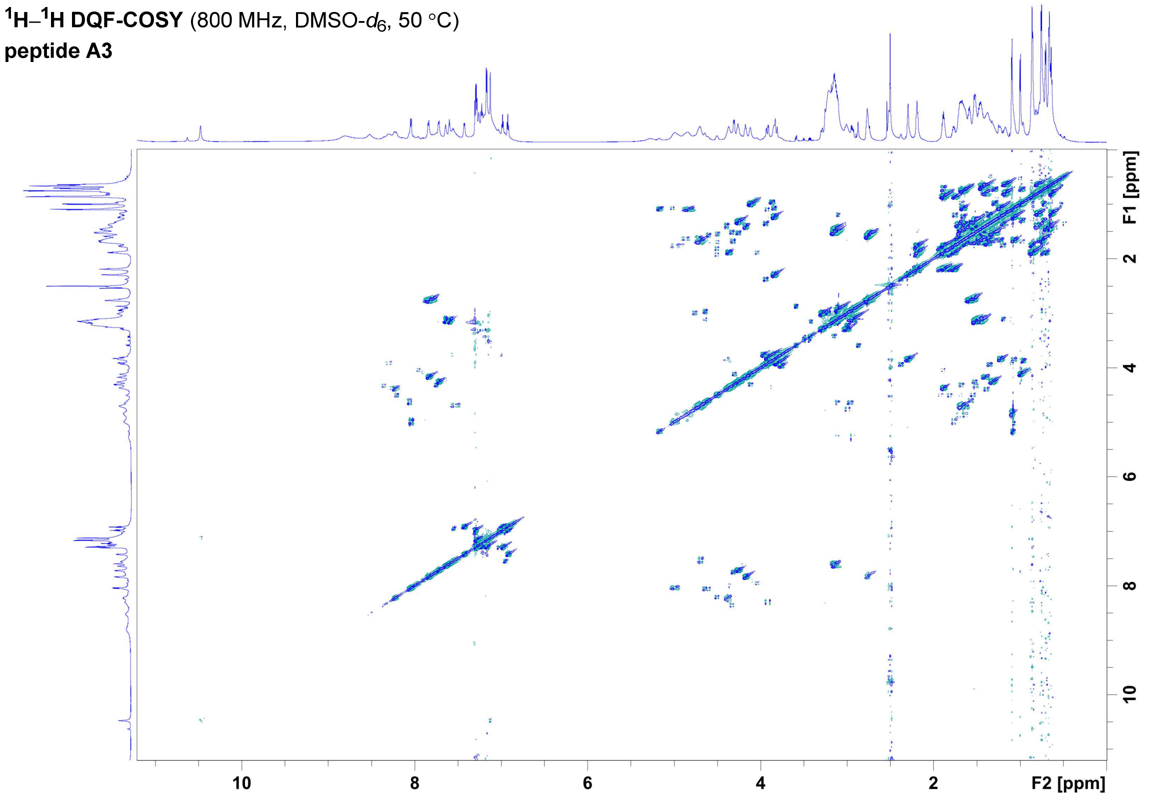

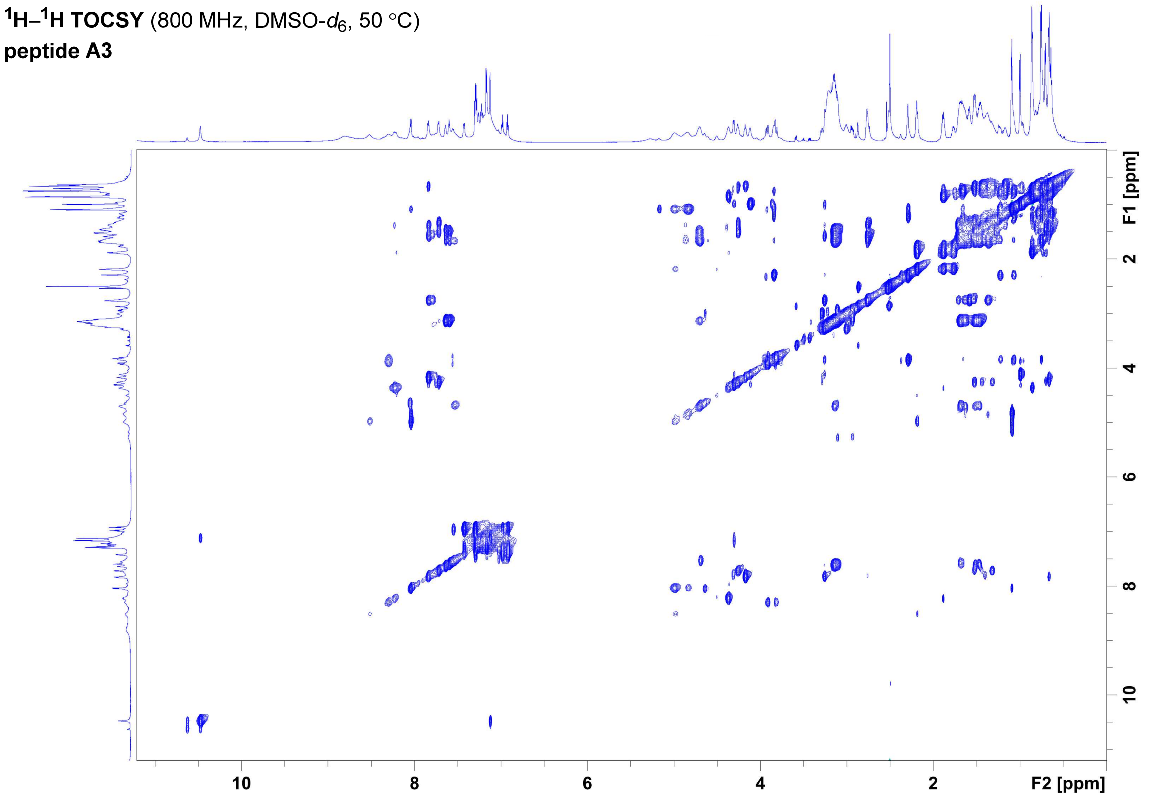

Supplementary Figure 52. ^{1}H–^{1}H DQF-COSY and ^{1}H–^{1}H TOCSY spectra of **A3**. The spectra were obtained in DMSO-d_6 at 50 °C.

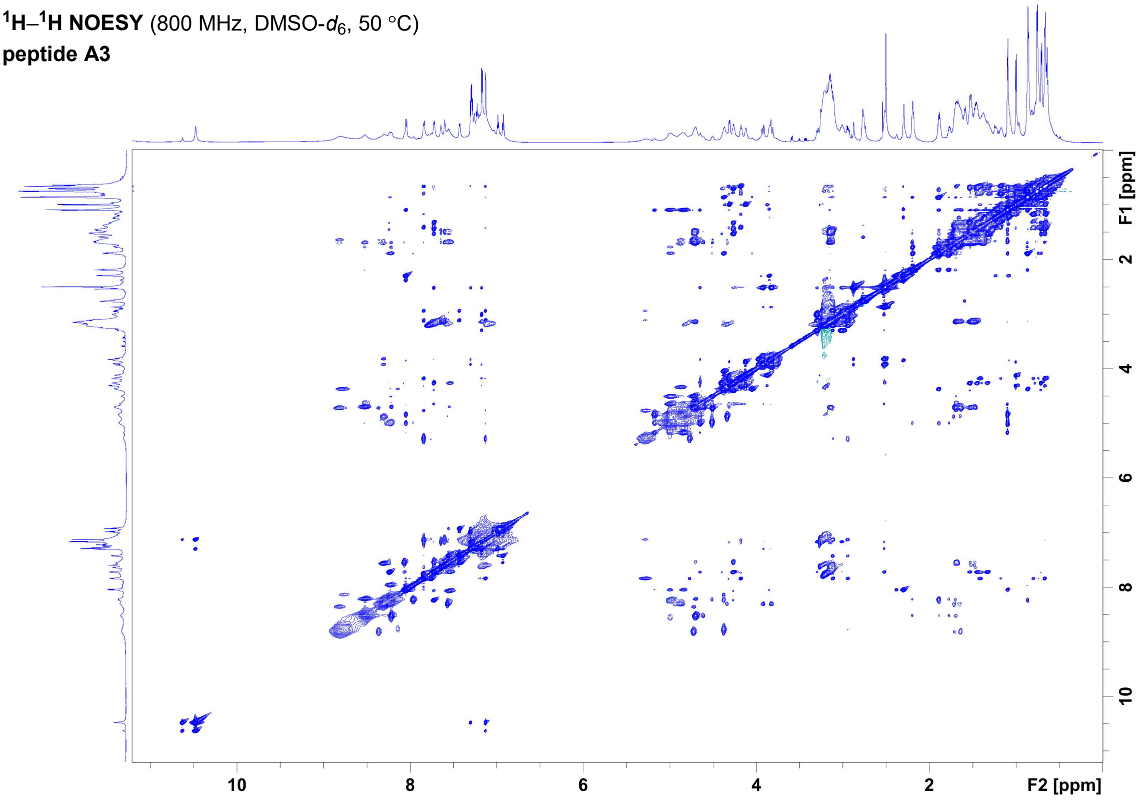

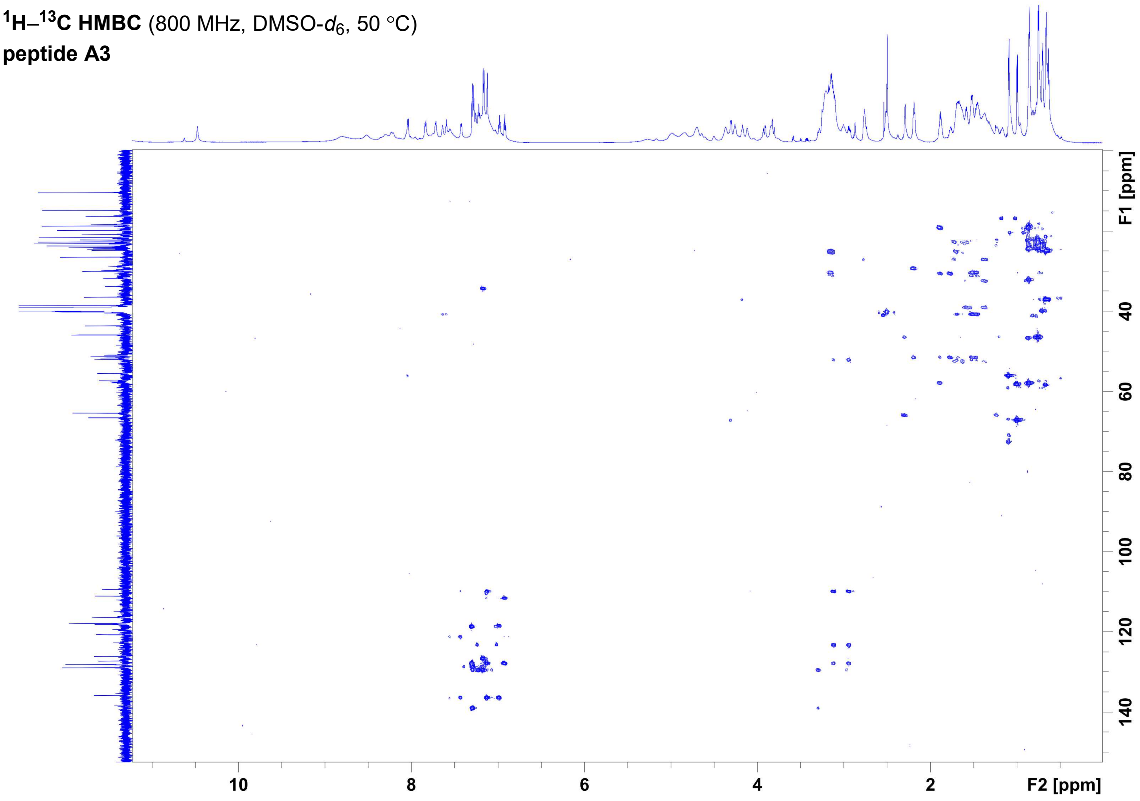

Supplementary Figure 53. ^{1}H–^{1}H NOESY and ^{1}H–^{13}C HMBC spectra of **A3**. The spectra were obtained in DMSO-d_6 at 50 °C.

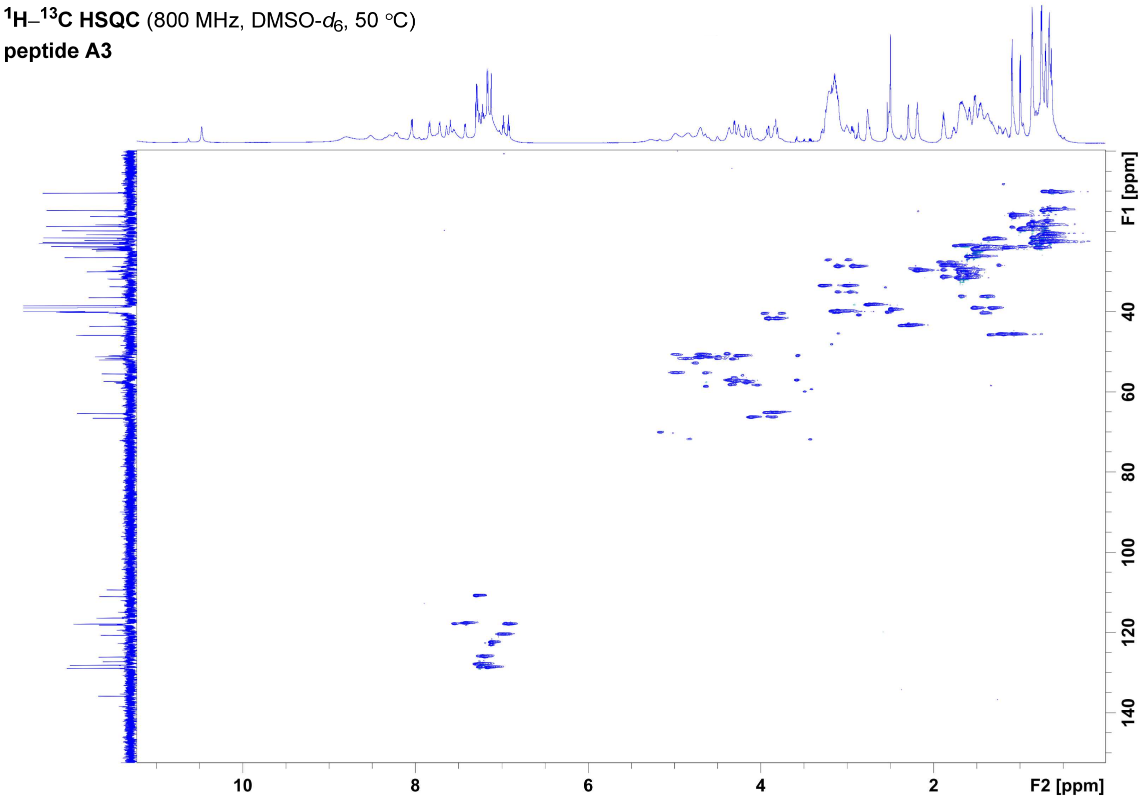

Supplementary Figure 54. ^{1}H–^{13}C HSQC spectrum of **A3**. The spectrum was obtained in DMSO-d_6 at 50 °C.

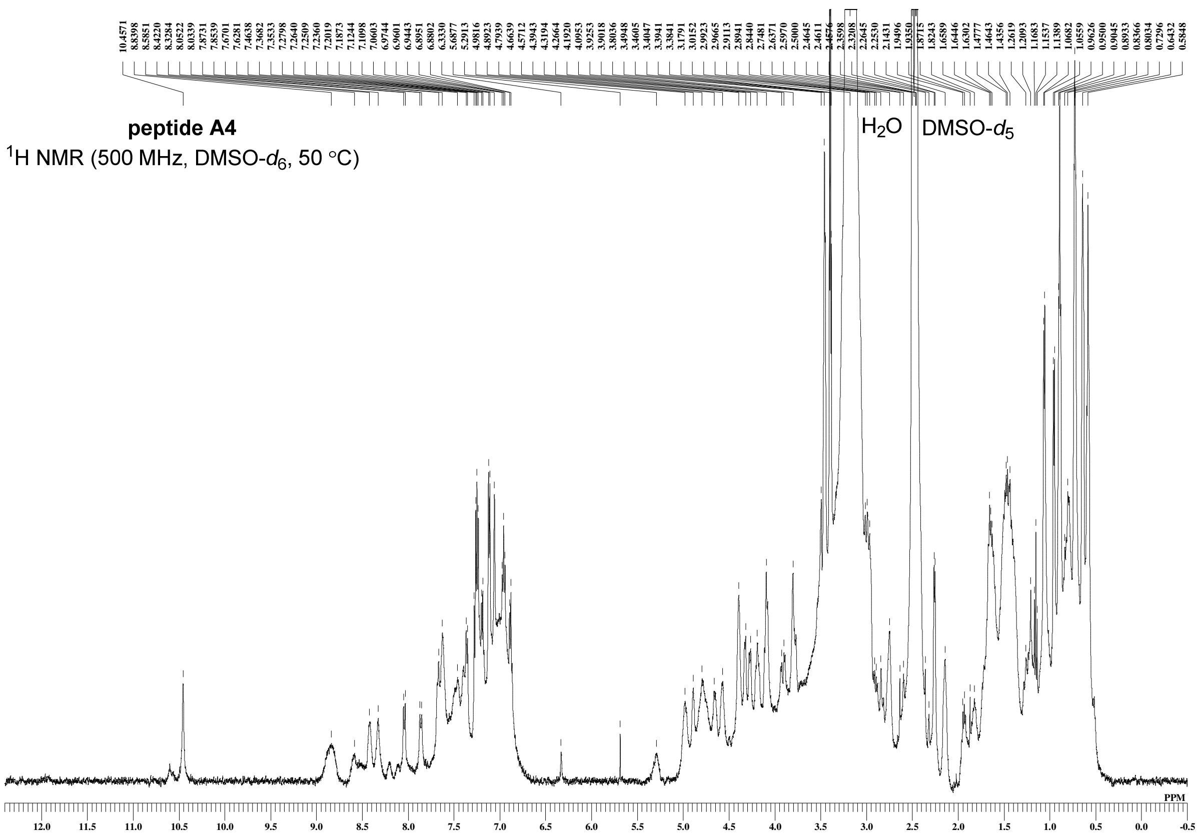

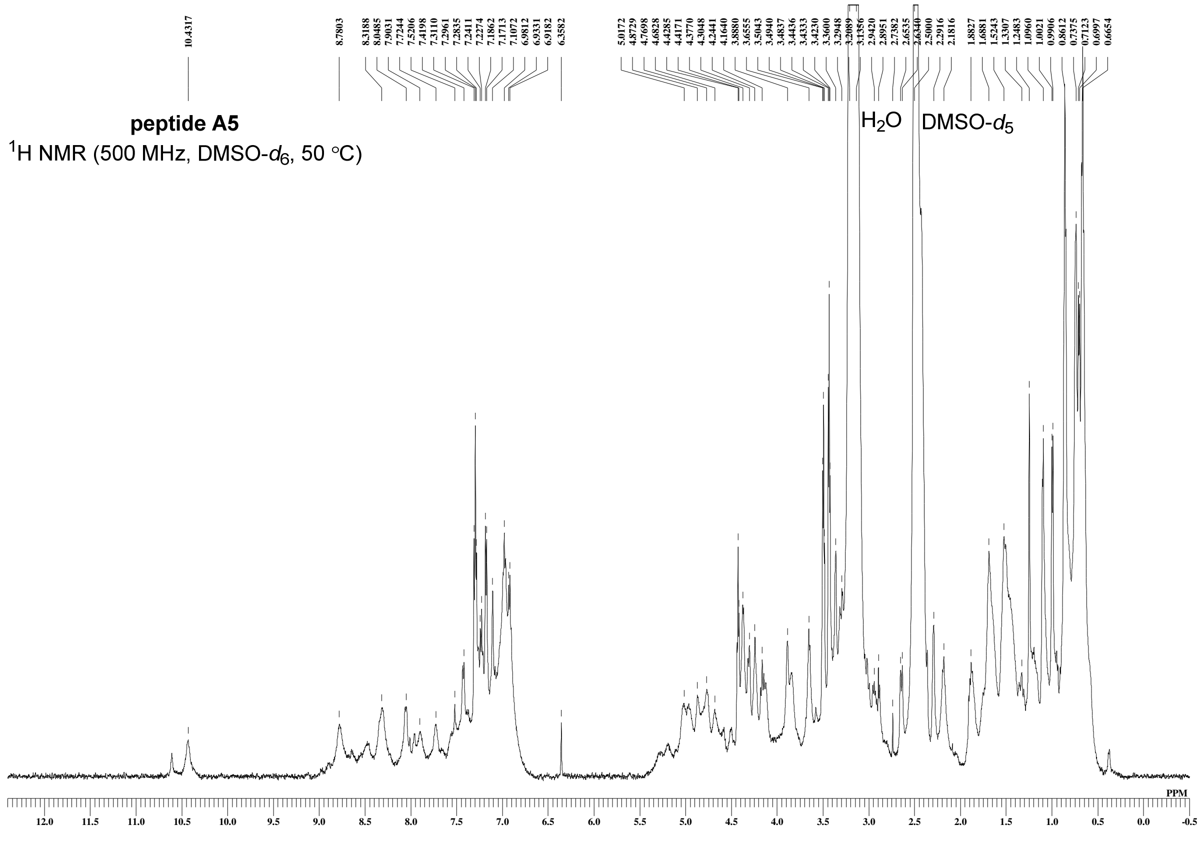

Supplementary Figure 55. ^{1}H NMR spectra of **A4** and **A5**. The spectra were obtained in DMSO-d_6 at 50 °C.

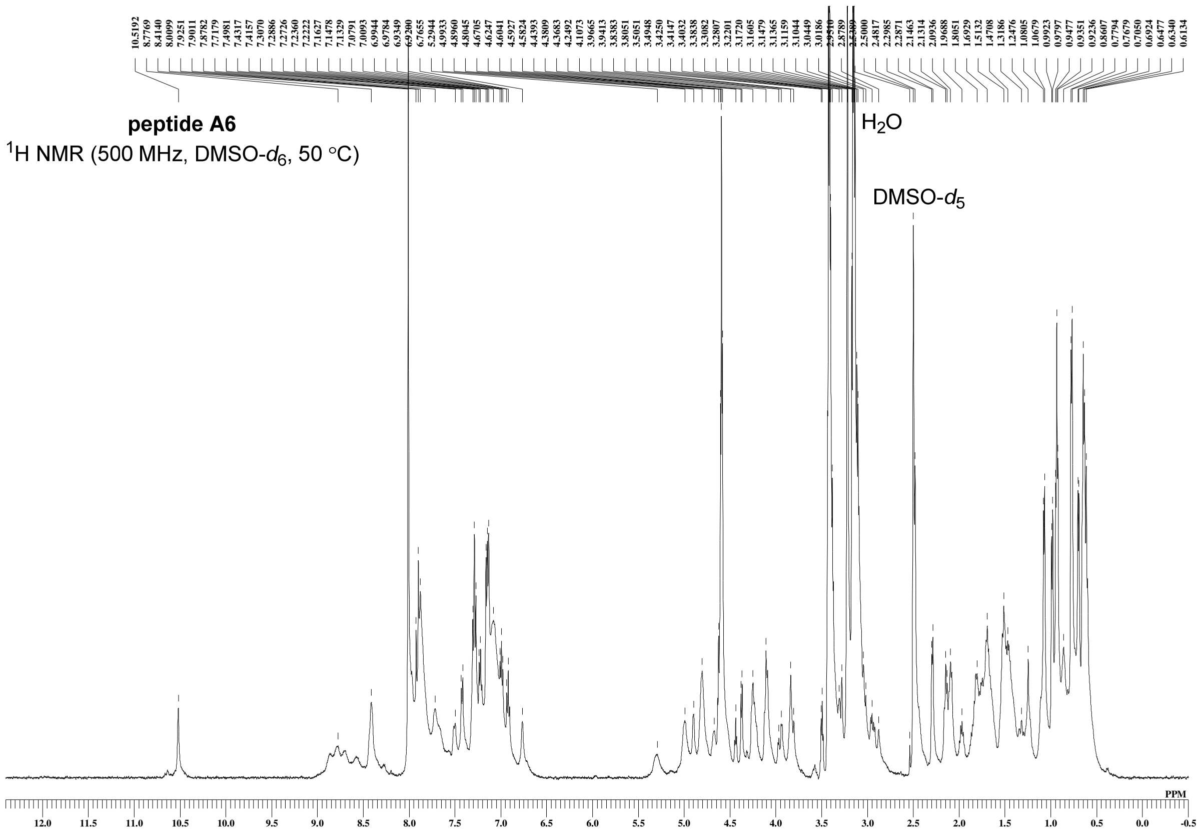

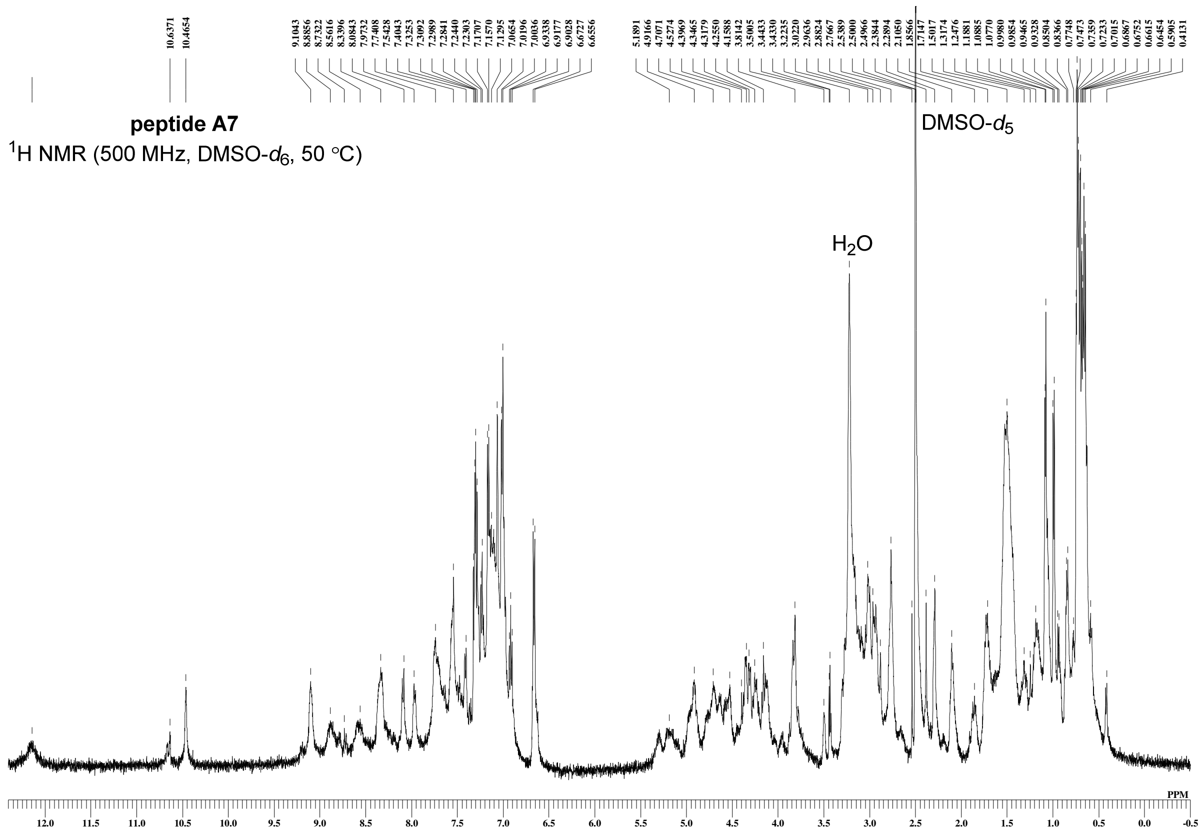

Supplementary Figure 56. ^{1}H NMR spectra of **A6** and **A7**. The spectra were obtained in DMSO-d_6 at 50 °C.

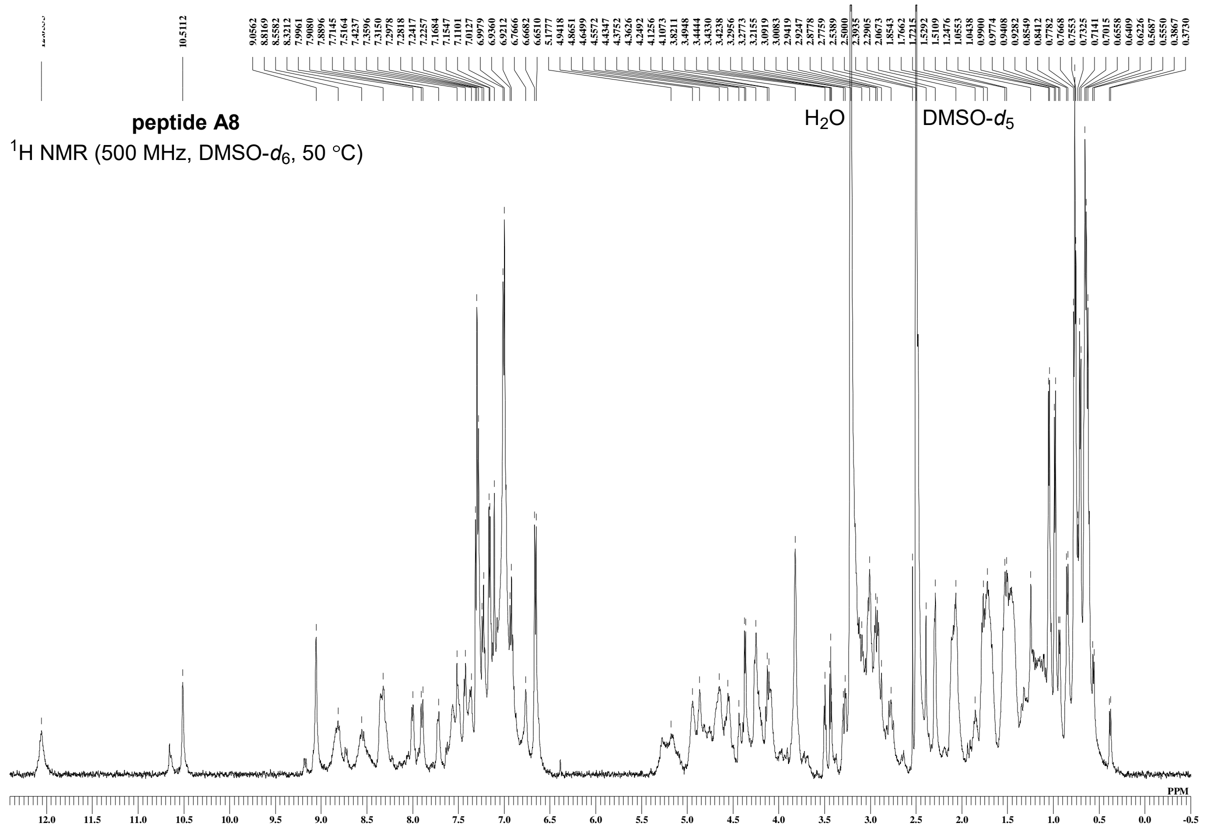

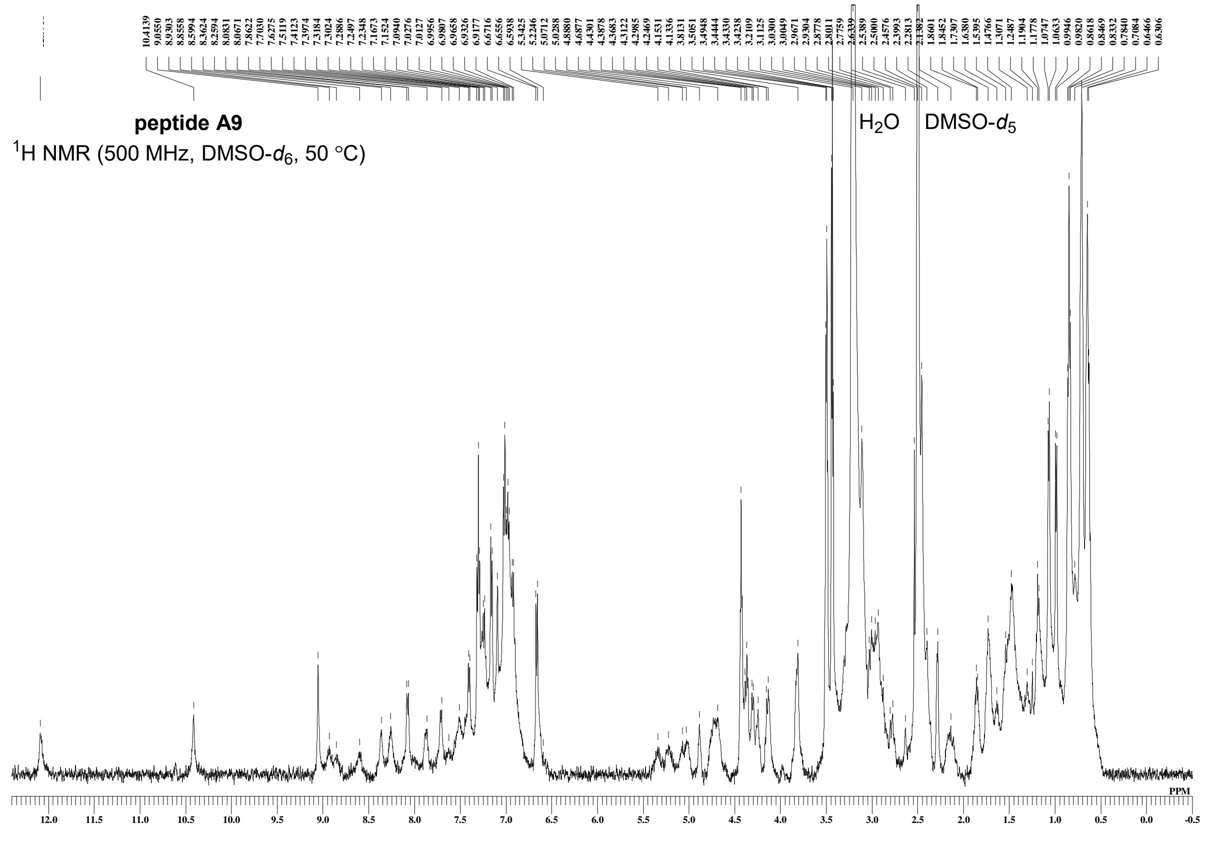

Supplementary Figure 57. ^{1}H NMR spectra of **A8** and **A9**. The spectra were obtained in DMSO-d_6 at 50 °C.

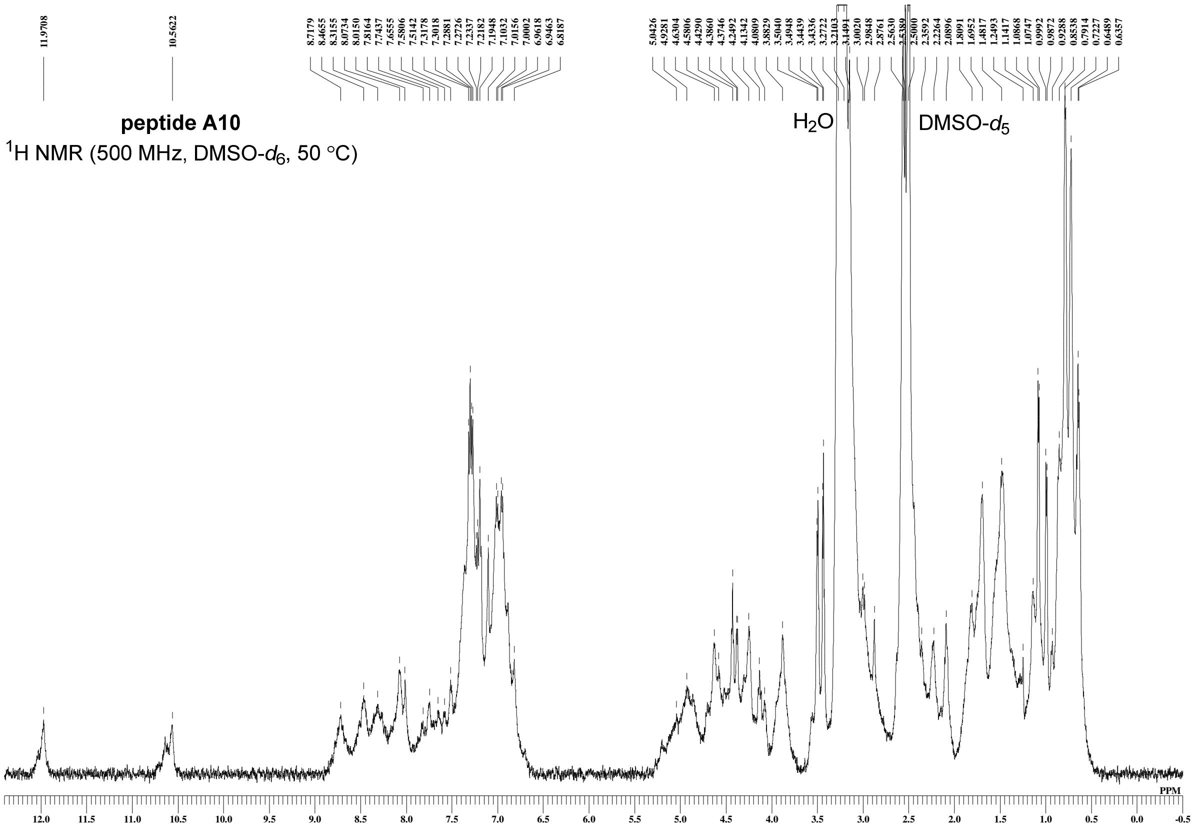

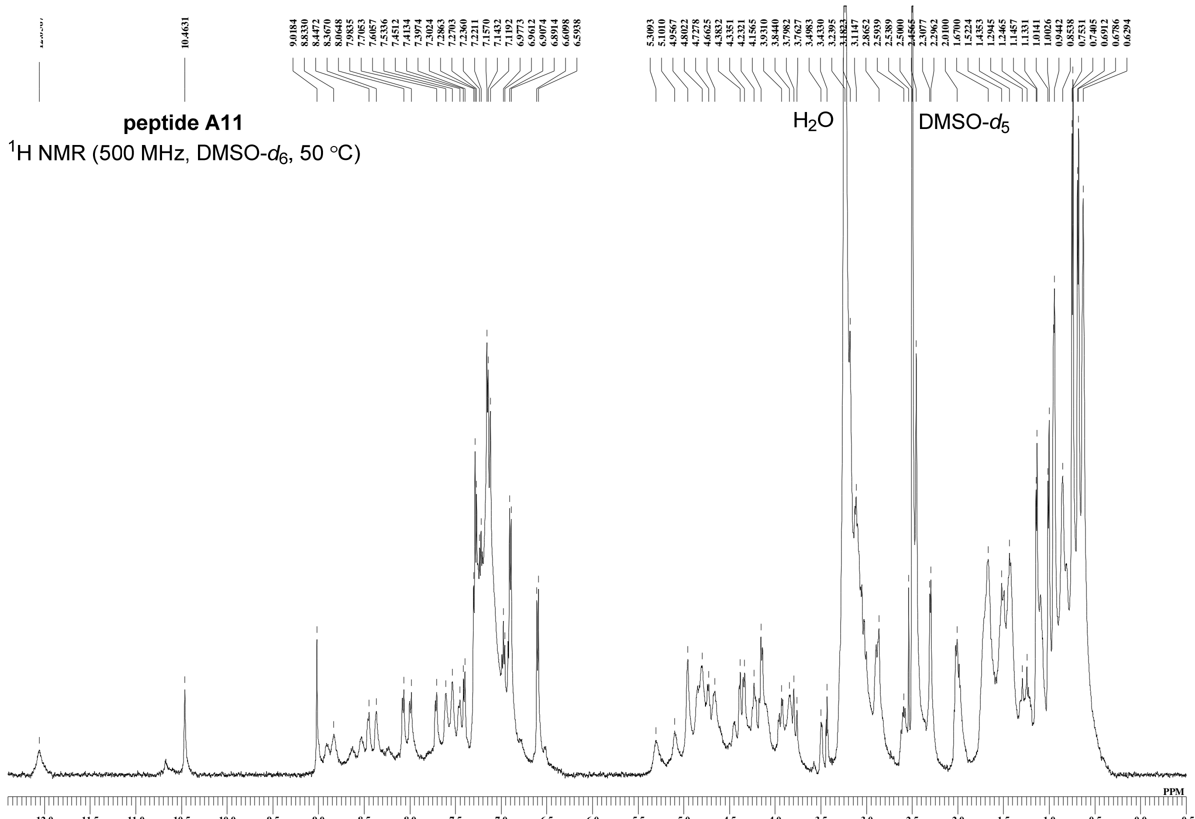

Supplementary Figure 58. ^{1}H NMR spectra of **A10** and **A11**. The spectra were obtained in DMSO-d_6 at 50 °C.

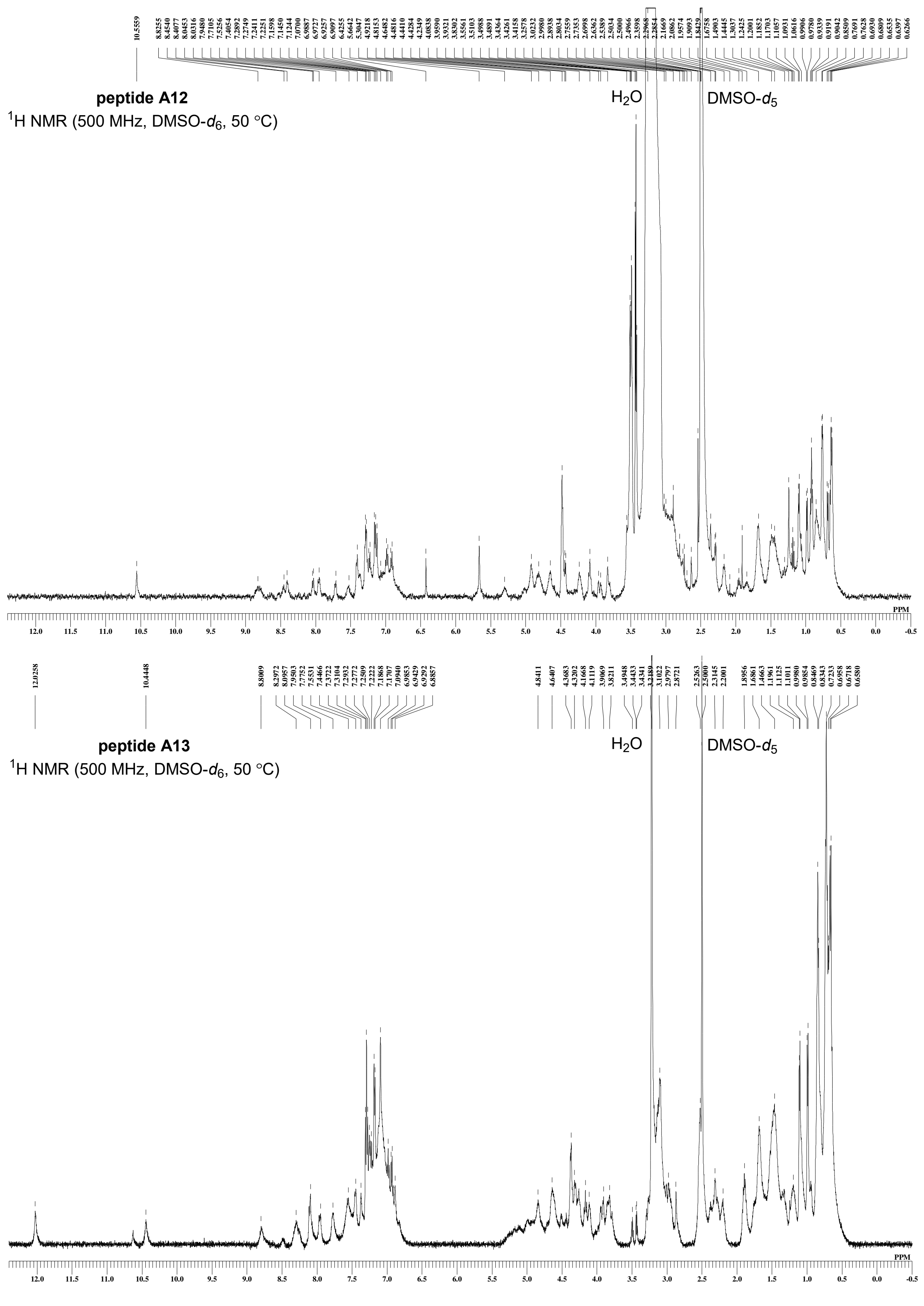

Supplementary Figure 59. ^{1}H NMR spectra of **A12** and **A13**. The spectra were obtained in DMSO-d_6 at 50 °C.

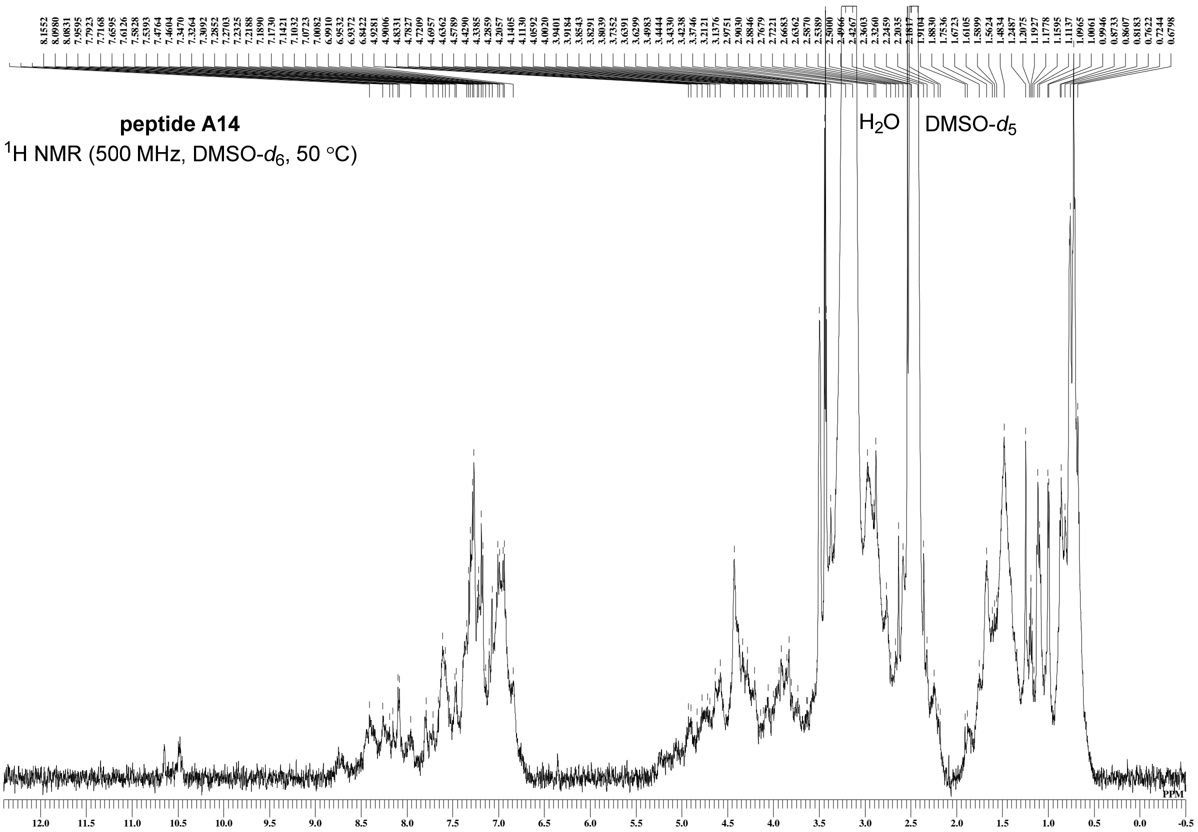

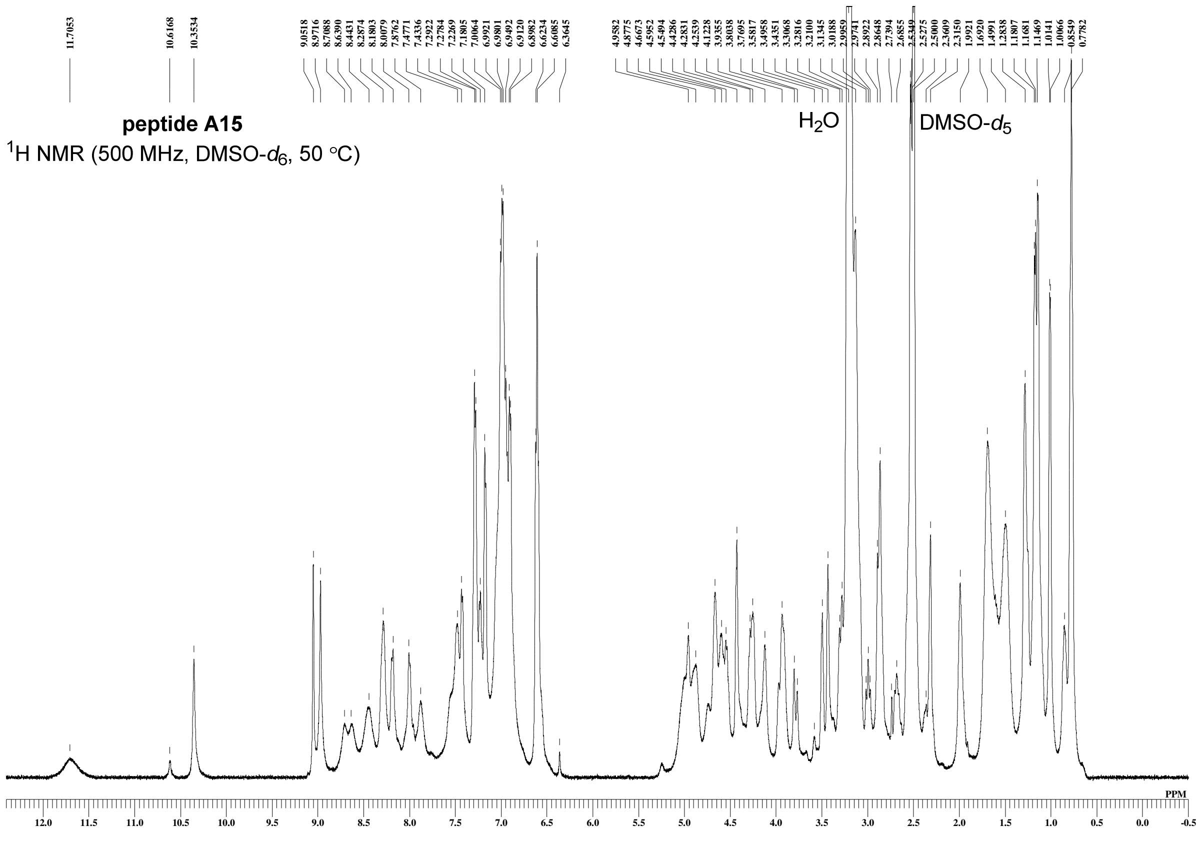

Supplementary Figure 60. ^{1}H NMR spectra of **A14** and **A15**. The spectra were obtained in DMSO-d_6 at 50 °C.

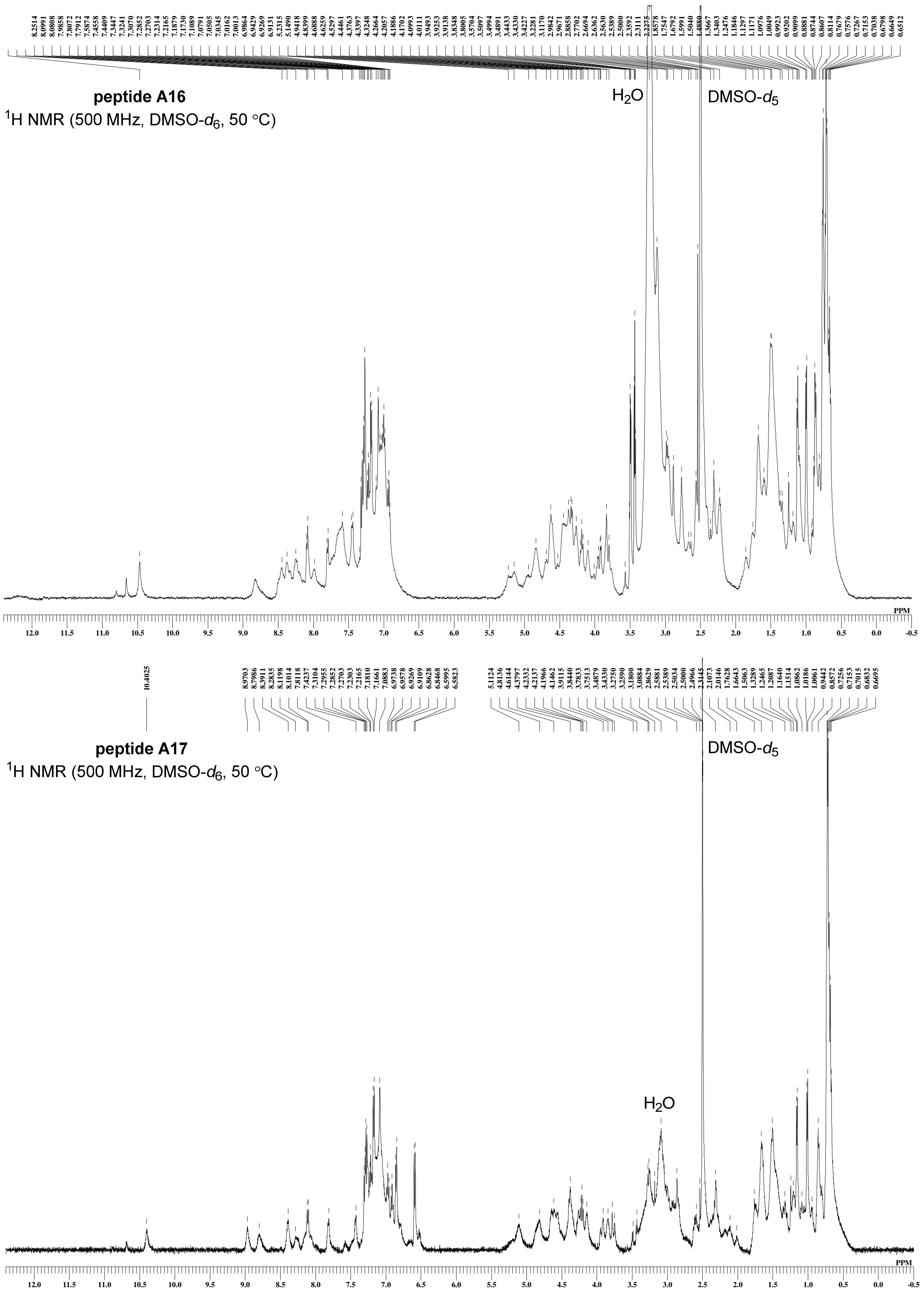

Supplementary Figure 61. ^{1}H NMR spectra of **A16** and **A17**. The spectra were obtained in DMSO-d_6 at 50 °C.

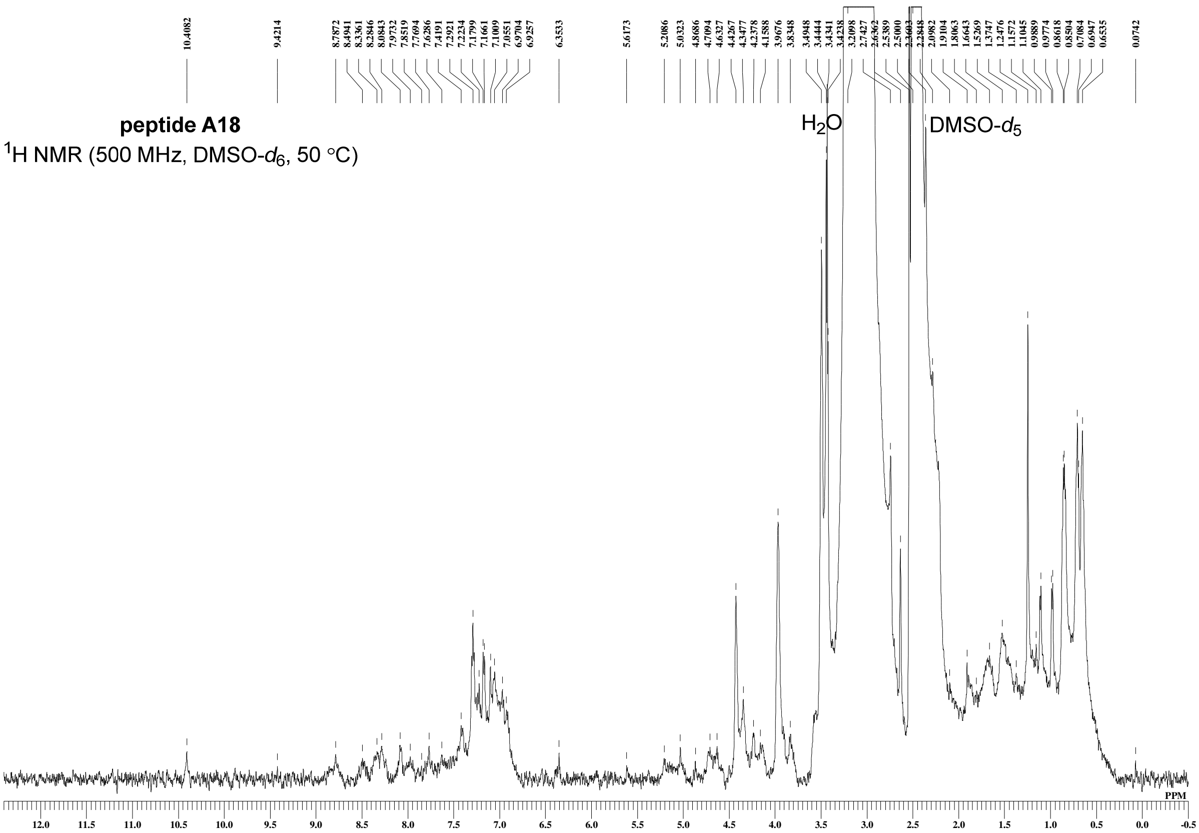

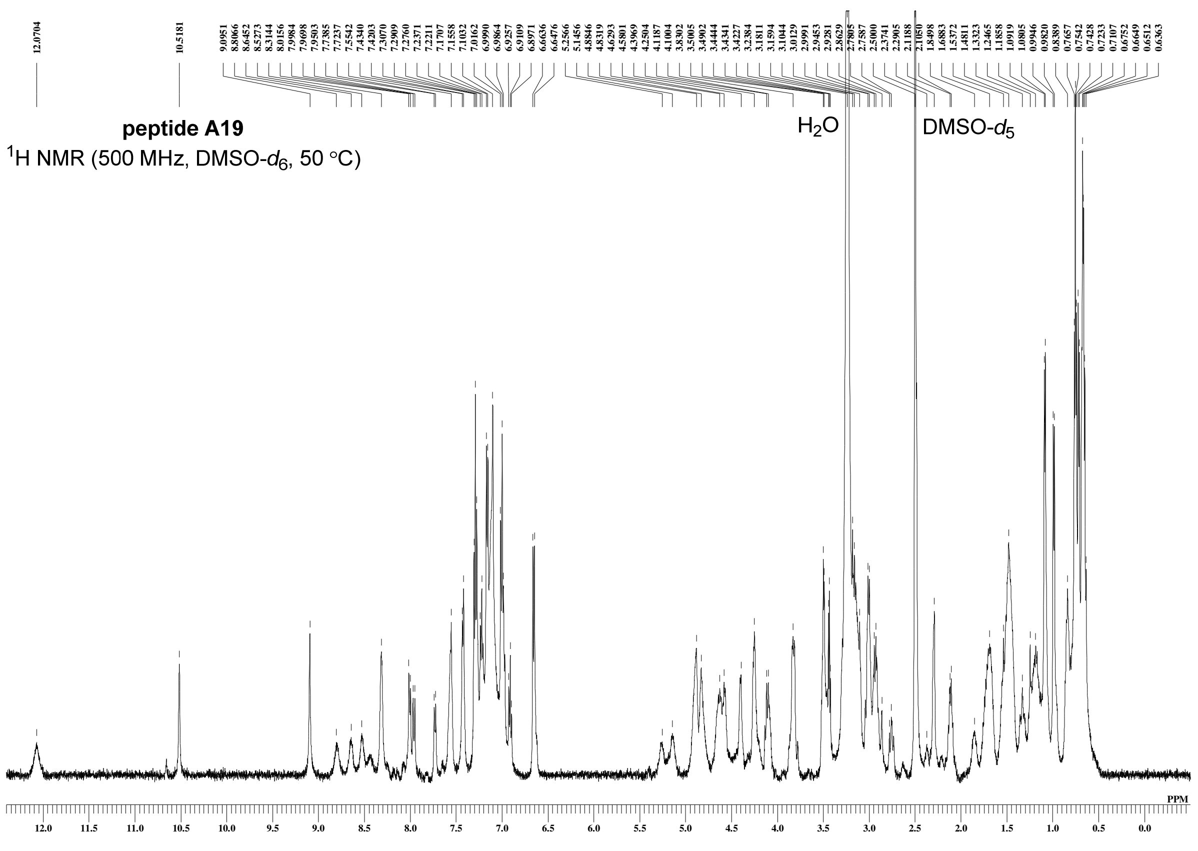

Supplementary Figure 62. ^{1}H NMR spectra of **A18** and **A19**. The spectra were obtained in DMSO-d_6 at 50 °C.

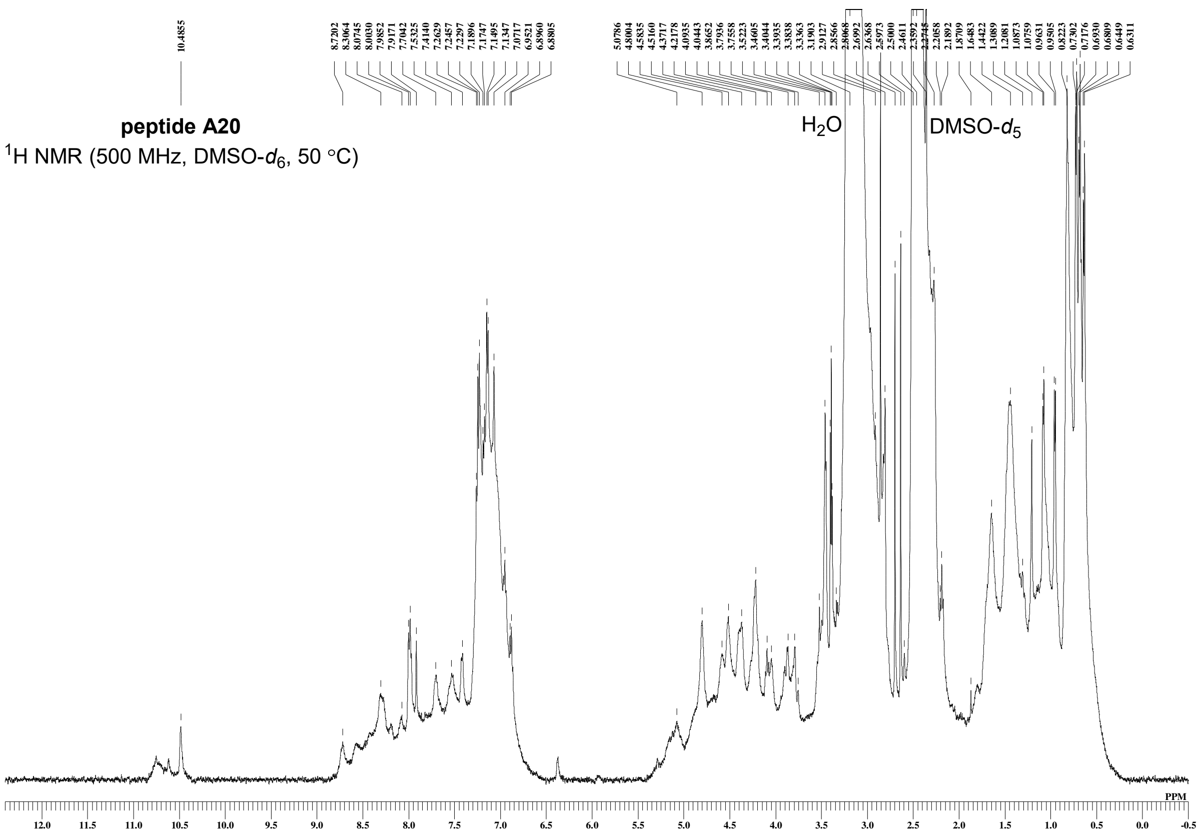

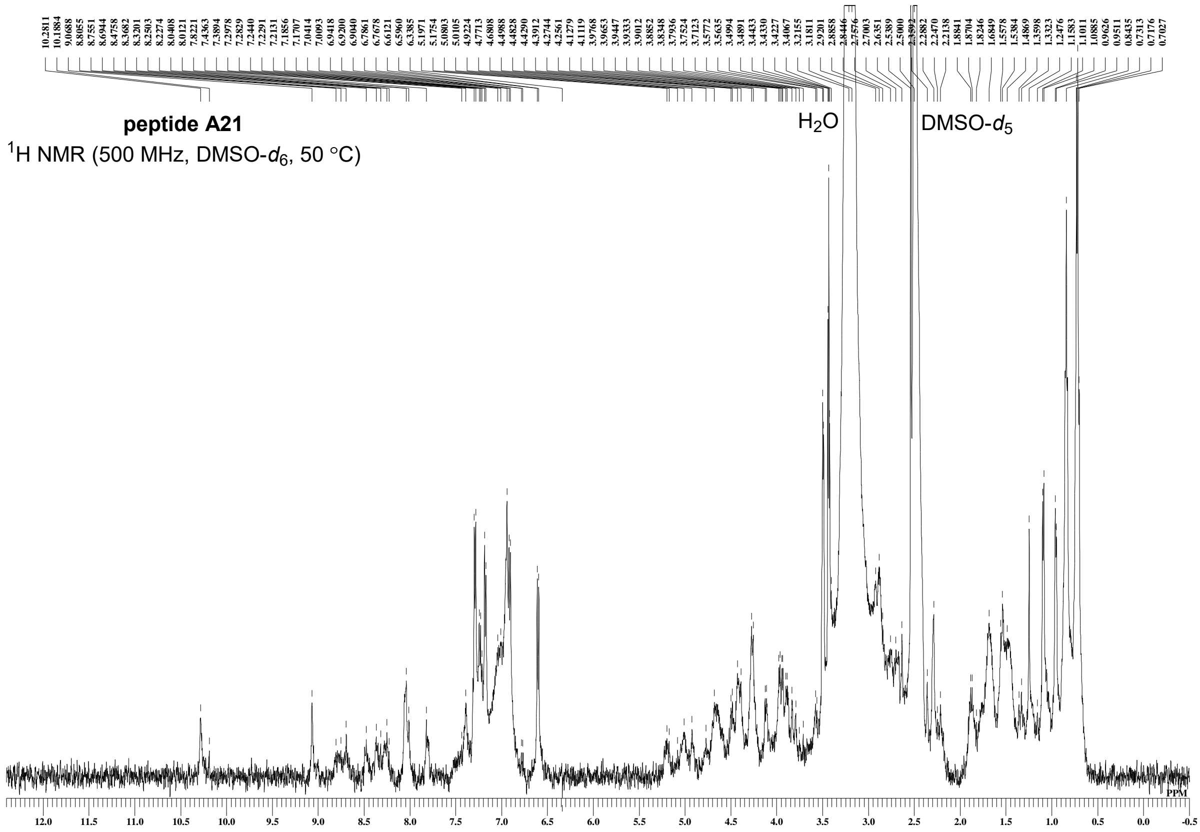

Supplementary Figure 63. ^{1}H NMR spectra of **A20** and **A21**. The spectra were obtained in DMSO-d_6 at 50 °C.

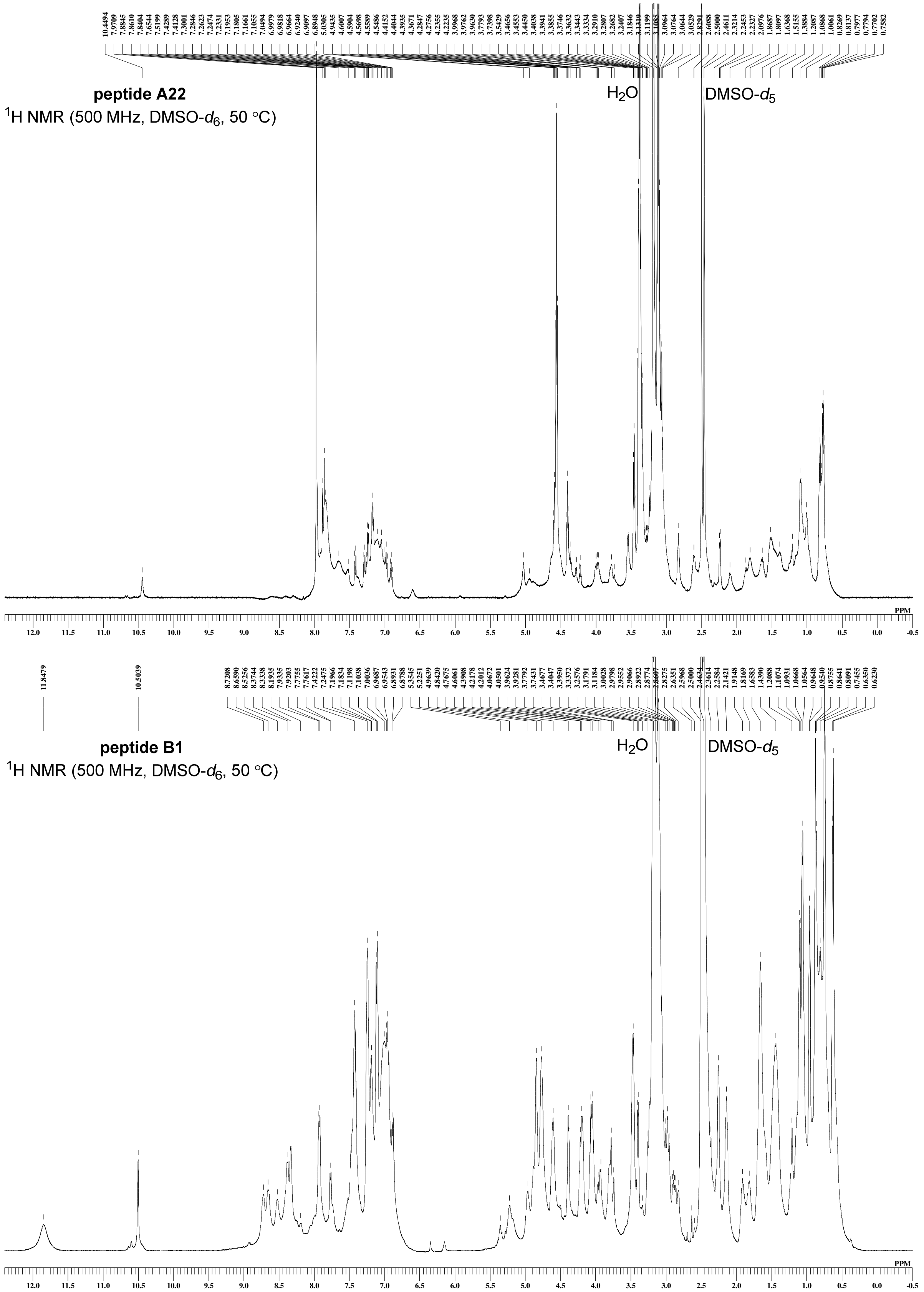

Supplementary Figure 64. ^{1}H NMR spectra of **A22** and **B1**. The spectra were obtained in DMSO-d_6 at 50 °C.

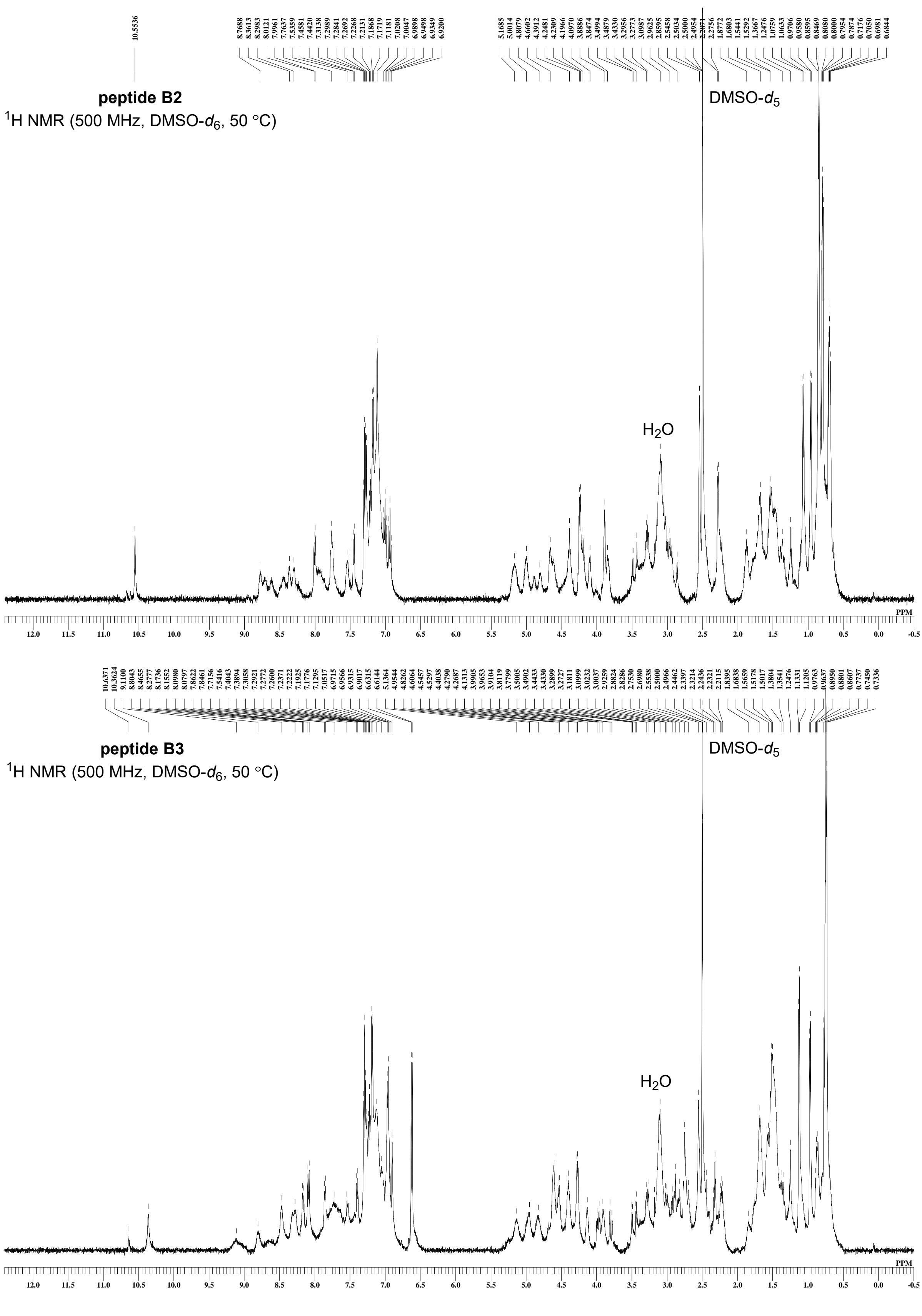

Supplementary Figure 65. ^{1}H NMR spectra of **B2** and **B3**. The spectra were obtained in DMSO-d_6 at 50 °C.

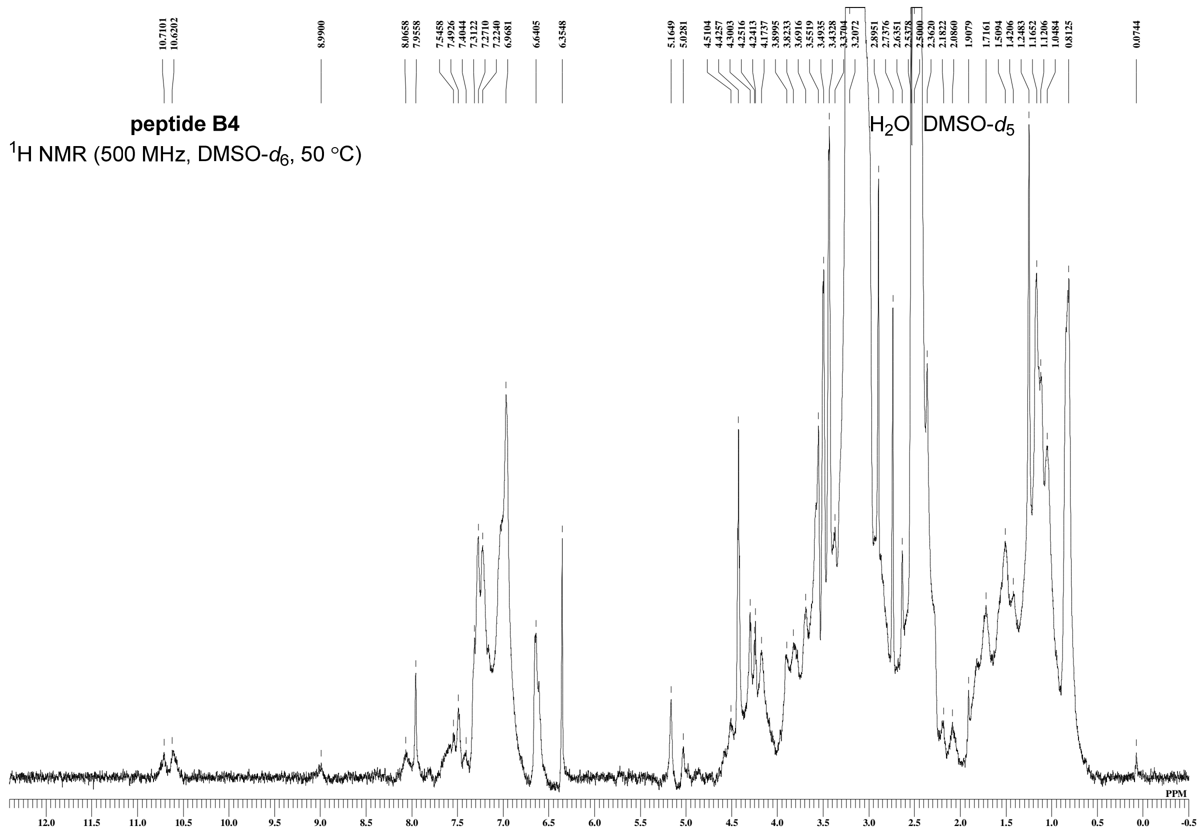

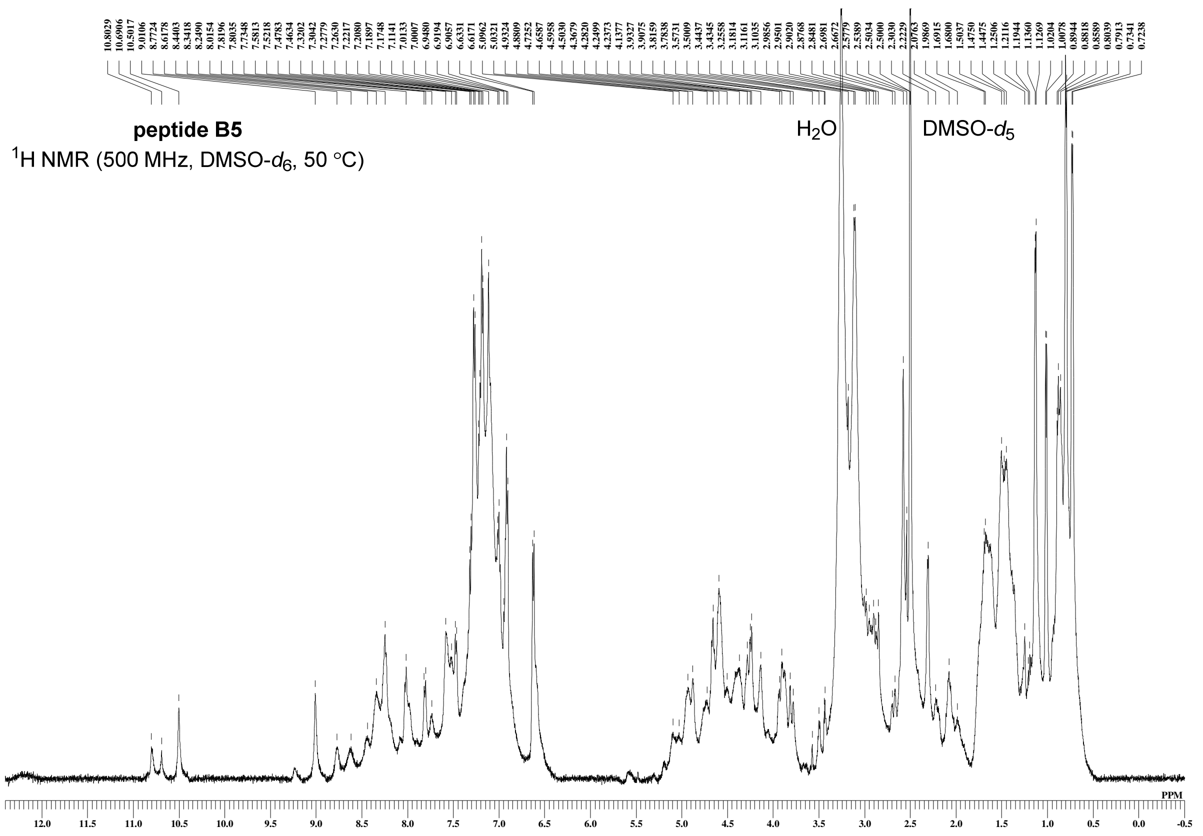

Supplementary Figure 66. ^{1}H NMR spectra of **B4** and **B5**. The spectra were obtained in DMSO-d_6 at 50 °C.

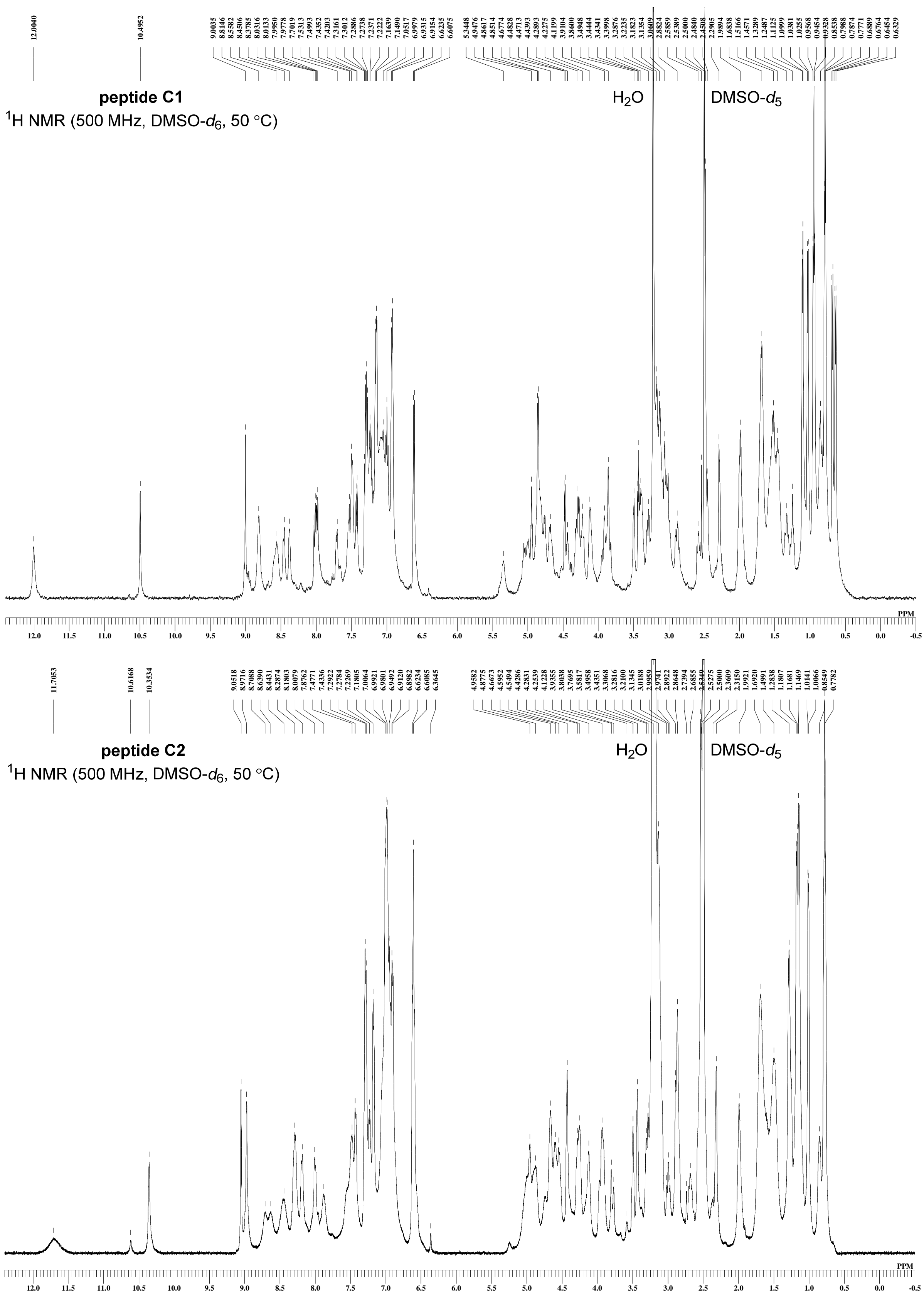

Supplementary Figure 67. ^1H NMR spectra of **C1** and **C2**. The spectra were obtained in DMSO-d_6 at 50 °C.

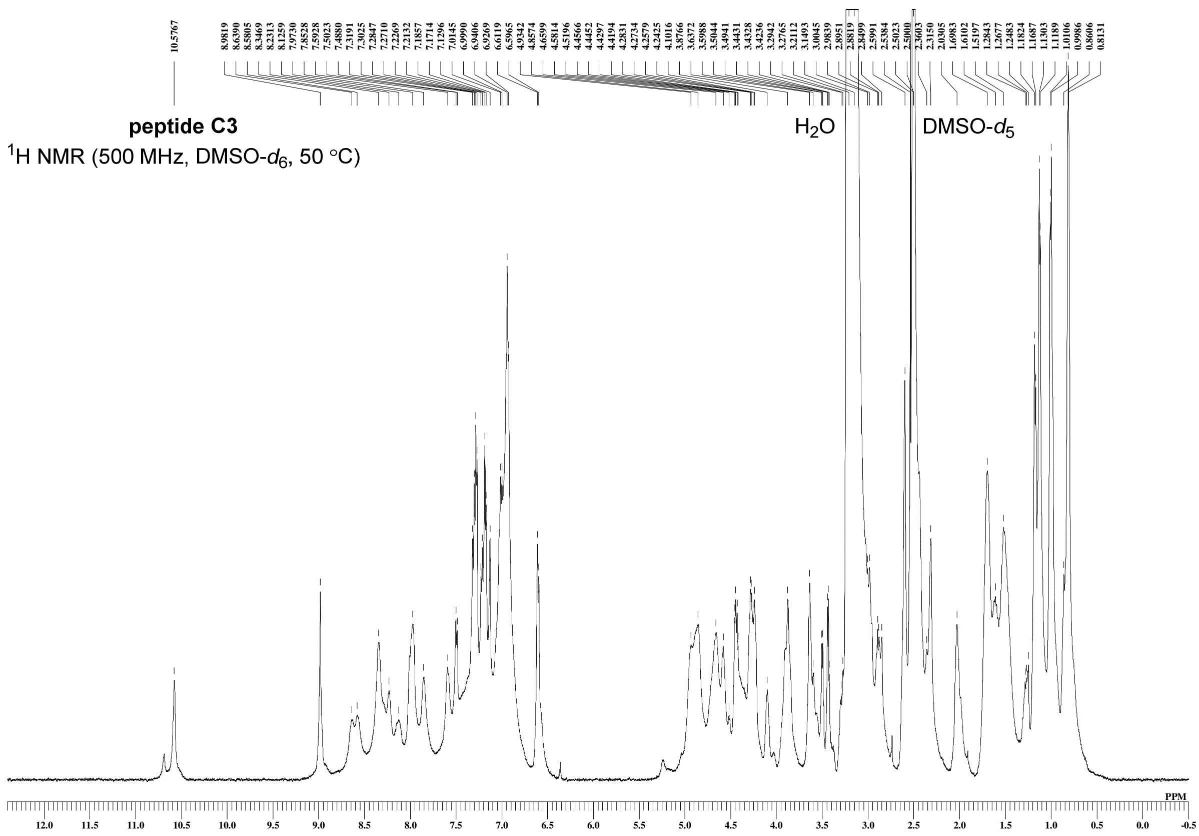

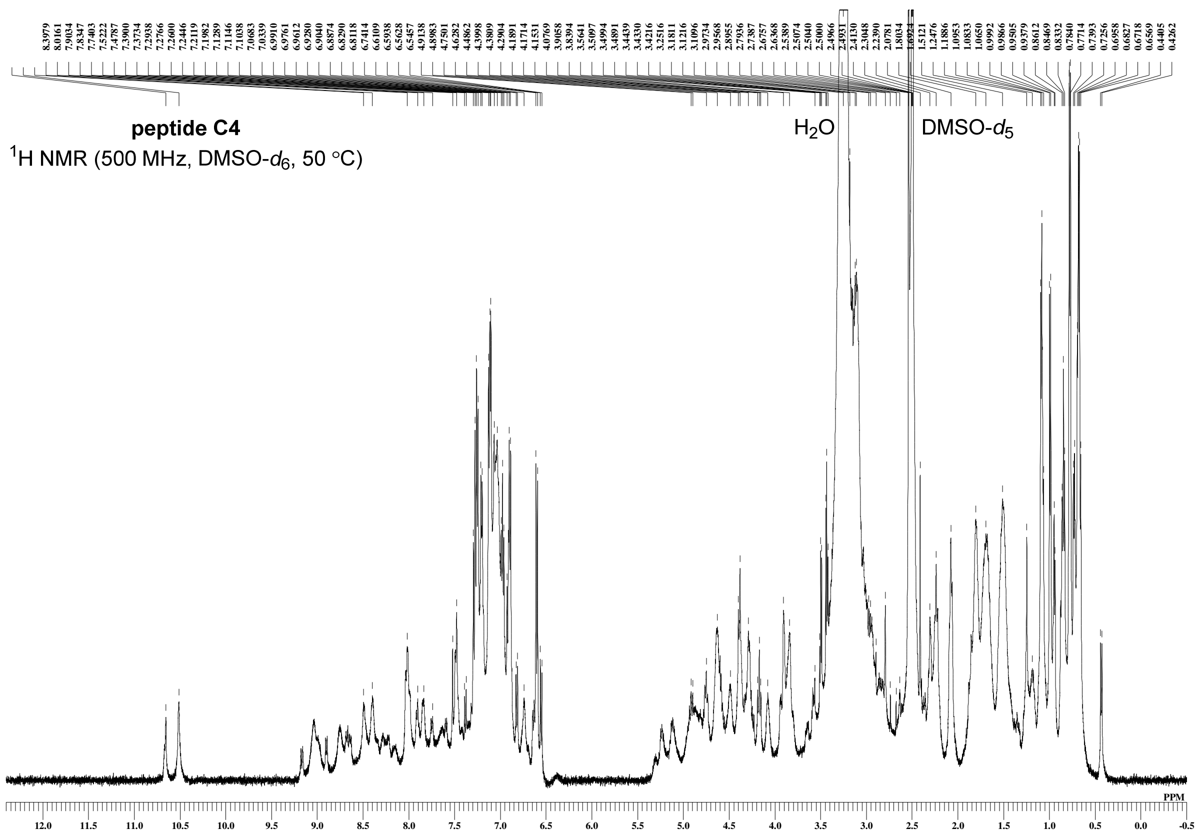

Supplementary Figure 68. ^{1}H NMR spectra of **C3** and **C4**. The spectra were obtained in DMSO-d_6 at 50 °C.

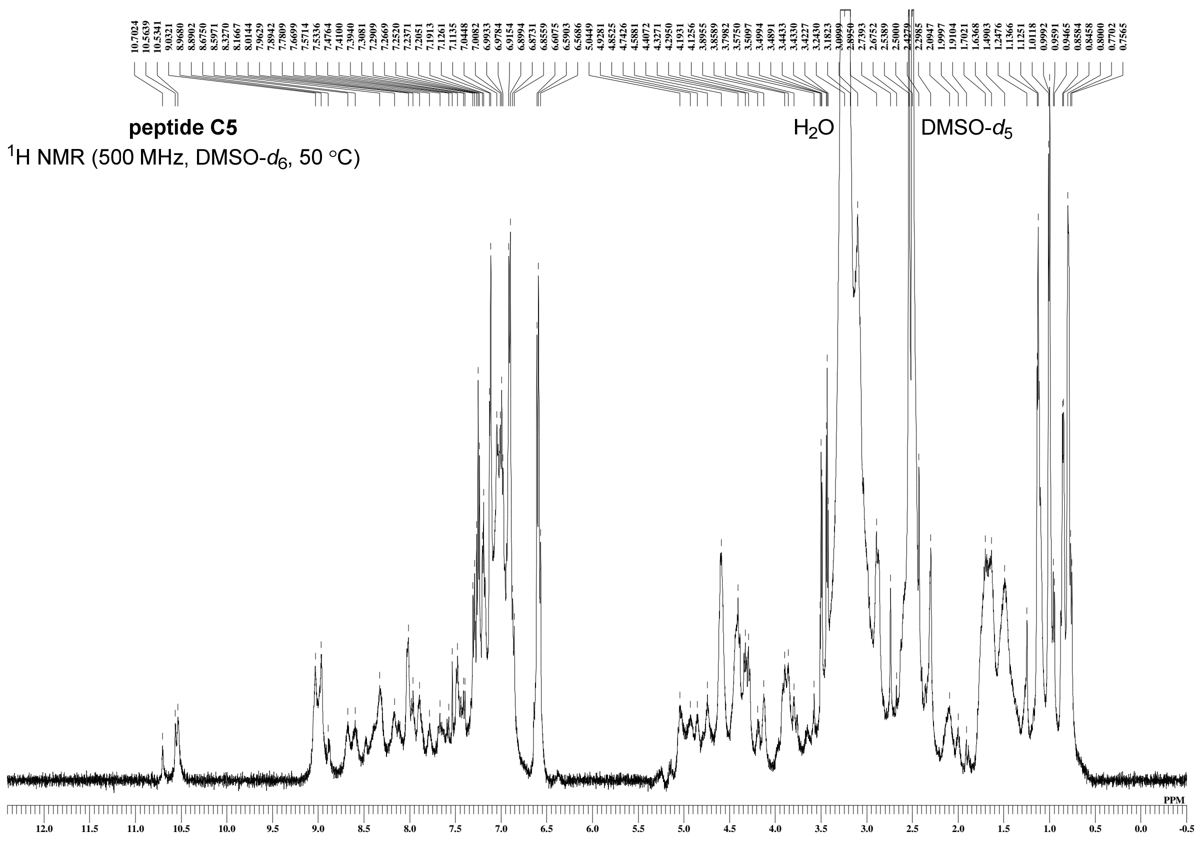

Supplementary Figure 69. ^{1}H NMR spectrum of **C5**. The spectrum was obtained in DMSO-d_6 at 50 °C.

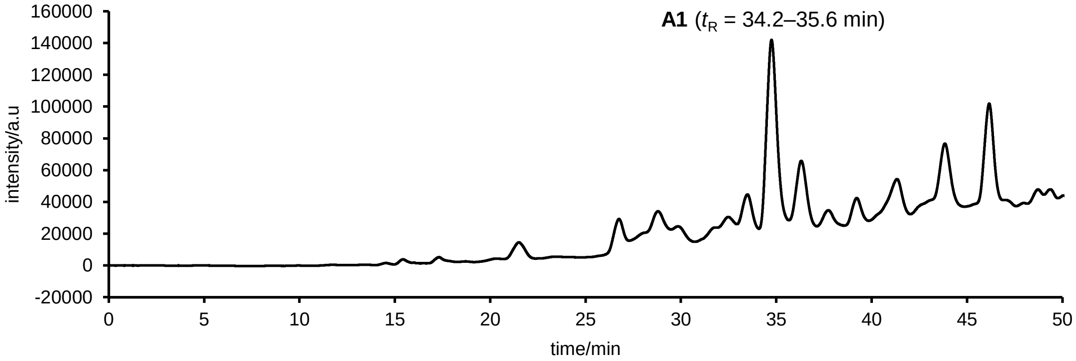

Supplementary Figure 70. HPLC chart for 1st HPLC purification of **A1**. Column: Inertsil C8-3 20 × 250 mm, eluent A: MeOH + 0.05% TFA, eluent B: H_2O + 0.05% TFA, linear gradient A/B = 60/45 to 85/15 over 50 min, flow rate: 5.0 mL/min, detection: photodiode array detector 199–651 nm (UV chromatogram: 280 nm).

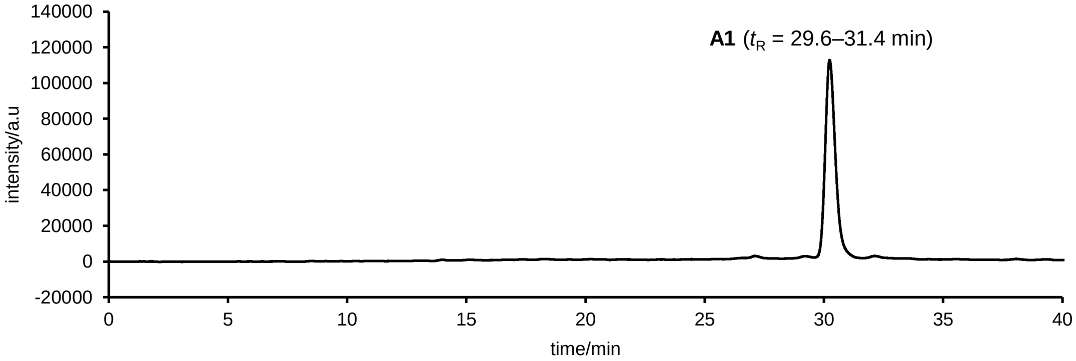

Supplementary Figure 71. HPLC chart for 2nd HPLC purification of **A1**. Column: Inertsil C8-3 20 × 250 mm, eluent A: MeCN + 0.05% TFA, eluent B: H_2O + 0.05% TFA, linear gradient A/B = 37.5/62.5 to 45/55 over 30 min, then 45/55 over 10 min, flow rate: 5.0 mL/min, detection: photodiode array detector 199–651 nm (UV chromatogram: 280 nm).

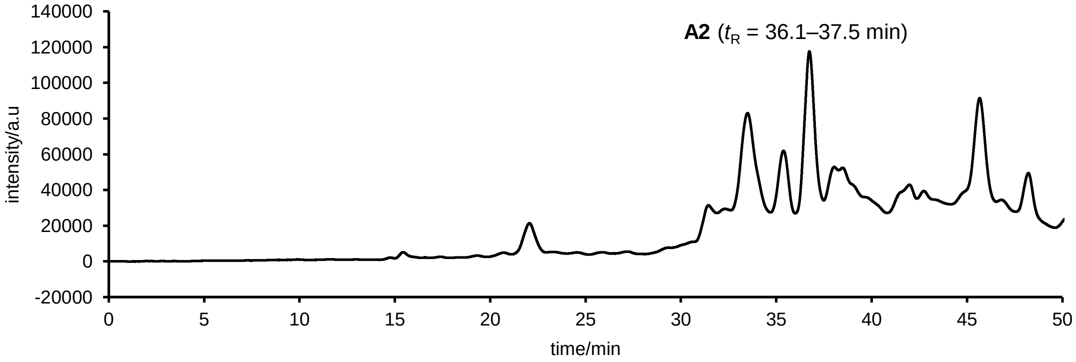

Supplementary Figure 72. HPLC chart for 1st HPLC purification of **A2**. Column: Inertsil C8-3 20 × 250 mm, eluent A: MeOH + 0.05% TFA, eluent B: H_2O + 0.05% TFA, linear gradient A/B = 60/40 to 85/15 over 50 min, flow rate: 5.0 mL/min, detection: photodiode array detector 199–651 nm (UV chromatogram: 280 nm).

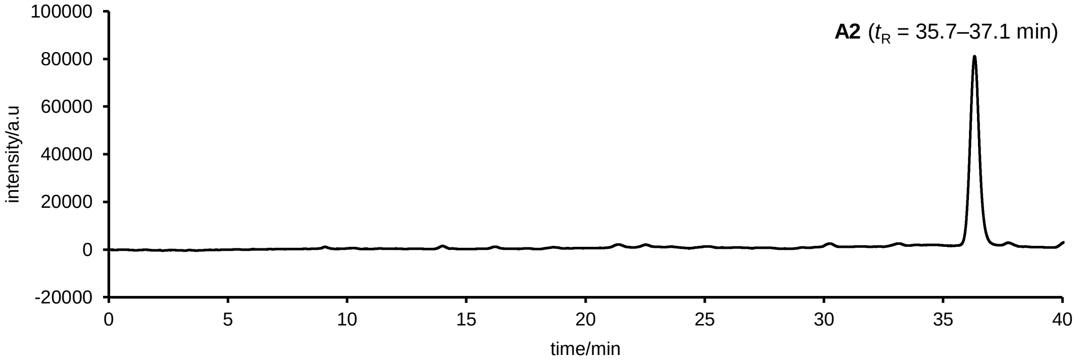

Supplementary Figure 73. HPLC chart for 2nd HPLC purification of **A2**. Column: Inertsil C8-3 20 × 250 mm, eluent A: MeCN + 0.05% TFA, eluent B: H_2O + 0.05% TFA, linear gradient A/B = 37.5/62.5 to 45/55 over 30 min, then 45/55 over 10 min, flow rate: 5.0 mL/min, detection: photodiode array detector 199–651 nm (UV chromatogram: 280 nm).

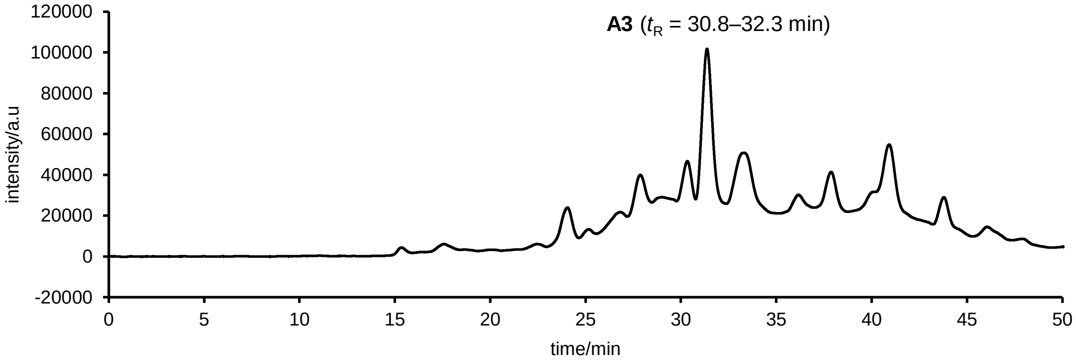

Supplementary Figure 74. HPLC chart for 1st HPLC purification of **A3**. Column: Inertsil C8-3 20 × 250 mm, eluent A: MeOH + 0.05% TFA, eluent B: H_2O + 0.05% TFA, linear gradient A/B = 60/40 to 85/15 over 50 min, flow rate: 5.0 mL/min, detection: photodiode array detector 199–651 nm (UV chromatogram: 280 nm).

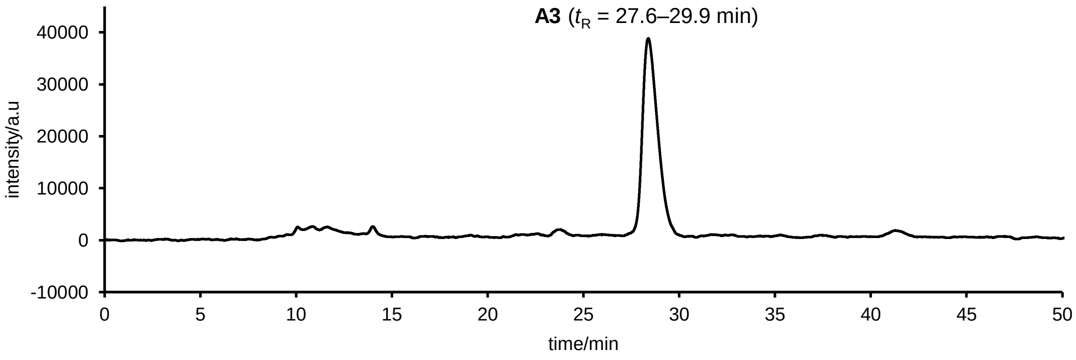

Supplementary Figure 75. HPLC chart for 2nd HPLC purification of **A3**. Column: Inertsil C8-3 20 × 250 mm, eluent A: MeCN + 0.05% TFA, eluent B: H_2O + 0.05% TFA, A/B = 37.5/62.5 over 50 min, flow rate: 5.0 mL/min, detection: photodiode array detector 199–651 nm (UV chromatogram: 280 nm).

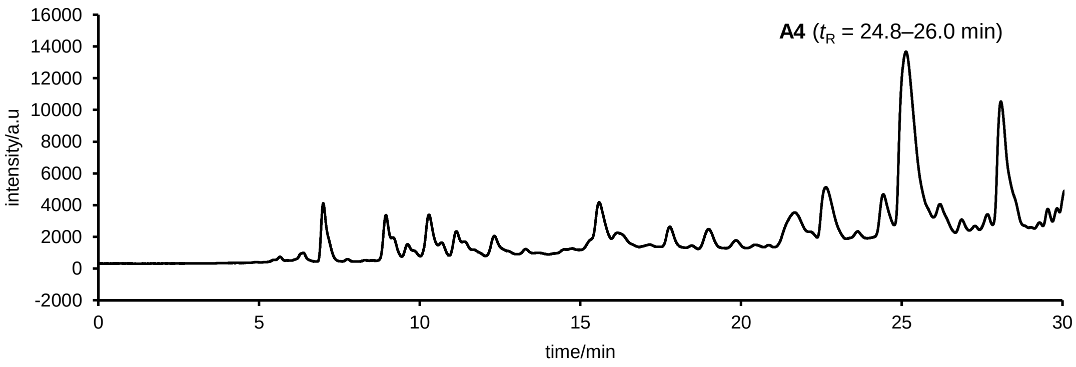

Supplementary Figure 76. HPLC chart for 1st HPLC purification of **A4**. Column: Inertsil ODS-4 10 × 250 mm, eluent A: MeCN + 0.05% TFA, eluent B: H_2O + 0.05% TFA, linear gradient A/B = 35/65 to 50/50 over 25 min, then 50/50 over 5 min, flow rate: 3.0 mL/min, detection: UV 280 nm.

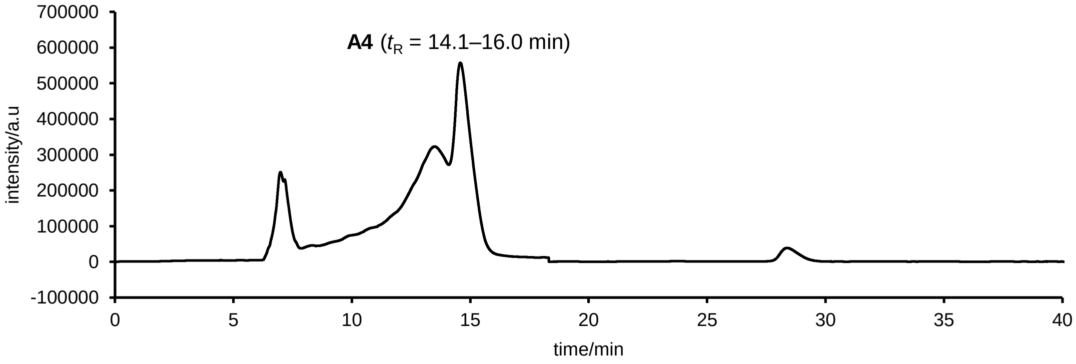

Supplementary Figure 77. HPLC chart for 2nd HPLC purification of **A4**. Column: Inertsil ODS-4 10 × 250 mm, eluent A: MeOH + 0.05% TFA, eluent B: H_2O + 0.05% TFA, linear gradient A/B = 70/30 to 90/10 over 40 min, flow rate: 2.0 mL/min, detection: UV 220 nm.

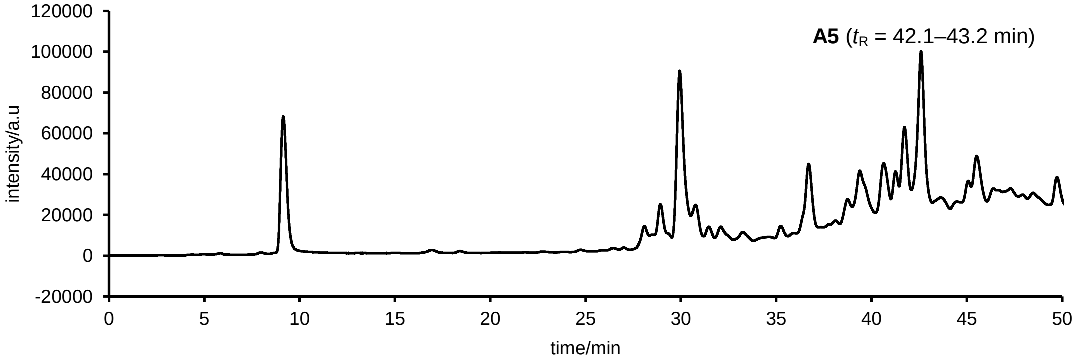

Supplementary Figure 78. HPLC chart for 1st HPLC purification of **A5**. Column: Inertsil C8-3 10 × 250 mm, eluent A: MeCN + 0.05% TFA, eluent B: H_2O + 0.05% TFA, linear gradient A/B = 25/75 to 50/50 over 50 min, flow rate: 3.0 mL/min, detection: photodiode array detector 199–651 nm (UV chromatogram: 280 nm).

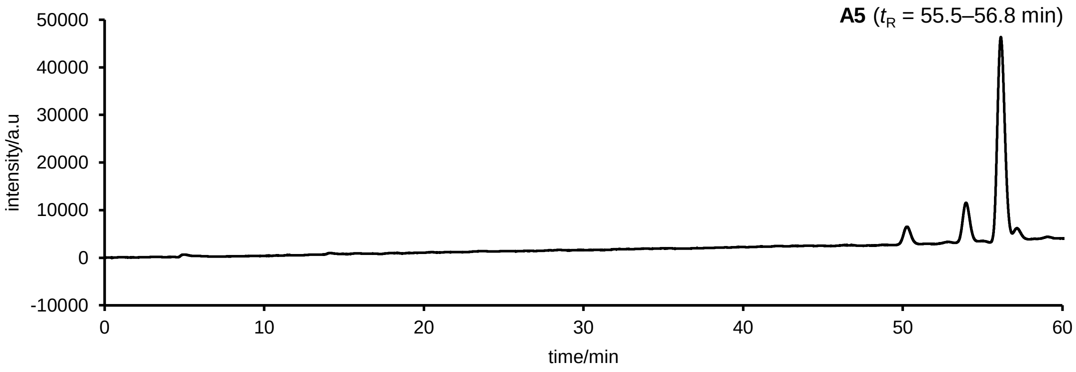

Supplementary Figure 79. HPLC chart for 2nd HPLC purification of **A5**. Column: Inertsil C8-3 10 × 250 mm, eluent A: MeOH + 0.05% TFA, eluent B: H_2O + 0.05% TFA, linear gradient A/B = 45/55 to 75/25 over 60 min, flow rate: 3.0 mL/min, detection: photodiode array detector 199–651 nm (UV chromatogram: 280 nm).

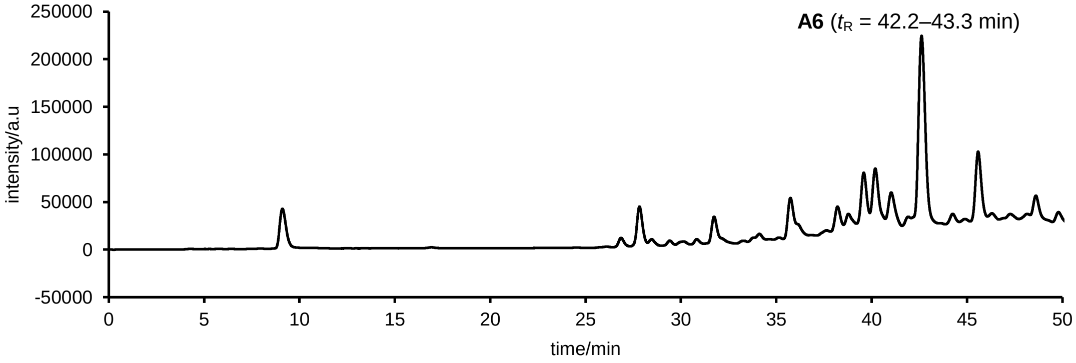

Supplementary Figure 80. HPLC chart for 1st HPLC purification of **A6**. Column: Inertsil C8-3 10 × 250 mm, eluent A: MeCN + 0.05% TFA, eluent B: H_2O + 0.05% TFA, linear gradient A/B = 25/75 to 50/50 over 50 min, flow rate: 3.0 mL/min, detection: photodiode array detector 199–651 nm (UV chromatogram: 280 nm).

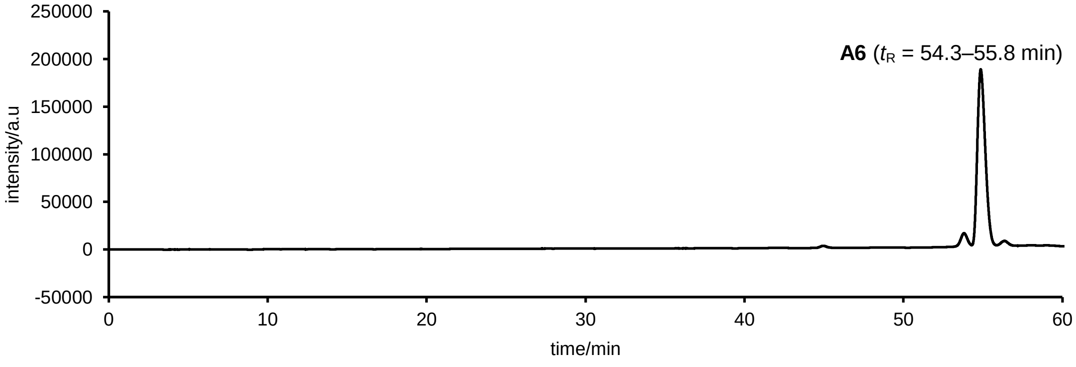

Supplementary Figure 81. HPLC chart for 2nd HPLC purification of **A6**. Column: Inertsil C8-3 10 × 250 mm, eluent A: MeOH + 0.05% TFA, eluent B: H_2O + 0.05% TFA, linear gradient A/B = 45/55 to 75/25 over 60 min, flow rate: 3.0 mL/min, detection: photodiode array detector 199–651 nm (UV chromatogram: 280 nm).

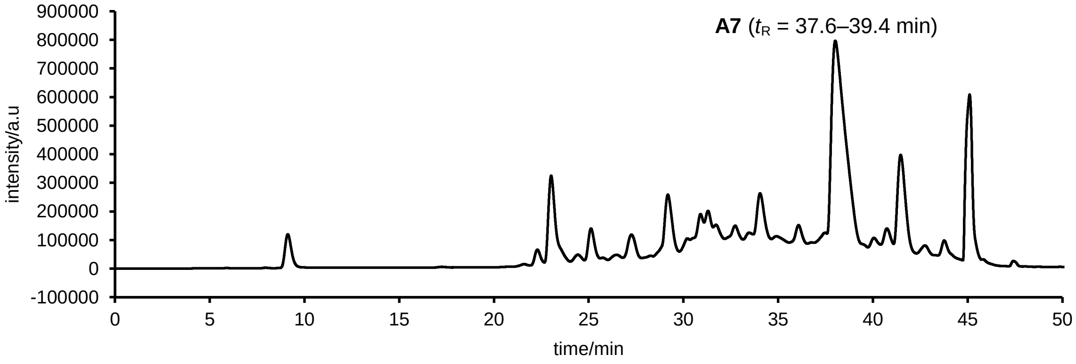

Supplementary Figure 82. HPLC chart for 1st HPLC purification of **A7**. Column: Inertsil C8-3 10 × 250 mm, eluent A: MeCN + 0.05% TFA, eluent B: H_2O + 0.05% TFA, linear gradient A/B = 25/75 to 50/50 over 50 min, flow rate: 3.0 mL/min, detection: photodiode array detector 199–651 nm (UV chromatogram: 280 nm).

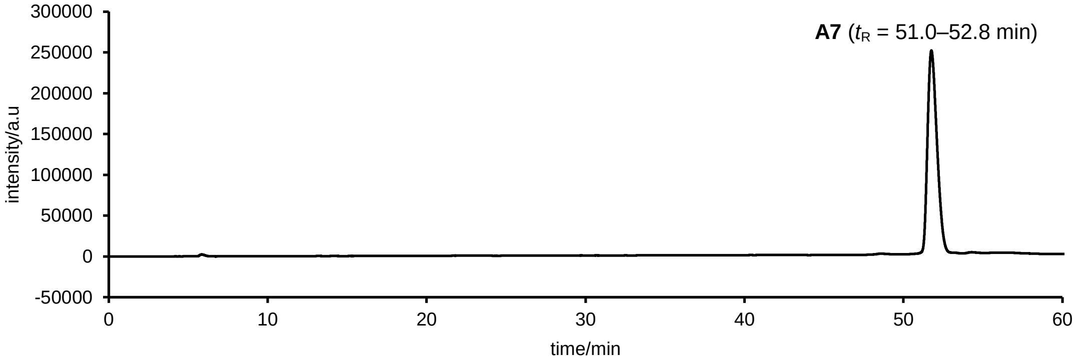

Supplementary Figure 83. HPLC chart for 2nd HPLC purification of **A7**. Column: Inertsil C8-3 10 × 250 mm, eluent A: MeOH + 0.05% TFA, eluent B: H_2O + 0.05% TFA, linear gradient A/B = 45/55 to 75/25 over 60 min, flow rate: 3.0 mL/min, detection: photodiode array detector 199–651 nm (UV chromatogram: 280 nm).

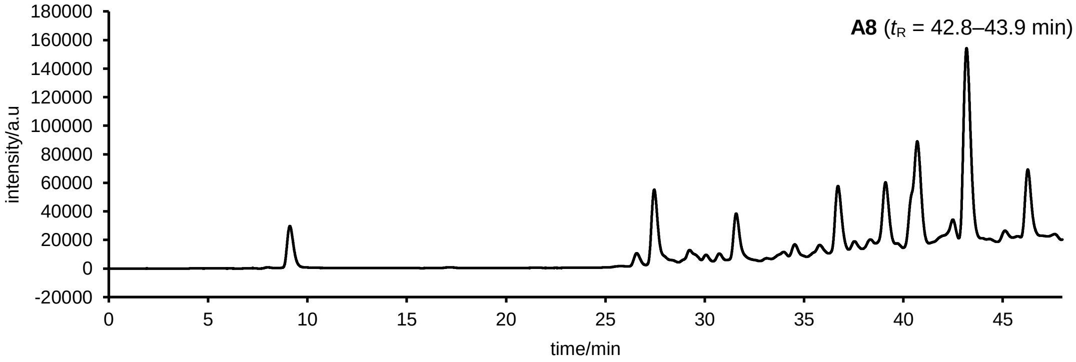

Supplementary Figure 84. HPLC chart for 1st HPLC purification of **A8**. Column: Inertsil C8-3 10 × 250 mm, eluent A: MeCN + 0.05% TFA, eluent B: H_2O + 0.05% TFA, linear gradient A/B = 25/75 to 49/51 over 48 min, flow rate: 3.0 mL/min, detection: photodiode array detector 199–651 nm (UV chromatogram: 280 nm).

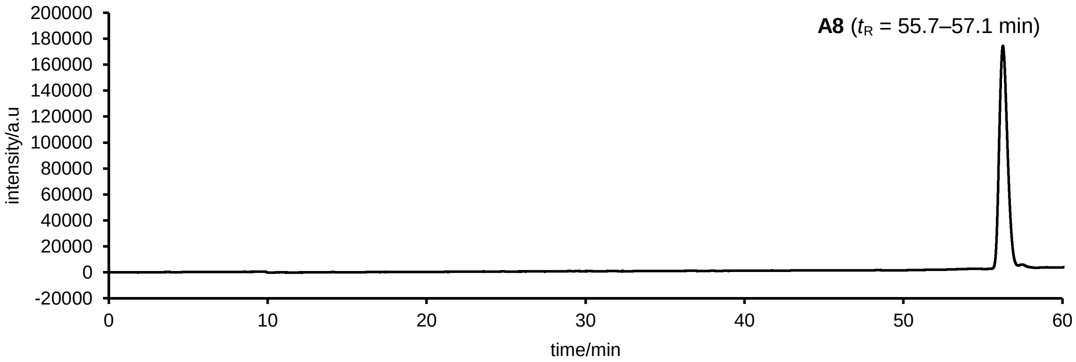

Supplementary Figure 85. HPLC chart for 2nd HPLC purification of **A8**. Column: Inertsil C8-3 10 × 250 mm, eluent A: MeOH + 0.05% TFA, eluent B: H_2O + 0.05% TFA, linear gradient A/B = 45/55 to 75/25 over 60 min, flow rate: 3.0 mL/min, detection: photodiode array detector 199–651 nm (UV chromatogram: 280 nm).

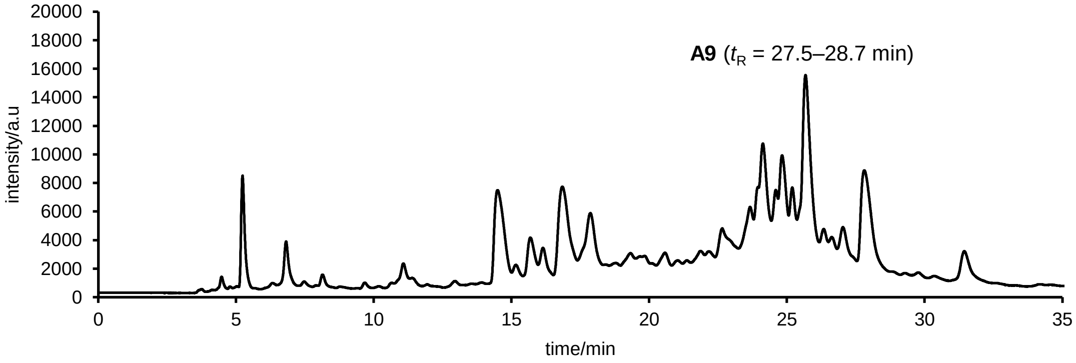

Supplementary Figure 86. HPLC chart for 1st HPLC purification of **A9**. Column: Inertsil ODS-4 4.6 × 250 mm, eluent A: MeCN + 0.05% TFA, eluent B: H_2O + 0.05% TFA, linear gradient A/B = 35/65 to 50/50 over 20 min, then 50/50 over 15 min, flow rate: 1.0 mL/min, detection: UV 280 nm.

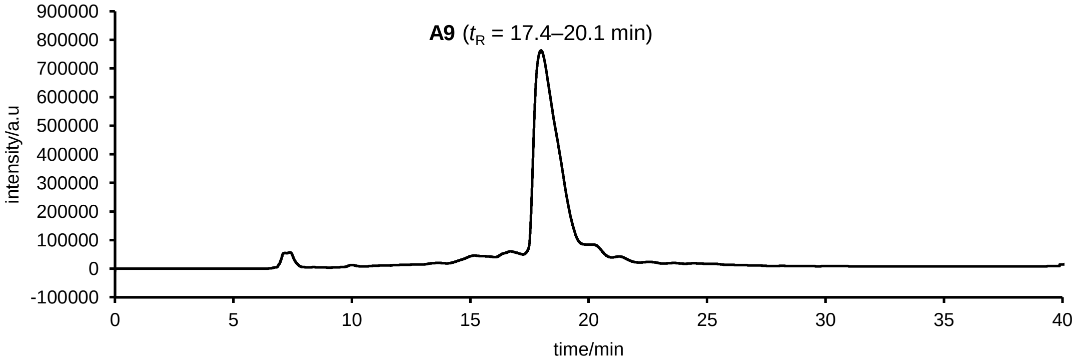

Supplementary Figure 87. HPLC chart for 2nd HPLC purification of **A9**. Column: Inertsil ODS-4 10 × 250 mm, eluent A: MeOH + 0.05% TFA, eluent B: H_2O + 0.05% TFA, linear gradient A/B = 70/30 to 90/10 over 40 min, flow rate: 2.0 mL/min, detection: UV 220 nm.

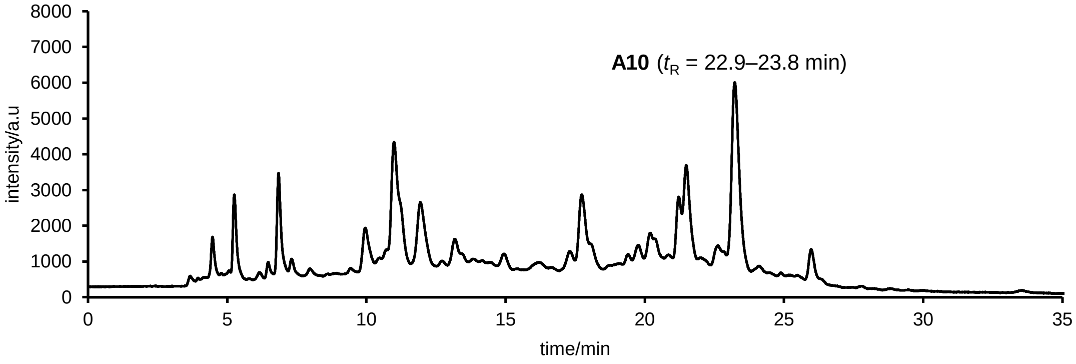

Supplementary Figure 88. HPLC chart for 1st HPLC purification of **A10**. Column: Inertsil ODS-4 4.6 × 250 mm, eluent A: MeCN + 0.05% TFA, eluent B: H_2O + 0.05% TFA, linear gradient A/B = 35/65 to 50/50 over 20 min, then 50/50 over 15 min, flow rate: 1.0 mL/min, detection: UV 280 nm.

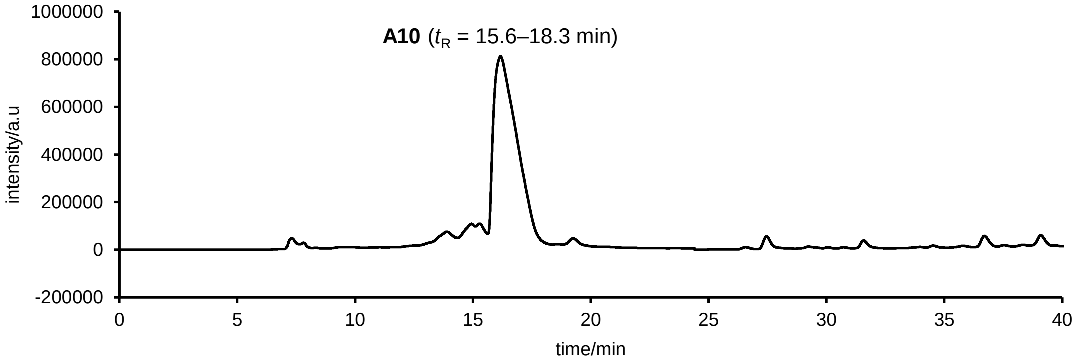

Supplementary Figure 89. HPLC chart for 2nd HPLC purification of **A10**. Column: Inertsil ODS-4 10 × 250 mm, eluent A: MeOH + 0.05% TFA, eluent B: H_2O + 0.05% TFA, linear gradient A/B = 70/30 to 90/10 over 40 min, flow rate: 2.0 mL/min, detection: UV 220 nm.

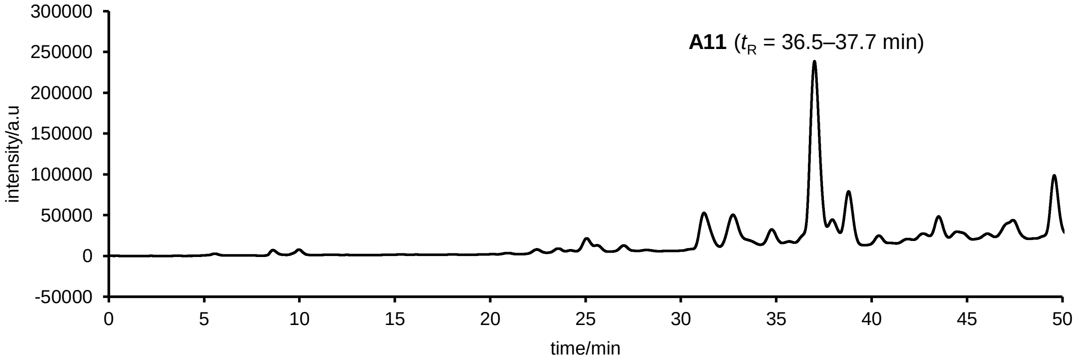

Supplementary Figure 90. HPLC chart for 1st HPLC purification for **A11**. Column: Inertsil C8-3 10 × 250 mm, eluent A: MeOH + 0.05% TFA, eluent B: H_2O + 0.05% TFA, linear gradient A/B = 55/45 to 80/20 over 50 min, flow rate: 3.0 mL/min, detection: photodiode array detector 199–651 nm (UV chromatogram: 280 nm).

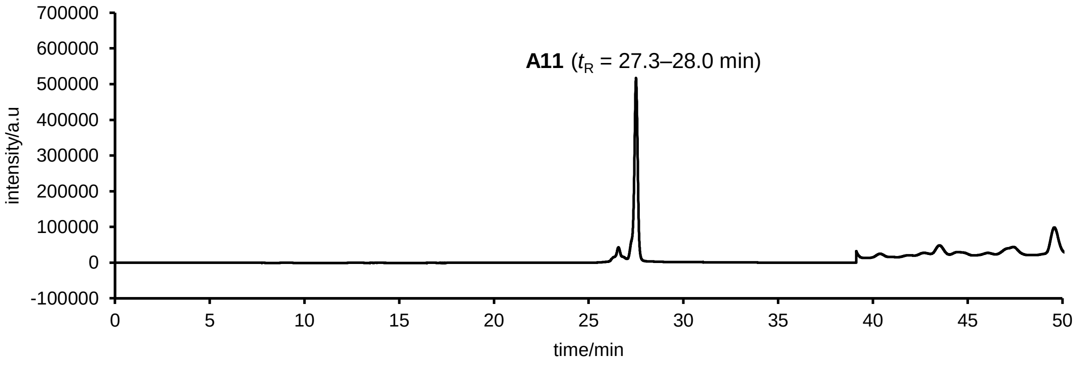

Supplementary Figure 91. HPLC chart for 2nd HPLC purification for **A11**. Column: Inertsil C8-3 10 × 250 mm, eluent A: MeOH/MeCN (2/1) + 0.05% TFA, eluent B: H_2O + 0.05% TFA, linear gradient A/B = 40/60 to 75/25 over 50 min, flow rate: 3.0 mL/min, detection: photodiode array detector 199–651 nm (UV chromatogram: 280 nm).

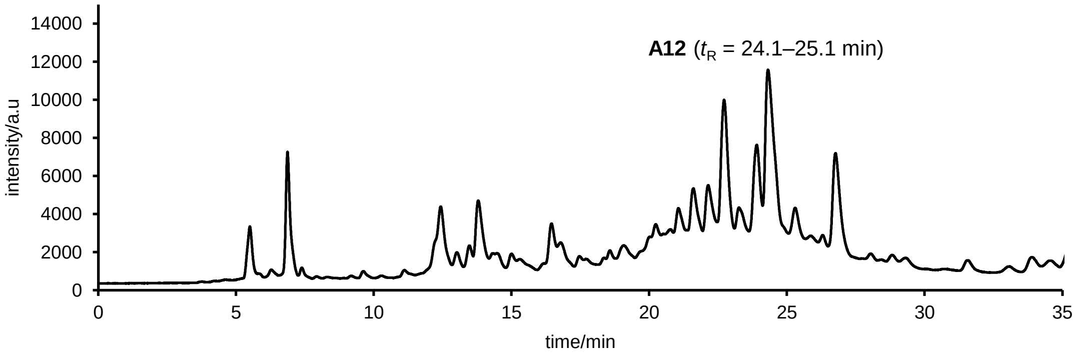

Supplementary Figure 92. HPLC chart for 1st HPLC purification of **A12**. Column: Inertsil ODS-4 10 × 250 mm, eluent A: MeCN + 0.05% TFA, eluent B: H_2O + 0.05% TFA, linear gradient A/B = 35/65 to 50/50 over 25 min, then 50/50 over 10 min, flow rate: 3.0 mL/min, detection: UV 280 nm.

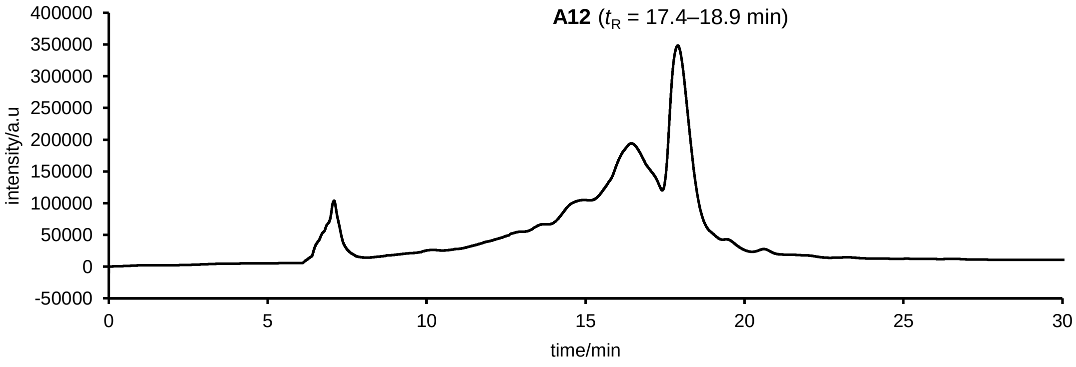

Supplementary Figure 93. HPLC chart for 2nd HPLC purification of **A12**. Column: Inertsil ODS-4 10 × 250 mm, eluent A: MeOH + 0.05% TFA, eluent B: H_2O + 0.05% TFA, linear gradient A/B = 70/30 to 85/15 over 30 min, flow rate: 2.0 mL/min, detection: UV 220 nm.

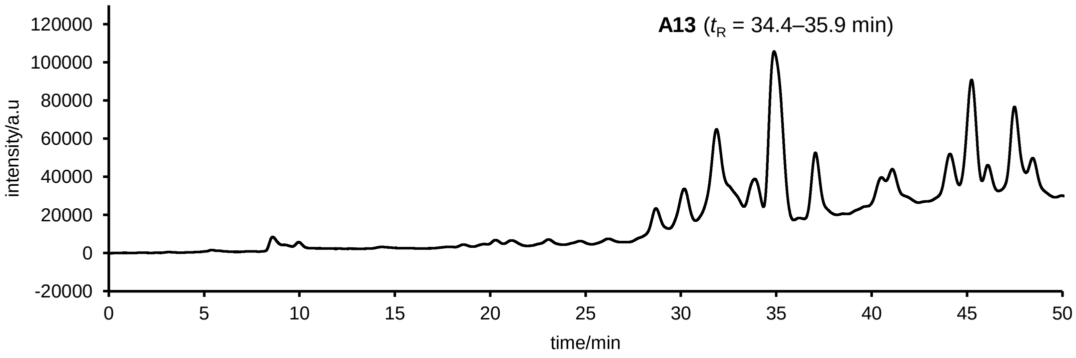

Supplementary Figure 94. HPLC chart for 1st HPLC purification for **A13**. Column: Inertsil C8-3 10 × 250 mm, eluent A: MeOH + 0.05% TFA, eluent B: H_2O + 0.05% TFA, linear gradient A/B = 55/45 to 80/20 over 50 min, flow rate: 3.0 mL/min, detection: photodiode array detector 199–651 nm (UV chromatogram: 280 nm).

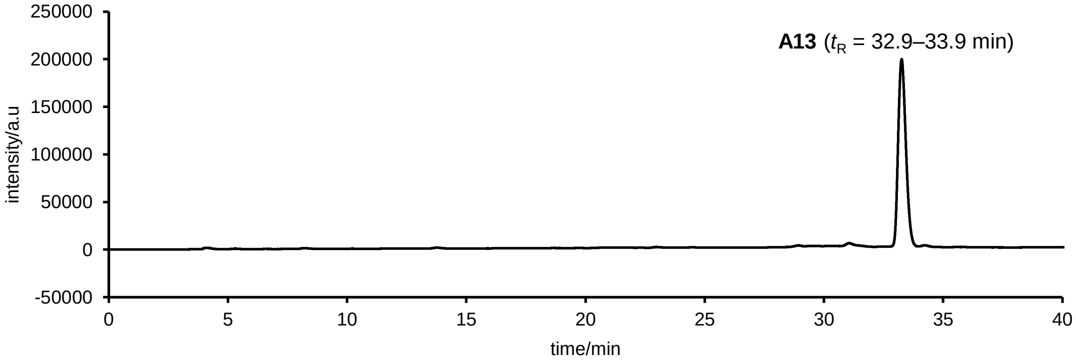

Supplementary Figure 95. HPLC chart for 2nd HPLC purification for **A13**. Column: Inertsil C8-3 10 × 250 mm, eluent A: MeOH + 0.05% TFA, eluent B: H_2O + 0.05% TFA, linear gradient A/B = 30/70 to 50/50 over 40 min, flow rate: 3.0 mL/min, detection: photodiode array detector 199–651 nm (UV chromatogram: 280 nm).

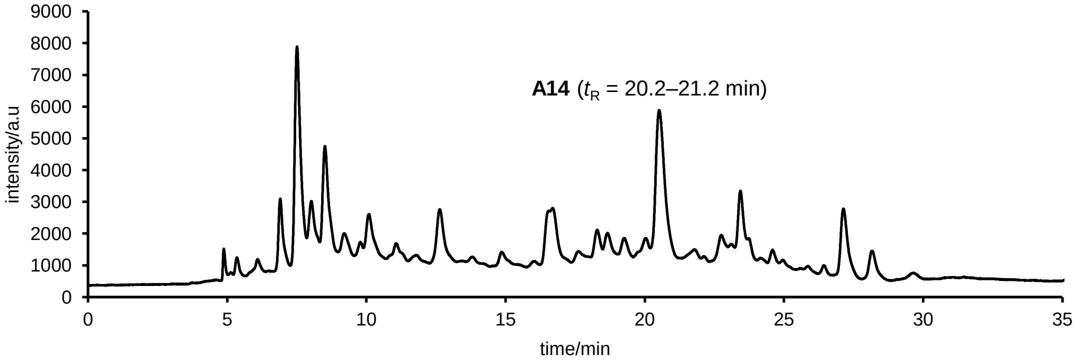

Supplementary Figure 96. HPLC chart for 1st HPLC purification of **A14**. Column: Inertsil ODS-4 10 × 250 mm, eluent A: MeCN + 0.05% TFA, eluent B: H_2O + 0.05% TFA, linear gradient A/B = 35/65 to 50/50 over 25 min, then 50/50 over 10 min, flow rate: 3.0 mL/min, detection: UV 280 nm.

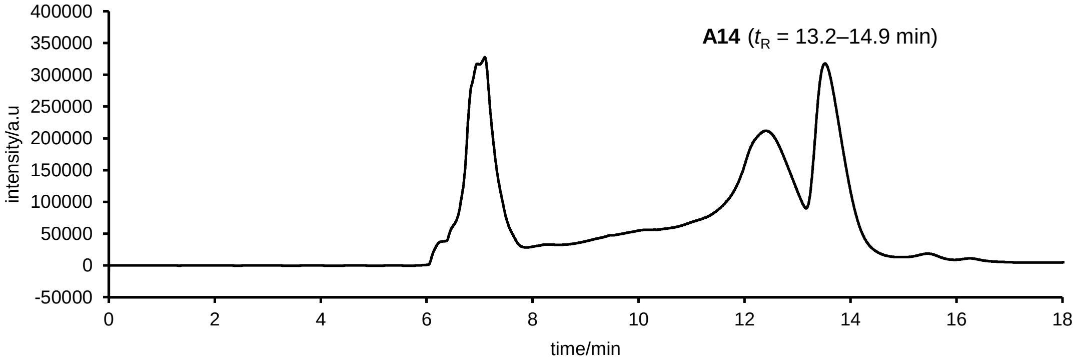

Supplementary Figure 97. HPLC chart for 2nd HPLC purification of **A14**. Column: Inertsil ODS-4 10 × 250 mm, eluent A: MeOH + 0.05% TFA, eluent B: H_2O + 0.05% TFA, linear gradient A/B = 70/30 to 79/21 over 18 min, flow rate: 2.0 mL/min, detection: UV 220 nm.

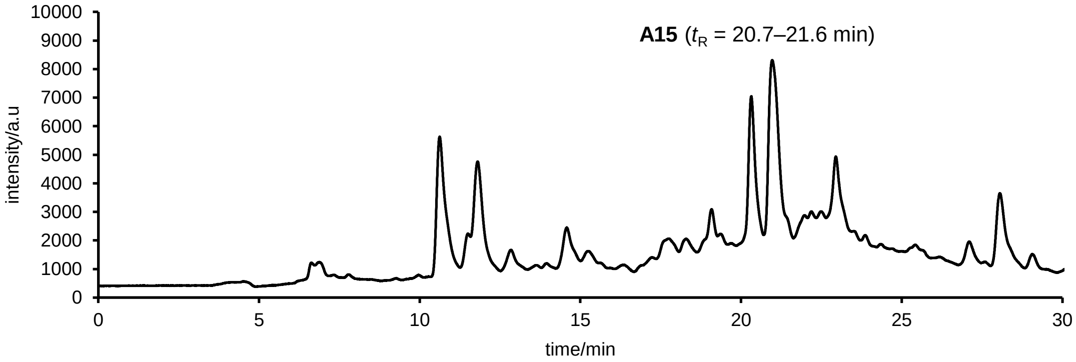

Supplementary Figure 98. HPLC chart for 1st HPLC purification of **A15**. Column: Inertsil ODS-4 10 × 250 mm, eluent A: MeCN + 0.05% TFA, eluent B: H_2O + 0.05% TFA, linear gradient A/B = 35/65 to 50/50 over 20 min, then 50/50 over 10 min, flow rate: 3.0 mL/min, detection: UV 280 nm.

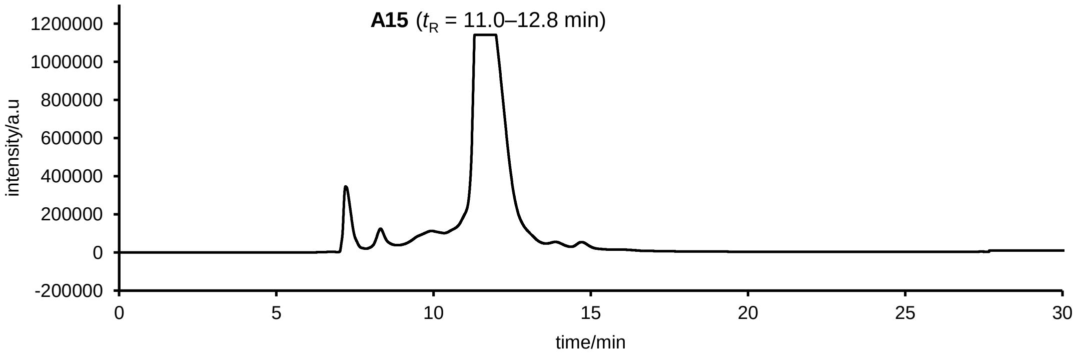

Supplementary Figure 99. HPLC chart for 2nd HPLC purification of **A15**. Column: Inertsil ODS-4 10 × 250 mm, eluent A: MeOH + 0.05% TFA, eluent B: H_2O + 0.05% TFA, linear gradient A/B = 70/30 to 85/15 over 30 min, flow rate: 2.0 mL/min, detection: UV 220 nm.

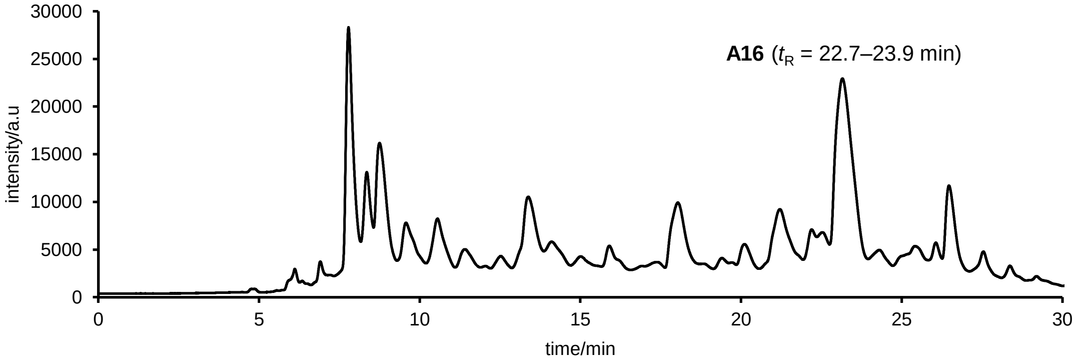

Supplementary Figure 100. HPLC chart for 1st HPLC purification of **A16**. Column: Inertsil ODS-4 10 × 250 mm, eluent A: MeCN + 0.05% TFA, eluent B: H_2O + 0.05% TFA, linear gradient A/B = 35/65 to 50/50 over 25 min, then 50/50 over 5 min, flow rate: 3.0 mL/min, detection: UV 280 nm.

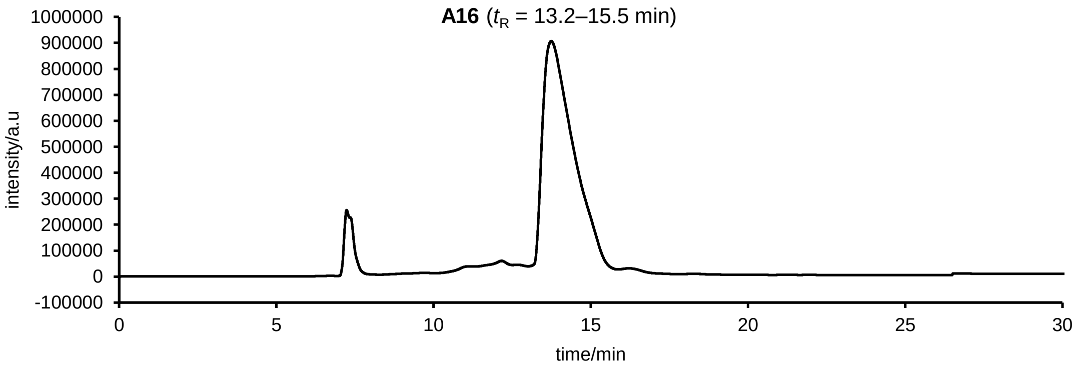

Supplementary Figure 101. HPLC chart for 2nd HPLC purification of **A16**. Column: Inertsil ODS-4 10 × 250 mm, eluent A: MeOH + 0.05% TFA, eluent B: H_2O + 0.05% TFA, linear gradient A/B = 70/30 to 85/15 over 30 min, flow rate: 2.0 mL/min, detection: UV 220 nm.

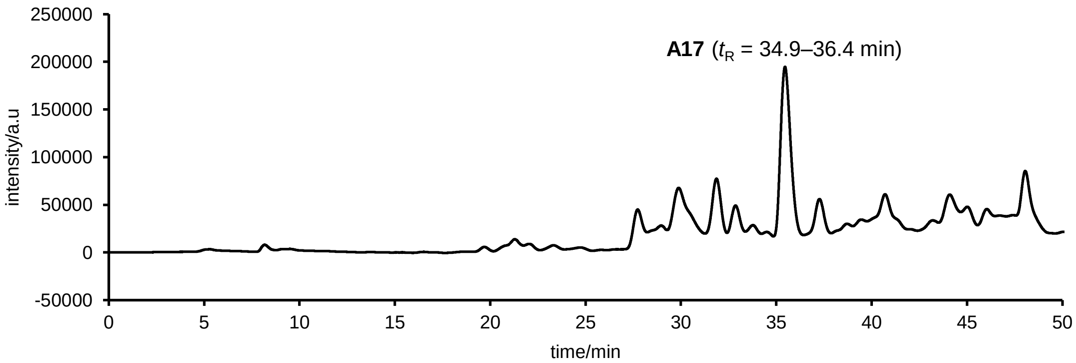

Supplementary Figure 102. HPLC chart for 1st HPLC purification for **A17**. Column: Inertsil C8-3 10 × 250 mm, eluent A: MeOH + 0.05% TFA, eluent B: H_2O + 0.05% TFA, linear gradient A/B = 55/45 to 80/20 over 50 min, flow rate: 3.0 mL/min, detection: photodiode array detector 199–651 nm (UV chromatogram: 280 nm).

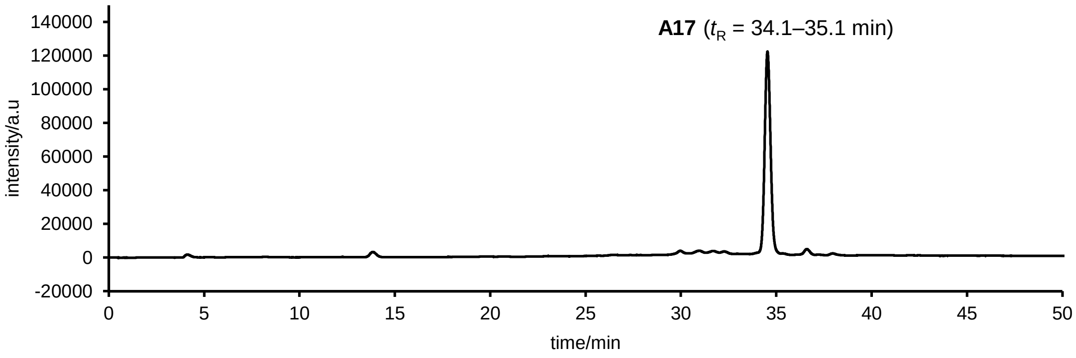

Supplementary Figure 103. HPLC chart for 2nd HPLC purification for **A17**. Column: Inertsil C8-3 10 × 250 mm, eluent A: MeOH + 0.05% TFA, eluent B: H_2O + 0.05% TFA, linear gradient A/B = 30/70 to 55/45 over 50 min, flow rate: 3.0 mL/min, detection: photodiode array detector 199–651 nm (UV chromatogram: 280 nm).

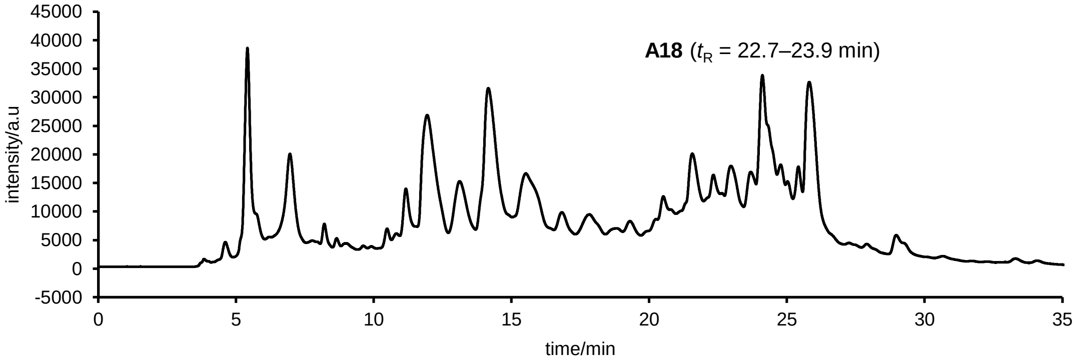

Supplementary Figure 104. HPLC chart for 1st HPLC purification of **A18**. Column: Inertsil ODS-4 4.6 × 250 mm, eluent A: MeCN + 0.05% TFA, eluent B: H_2O + 0.05% TFA, linear gradient A/B = 35/65 to 50/50 over 20 min, then 50/50 over 15 min, flow rate: 1.0 mL/min, detection: UV 280 nm.

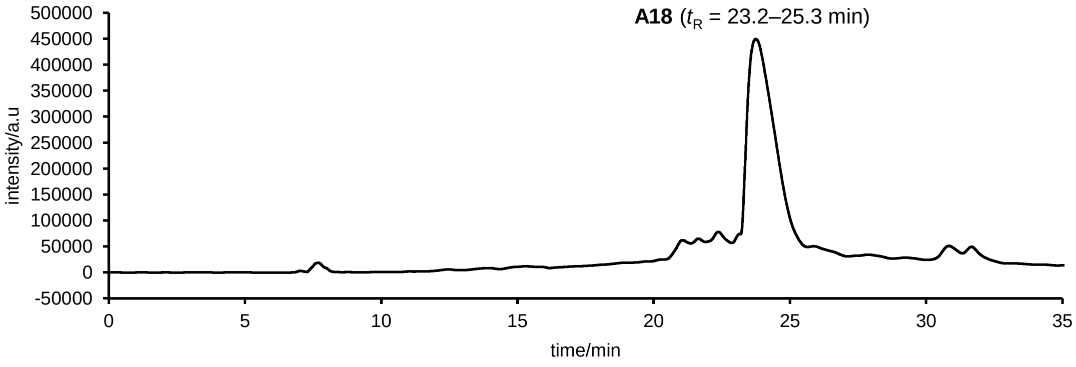

Supplementary Figure 105. HPLC chart for 2nd HPLC purification of **A18**. Column: Inertsil ODS-4 10 × 250 mm, eluent A: MeOH + 0.05% TFA, eluent B: H_2O + 0.05% TFA, linear gradient A/B = 70/30 to 87.5/12.5 over 35 min, flow rate: 2.0 mL/min, detection: UV 220 nm.

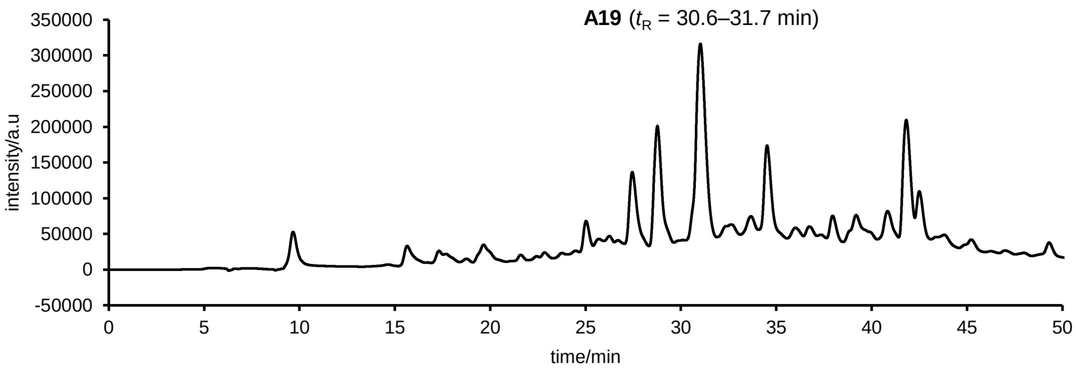

Supplementary Figure 106. HPLC chart for 1st HPLC purification of **A19**. Column: Inertsil C8-3 10 × 250 mm, eluent A: MeCN + 0.05% TFA, eluent B: H_2O + 0.05% TFA, linear gradient A/B = 32.5/67.5 to 57.5/42.5 over 50 min, flow rate: 3.0 mL/min, detection: photodiode array detector 199–651 nm (UV chromatogram: 280 nm).

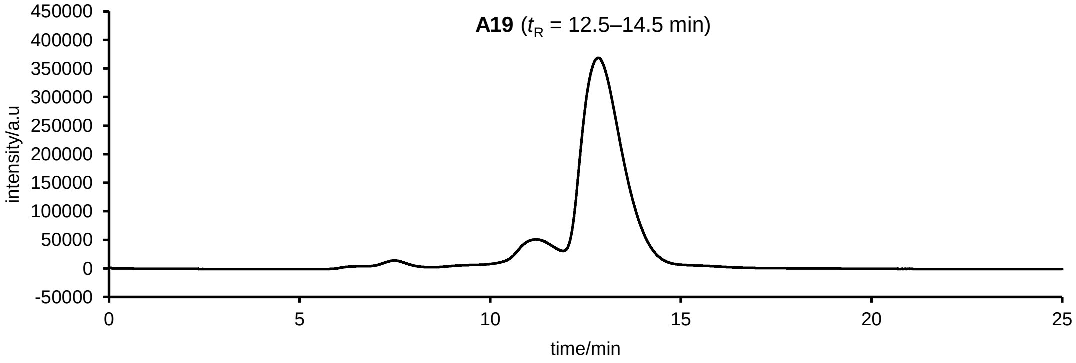

Supplementary Figure 107. HPLC chart for 2nd HPLC purification of **A19**. Column: Inertsil C8-3 10 × 250 mm, eluent A: MeOH + 0.05% TFA, eluent B: H_2O + 0.05% TFA, A/B = 67.5/32.5 over 25 min, flow rate: 3.0 mL/min, detection: photodiode array detector 199–651 nm (UV chromatogram: 280 nm).

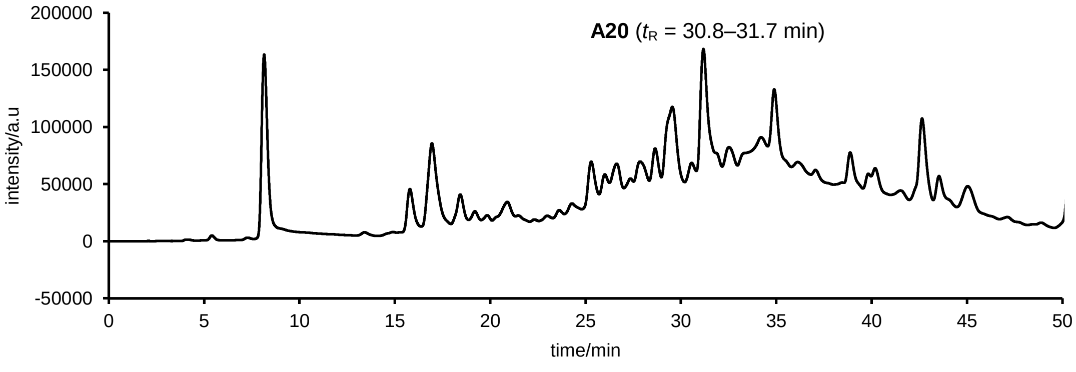

Supplementary Figure 108. HPLC chart for 1st HPLC purification of **A20**. Column: Inertsil C8-3 10 × 250 mm, eluent A: MeCN + 0.05% TFA, eluent B: H_2O + 0.05% TFA, linear gradient A/B = 30/70 to 55/45 over 50 min, flow rate: 3.0 mL/min, detection: photodiode array detector 199–651 nm (UV chromatogram: 280 nm).

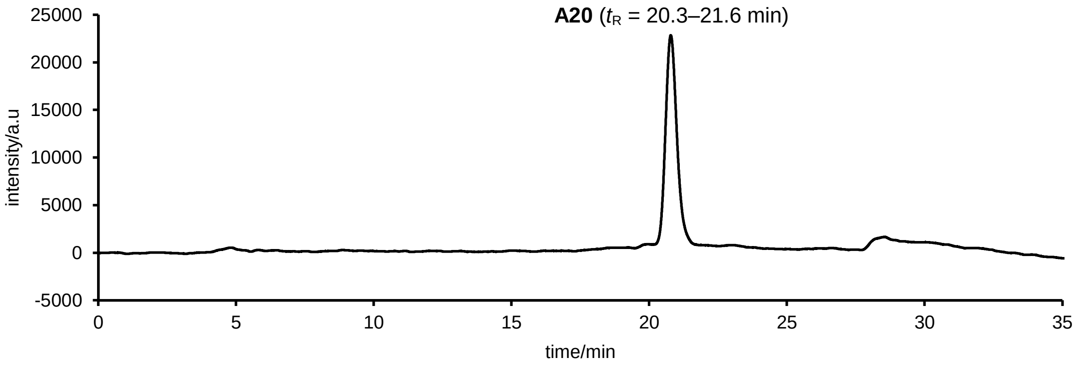

Supplementary Figure 109. HPLC chart for 2nd HPLC purification of **A20**. Column: Inertsil C8-3 10 × 250 mm, eluent A: MeOH + 0.05% TFA, eluent B: H_2O + 0.05% TFA, linear gradient A/B = 60/40 to 80/20 over 35 min, flow rate: 3.0 mL/min, detection: photodiode array detector 199–651 nm (UV chromatogram: 280 nm).

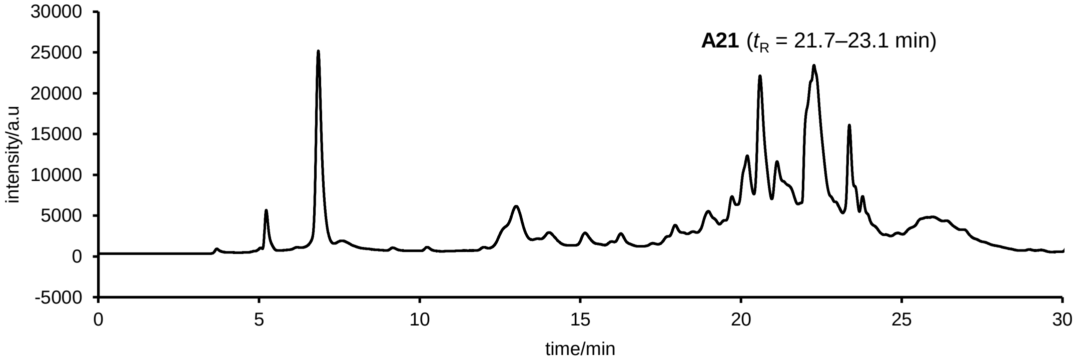

Supplementary Figure 110. HPLC chart for 1st HPLC purification of **A21**. Column: Inertsil ODS-4 10 × 250 mm, eluent A: MeCN + 0.05% TFA, eluent B: H_2O + 0.05% TFA, A/B = 35/65 over 5 min, linear gradient 35/65 to 60/40 over 25 min, flow rate: 3.0 mL/min, detection: UV 280 nm.

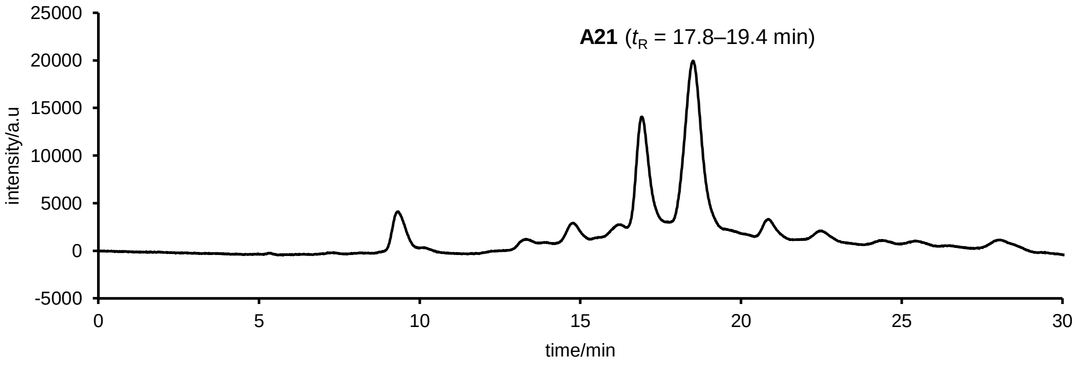

Supplementary Figure 111. HPLC chart for 2nd HPLC purification of **A21**. Column: Inertsil ODS-4 10 × 250 mm, eluent A: MeOH + 0.05% TFA, eluent B: H_2O + 0.05% TFA, linear gradient A/B = 65/35 to 70/30 over 25 min, then 70/30 over 5 min, flow rate: 3.0 mL/min, detection: photodiode array detector 199–651 nm (UV chromatogram: 280 nm).

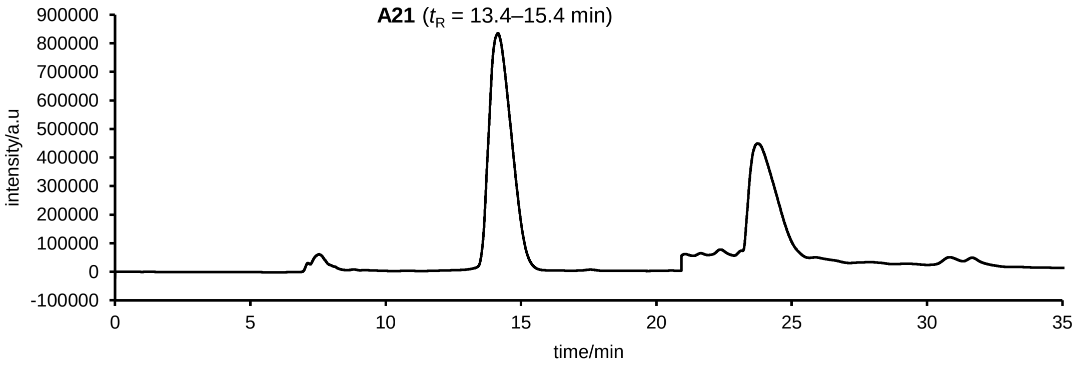

Supplementary Figure 112. HPLC chart for 3rd HPLC purification of **A21**. Column: Inertsil ODS-4 10 × 250 mm, eluent A: MeOH + 0.05% TFA, eluent B: H_2O + 0.05% TFA, linear gradient A/B = 70/30 to 87.5/12.5 over 35 min min, flow rate: 2.0 mL/min, detection: UV 220 nm.

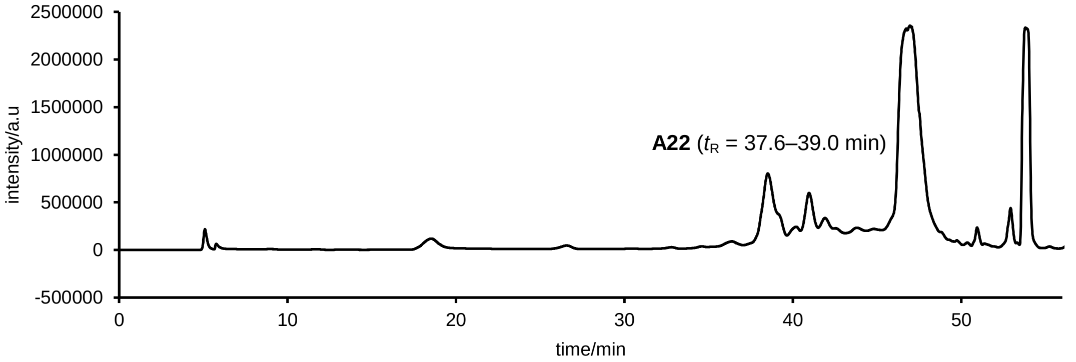

Supplementary Figure 113. HPLC chart for 1st HPLC purification of **A22**. Column: Inertsil C8-3 10 × 250 mm, eluent A: MeOH + 0.05% TFA, eluent B: H_2O + 0.05% TFA, linear gradient A/B = 24/76 to 73/27 over 56 min, flow rate: 3.0 mL/min, detection: photodiode array detector 199–651 nm (UV chromatogram: 280 nm).

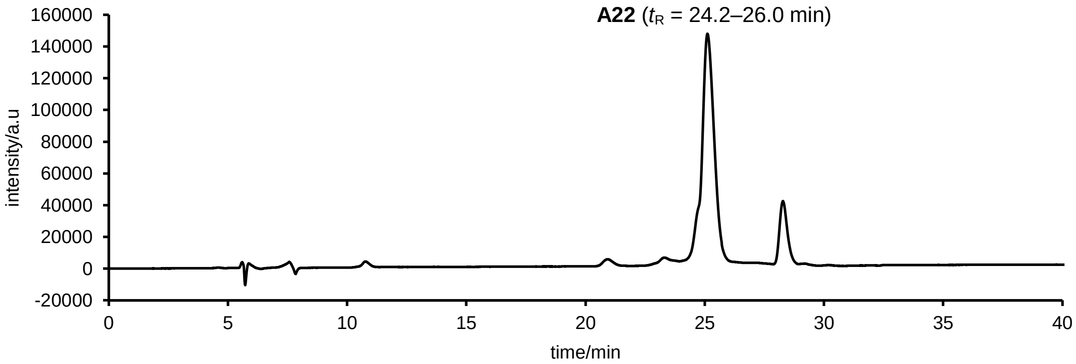

Supplementary Figure 114. HPLC chart for 2nd HPLC purification of **A22**. Column: Inertsil C8-3 10 × 250 mm, eluent A: MeCN + 0.05% TFA, eluent B: H_2O + 0.05% TFA, linear gradient A/B = 20/80 to 35/65 over 40 min, flow rate: 3.0 mL/min, detection: photodiode array detector 199–651 nm (UV chromatogram: 280 nm).

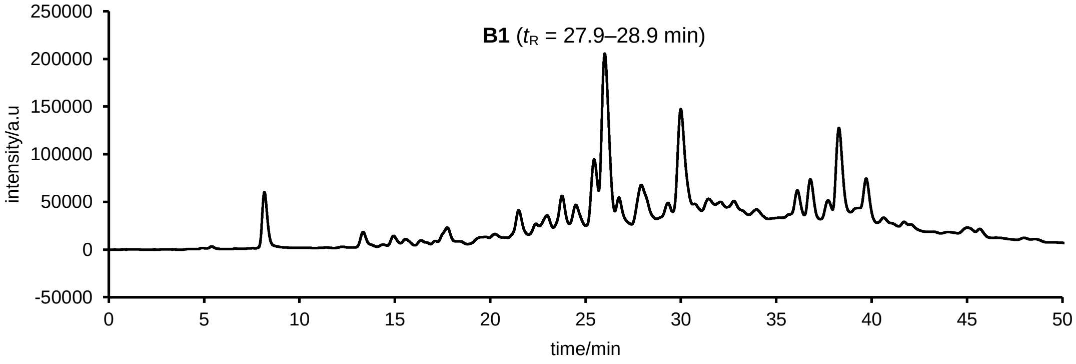

Supplementary Figure 115. HPLC chart for 1st HPLC purification of **B1**. Column: Inertsil C8-3 10 × 250 mm, eluent A: MeCN + 0.05% TFA, eluent B: H_2O + 0.05% TFA, linear gradient A/B = 30/70 to 55/45 over 50 min, flow rate: 3.0 mL/min, detection: photodiode array detector 199–651 nm (UV chromatogram: 280 nm).

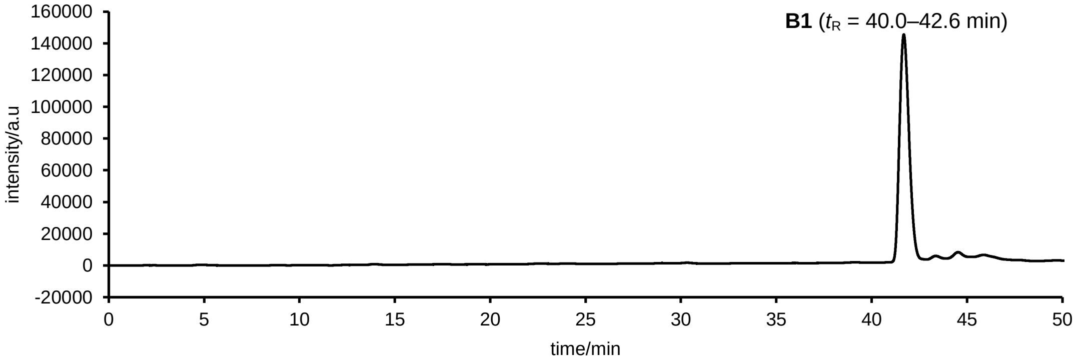

Supplementary Figure 116. HPLC chart for 2nd HPLC purification of **B1**. Column: Inertsil C8-3 10 × 250 mm, eluent A: MeOH + 0.05% TFA, eluent B: H_2O + 0.05% TFA, linear gradient A/B = 45/55 to 70/30 over 50 min, flow rate: 3.0 mL/min, detection: photodiode array detector 199–651 nm (UV chromatogram: 280 nm).

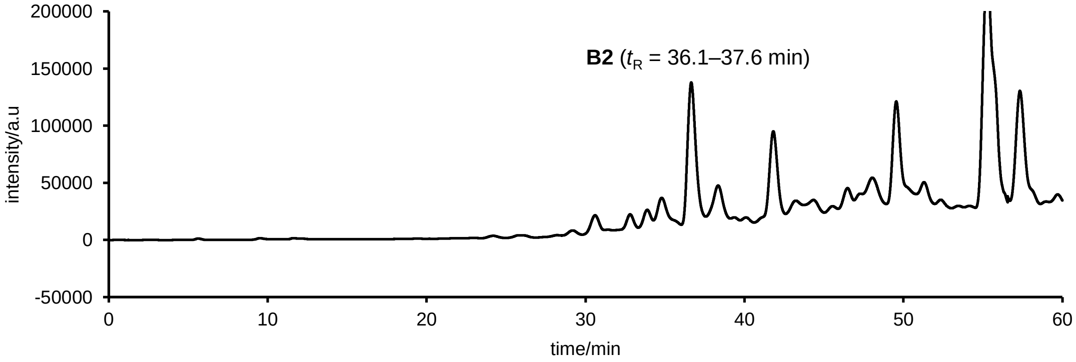

Supplementary Figure 117. HPLC chart for 1st HPLC purification for **B2**. Column: Inertsil C8-3 10 × 250 mm, eluent A: MeOH + 0.05% TFA, eluent B: H_2O + 0.05% TFA, linear gradient A/B = 40/60 to 70/30 over 60 min, flow rate: 3.0 mL/min, detection: photodiode array detector 199–651 nm (UV chromatogram: 280 nm).

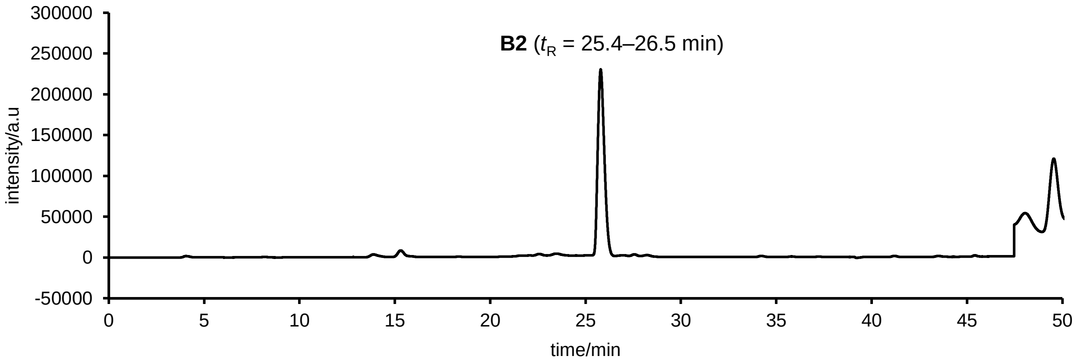

Supplementary Figure 118. HPLC chart for 2nd HPLC purification for **B2**. Column: Inertsil C8-3 10 × 250 mm, eluent A: MeOH + 0.05% TFA, eluent B: H_2O + 0.05% TFA, linear gradient A/B = 25/75 to 50/50 over 50 min, flow rate: 3.0 mL/min, detection: photodiode array detector 199–651 nm (UV chromatogram: 280 nm).

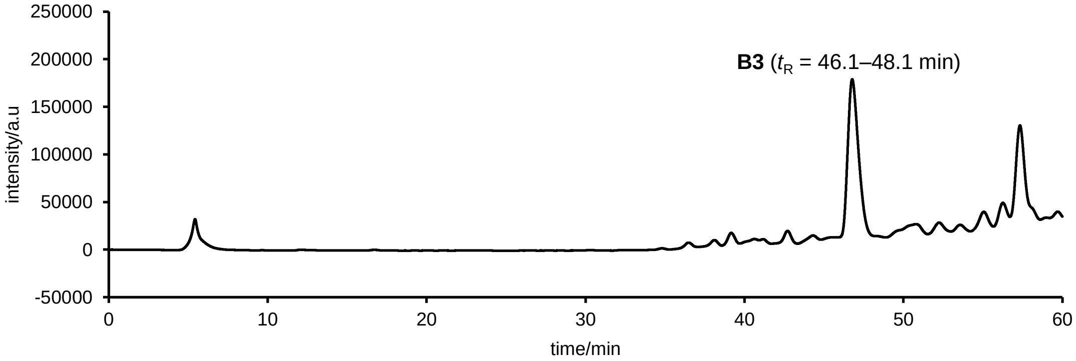

Supplementary Figure 119. HPLC chart for 1st HPLC purification for **B3**. Column: Inertsil C8-3 10 × 250 mm, eluent A: MeOH + 0.05% TFA, eluent B: H_2O + 0.05% TFA, linear gradient A/B = 40/60 to 70/30 over 60 min, flow rate: 3.0 mL/min, detection: photodiode array detector 199–651 nm (UV chromatogram: 280 nm).

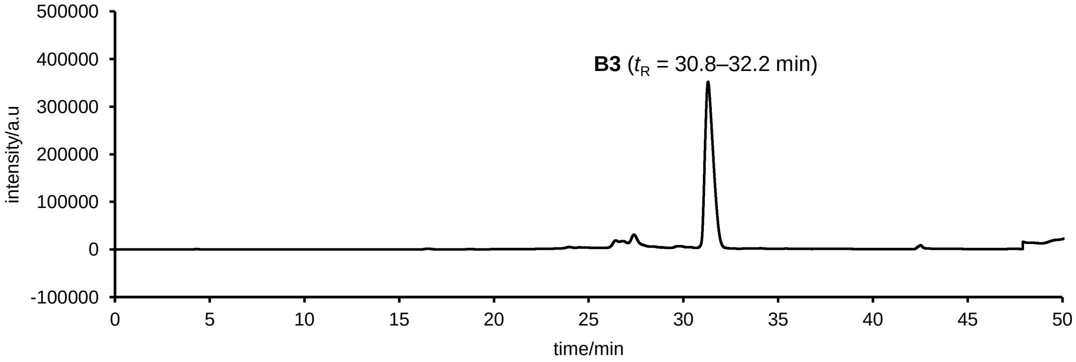

Supplementary Figure 120. HPLC chart for 2nd HPLC purification for **B3**. Column: Inertsil C8-3 10 × 250 mm, eluent A: MeCN + 0.05% TFA, eluent B: H_2O + 0.05% TFA, linear gradient A/B = 25/75 to 50/50 over 50 min, flow rate: 3.0 mL/min, detection: photodiode array detector 199–651 nm (UV chromatogram: 280 nm).

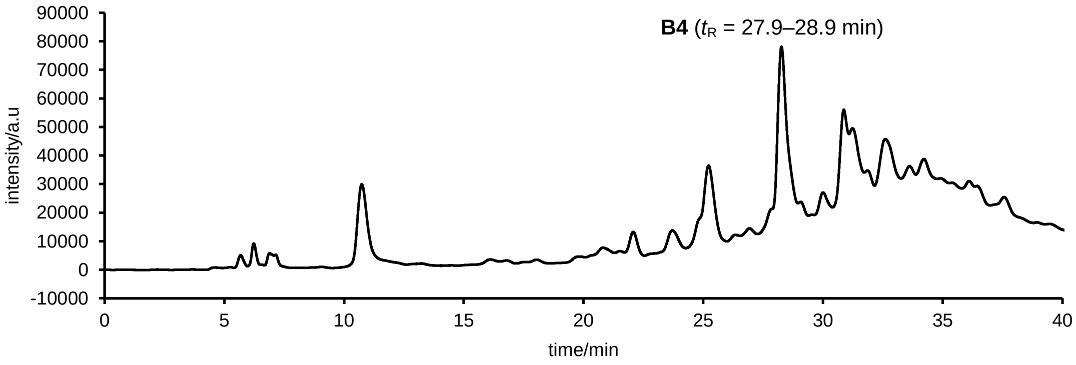

Supplementary Figure 121. HPLC chart for 1st HPLC purification of **B4**. Column: Inertsil C8-3 10 × 250 mm, eluent A: MeCN + 0.05% TFA, eluent B: H_2O + 0.05% TFA, linear gradient A/B = 25/75 to 35/65 over 40 min, flow rate: 3.0 mL/min, detection: photodiode array detector 199–651 nm (UV chromatogram: 280 nm).

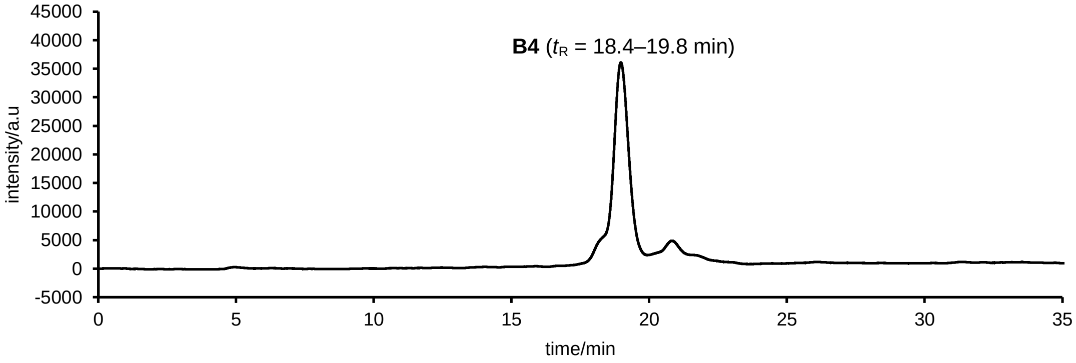

Supplementary Figure 122. HPLC chart for 2nd HPLC purification of **B4**. Column: Inertsil C8-3 10 × 250 mm, eluent A: MeOH + 0.05% TFA, eluent B: H_2O + 0.05% TFA, linear gradient A/B = 45/55 to 62.5/57.5 over 50 min, flow rate: 3.0 mL/min, detection: photodiode array detector 199–651 nm (UV chromatogram: 280 nm).

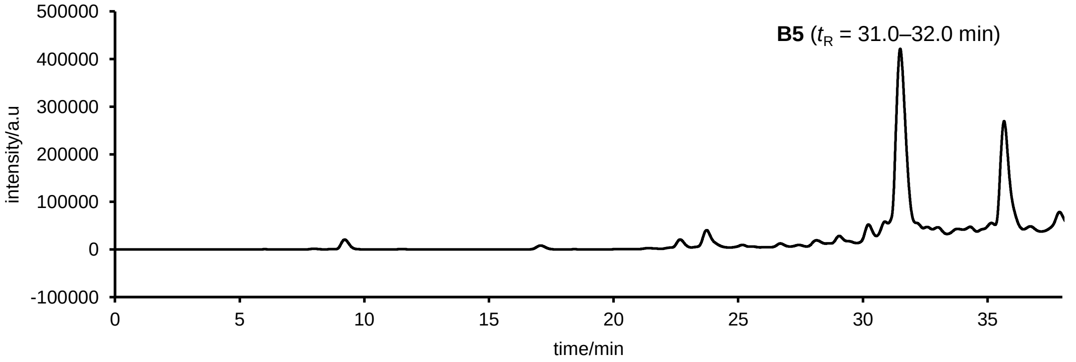

Supplementary Figure 123. HPLC chart for 1st HPLC purification for **B5**. Column: Inertsil C8-3 10 × 250 mm, eluent A: MeCN + 0.05% TFA, eluent B: H_2O + 0.05% TFA, linear gradient A/B = 25/75 to 44/56 over 38 min, flow rate: 3.0 mL/min, detection: photodiode array detector 199–651 nm (UV chromatogram: 280 nm).

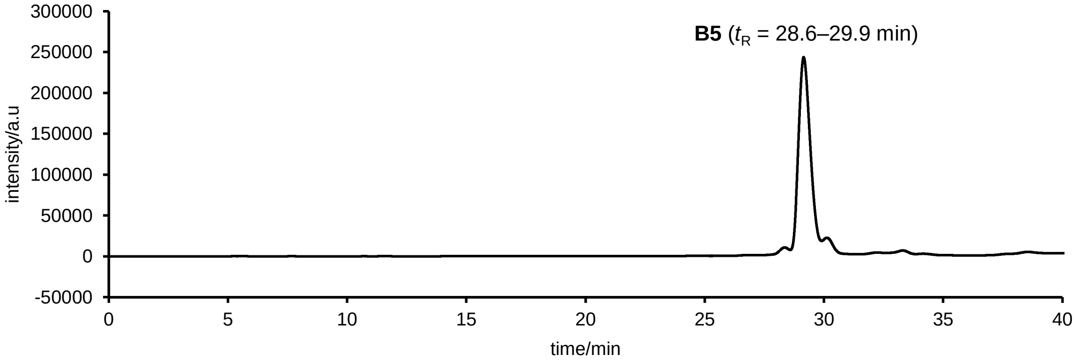

Supplementary Figure 124. HPLC chart for 2nd HPLC purification of **B5**. Column: Inertsil C8-3 10 × 250 mm, eluent A: MeOH + 0.05% TFA, eluent B: H_2O + 0.05% TFA, linear gradient A/B = 50/50 to 70/30 over 40 min, flow rate: 3.0 mL/min, detection: photodiode array detector 199–651 nm (UV chromatogram: 280 nm).

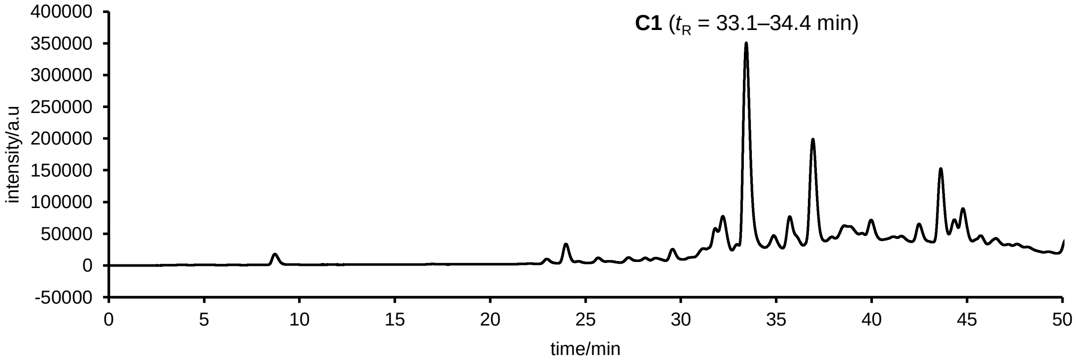

Supplementary Figure 125. HPLC chart for 1st HPLC purification of **C1**. Column: Inertsil C8-3 10 × 250 mm, eluent A: MeCN + 0.05% TFA, eluent B: H_2O + 0.05% TFA, linear gradient A/B = 25/75 to 50/50 over 50 min, flow rate: 3.0 mL/min, detection: photodiode array detector 199–651 nm (UV chromatogram: 280 nm).

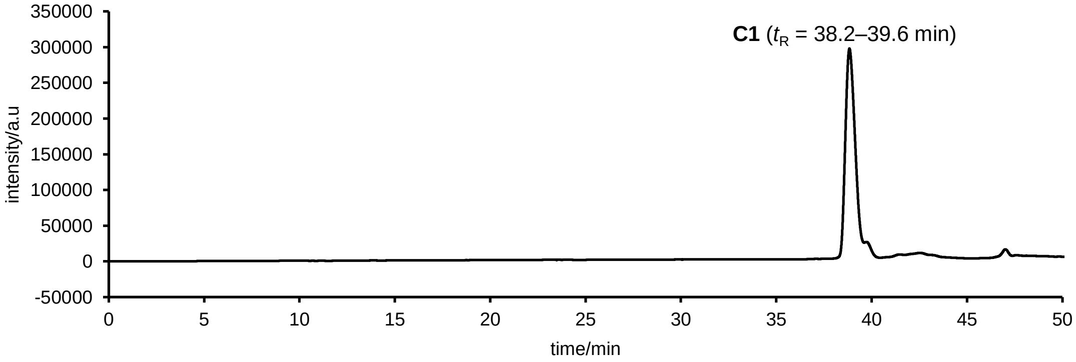

Supplementary Figure 126. HPLC chart for 2nd HPLC purification of **C1**. Column: Inertsil C8-3 10 × 250 mm, eluent A: MeOH + 0.05% TFA, eluent B: H_2O + 0.05% TFA, linear gradient A/B = 45/55 to 70/30 over 50 min, flow rate: 3.0 mL/min, detection: photodiode array detector 199–651 nm (UV chromatogram: 280 nm).

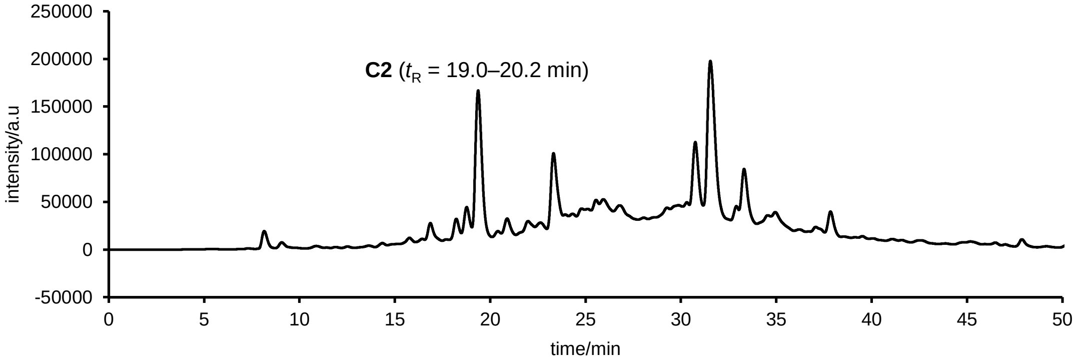

Supplementary Figure 127. HPLC chart for 1st HPLC purification of **C2**. Column: Inertsil C8-3 10 × 250 mm, eluent A: MeCN + 0.05% TFA, eluent B: H_2O + 0.05% TFA, linear gradient A/B = 30/70 to 55/45 over 50 min, flow rate: 3.0 mL/min, detection: photodiode array detector 199–651 nm (UV chromatogram: 280 nm).

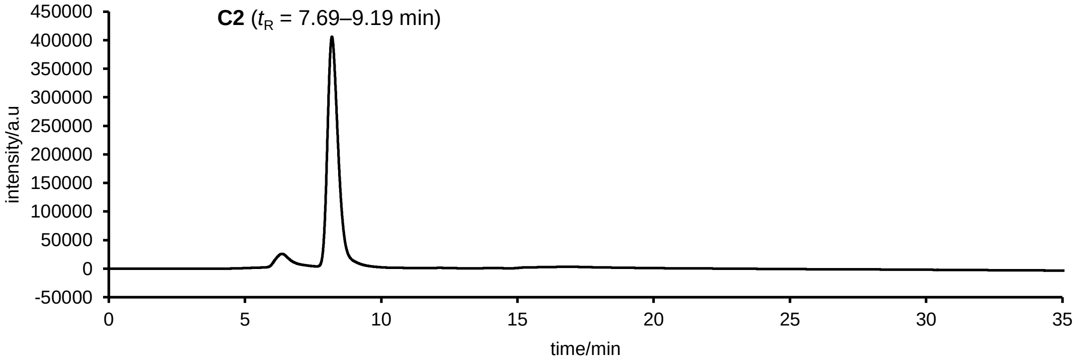

Supplementary Figure 128. HPLC chart for 2nd HPLC purification of **C2**. Column: Inertsil C8-3 10 × 250 mm, eluent A: MeOH + 0.05% TFA, eluent B: H_2O + 0.05% TFA, linear gradient A/B = 60/40 to 80/20 over 35 min, flow rate: 3.0 mL/min, detection: photodiode array detector 199–651 nm (UV chromatogram: 280 nm).

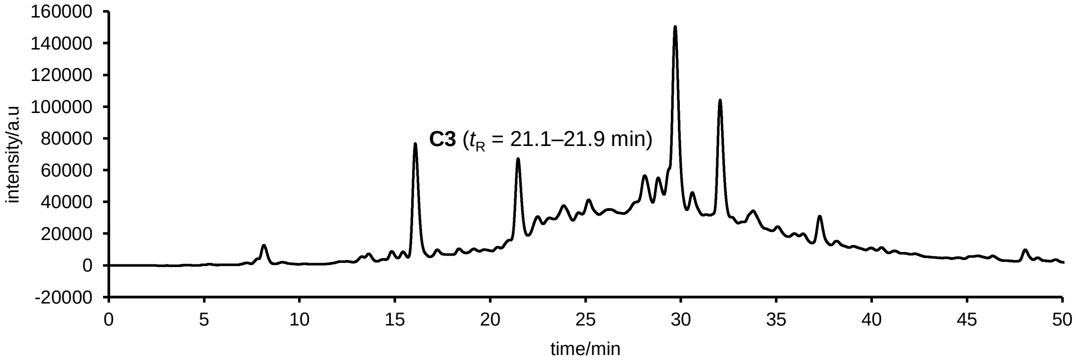

Supplementary Figure 129. HPLC chart for 1st HPLC purification of **C3**. Column: Inertsil C8-3 10 × 250 mm, eluent A: MeCN + 0.05% TFA, eluent B: H_2O + 0.05% TFA, linear gradient A/B = 30/70 to 55/45 over 50 min, flow rate: 3.0 mL/min, detection: photodiode array detector 199–651 nm (UV chromatogram: 280 nm).

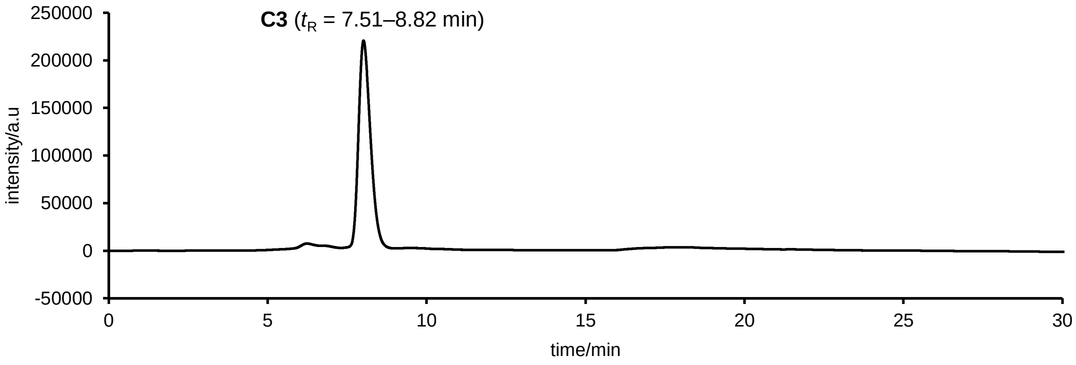

Supplementary Figure 130. HPLC chart for 2nd HPLC purification of **C3**. Column: Inertsil C8-3 10 × 250 mm, eluent A: MeOH + 0.05% TFA, eluent B: H_2O + 0.05% TFA, linear gradient A/B = 60/40 to 73.3/26.7 over 30 min, flow rate: 3.0 mL/min, detection: photodiode array detector 199–651 nm (UV chromatogram: 280 nm).

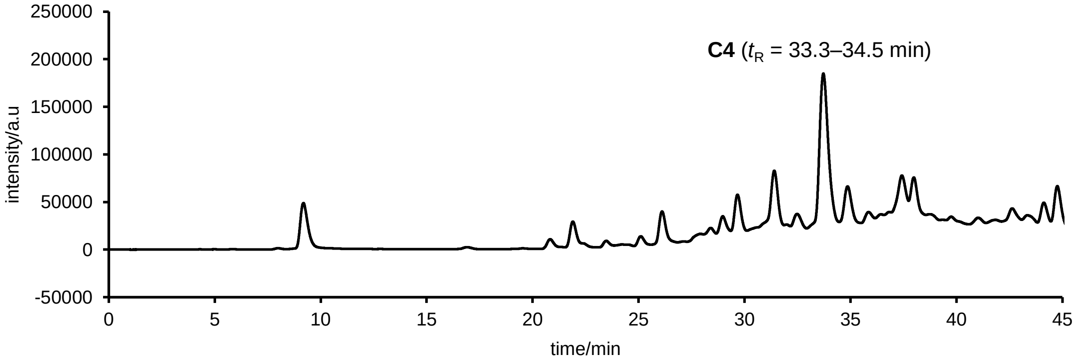

Supplementary Figure 131. HPLC chart for 1st HPLC purification of **C4**. Column: Inertsil C8-3 10 × 250 mm, eluent A: MeCN + 0.05% TFA, eluent B: H_2O + 0.05% TFA, linear gradient A/B = 25/75 to 47.5/52.5 over 45 min, flow rate: 3.0 mL/min, detection: photodiode array detector 199–651 nm (UV chromatogram: 280 nm).

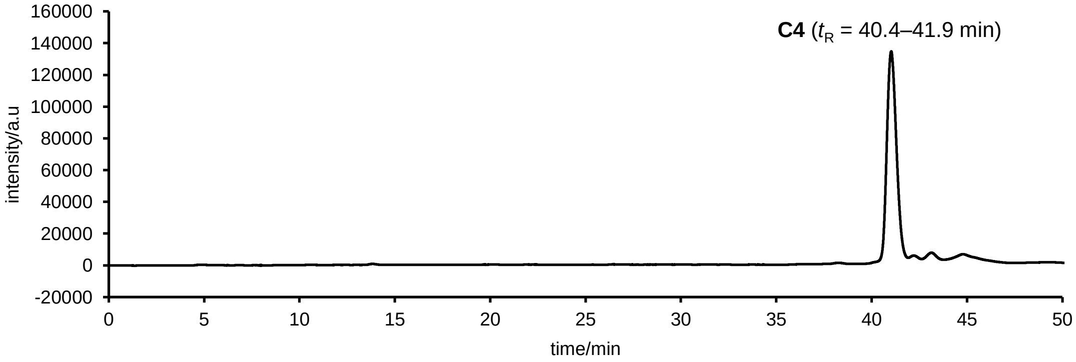

Supplementary Figure 132. HPLC chart for 2nd HPLC purification of **C4**. Column: Inertsil C8-3 10 × 250 mm, eluent A: MeOH + 0.05% TFA, eluent B: H_2O + 0.05% TFA, linear gradient A/B = 45/55 to 70/30 over 50 min, flow rate: 3.0 mL/min, detection: photodiode array detector 199–651 nm (UV chromatogram: 280 nm).

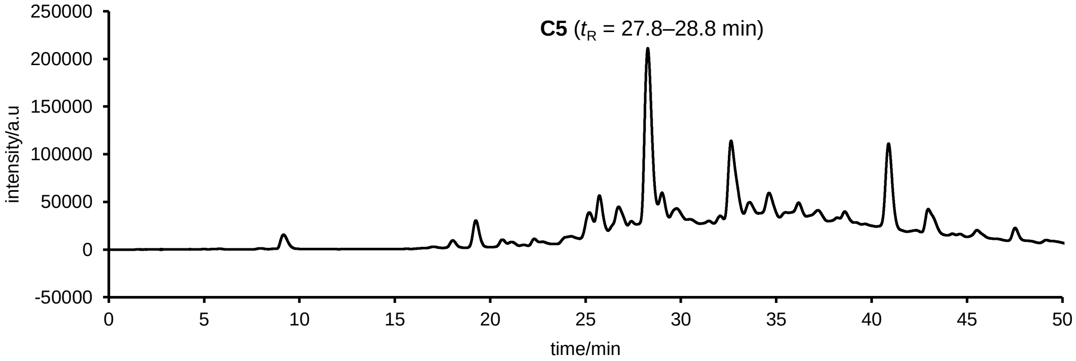

Supplementary Figure 133. HPLC chart for 1st HPLC purification of **C5**. Column: Inertsil C8-3 10 × 250 mm, eluent A: MeCN + 0.05% TFA, eluent B: H_2O + 0.05% TFA, linear gradient A/B = 25/75 to 50/50 over 50 min, flow rate: 3.0 mL/min, detection: photodiode array detector 199–651 nm (UV chromatogram: 280 nm).

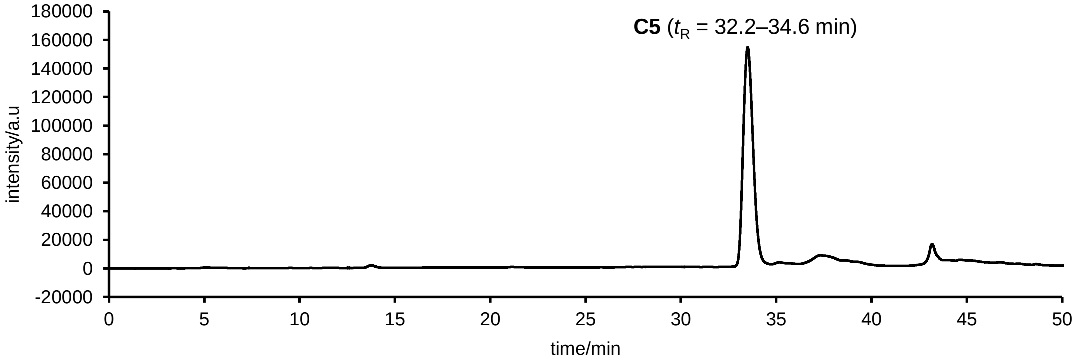

Supplementary Figure 134. HPLC chart for 2nd HPLC purification of **C5**. Column: Inertsil C8-3 10 × 250 mm, eluent A: MeOH + 0.05% TFA, eluent B: H_2O + 0.05% TFA, linear gradient A/B = 45/55 to 70/30 over 50 min, flow rate: 3.0 mL/min, detection: photodiode array detector 199–651 nm (UV chromatogram: 280 nm).

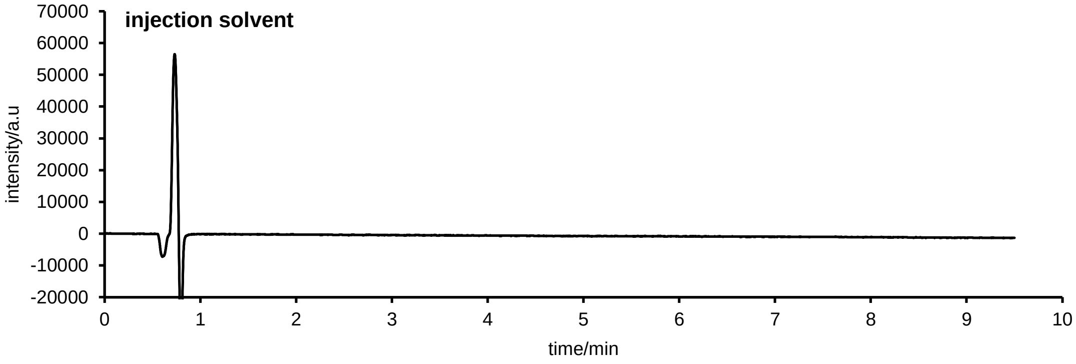

Supplementary Figure 135. UHPLC chart of the injection solvent. Column: Accucore C18 2.1 ×150 mm, eluent A: MeCN + 0.05% TFA, eluent B: H_2O + 0.05% TFA, A/B = 40/60, flow rate: 0.40 mL/min, detection: photodiode array detector 200–648 nm (UV chromatogram: 280 nm), temperature: 40 ºC.

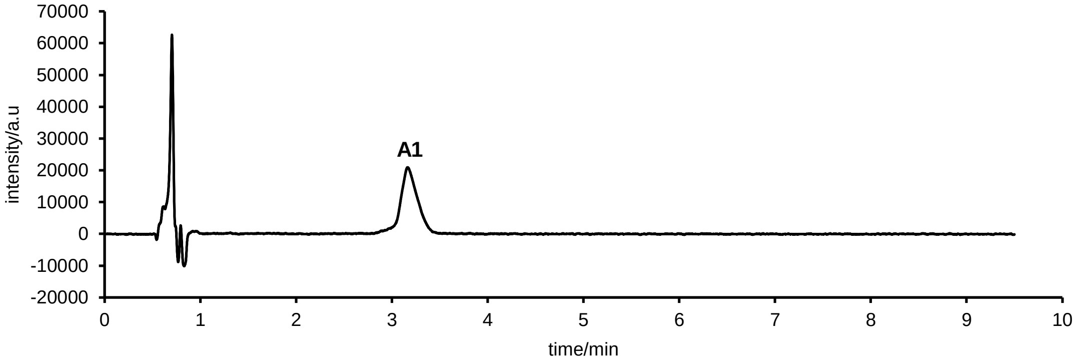

Supplementary Figure 136. UHPLC chart of purified **A1**. Column: Accucore C18 2.1 ×150 mm, eluent A: MeCN + 0.05% TFA, eluent B: H_2O + 0.05% TFA, A/B = 40/60, flow rate: 0.40 mL/min, detection: photodiode array detector 200–648 nm (UV chromatogram: 280 nm), temperature: 40 ºC.

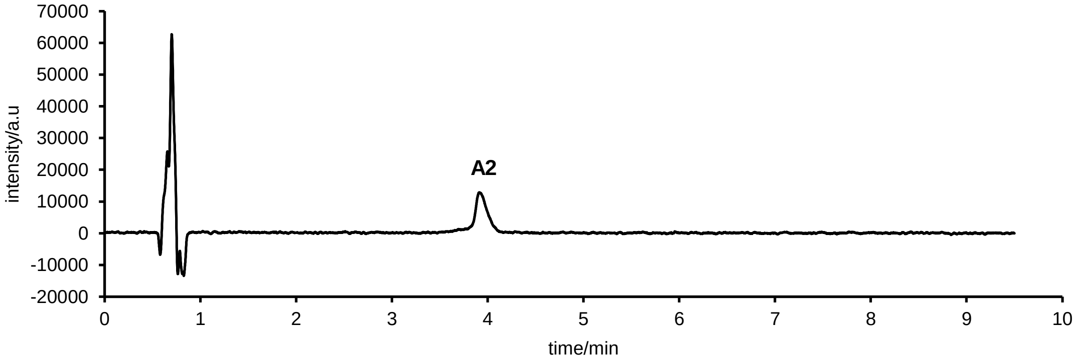

Supplementary Figure 137. UHPLC chart of purified **A2**. Column: Accucore C18 2.1 ×150 mm, eluent A: MeCN + 0.05% TFA, eluent B: H_2O + 0.05% TFA, A/B = 40/60, flow rate: 0.40 mL/min, detection: photodiode array detector 200–648 nm (UV chromatogram: 280 nm), temperature: 40 ºC.

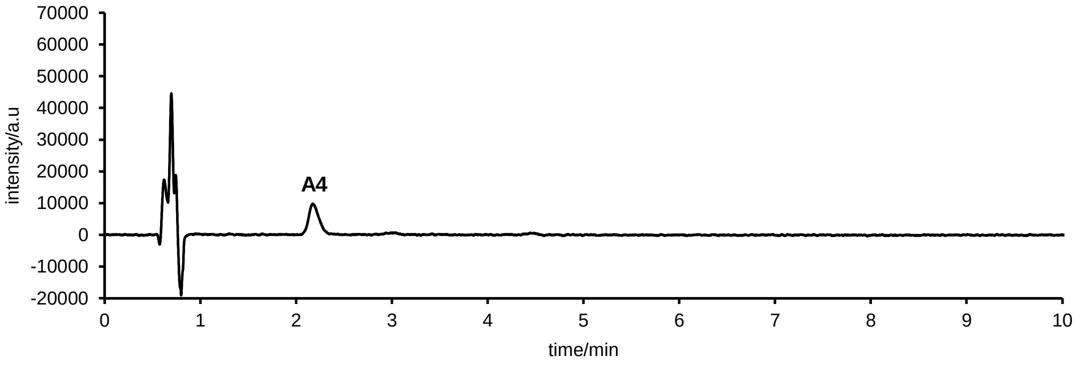

Supplementary Figure 138. UHPLC chart of purified **A4**. Column: Accucore C18 2.1 ×150 mm, eluent A: MeCN + 0.05% TFA, eluent B: H_2O + 0.05% TFA, A/B = 40/60, flow rate: 0.40 mL/min, detection: photodiode array detector 200–648 nm (UV chromatogram: 280 nm), temperature: 40 ºC.

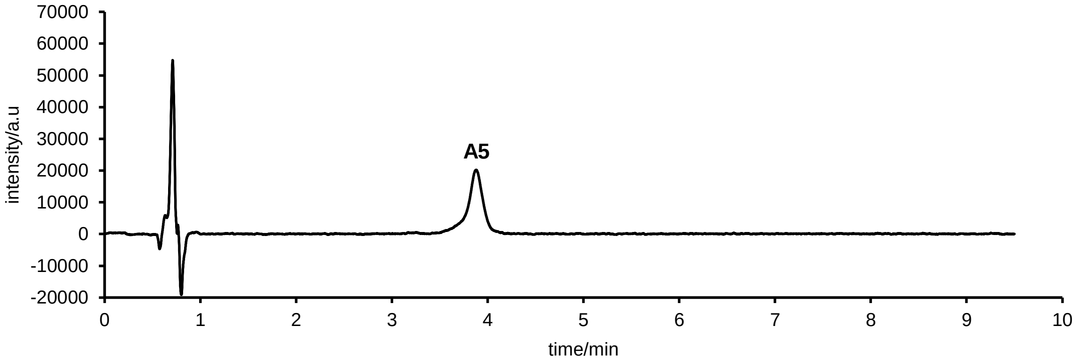

Supplementary Figure 139. UHPLC chart of purified **A5**. Column: Accucore C18 2.1 ×150 mm, eluent A: MeCN + 0.05% TFA, eluent B: H_2O + 0.05% TFA, A/B = 40/60, flow rate: 0.40 mL/min, detection: photodiode array detector 200–648 nm (UV chromatogram: 280 nm), temperature: 40 ºC.

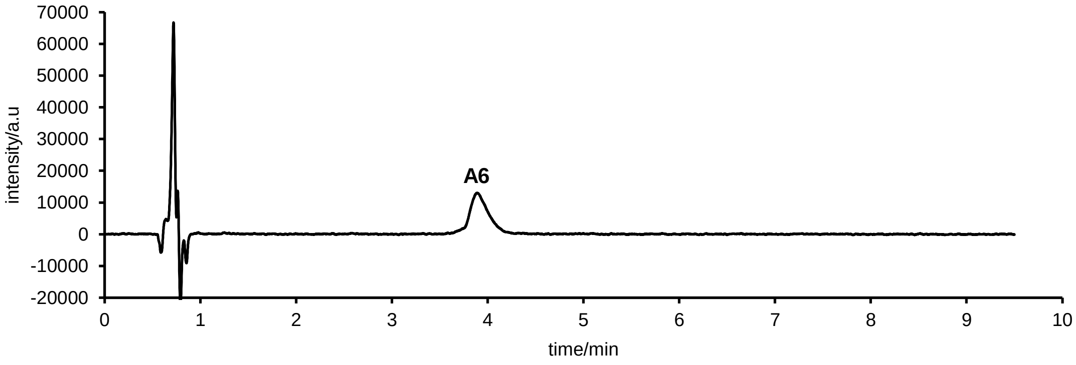

Supplementary Figure 140. UHPLC chart of purified **A6**. Column: Accucore C18 2.1 ×150 mm, eluent A: MeCN + 0.05% TFA, eluent B: H_2O + 0.05% TFA, A/B = 40/60, flow rate: 0.40 mL/min, detection: photodiode array detector 200–648 nm (UV chromatogram: 280 nm), temperature: 40 ºC.

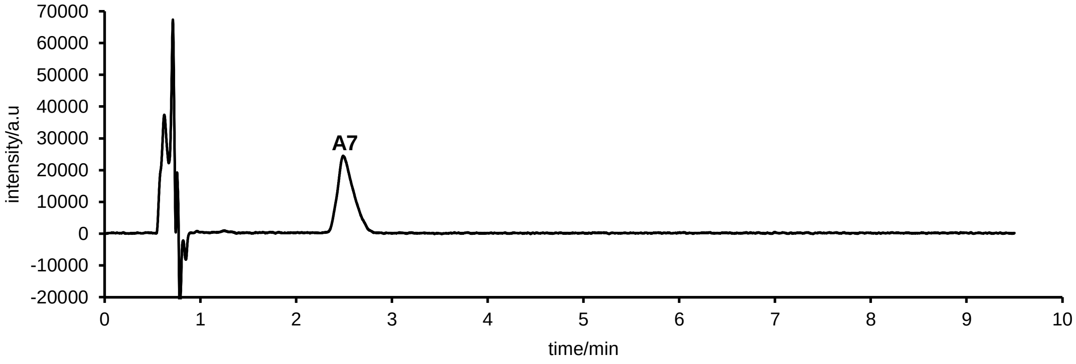

Supplementary Figure 141. UHPLC chart of purified **A7**. Column: Accucore C18 2.1 ×150 mm, eluent A: MeCN + 0.05% TFA, eluent B: H_2O + 0.05% TFA, A/B = 40/60, flow rate: 0.40 mL/min, detection: photodiode array detector 200–648 nm (UV chromatogram: 280 nm), temperature: 40 ºC.

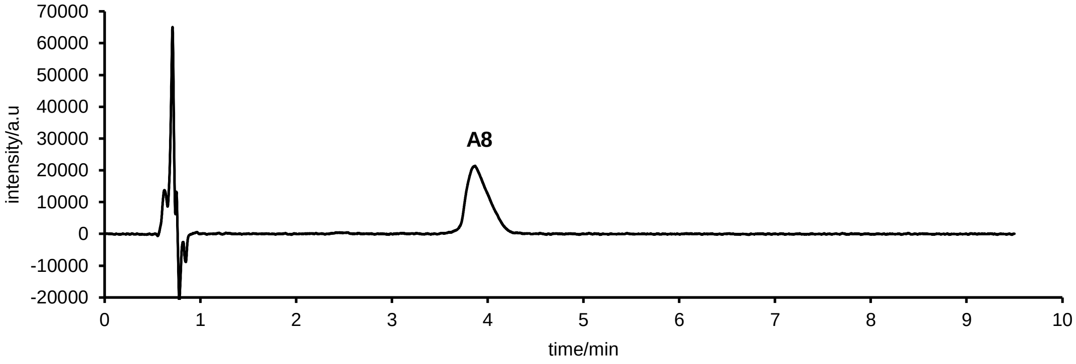

Supplementary Figure 142. UHPLC chart of the purified **A8**. Column: Accucore C18 2.1 ×150 mm, eluent A: MeCN + 0.05% TFA, eluent B: H_2O + 0.05% TFA, A/B = 40/60, flow rate: 0.40 mL/min, detection: photodiode array detector 200–648 nm (UV chromatogram: 280 nm), temperature: 40 ºC.

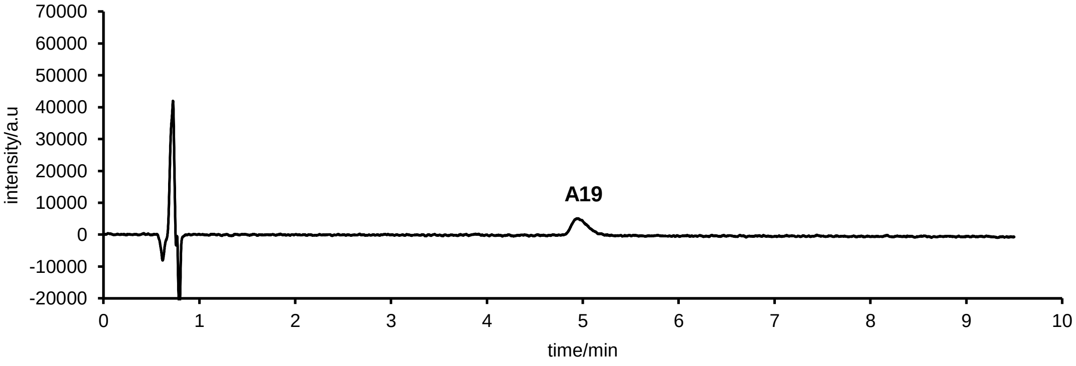

Supplementary Figure 143. UHPLC chart of purified **A19**. Column: Accucore C18 2.1 ×150 mm, eluent A: MeCN + 0.05% TFA, eluent B: H_2O + 0.05% TFA, A/B = 40/60, flow rate: 0.40 mL/min, detection: photodiode array detector 200–648 nm (UV chromatogram: 280 nm), temperature: 40 ºC.

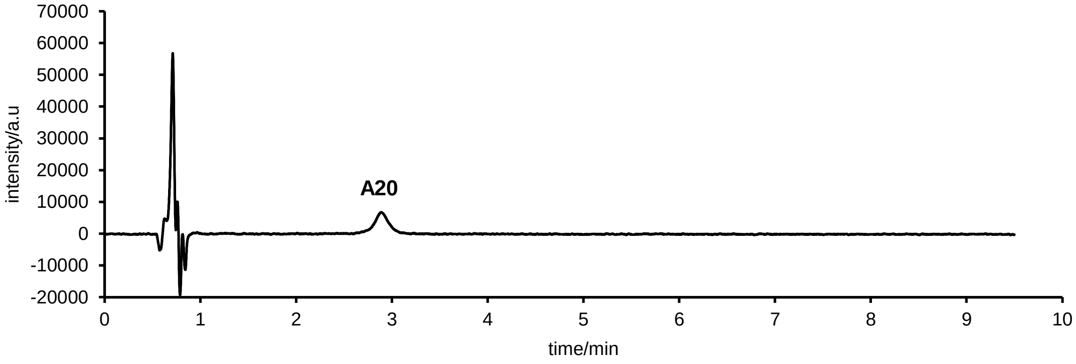

Supplementary Figure 144. UHPLC chart of the purified **A20**. Column: Accucore C18 2.1 ×150 mm, eluent A: MeCN + 0.05% TFA, eluent B: H_2O + 0.05% TFA, A/B = 40/60, flow rate: 0.40 mL/min, detection: photodiode array detector 200–648 nm (UV chromatogram: 280 nm), temperature: 40 ºC.

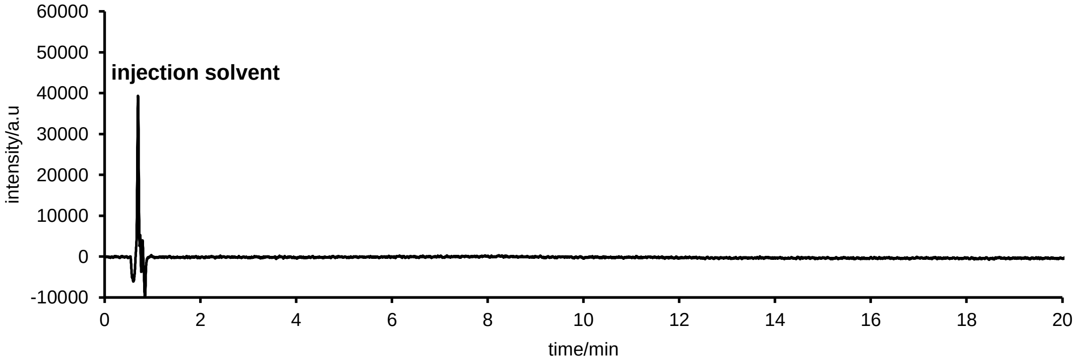

Supplementary Figure 145. UHPLC chart of the injection solvent. Column: Accucore C18 2.1 ×150 mm, eluent A: MeCN + 0.05% TFA, eluent B: H_2O + 0.05% TFA, A/B = 37.5/62.5, flow rate: 0.40 mL/min, detection: photodiode array detector 200–648 nm (UV chromatogram: 280 nm), temperature: 40 ºC.

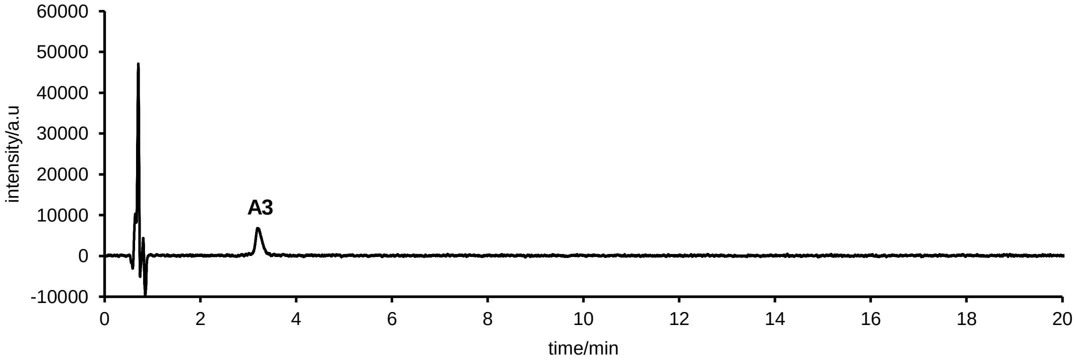

Supplementary Figure 146. UHPLC chart of purified **A3**. Column: Accucore C18 2.1 ×150 mm, eluent A: MeCN + 0.05% TFA, eluent B: H_2O + 0.05% TFA, A/B = 37.5/62.5, flow rate: 0.40 mL/min, detection: photodiode array detector 200–648 nm (UV chromatogram: 280 nm), temperature: 40 ºC.

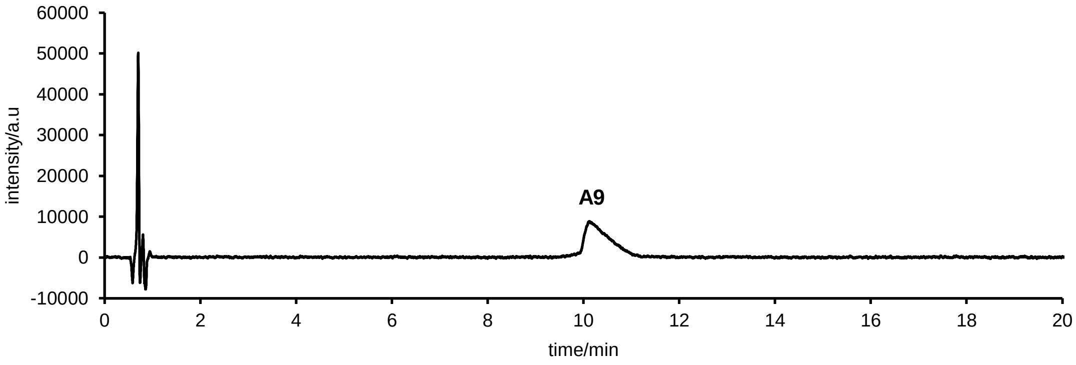

Supplementary Figure 147. UHPLC chart of purified **A9**. Column: Accucore C18 2.1 ×150 mm, eluent A: MeCN + 0.05% TFA, eluent B: H_2O + 0.05% TFA, A/B = 37.5/62.5, flow rate: 0.40 mL/min, detection: photodiode array detector 200–648 nm (UV chromatogram: 280 nm), temperature: 40 ºC.

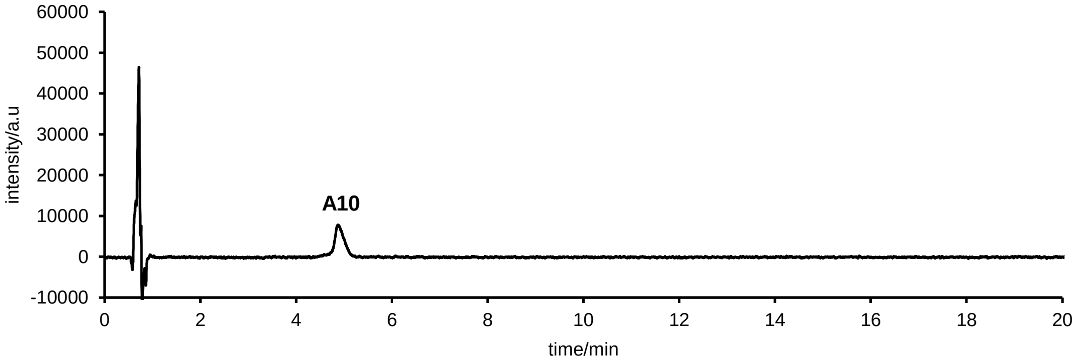

Supplementary Figure 148. UHPLC chart of purified **A10**. Column: Accucore C18 2.1 ×150 mm, eluent A: MeCN + 0.05% TFA, eluent B: H_2O + 0.05% TFA, A/B = 37.5/62.5, flow rate: 0.40 mL/min, detection: photodiode array detector 200–648 nm (UV chromatogram: 280 nm), temperature: 40 ºC.

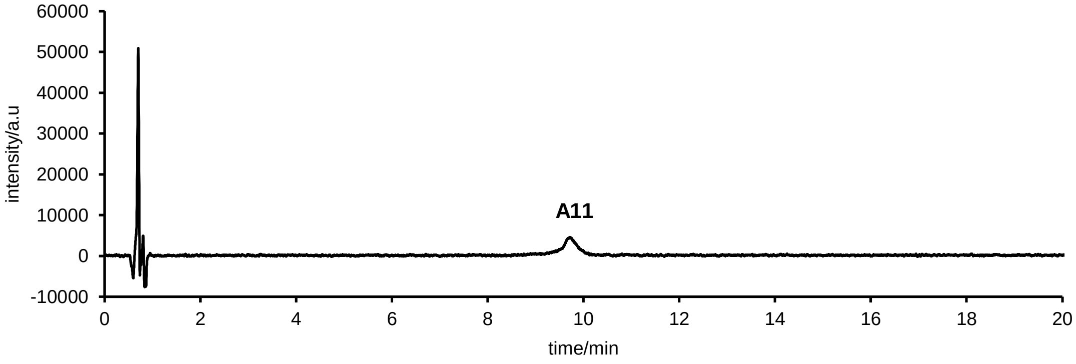

Supplementary Figure 149. UHPLC chart of purified **A11**. Column: Accucore C18 2.1 ×150 mm, eluent A: MeCN + 0.05% TFA, eluent B: H_2O + 0.05% TFA, A/B = 37.5/62.5, flow rate: 0.40 mL/min, detection: photodiode array detector 200–648 nm (UV chromatogram: 280 nm), temperature: 40 ºC.

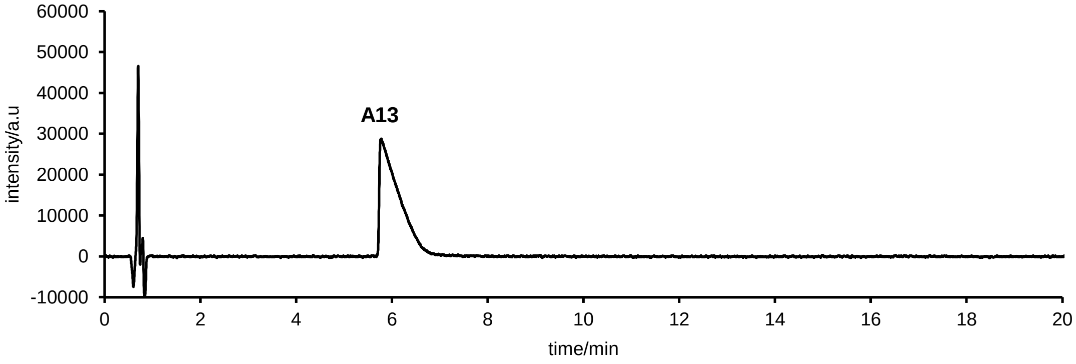

Supplementary Figure 150. UHPLC chart of purified **A13**. Column: Accucore C18 2.1 ×150 mm, eluent A: MeCN + 0.05% TFA, eluent B: H_2O + 0.05% TFA, A/B = 37.5/62.5, flow rate: 0.40 mL/min, detection: photodiode array detector 200–648 nm (UV chromatogram: 280 nm), temperature: 40 ºC.

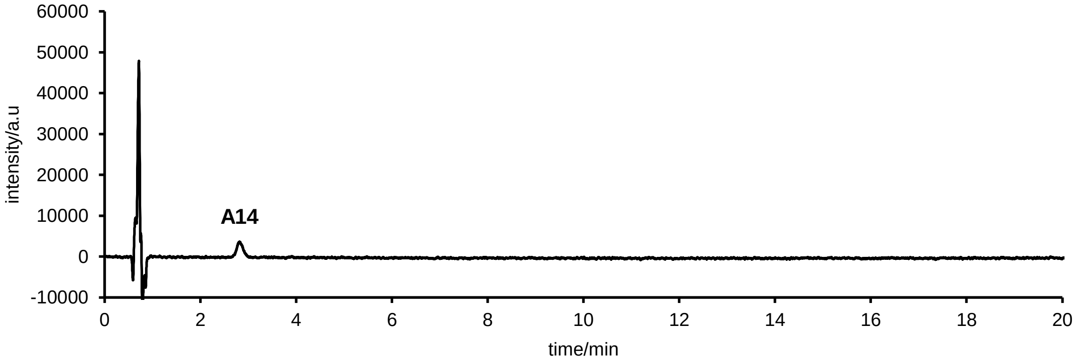

Supplementary Figure 151. UHPLC chart of purified **A14**. Column: Accucore C18 2.1 ×150 mm, eluent A: MeCN + 0.05% TFA, eluent B: H_2O + 0.05% TFA, A/B = 37.5/62.5, flow rate: 0.40 mL/min, detection: photodiode array detector 200–648 nm (UV chromatogram: 280 nm), temperature: 40 ºC.

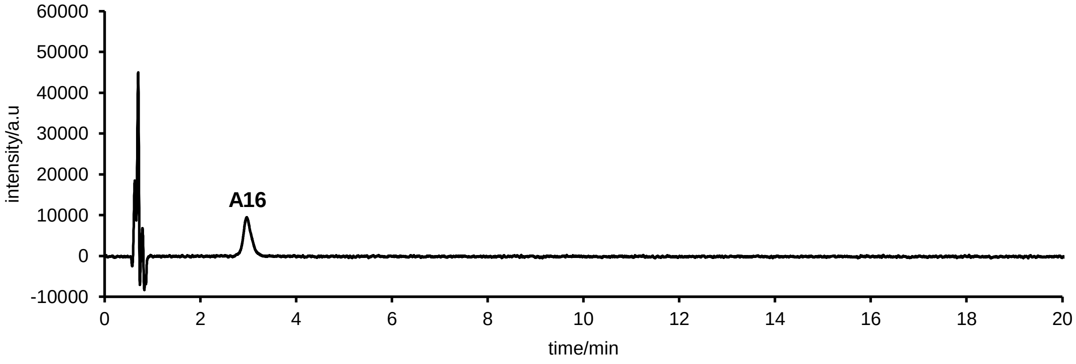

Supplementary Figure 152. UHPLC chart of purified **A16**. Column: Accucore C18 2.1 ×150 mm, eluent A: MeCN + 0.05% TFA, eluent B: H_2O + 0.05% TFA, A/B = 37.5/62.5, flow rate: 0.40 mL/min, detection: photodiode array detector 200–648 nm (UV chromatogram: 280 nm), temperature: 40 ºC.

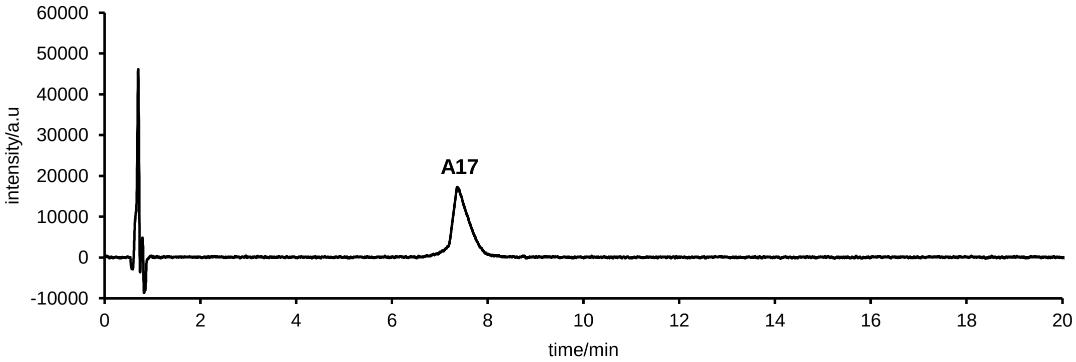

Supplementary Figure 153. UHPLC chart of purified **A17**. Column: Accucore C18 2.1 ×150 mm, eluent A: MeCN + 0.05% TFA, eluent B: H_2O + 0.05% TFA, A/B = 37.5/62.5, flow rate: 0.40 mL/min, detection: photodiode array detector 200–648 nm (UV chromatogram: 280 nm), temperature: 40 ºC.

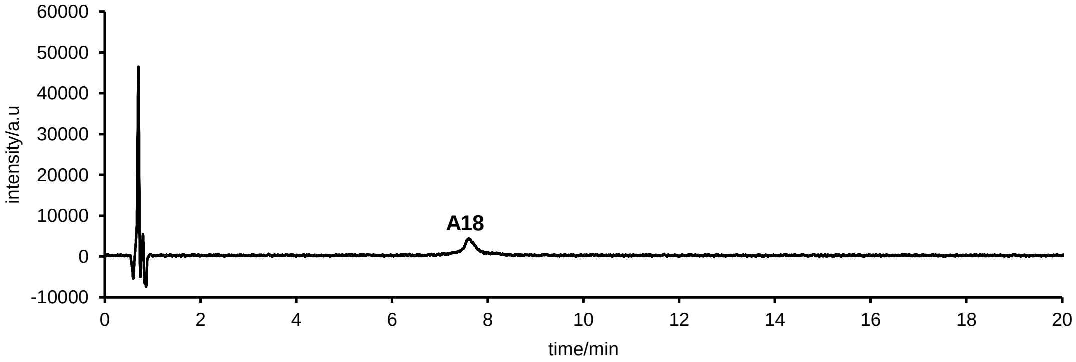

Supplementary Figure 154. UHPLC chart of purified **A18**. Column: Accucore C18 2.1 ×150 mm, eluent A: MeCN + 0.05% TFA, eluent B: H_2O + 0.05% TFA, A/B = 37.5/62.5, flow rate: 0.40 mL/min, detection: photodiode array detector 200–648 nm (UV chromatogram: 280 nm), temperature: 40 ºC.

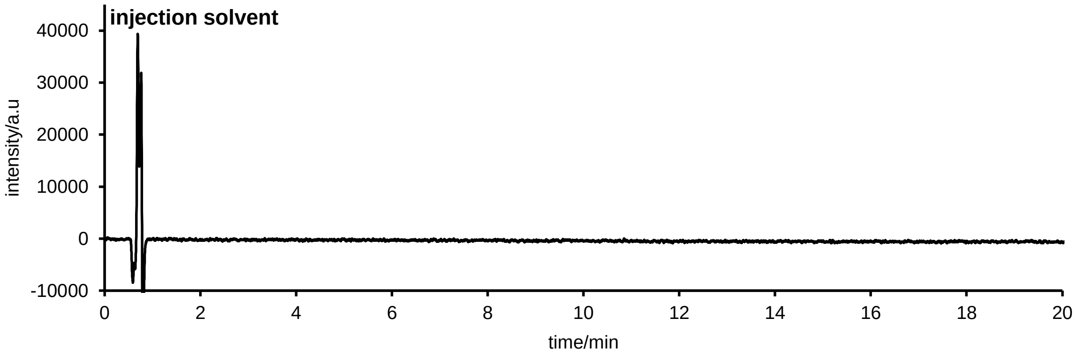

Supplementary Figure 155. UHPLC chart of the injection solvent. Column: Accucore C18 2.1 ×150 mm, eluent A: MeCN + 0.05% TFA, eluent B: H_2O + 0.05% TFA, A/B = 35/65, flow rate: 0.40 mL/min, detection: photodiode array detector 200–648 nm (UV chromatogram: 280 nm), temperature: 40 ºC.

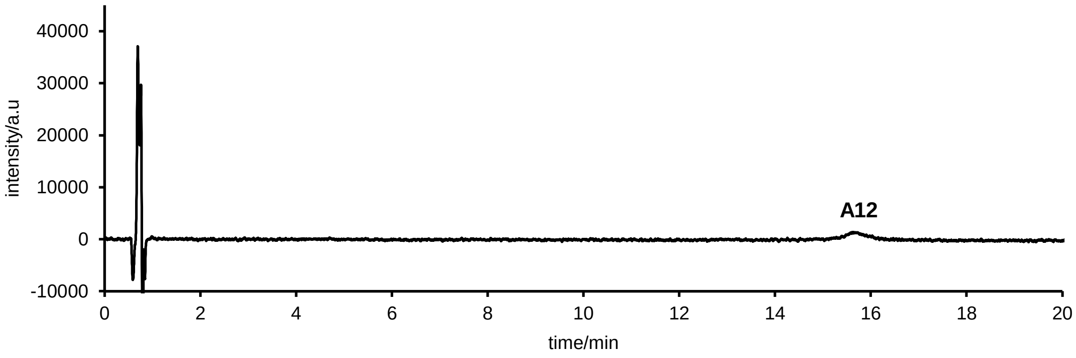

Supplementary Figure 156. UHPLC chart of purified **A12**. Column: Accucore C18 2.1 ×150 mm, eluent A: MeCN + 0.05% TFA, eluent B: H_2O + 0.05% TFA, A/B = 35/65, flow rate: 0.40 mL/min, detection: photodiode array detector 200–648 nm (UV chromatogram: 280 nm), temperature: 40 °C.

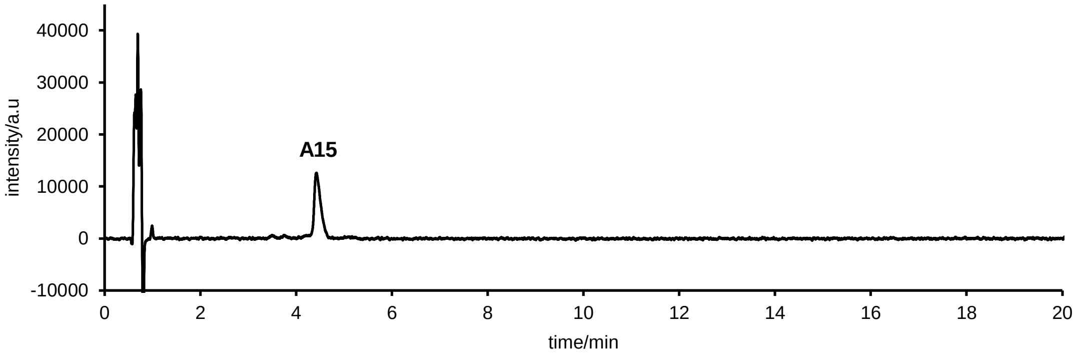

Supplementary Figure 157. UHPLC chart of purified **A15**. Column: Accucore C18 2.1 ×150 mm, eluent A: MeCN + 0.05% TFA, eluent B: H_2O + 0.05% TFA, A/B = 35/65, flow rate: 0.40 mL/min, detection: photodiode array detector 200–648 nm (UV chromatogram: 280 nm), temperature: 40 °C.

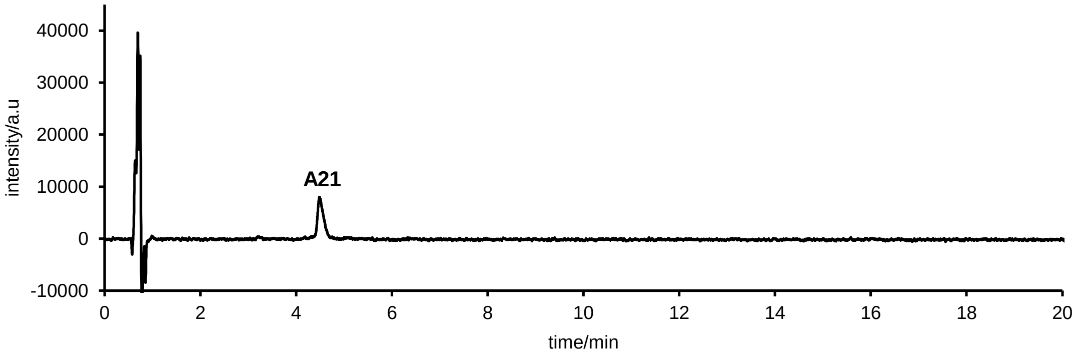

Supplementary Figure 158. UHPLC chart of purified **A21**. Column: Accucore C18 2.1 ×150 mm, eluent A: MeCN + 0.05% TFA, eluent B: H_2O + 0.05% TFA, A/B = 35/65, flow rate: 0.40 mL/min, detection: photodiode array detector 200–648 nm (UV chromatogram: 280 nm), temperature: 40 °C.

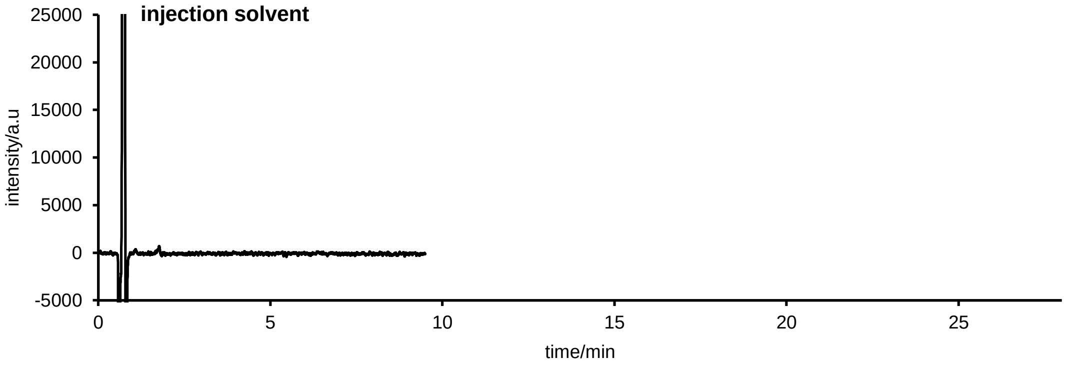

Supplementary Figure 159. UHPLC chart of the injection solvent. Column: Accucore C18 2.1 ×150 mm, eluent A: MeCN + 0.05% TFA, eluent B: H_2O + 0.05% TFA, A/B = 30/70, flow rate: 0.40 mL/min, detection: photodiode array detector 200–648 nm (UV chromatogram: 280 nm), temperature: 40 ºC.

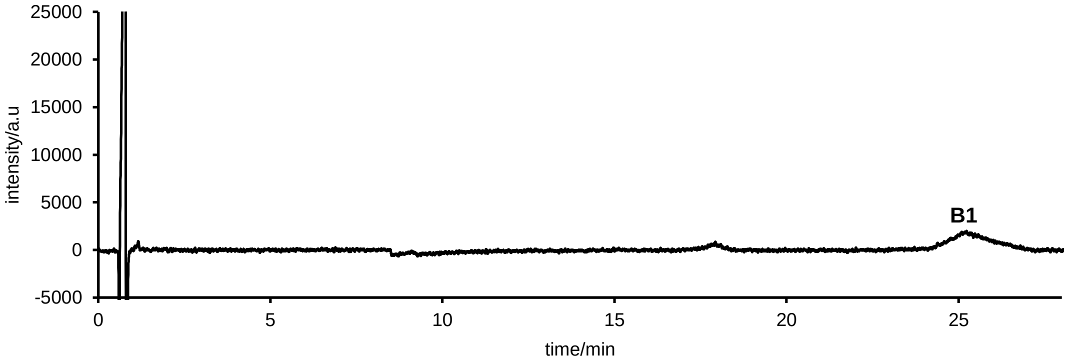

Supplementary Figure 160. UHPLC chart of purified **B1**. Column: Accucore C18 2.1 ×150 mm, eluent A: MeCN + 0.05% TFA, eluent B: H_2O + 0.05% TFA, A/B = 30/70, flow rate: 0.40 mL/min, detection: photodiode array detector 200–648 nm (UV chromatogram: 280 nm), temperature: 40 ºC.

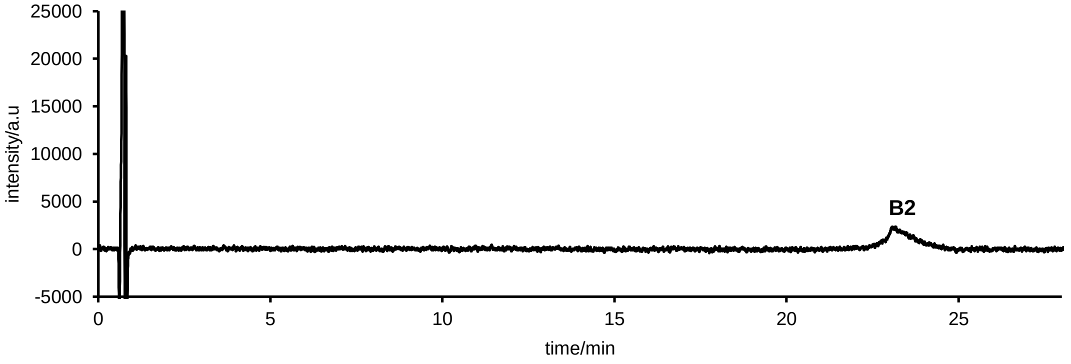

Supplementary Figure 161. UHPLC chart of purified **B2**. Column: Accucore C18 2.1 ×150 mm, eluent A: MeCN + 0.05% TFA, eluent B: H_2O + 0.05% TFA, A/B = 30/70, flow rate: 0.40 mL/min, detection: photodiode array detector 200–648 nm (UV chromatogram: 280 nm), temperature: 40 ºC.

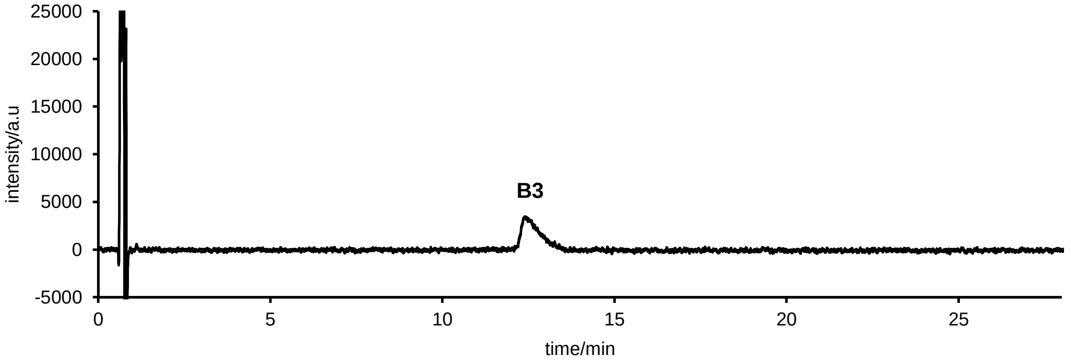

Supplementary Figure 162. UHPLC chart of purified **B3**. Column: Accucore C18 2.1 ×150 mm, eluent A: MeCN + 0.05% TFA, eluent B: H_2O + 0.05% TFA, A/B = 30/70, flow rate: 0.40 mL/min, detection: photodiode array detector 200–648 nm (UV chromatogram: 280 nm), temperature: 40 ºC.

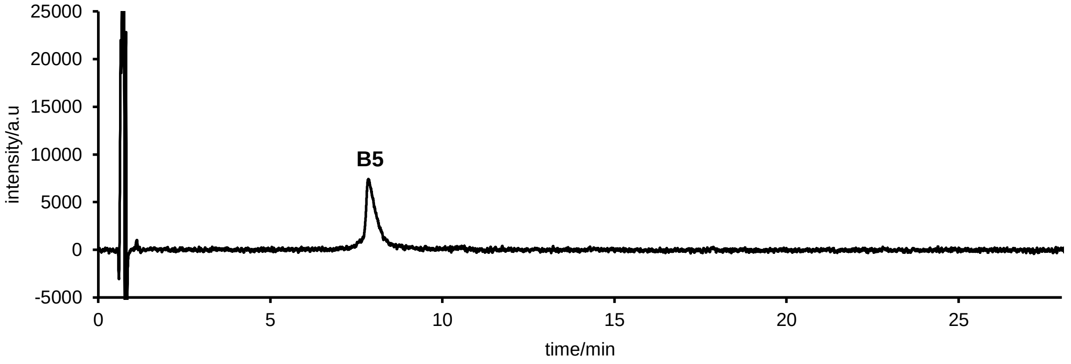

Supplementary Figure 163. UHPLC chart of purified **B5**. Column: Accucore C18 2.1 ×150 mm, eluent A: MeCN + 0.05% TFA, eluent B: H_2O + 0.05% TFA, A/B = 30/70, flow rate: 0.40 mL/min, detection: photodiode array detector 200–648 nm (UV chromatogram: 280 nm), temperature: 40 ºC.

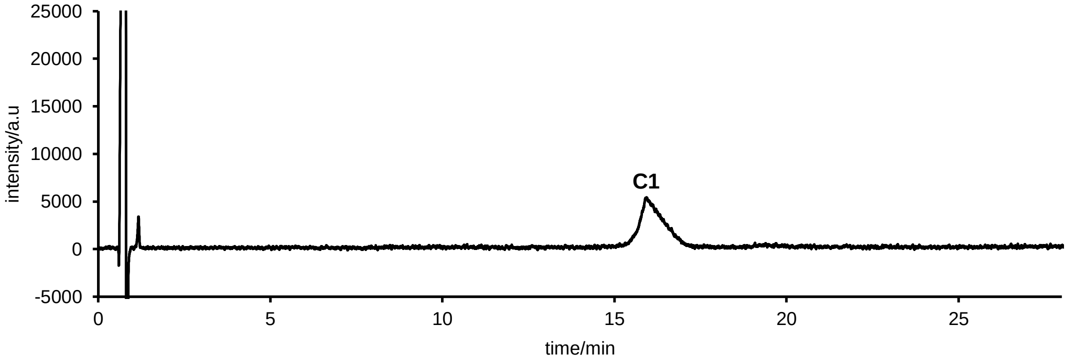

Supplementary Figure 164. UHPLC chart of purified **C1**. Column: Accucore C18 2.1 ×150 mm, eluent A: MeCN + 0.05% TFA, eluent B: H_2O + 0.05% TFA, A/B = 30/70, flow rate: 0.40 mL/min, detection: photodiode array detector 200–648 nm (UV chromatogram: 280 nm), temperature: 40 ºC.

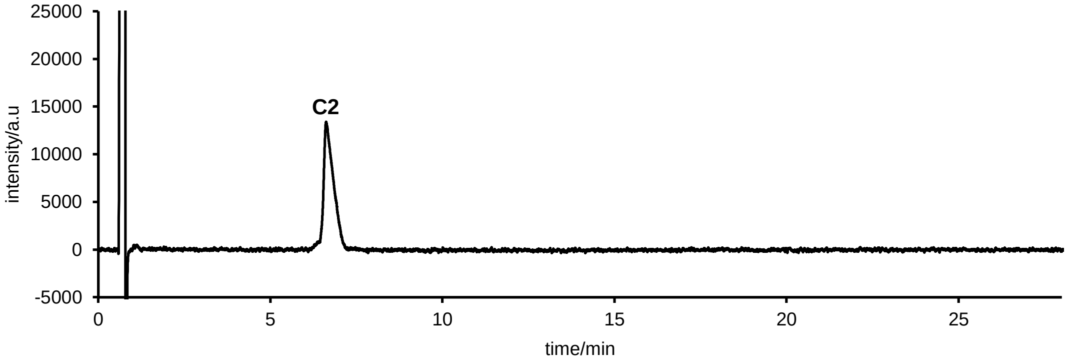

Supplementary Figure 165. UHPLC chart of purified **C2**. Column: Accucore C18 2.1 ×150 mm, eluent A: MeCN + 0.05% TFA, eluent B: H_2O + 0.05% TFA, A/B = 30/70, flow rate: 0.40 mL/min, detection: photodiode array detector 200–648 nm (UV chromatogram: 280 nm), temperature: 40 ºC.

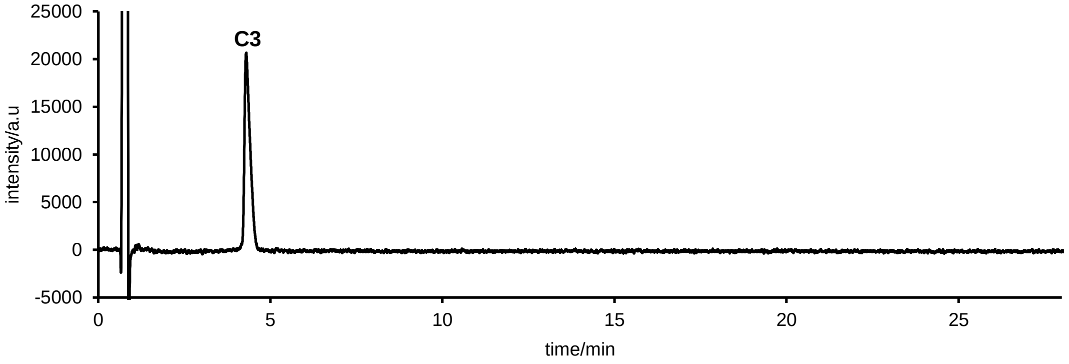

Supplementary Figure 166. UHPLC chart of purified **C3**. Column: Accucore C18 2.1 ×150 mm, eluent A: MeCN + 0.05% TFA, eluent B: H_2O + 0.05% TFA, A/B = 30/70, flow rate: 0.40 mL/min, detection: photodiode array detector 200–648 nm (UV chromatogram: 280 nm), temperature: 40 ºC.

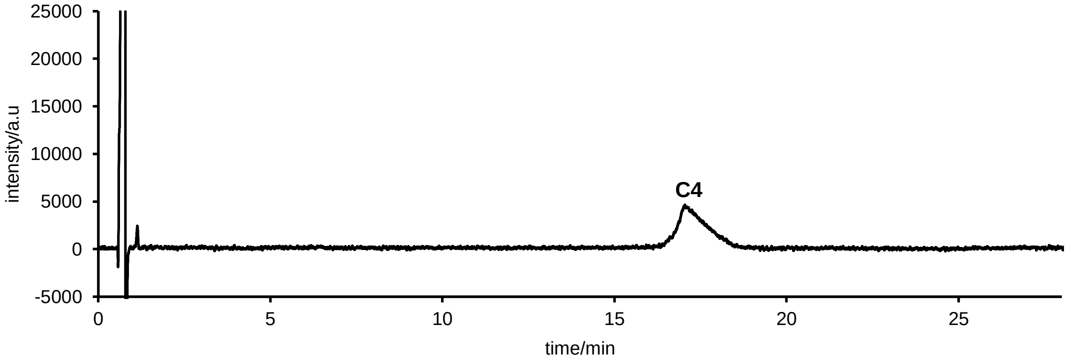

Supplementary Figure 167. UHPLC chart of purified **C4**. Column: Accucore C18 2.1 ×150 mm, eluent A: MeCN + 0.05% TFA, eluent B: H_2O + 0.05% TFA, A/B = 30/70, flow rate: 0.40 mL/min, detection: photodiode array detector 200–648 nm (UV chromatogram: 280 nm), temperature: 40 ºC.

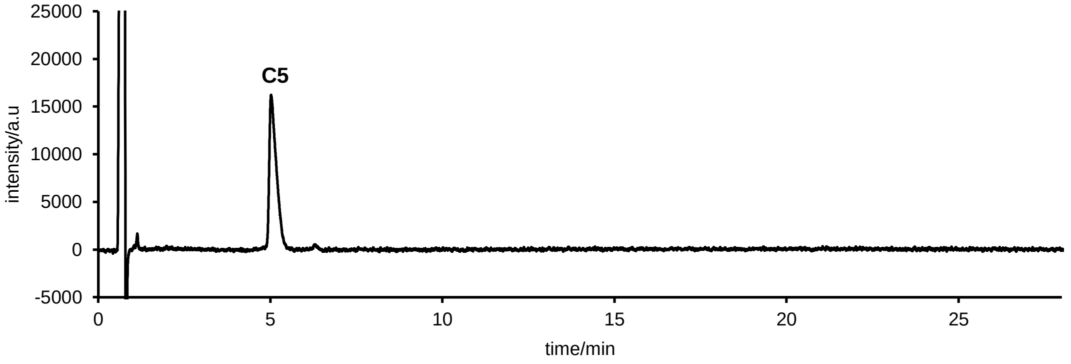

Supplementary Figure 168. UHPLC chart of purified **C5**. Column: Accucore C18 2.1 ×150 mm, eluent A: MeCN + 0.05% TFA, eluent B: H_2O + 0.05% TFA, A/B = 30/70, flow rate: 0.40 mL/min, detection: photodiode array detector 200–648 nm (UV chromatogram: 280 nm), temperature: 40 ºC.

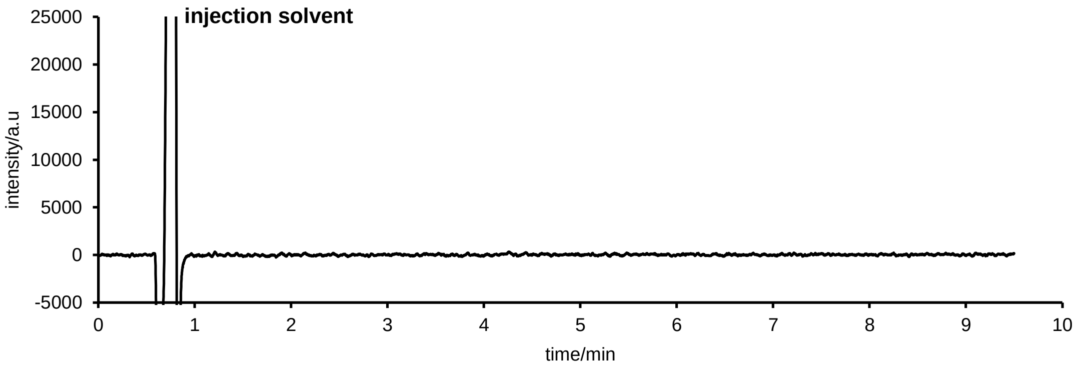

Supplementary Figure 169. UHPLC chart of the injection solvent. Column: Accucore C18 2.1 ×150 mm, eluent A: MeCN + 0.05% TFA, eluent B: H_2O + 0.05% TFA, A/B = 25/75, flow rate: 0.40 mL/min, detection: photodiode array detector 200–648 nm (UV chromatogram: 280 nm), temperature: 40 ºC.

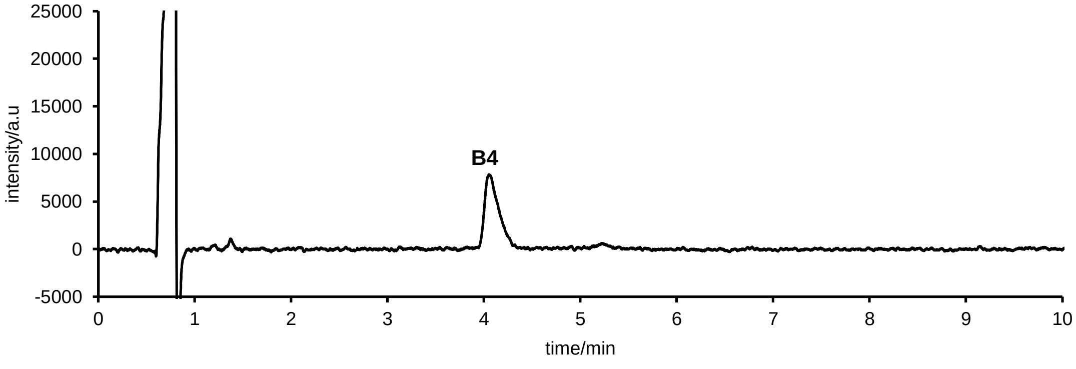

Supplementary Figure 170. UHPLC chart of purified **B4**. Column: Accucore C18 2.1 ×150 mm, eluent A: MeCN + 0.05% TFA, eluent B: H_2O + 0.05% TFA, A/B = 25/75, flow rate: 0.40 mL/min, detection: photodiode array detector 200–648 nm (UV chromatogram: 280 nm), temperature: 40 ºC.

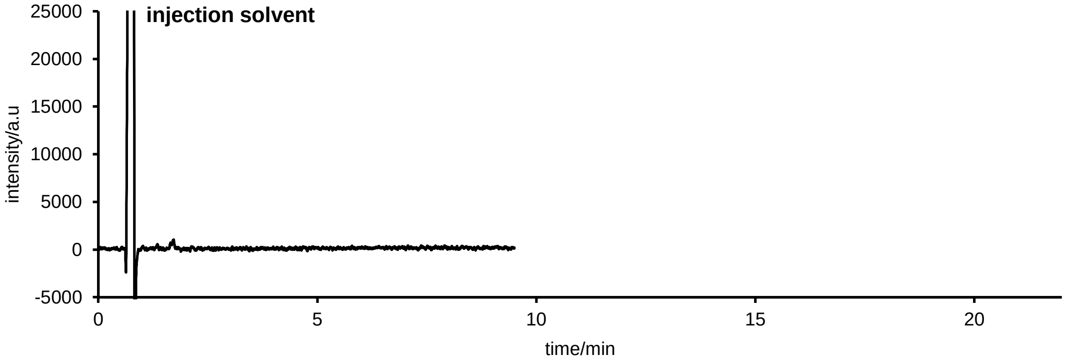

Supplementary Figure 171. UHPLC chart of the injection solvent. Column: Accucore C18 2.1 ×150 mm, eluent A: MeCN + 0.05% TFA, eluent B: H_2O + 0.05% TFA, A/B = 20/80, flow rate: 0.40 mL/min, detection: photodiode array detector 200–648 nm (UV chromatogram: 280 nm), temperature: 40 °C.

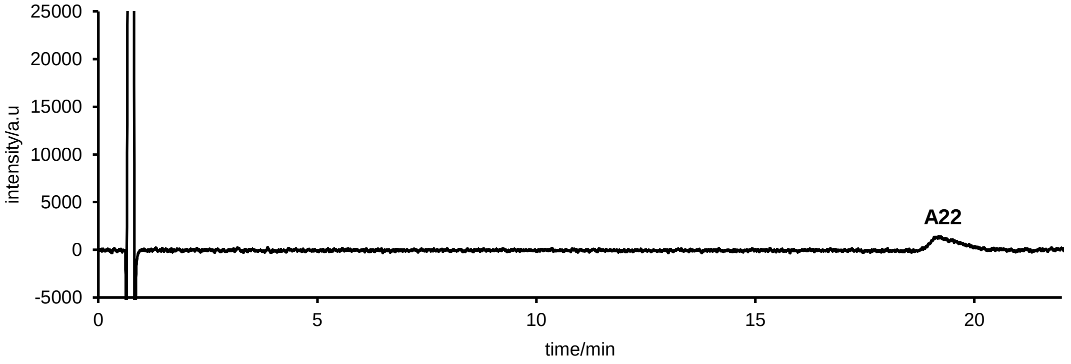

Supplementary Figure 172. UHPLC chart of purified **A22**. Column: Accucore C18 2.1 ×150 mm, eluent A: MeCN + 0.05% TFA, eluent B: H_2O + 0.05% TFA, A/B = 20/80, flow rate: 0.40 mL/min, detection: photodiode array detector 200–648 nm (UV chromatogram: 280 nm), temperature: 40 °C.

plate 1/line A/column 1

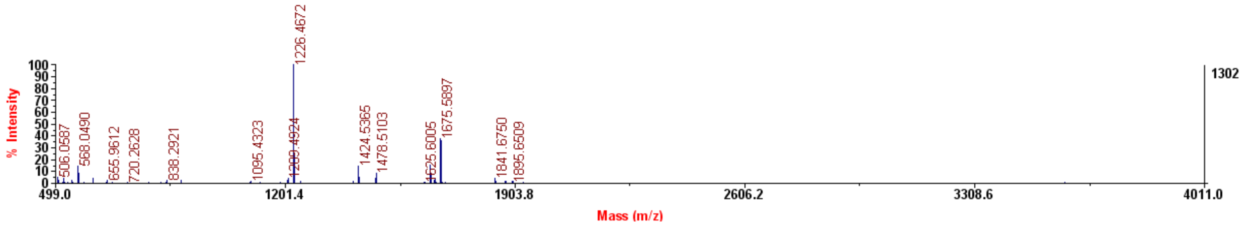

plate 1/line B/column 1

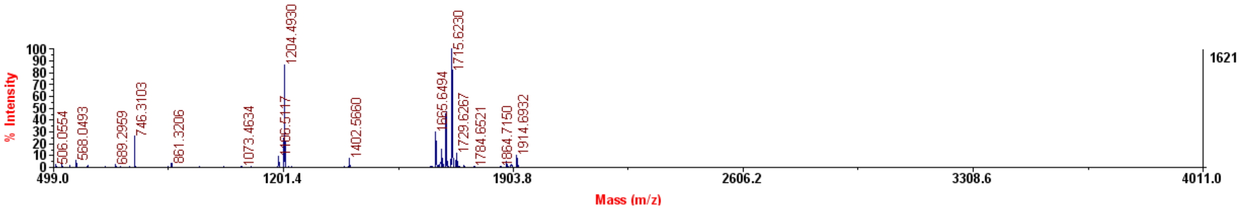

plate 1/line C/column 1

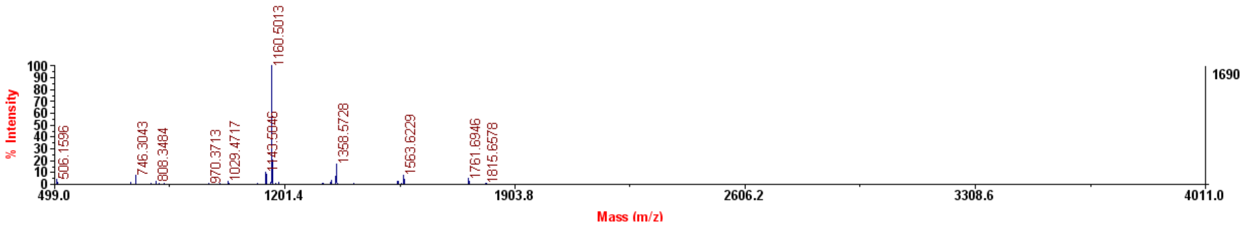

plate 1/line D/column 1

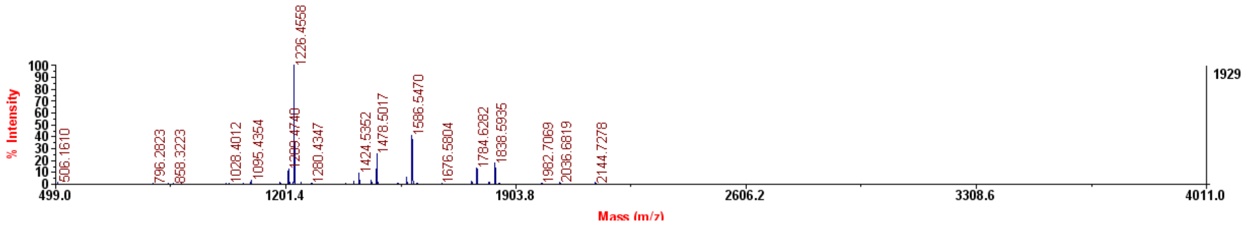

plate 1/line E/column 1

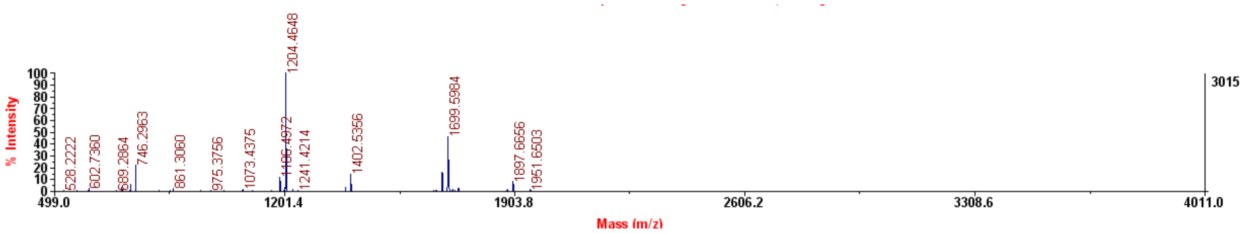

plate 1/line F/column 1

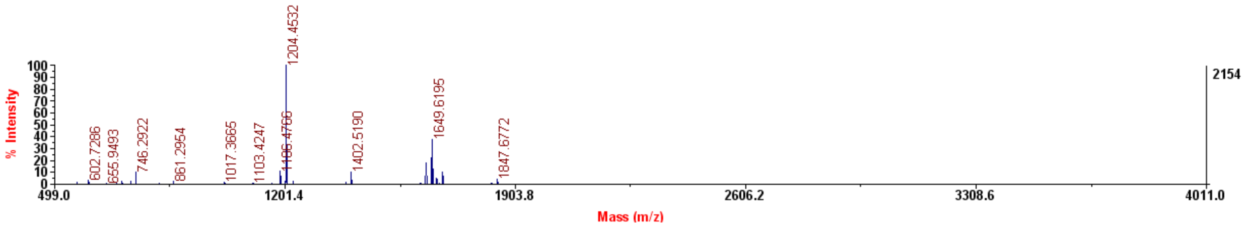

Supplementary Figure 173. MS spectra of plate 1. The data of A1–F1 are shown.

plate 1/line G/column 1

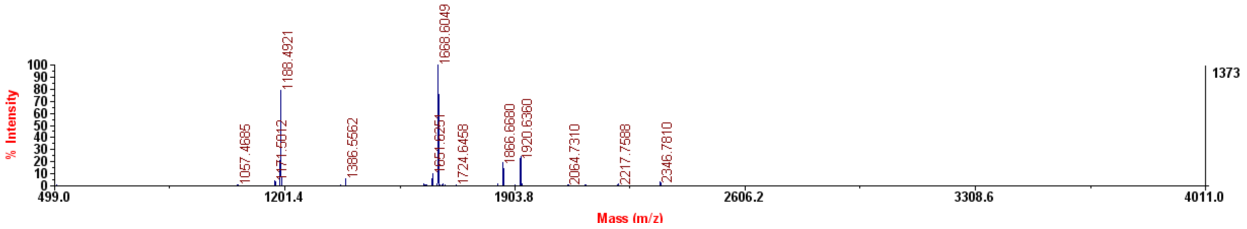

plate 1/line H/column 1

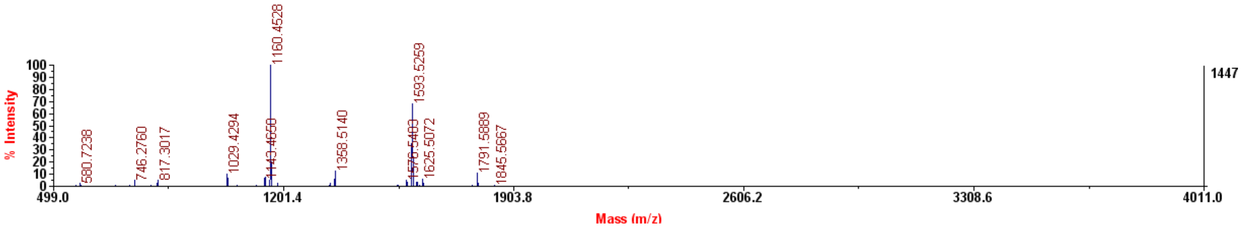

plate 1/line A/column 2

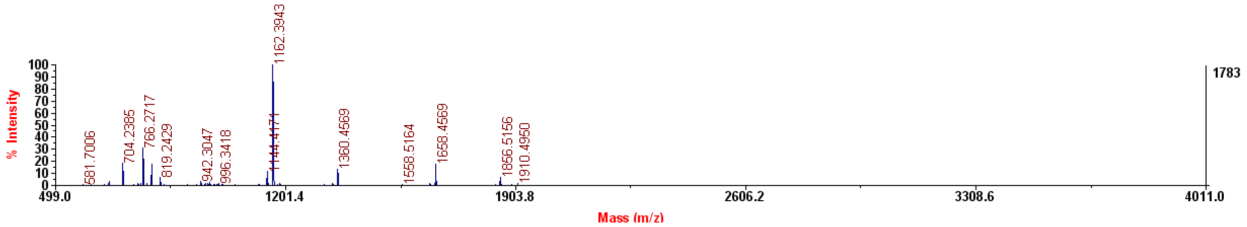

plate 1/line B/column 2

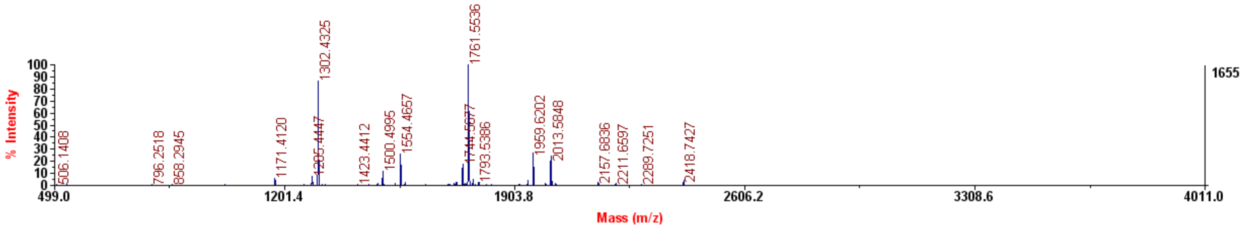

plate 1/line C/column 2

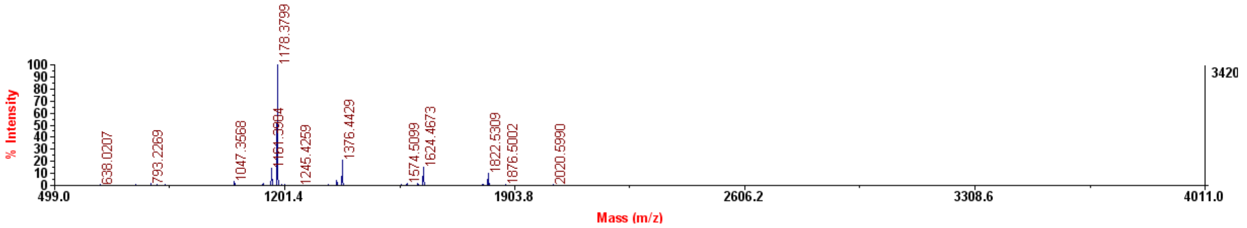

plate 1/line D/column 2

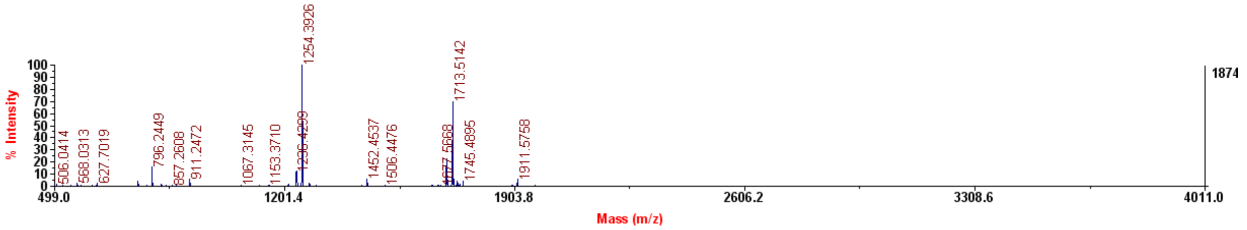

Supplementary Figure 174. MS spectra of plate 1. The data of G1, H1, and A2–D2 are shown.

plate 1/line E/column 2

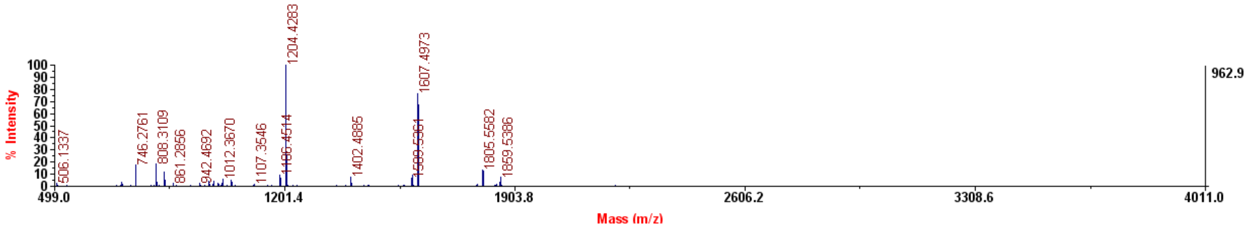

plate 1/line F/column 2

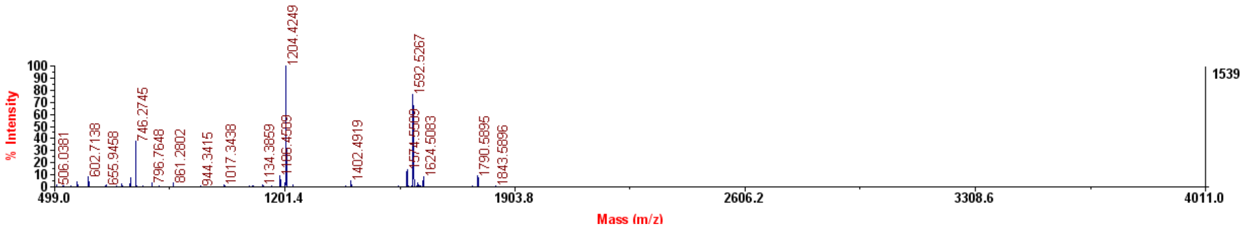

plate 1/line G/column 2

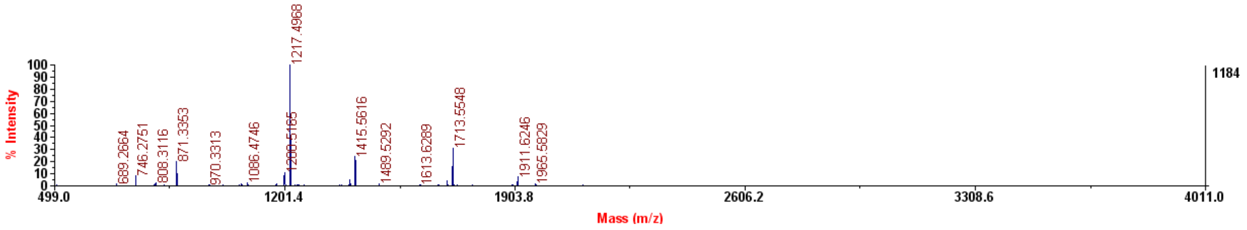

plate 1/line H/column 2

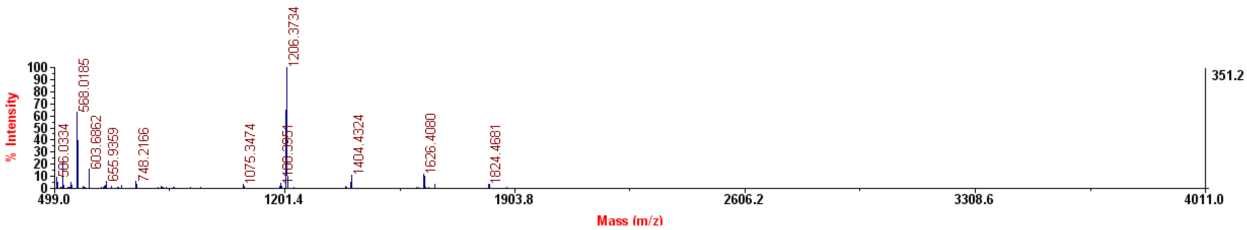

plate 1/line A/column 3

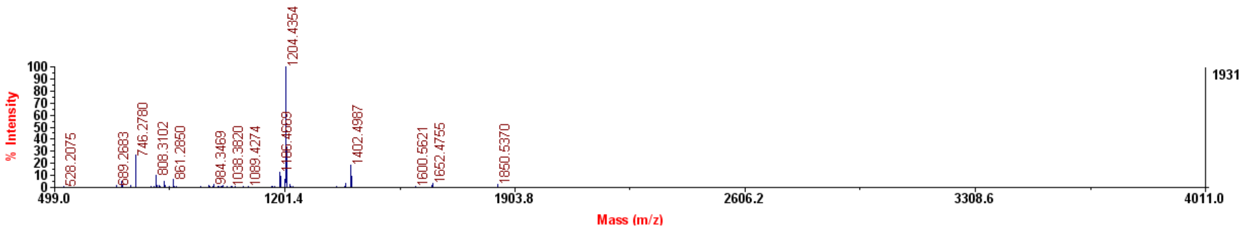

plate 1/line B/column 3

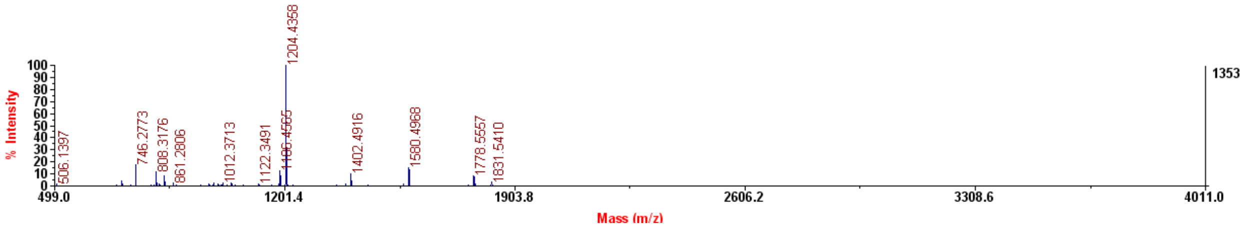

Supplementary Figure 175. MS spectra of plate 1. The data of E2–H2, A3, and B3 are shown.

plate 1/line C/column 3

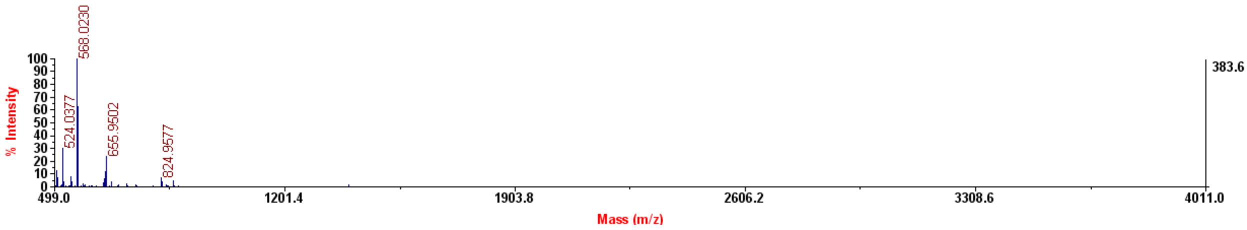

plate 1/line D/column 3

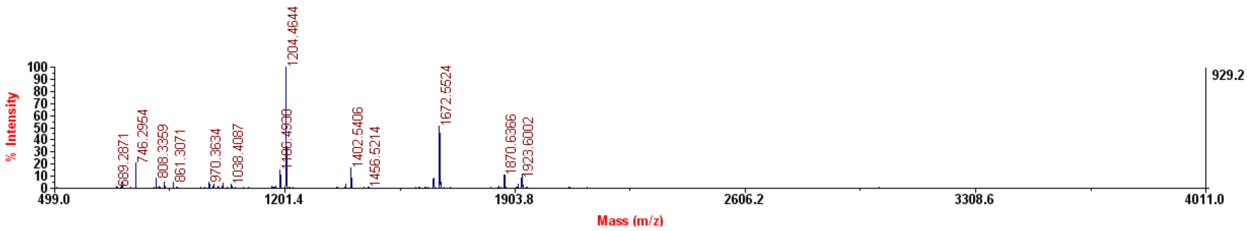

plate 1/line E/column 3

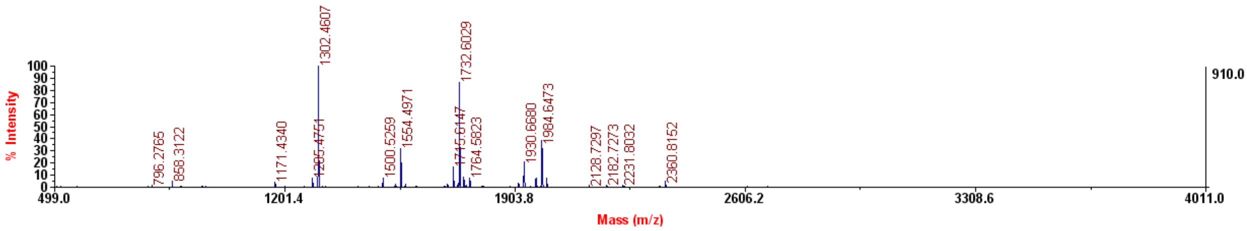

plate 1/line F/column 3

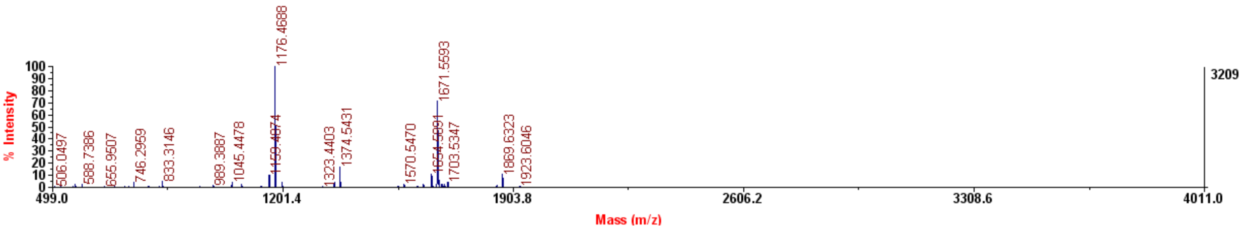

plate 1/line G/column 3

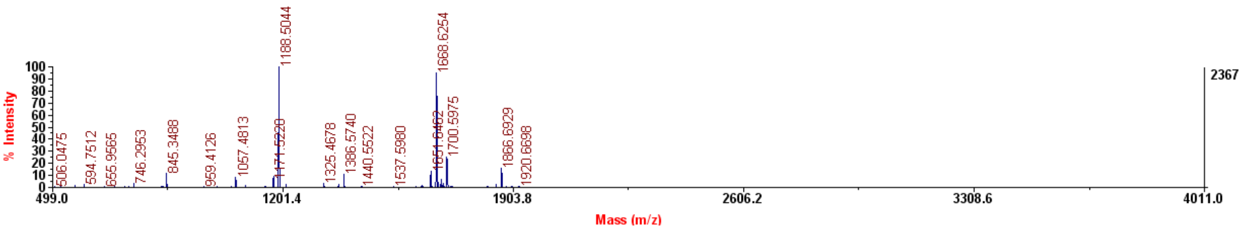

plate 1/line H/column 3

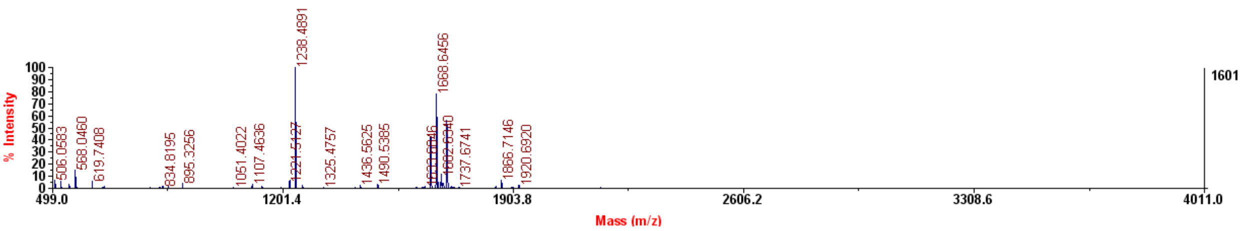

Supplementary Figure 176. MS spectra of plate 1. The data of C3–H3 are shown.

plate 1/line A/column 4

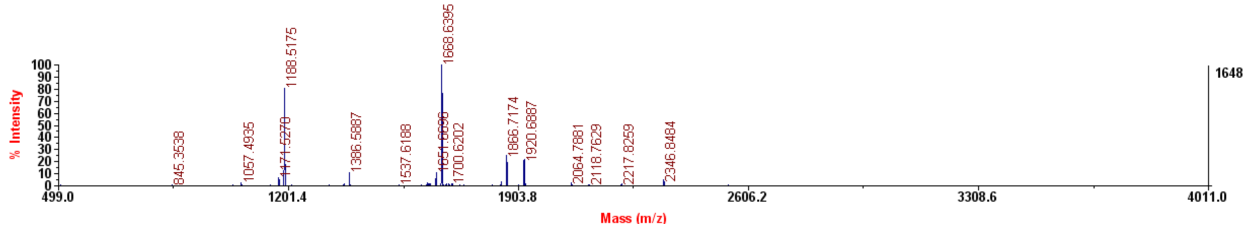

plate 1/line B/column 4

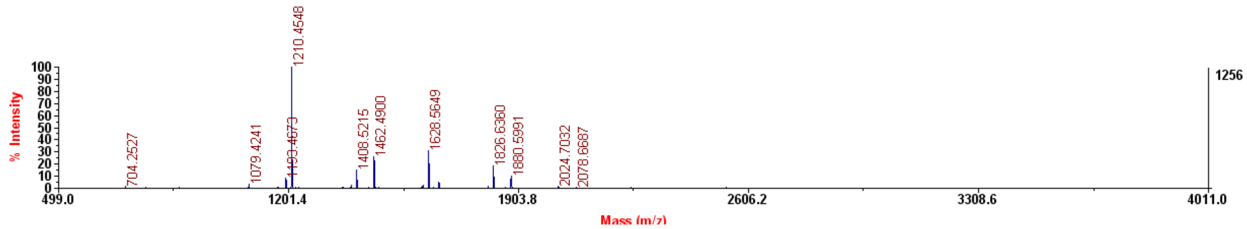

plate 1/line C/column 4

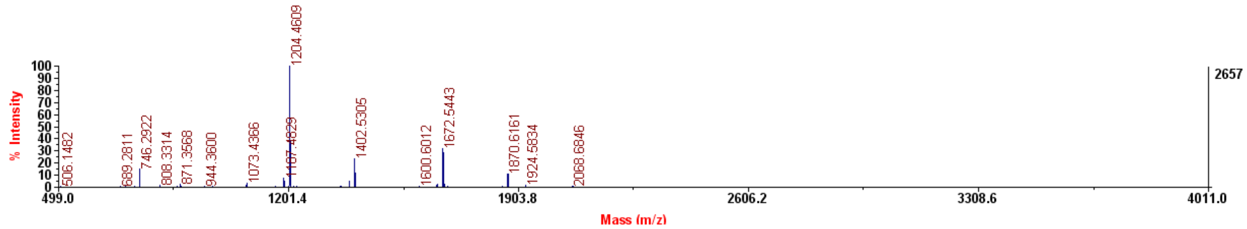

plate 1/line D/column 4

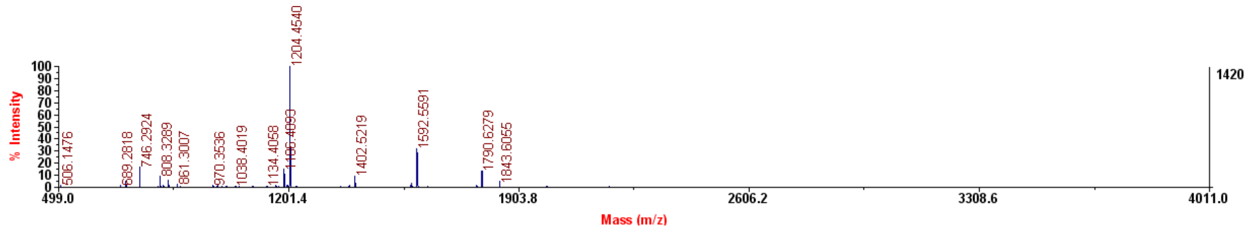

plate 1/line E/column 4

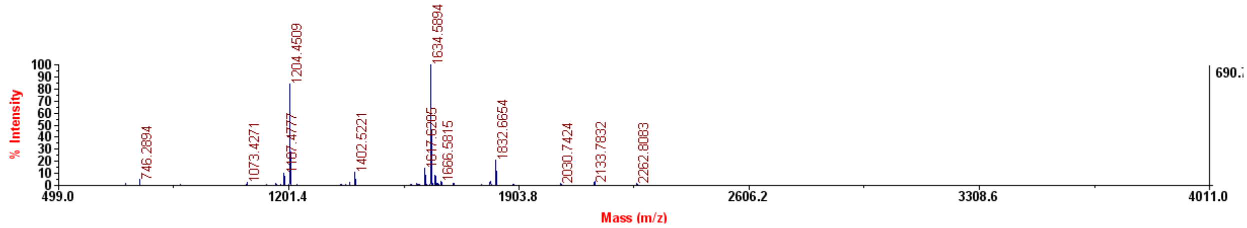

plate 1/line F/column 4

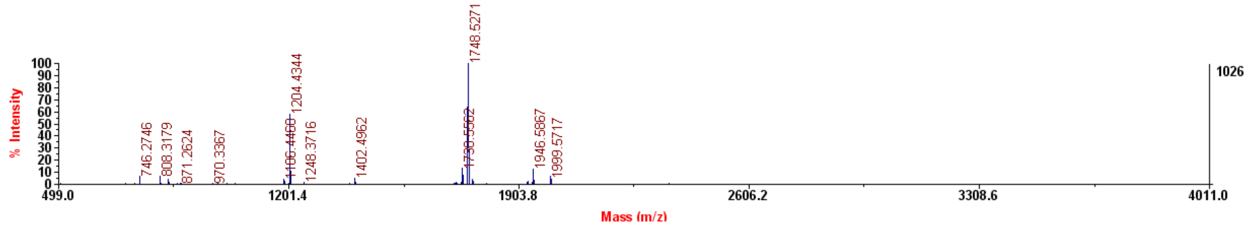

Supplementary Figure 177. MS spectra of plate 1. The data of A4–F4 are shown.

plate 1/line G/column 4

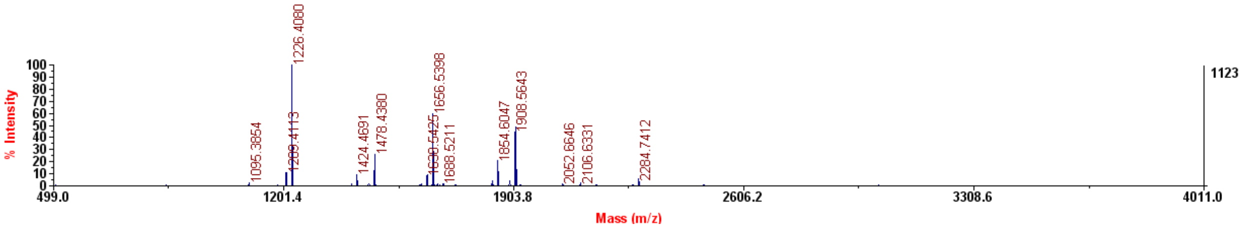

plate 1/line H/column 4

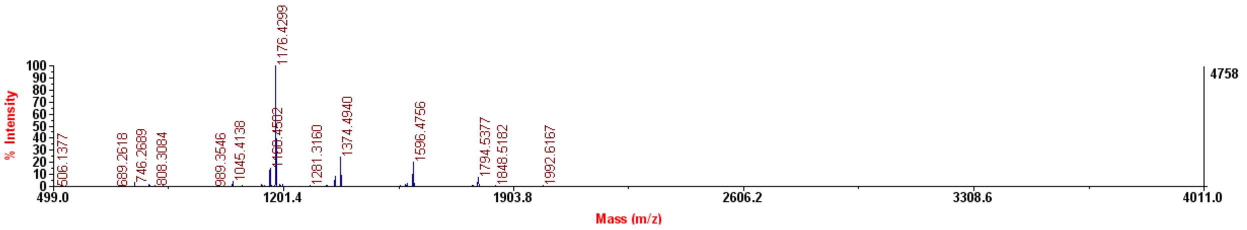

plate 1/line A/column 5

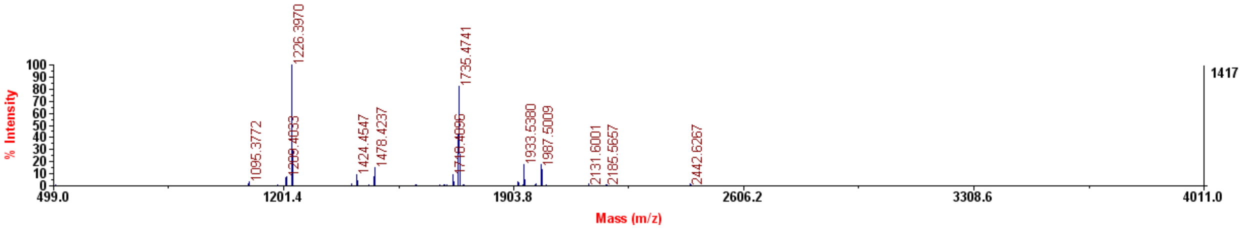

plate 1/line B/column 5

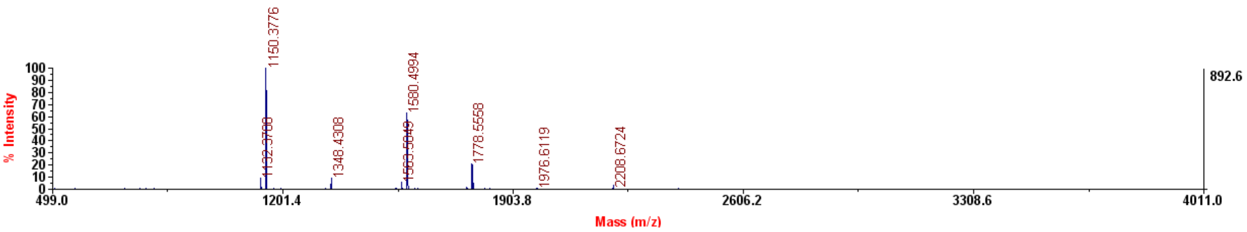

plate 1/line C/column 5

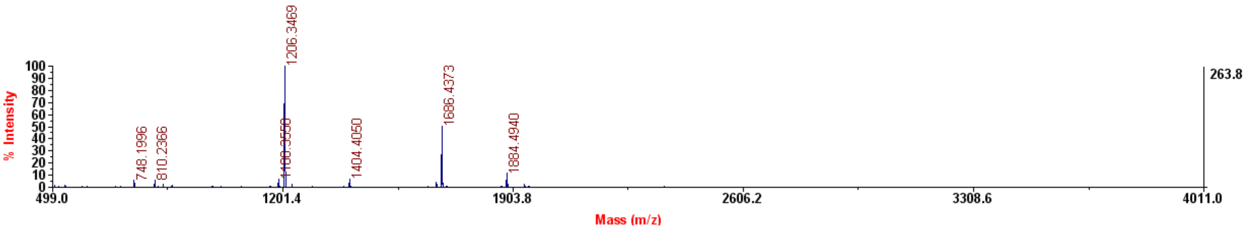

plate 1/line D/column 5

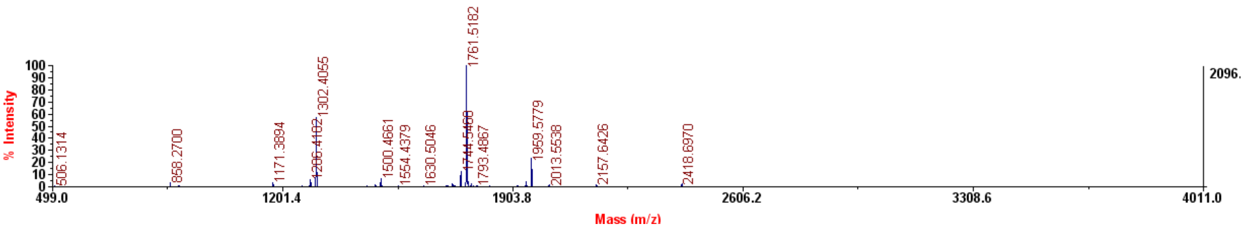

Supplementary Figure 178. MS spectra of plate 1. The data of G4, H4, and A5–D5 are shown.

plate 1/line E/column 5

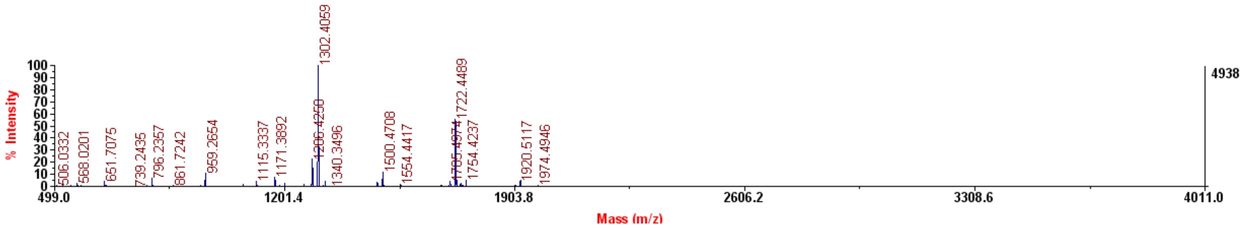

plate 1/line F/column 5

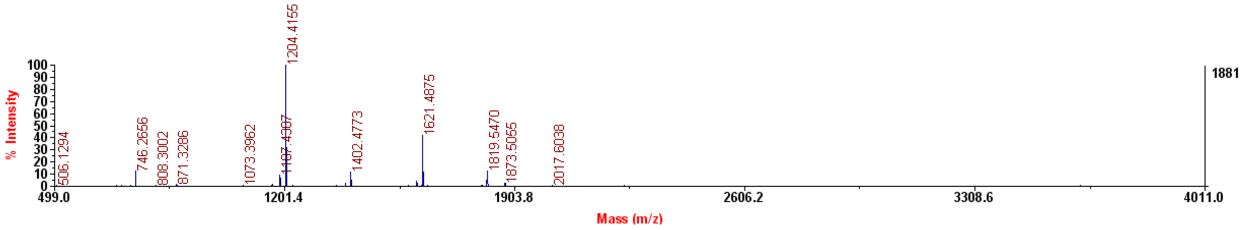

plate 1/line G/column 5

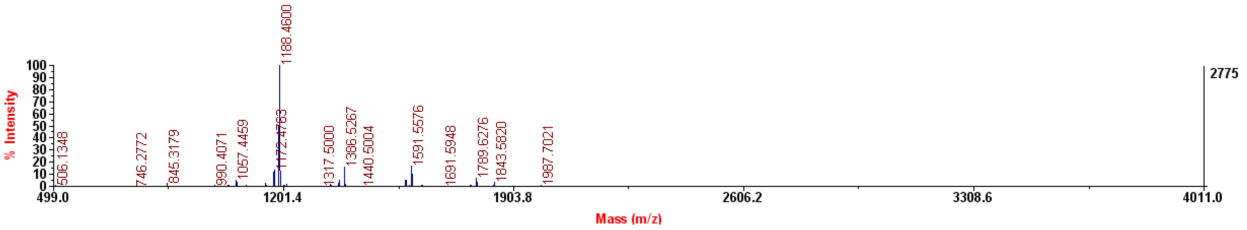

plate 1/line H/column 5

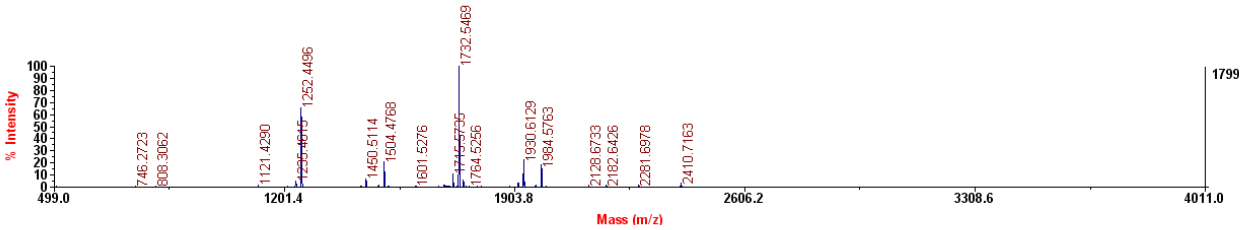

plate 1/line A/column 6

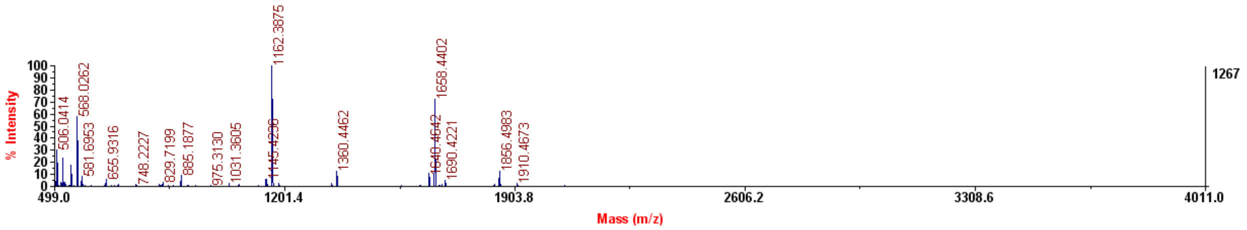

plate 1/line B/column 6

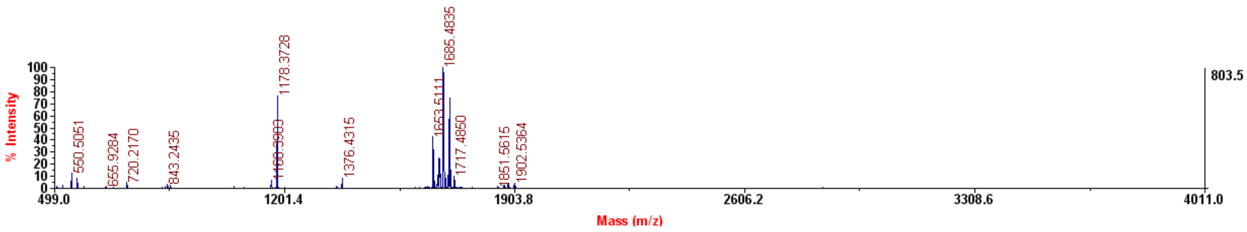

Supplementary Figure 179. MS spectra of plate 1. The data of E5–H5, A6, and B6 are shown.

plate 1/line C/column 6

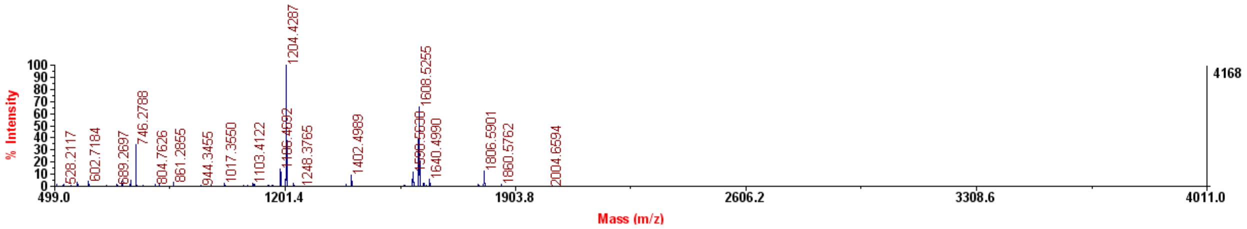

plate 1/line D/column 6

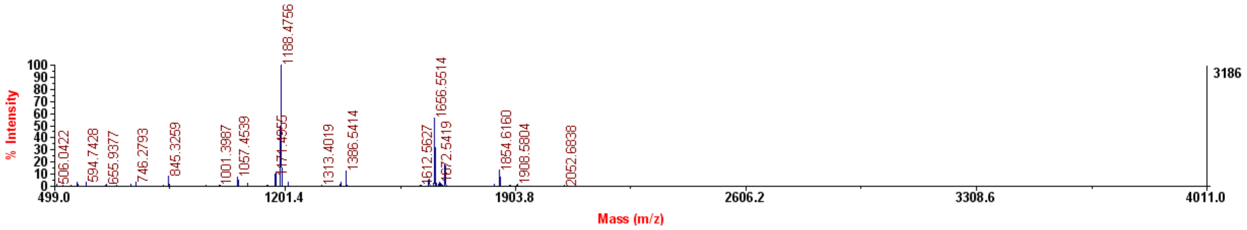

plate 1/line E/column 6

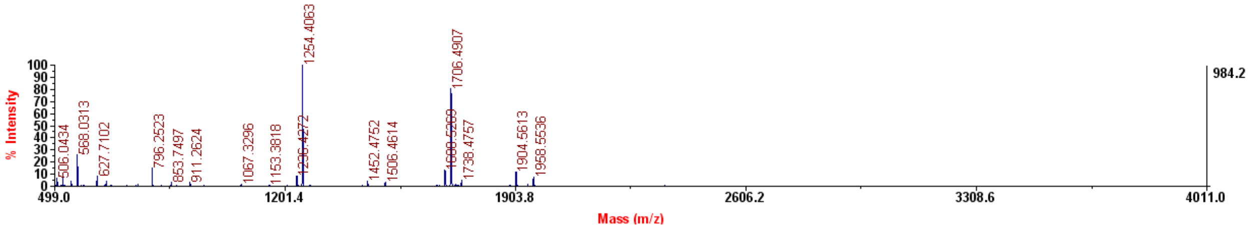

plate 1/line F/column 6

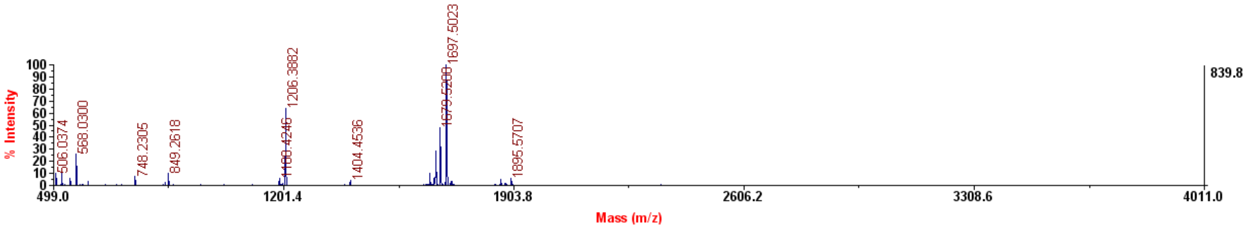

plate 1/line G/column 6

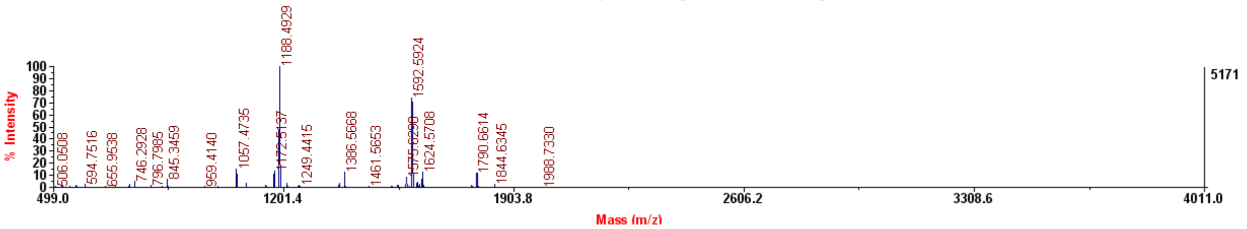

plate 1/line H/column 6

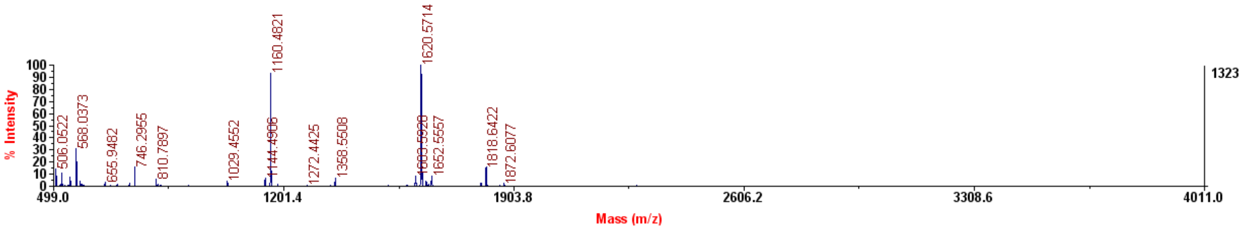

Supplementary Figure 180. MS spectra of plate 1. The data of C6–H6 are shown.

plate 1/line A/column 7

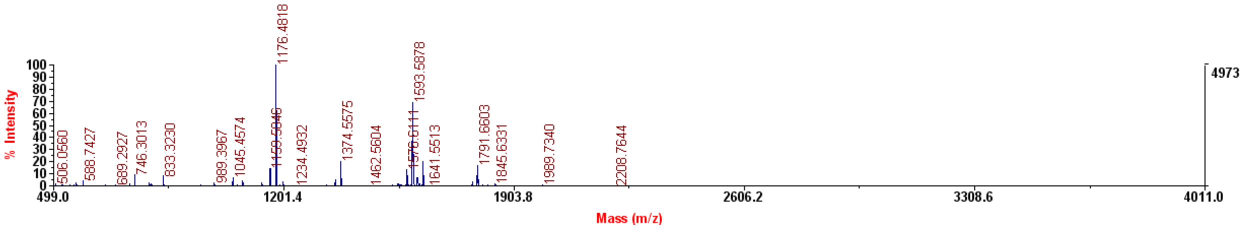

plate 1/line B/column 7

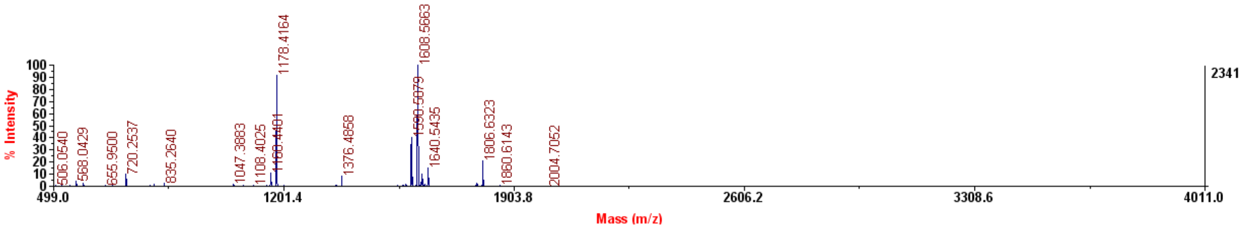

plate 1/line C/column 7

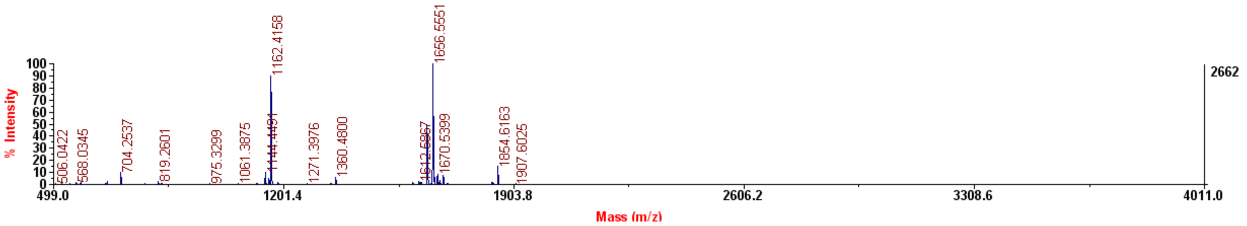

plate 1/line D/column 7

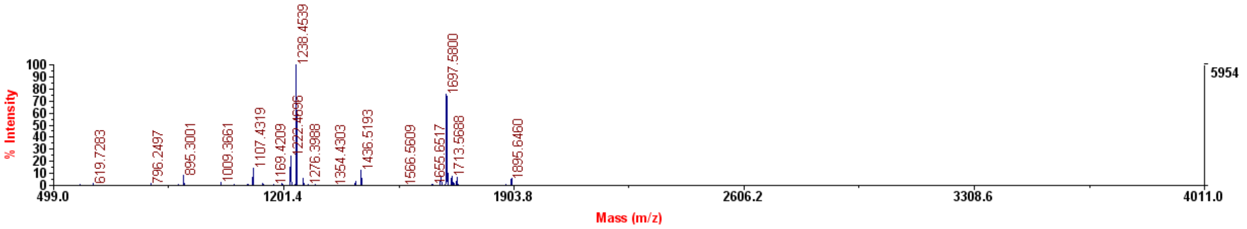

plate 1/line E/column 7

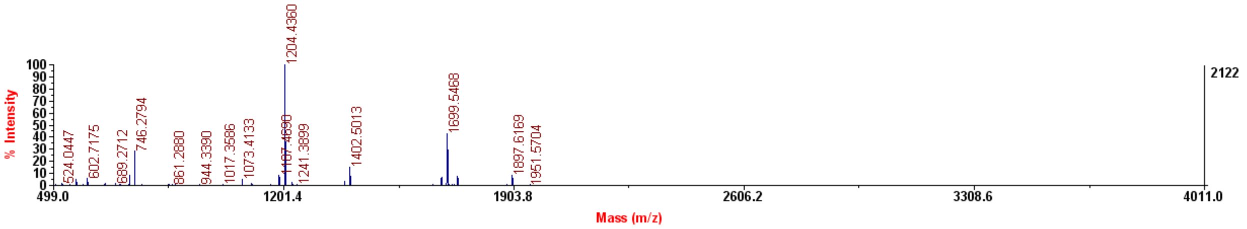

plate 1/line F/column 7

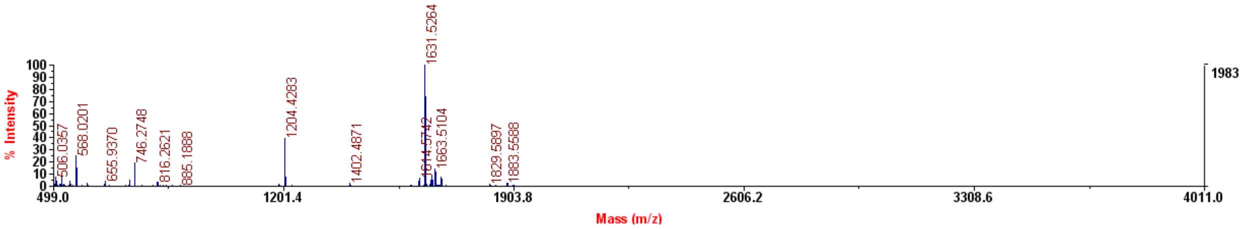

Supplementary Figure 181. MS spectra of plate 1. The data of A7–F7 are shown.

plate 1/line G/column 7

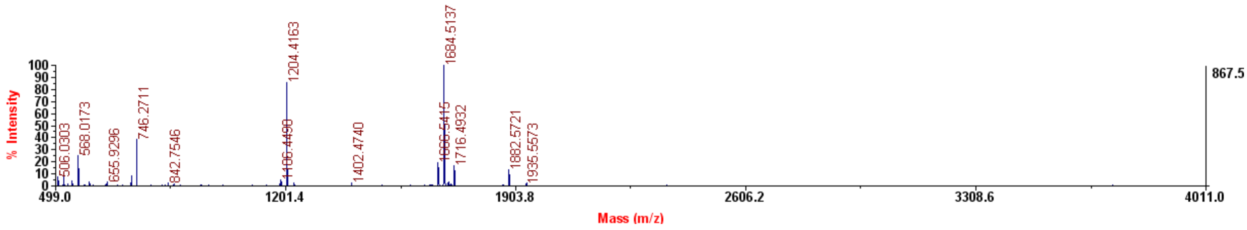

plate 1/line H/column 7

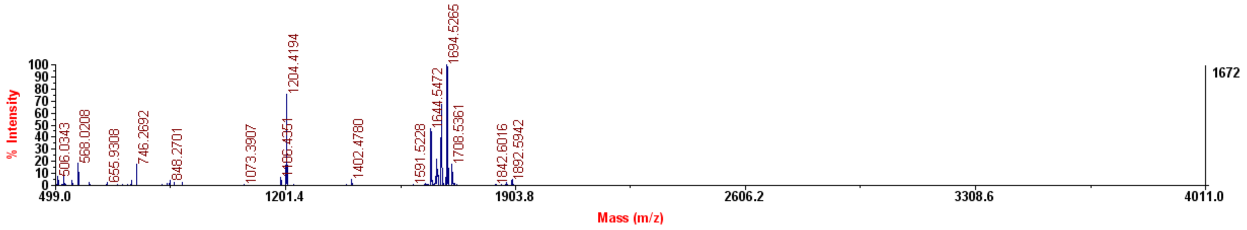

plate 1/line A/column 8

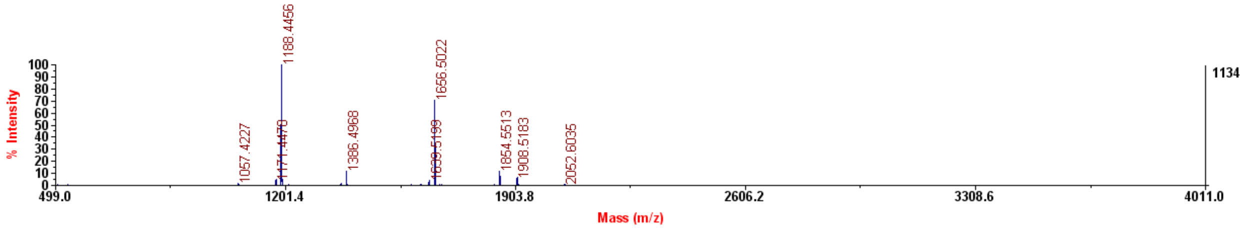

plate 1/line B/column 8

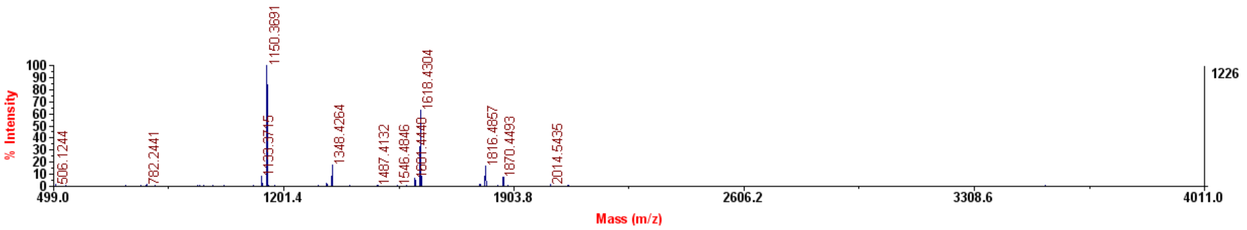

plate 1/line C/column 8

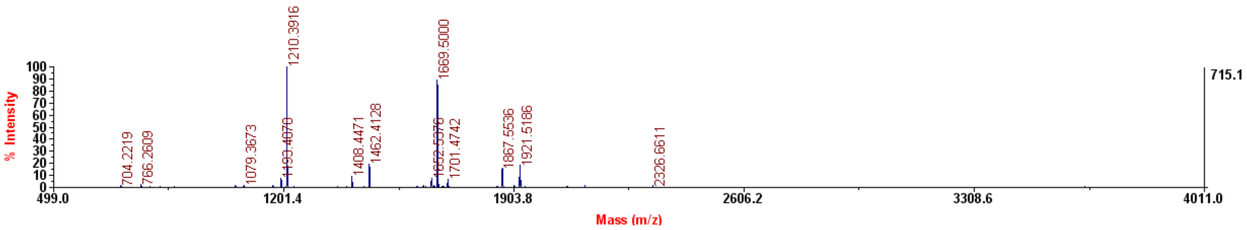

plate 1/line D/column 8

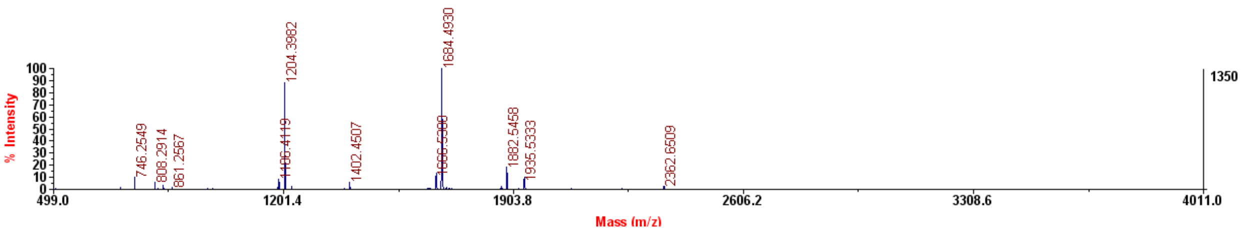

Supplementary Figure 182. MS spectra of plate 1. The data of G7, H7, and A8–D8 are shown.

plate 1/line E/column 8

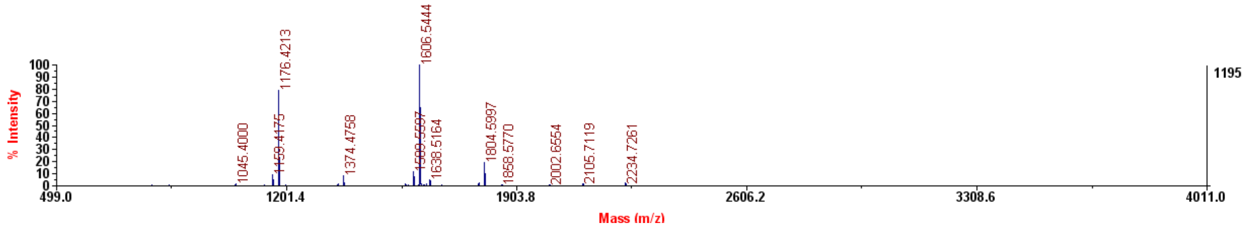

plate 1/line F/column 8

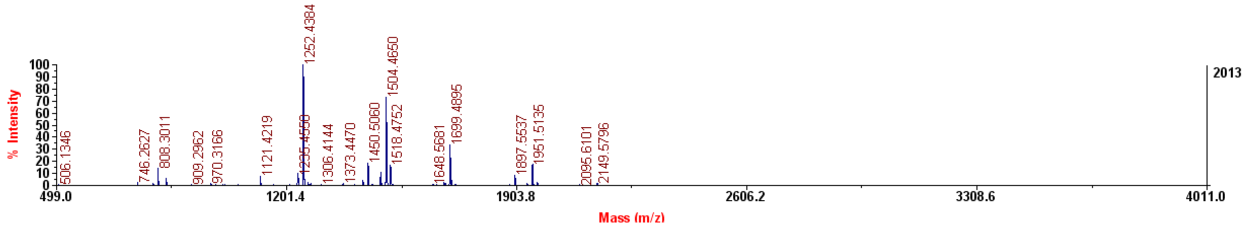

plate 1/line G/column 8

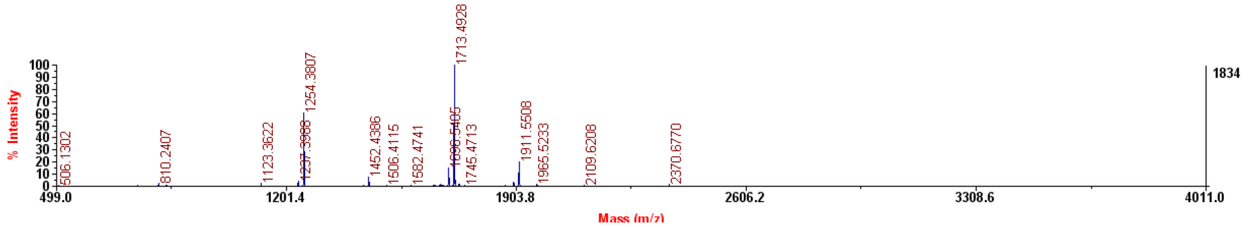

plate 1/line H/column 8

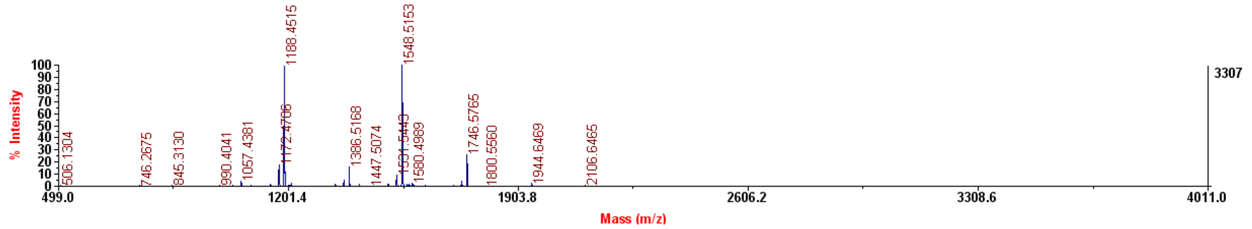

plate 1/line A/column 9

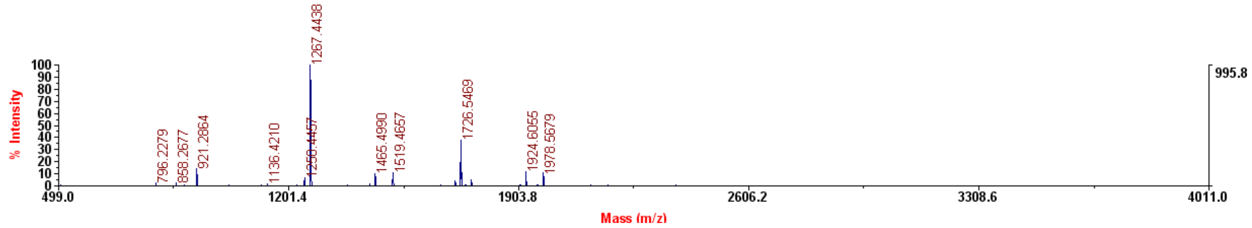

plate 1/line B/column 9

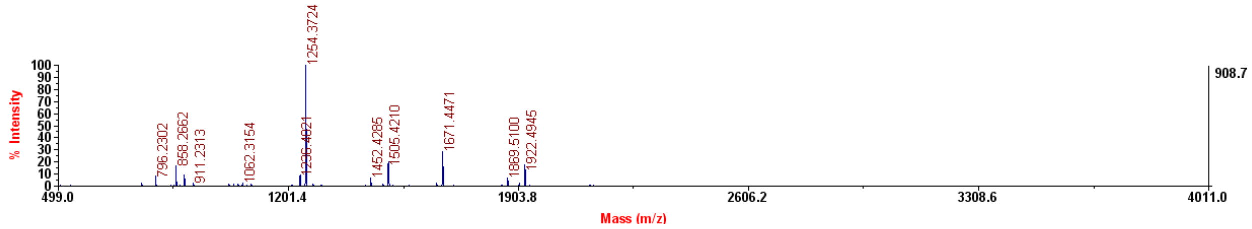

Supplementary Figure 183. MS spectra of plate 1. The data of E8–H8, A9, and B9 are shown.

plate 1/line C/column 9

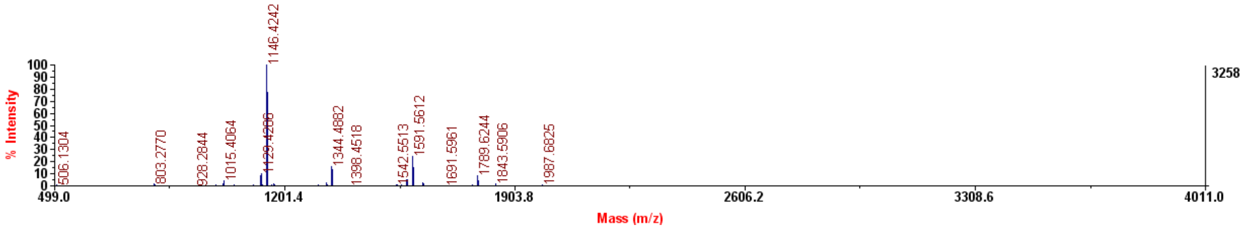

plate 1/line D/column 9

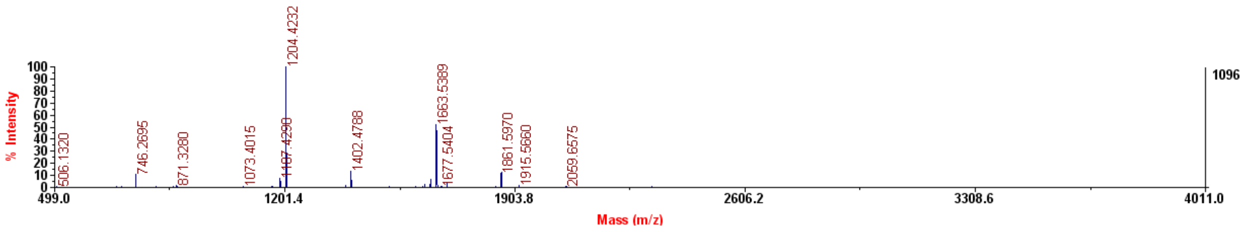

plate 1/line E/column 9

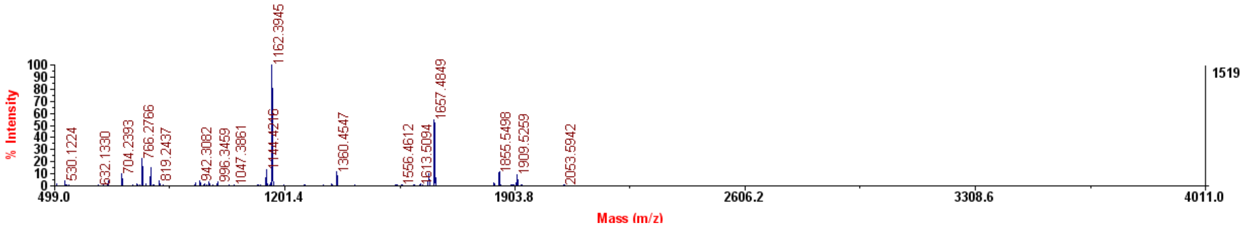

plate 1/line F/column 9

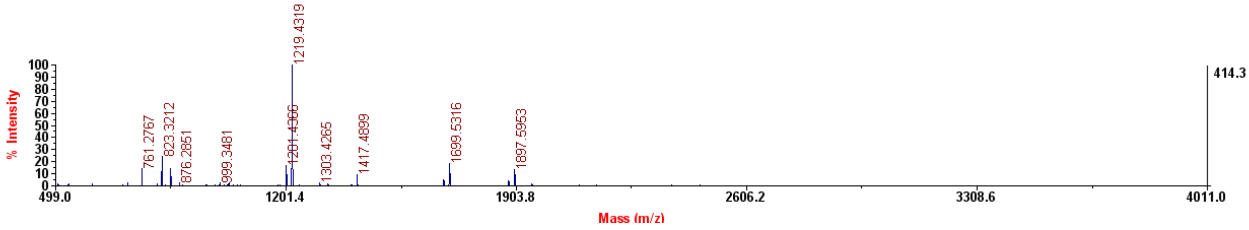

plate 1/line G/column 9

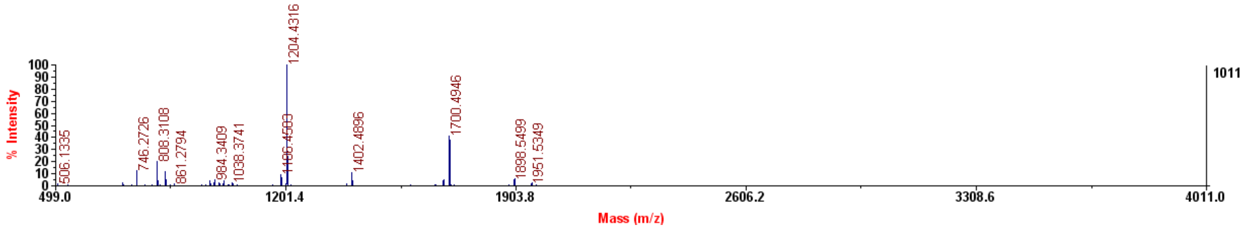

plate 1/line H/column 9

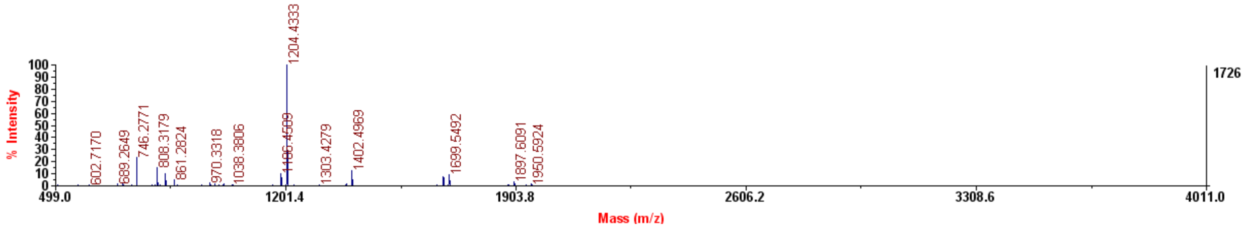

Supplementary Figure 184. MS spectra of plate 1. The data of C9–H9 are shown.

plate 1/line A/column 10

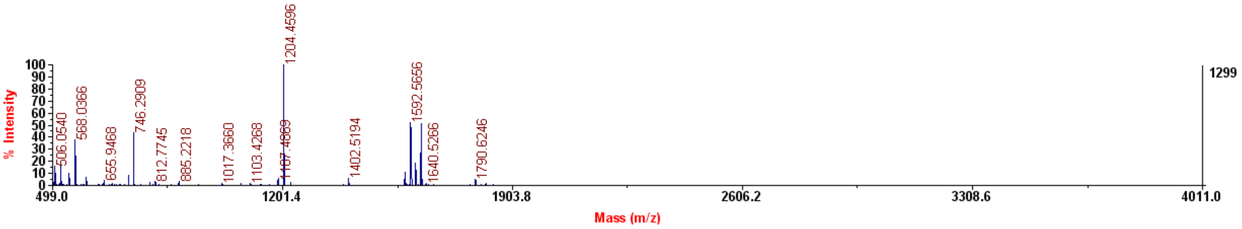

plate 1/line B/column 10

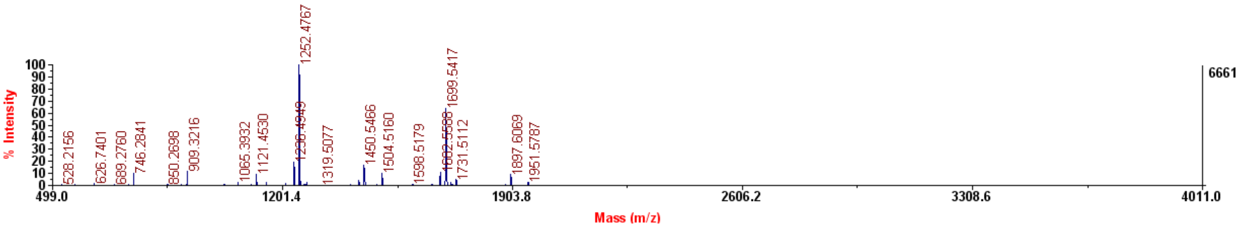

plate 1/line C/column 10

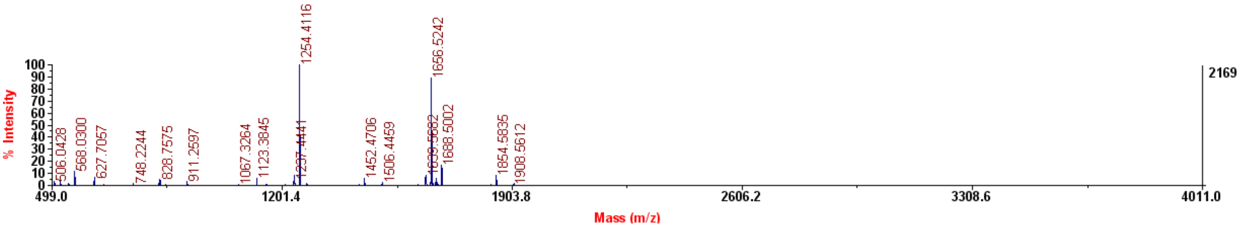

plate 1/line D/column 10

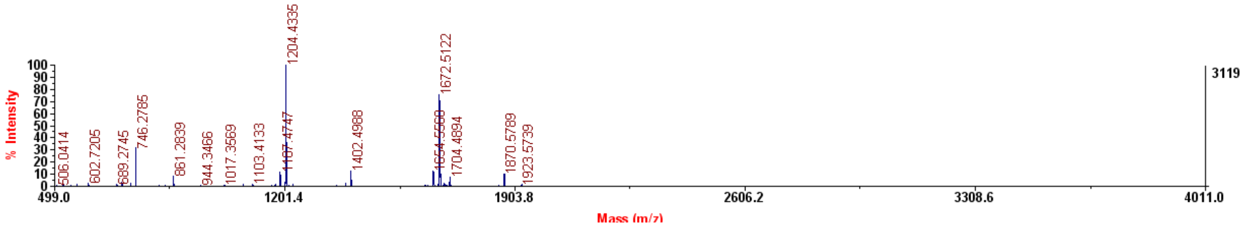

plate 1/line E/column 10

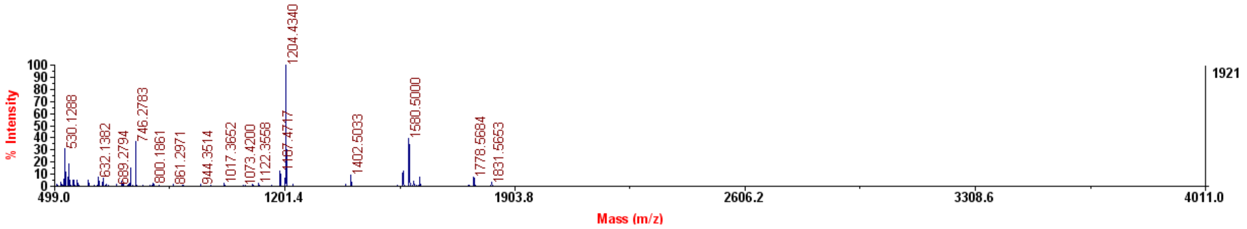

plate 1/line F/column 10

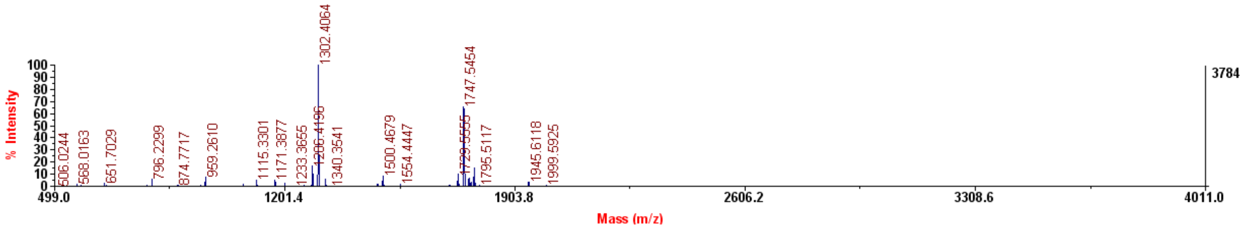

Supplementary Figure 185. MS spectra of plate 1. The data of A10–F10 are shown.

plate 1/line G/column 10

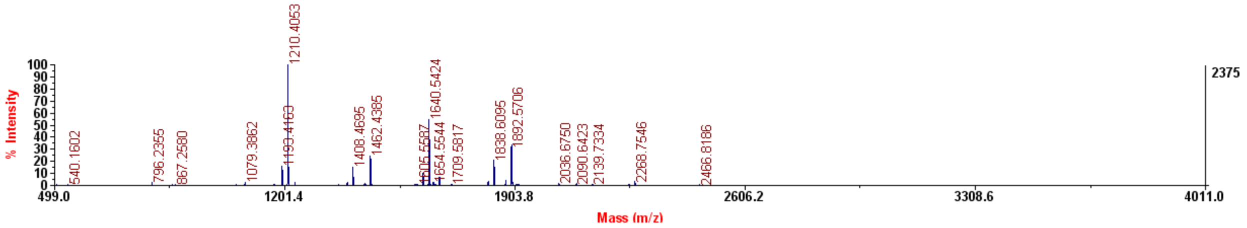

plate 1/line H/column 10

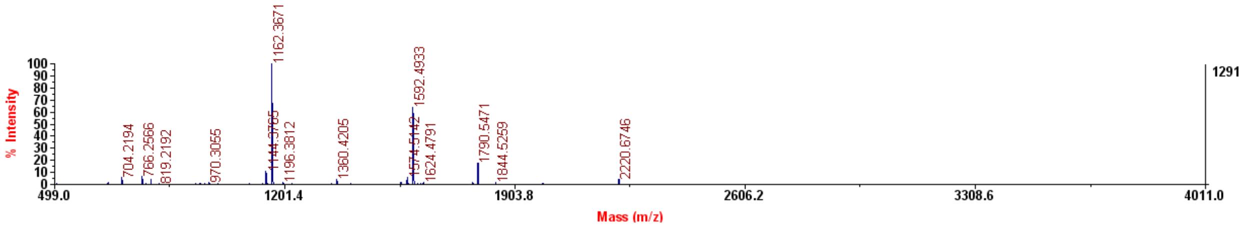

plate 1/line A/column 11

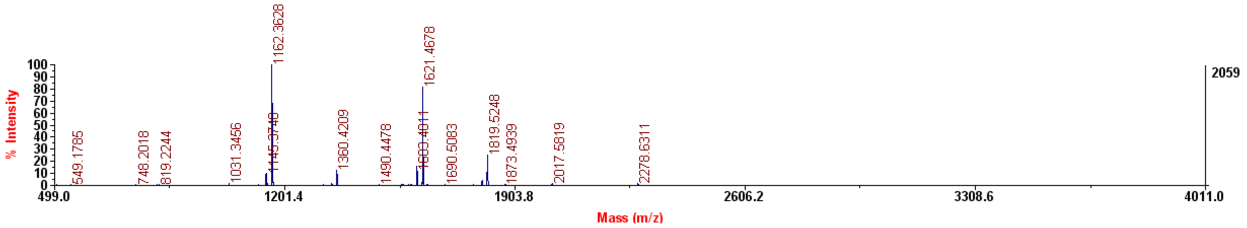

plate 1/line B/column 11

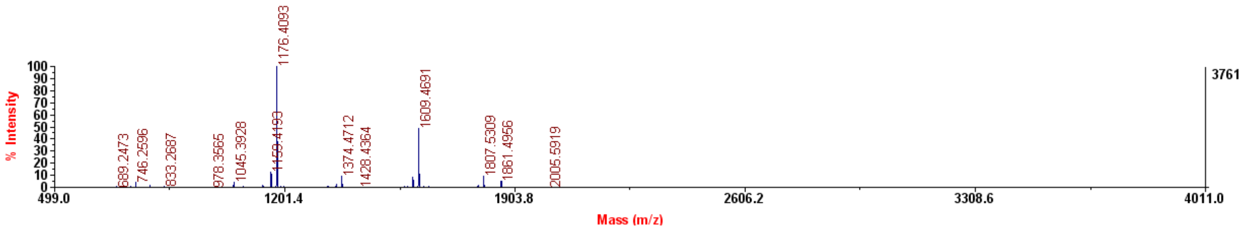

plate 1/line C/column 11

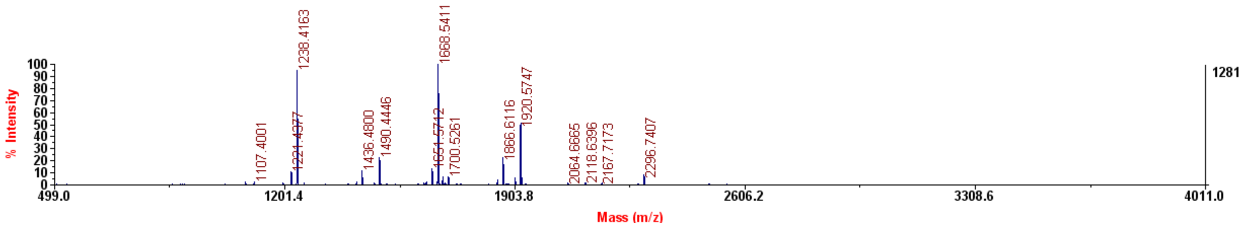

plate 1/line D/column 11

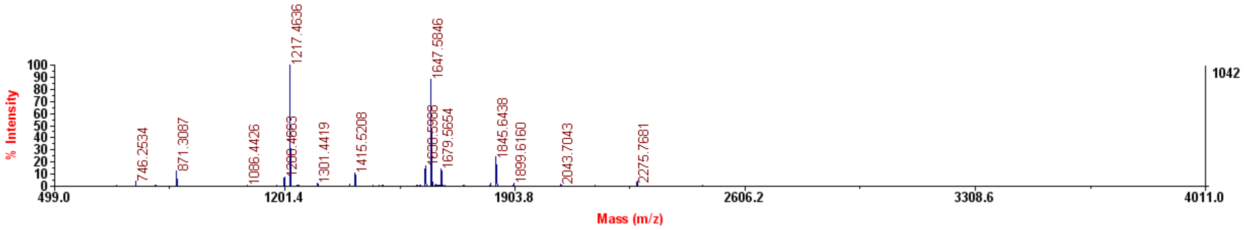

Supplementary Figure 186. MS spectra of plate 1. The data of G10, H10, and A11–D11 are shown.

plate 1/line E/column 11

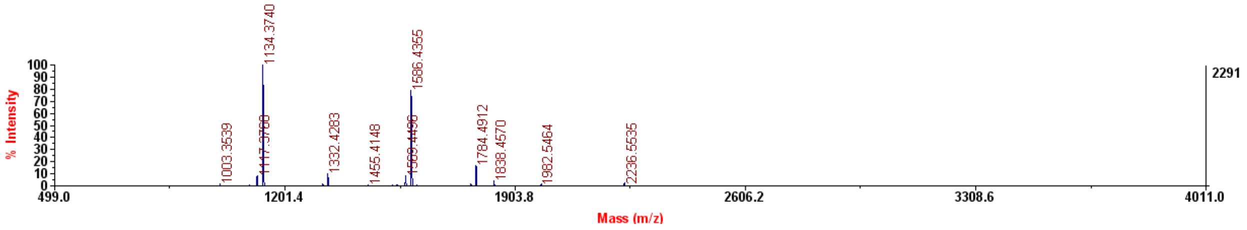

plate 1/line F/column 11

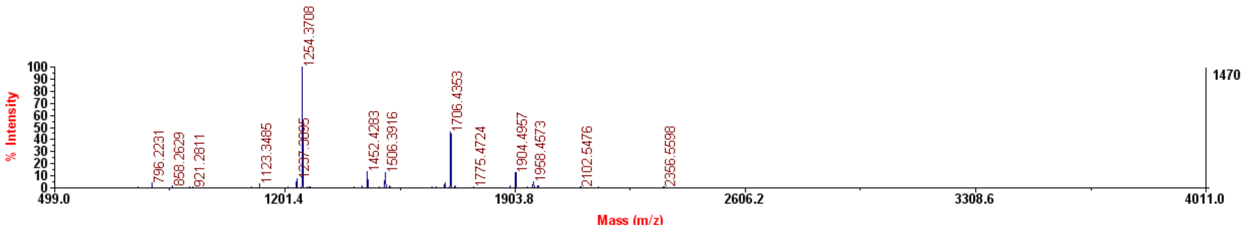

plate 1/line G/column 11

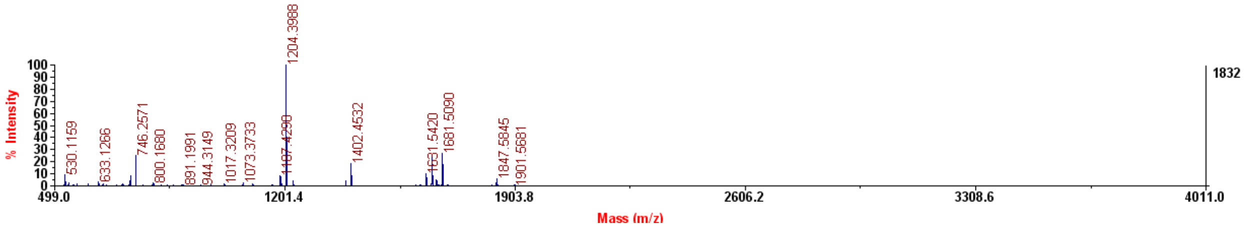

plate 1/line H/column 11

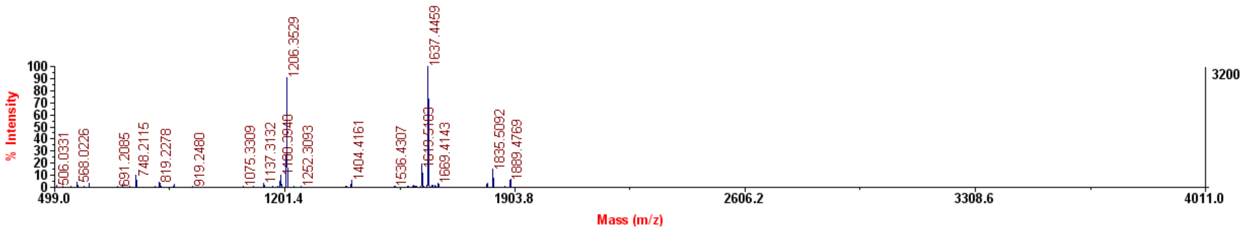

Supplementary Figure 187. MS spectra of plate 1. The data of E11–H11 are shown.

plate 2/line A/column 1

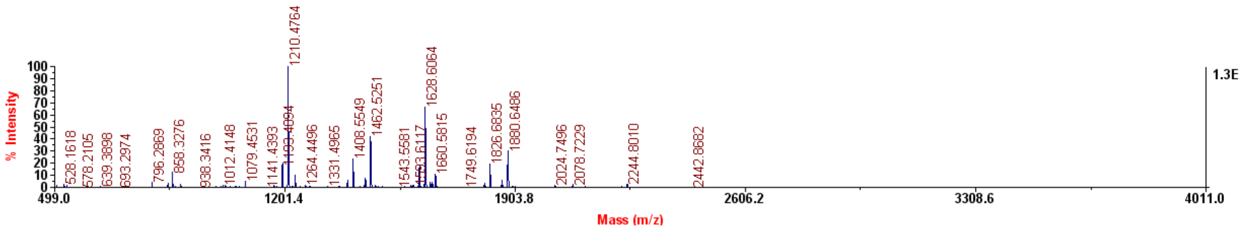

plate 2/line B/column 1

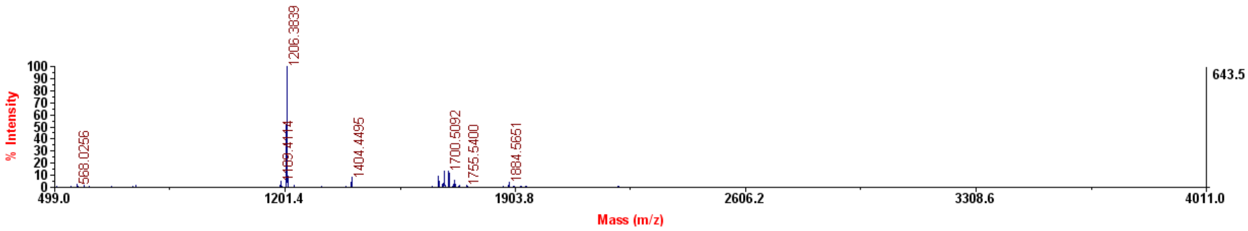

plate 2/line C/column 1

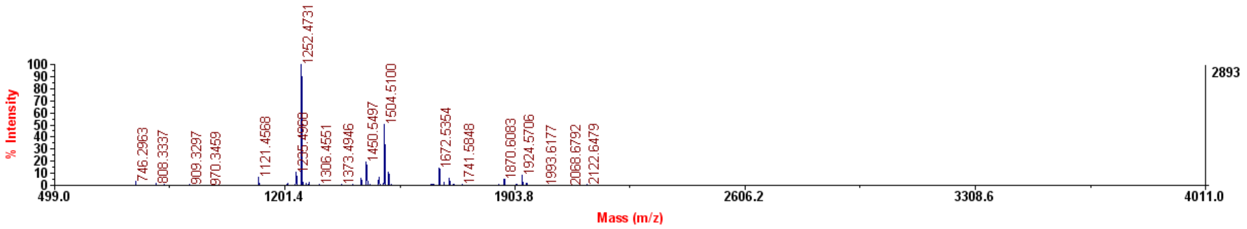

plate 2/line D/column 1

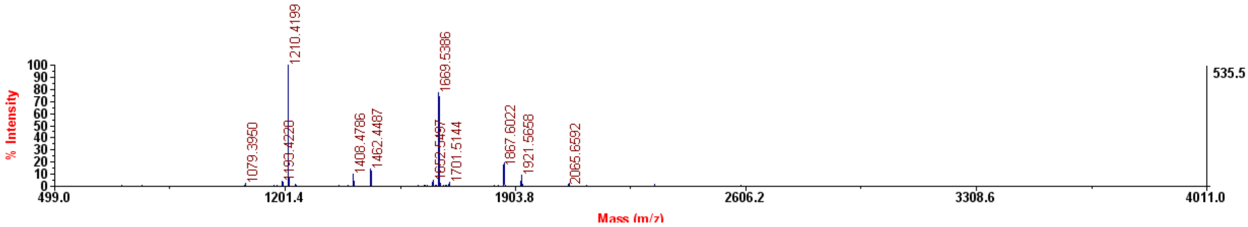

plate 2/line E/column 1

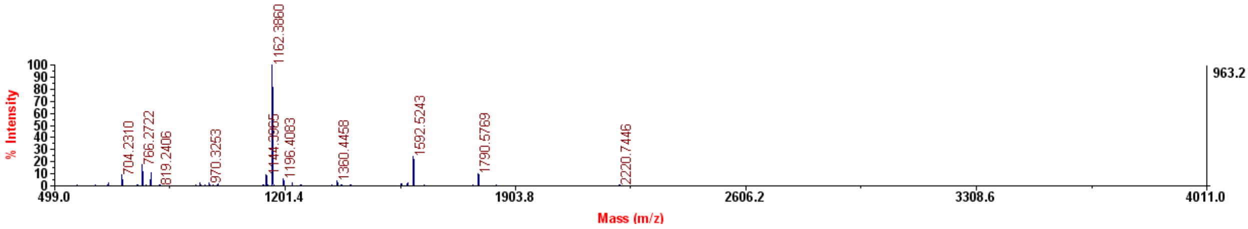

plate 2/line F/column 1

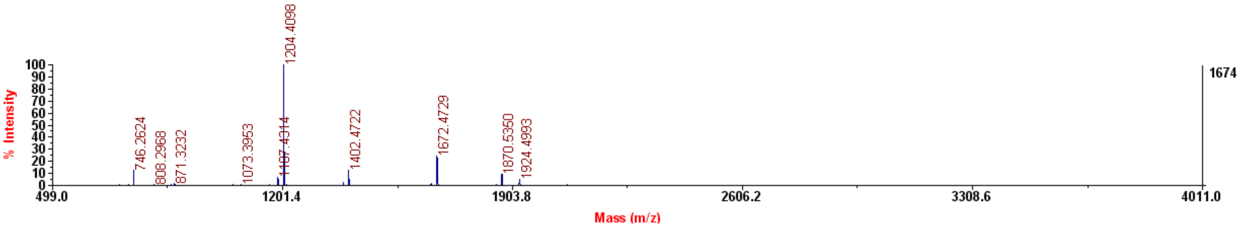

Supplementary Figure 188. MS spectra of plate 2. The data of A1–F1 are shown.

plate 2/line G/column 1

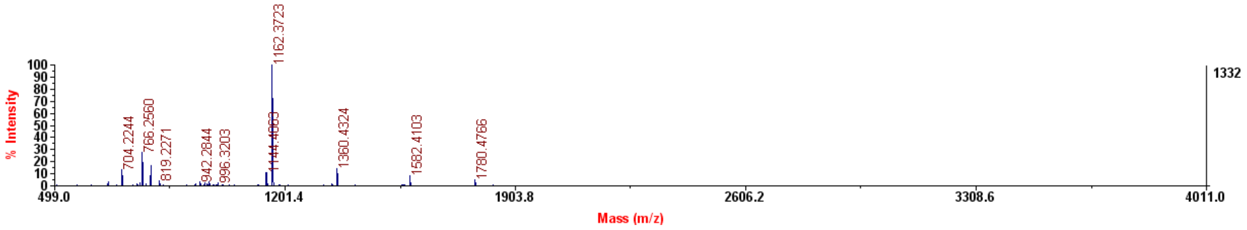

plate 2/line H/column 1

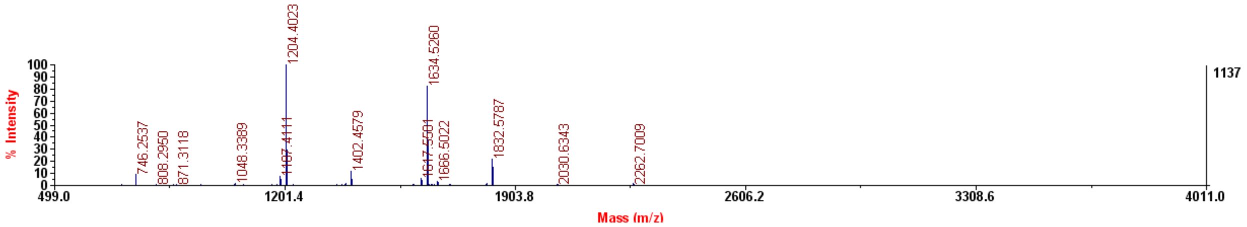

plate 2/line A/column 2

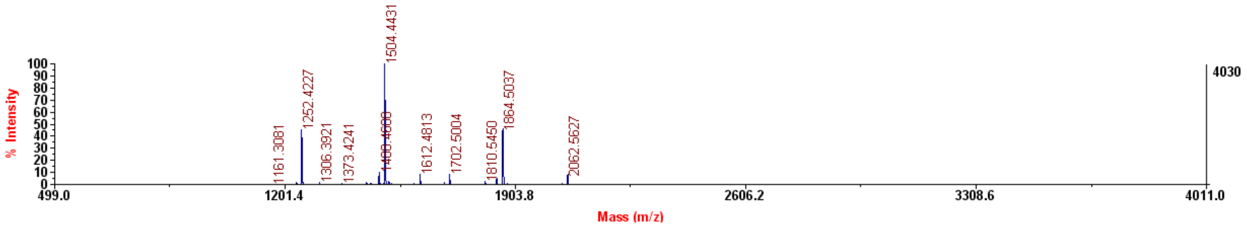

plate 2/line B/column 2

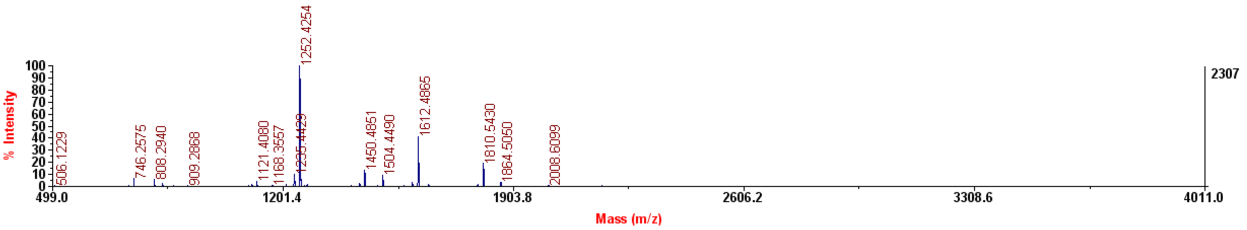

plate 2/line C/column 2

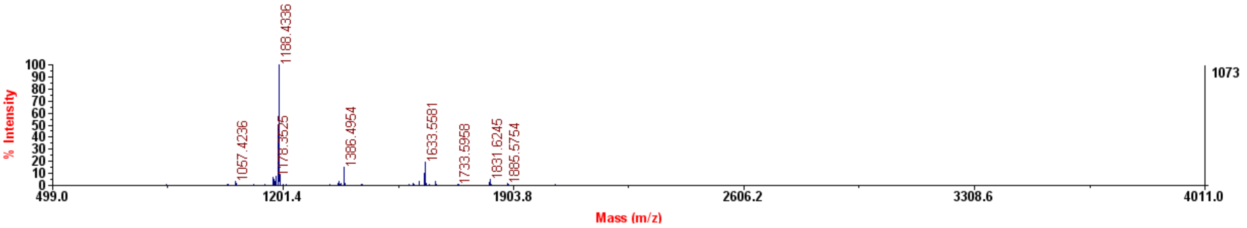

plate 2/line D/column 2

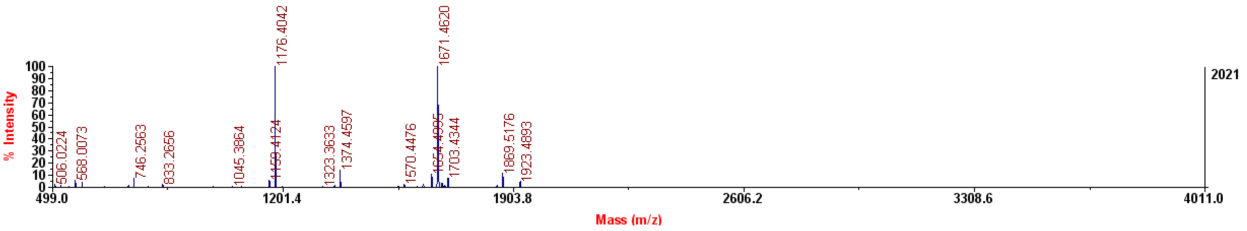

Supplementary Figure 189. MS spectra of plate 2. The data of G1, H1, and A2–D2 are shown.

plate 2/line E/column 2

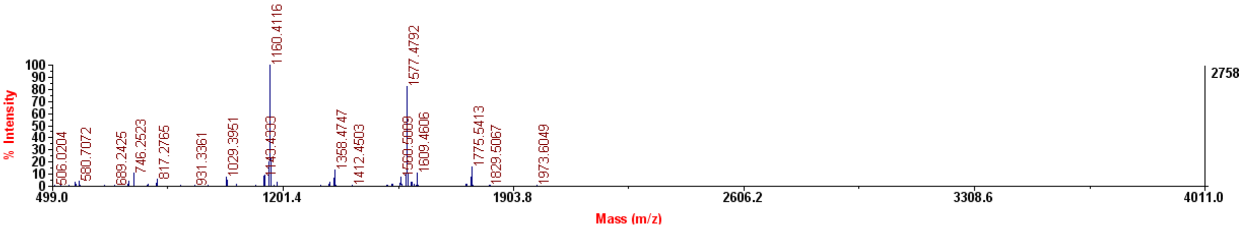

plate 2/line F/column 2

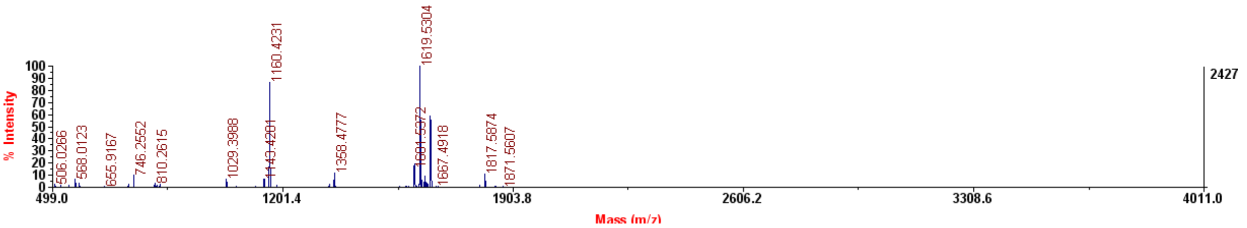

plate 2/line G/column 2

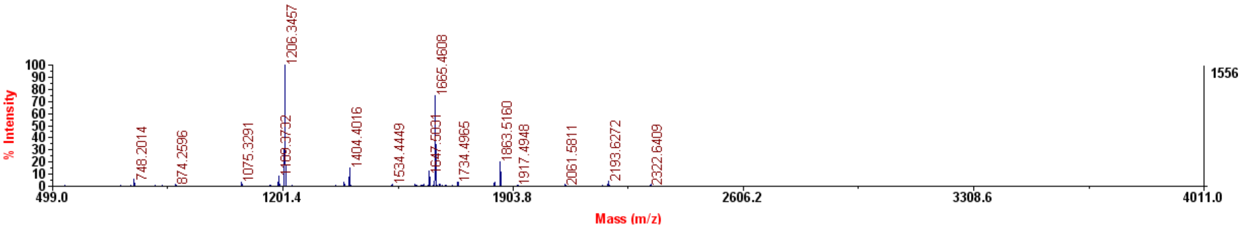

plate 2/line H/column 2

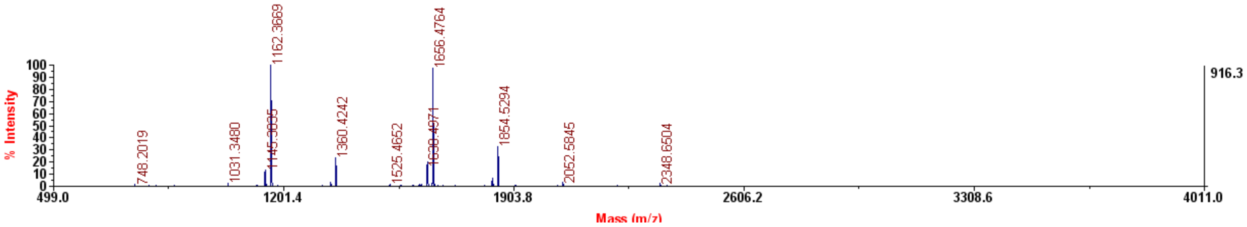

plate 2/line A/column 3

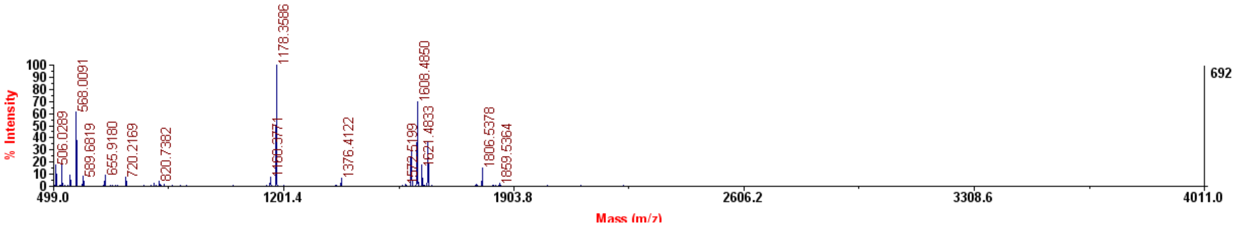

plate 2/line B/column 3

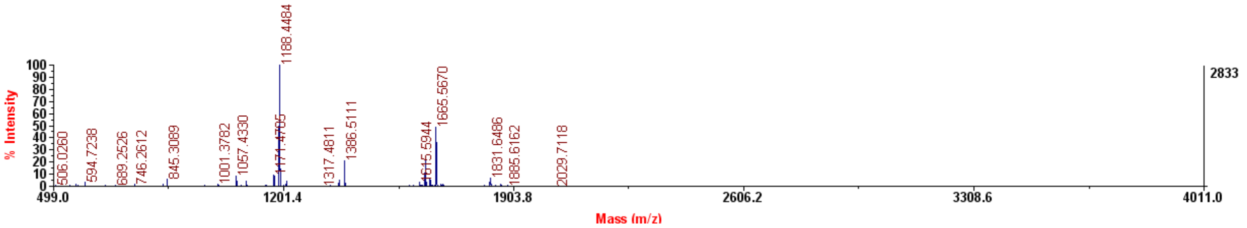

Supplementary Figure 190. MS spectra of plate 2. The data of E2–H2, A3, and B3 are shown.

plate 2/line C/column 3

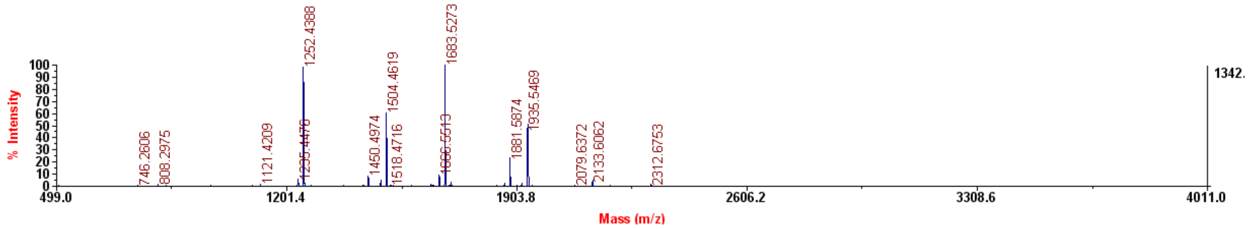

plate 2/line D/column 3

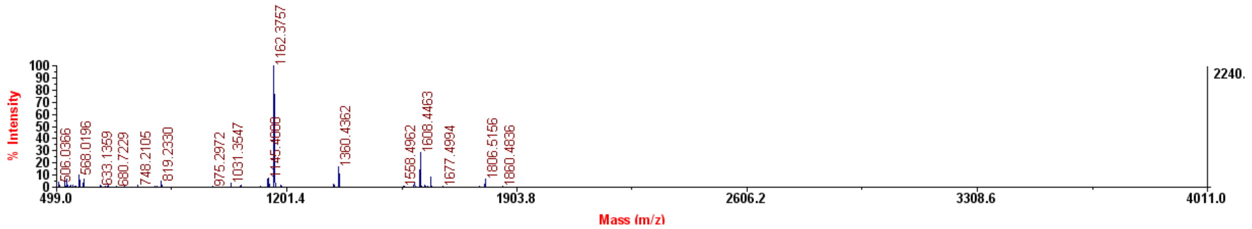

plate 2/line E/column 3

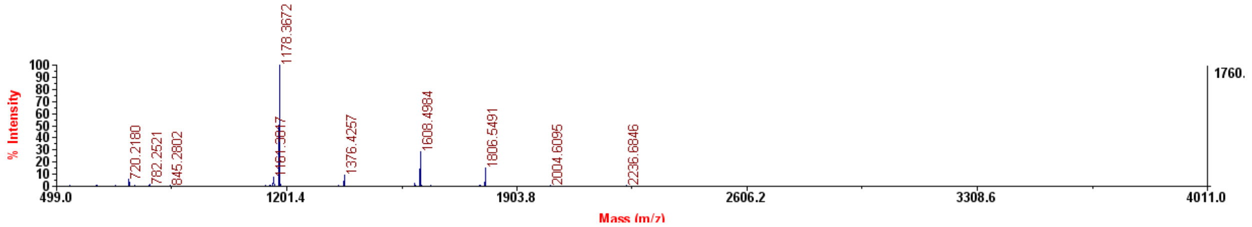

plate 2/line F/column 3

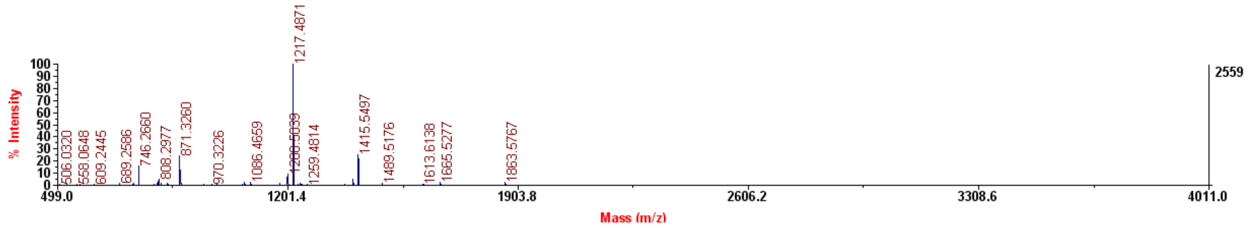

plate 2/line G/column 3

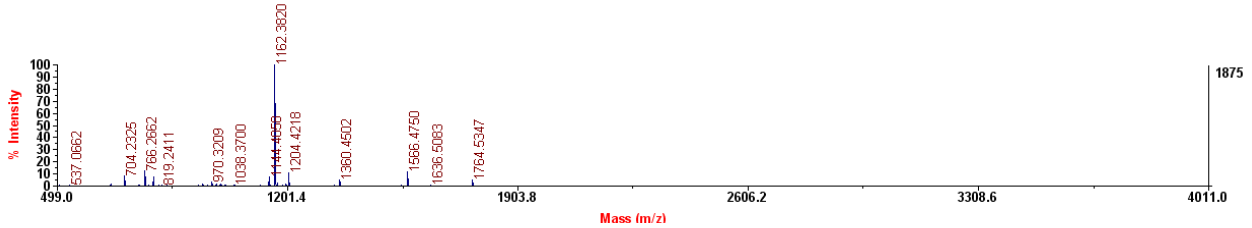

plate 2/line H/column 3

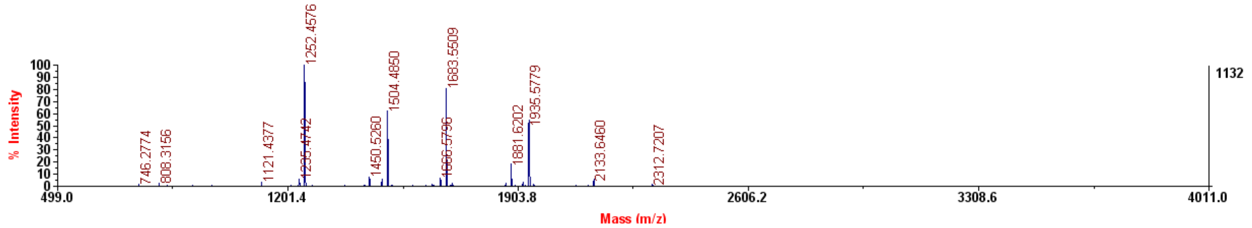

Supplementary Figure 191. MS spectra of plate 2. The data of C3–H3 are shown.

plate 2/line A/column 4

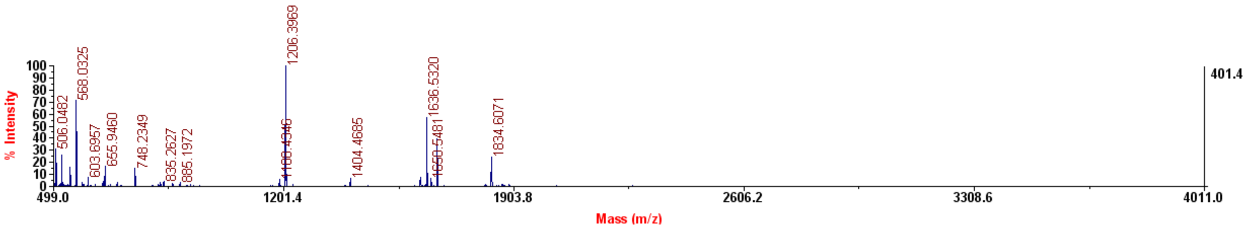

plate 2/line B/column 4

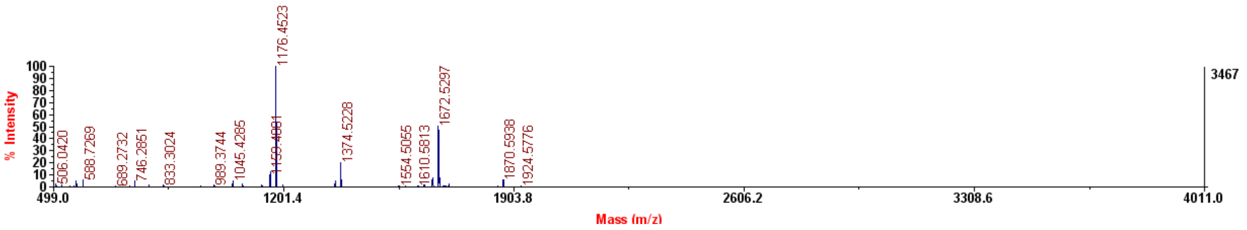

plate 2/line C/column 4

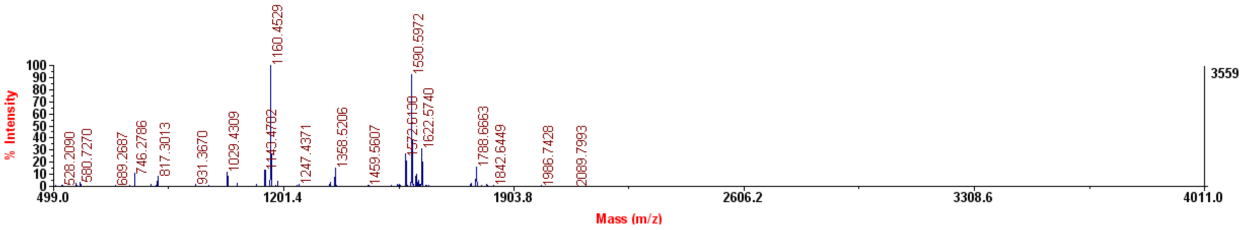

plate 2/line D/column 4

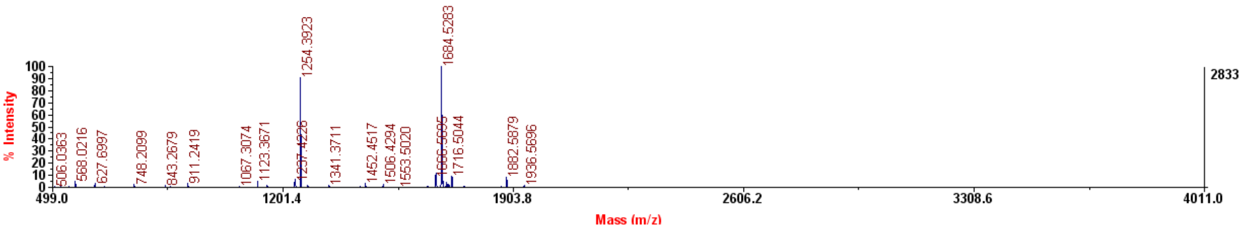

plate 2/line E/column 4

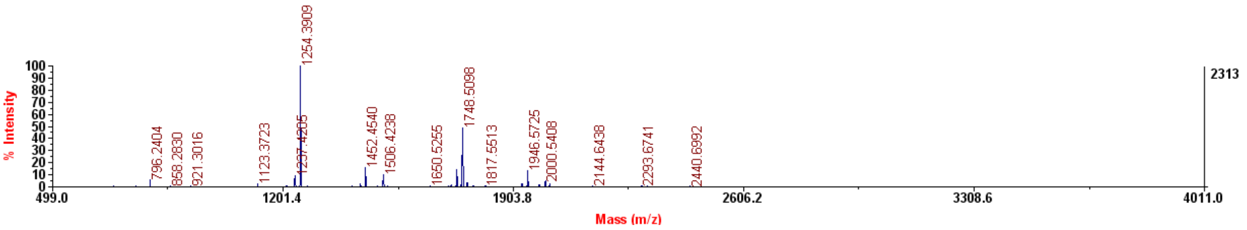

plate 2/line F/column 4

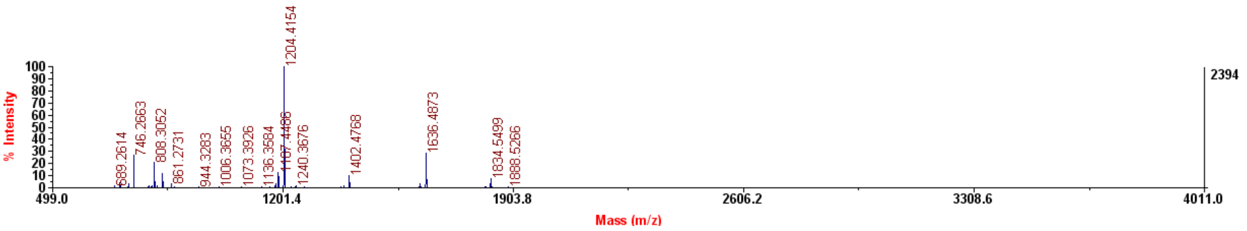

Supplementary Figure 192. MS spectra of plate 2. The data of A4–F4 are shown.

plate 2/line G/column 4

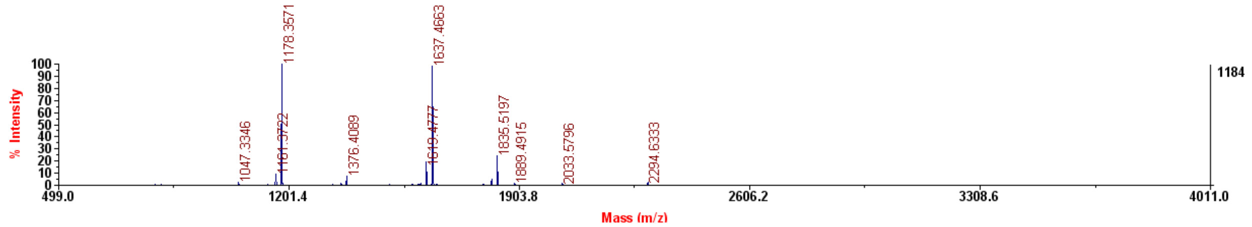

plate 2/line H/column 4

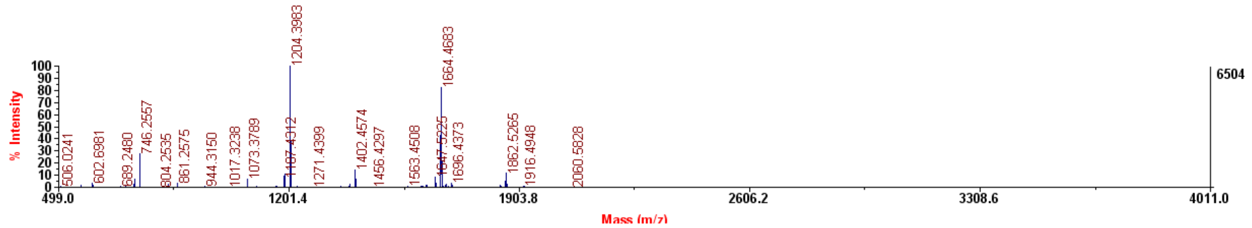

plate 2/line A/column 5

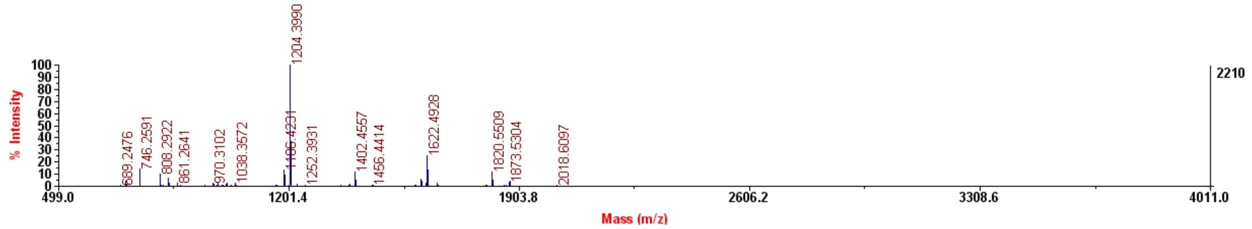

plate 2/line B/column 5

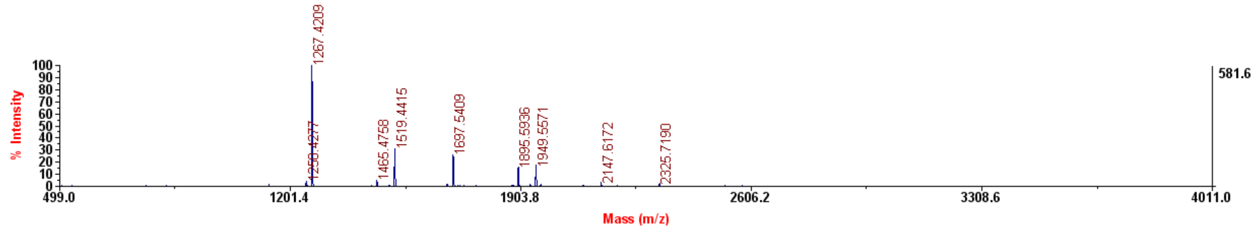

plate 2/line C/column 5

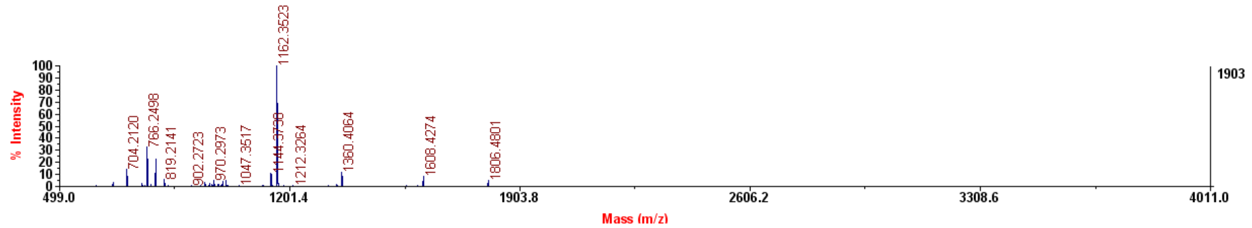

plate 2/line D/column 5

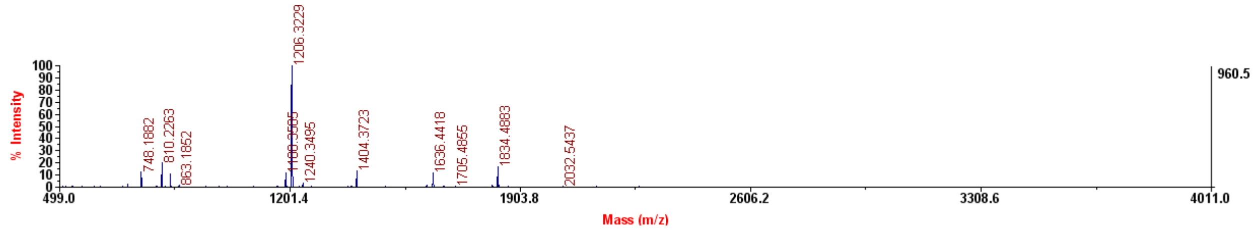

Supplementary Figure 193. MS spectra of plate 2. The data of G4, H4, and A5–D5 are shown.

plate 2/line E/column 5

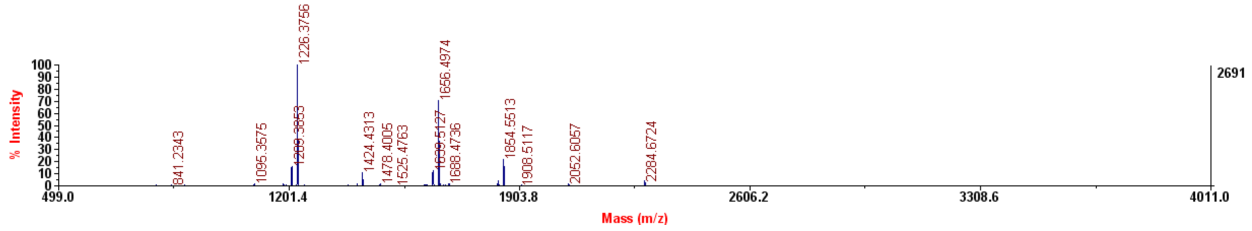

plate 2/line F/column 5

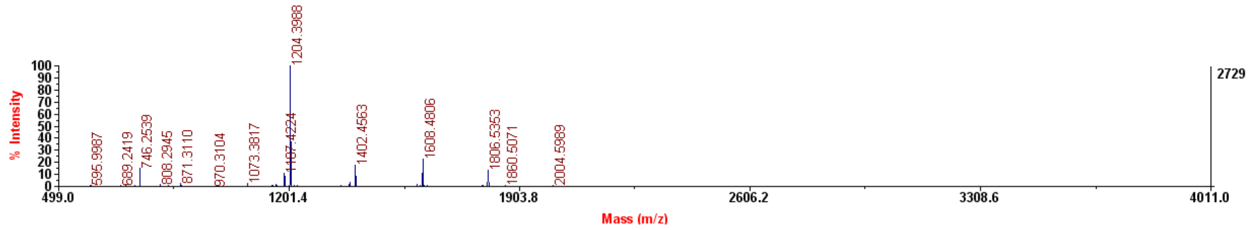

plate 2/line G/column 5

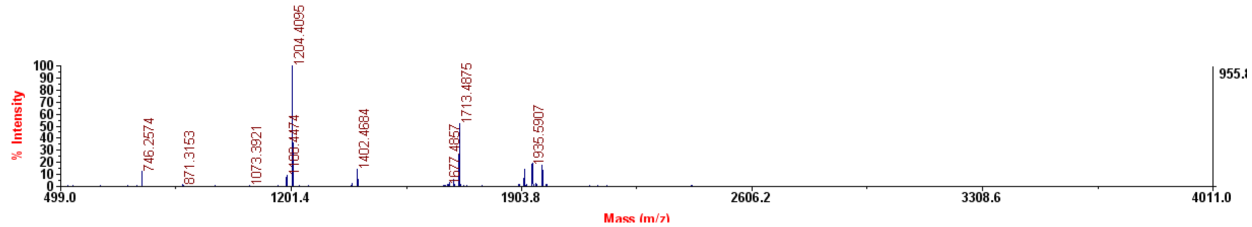

plate 2/line H/column 5

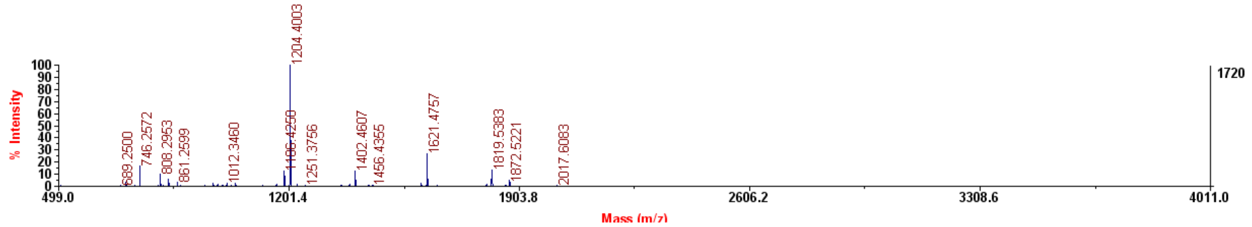

plate 2/line A/column 6

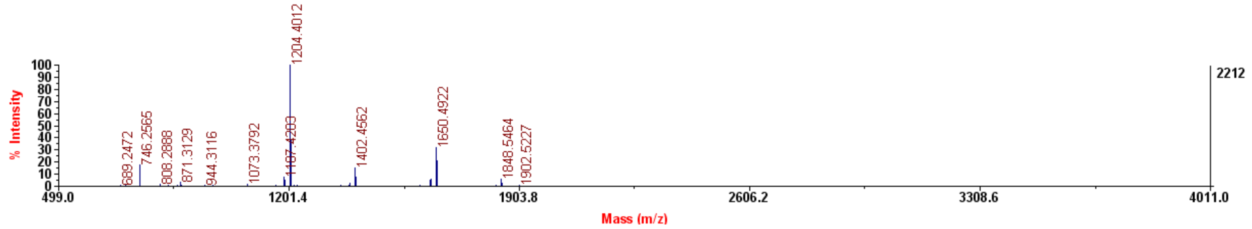

plate 2/line B/column 6

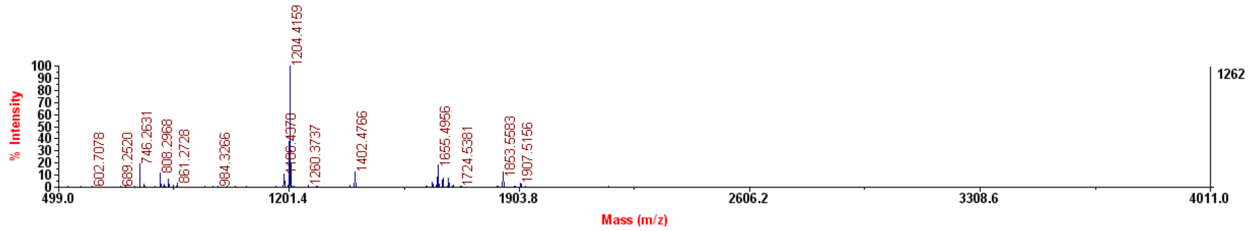

Supplementary Figure 194. MS spectra of plate 2. The data of E5–H5, A6, and B6 are shown.

plate 2/line C/column 6

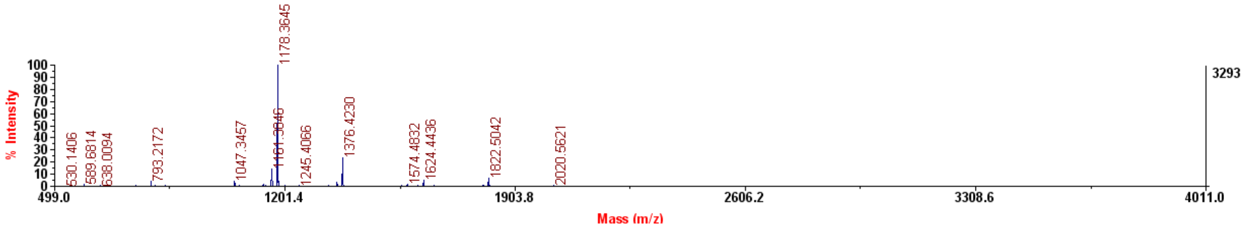

plate 2/line D/column 6

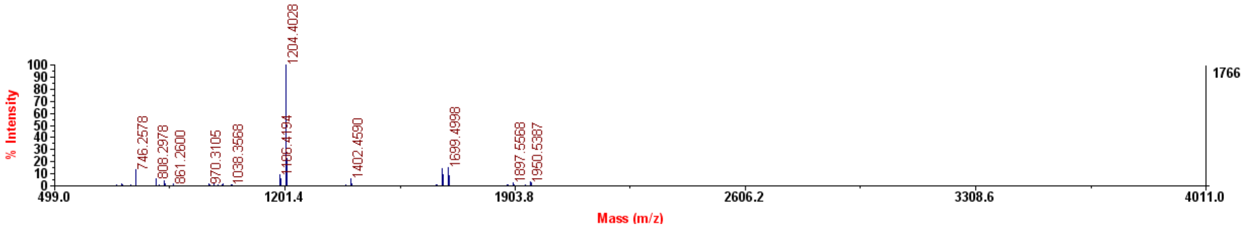

plate 2/line E/column 6

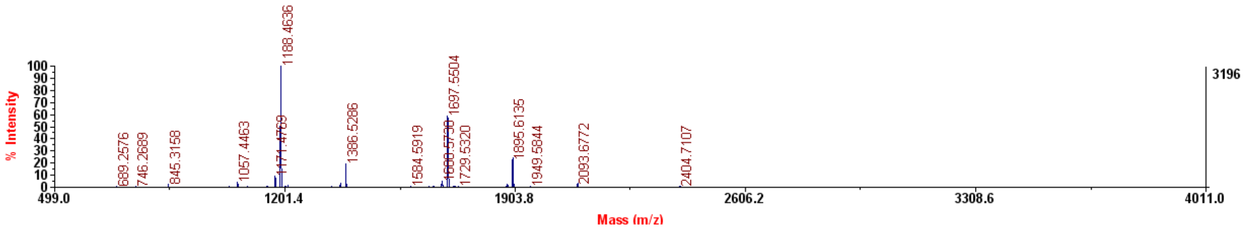

plate 2/line F/column 6

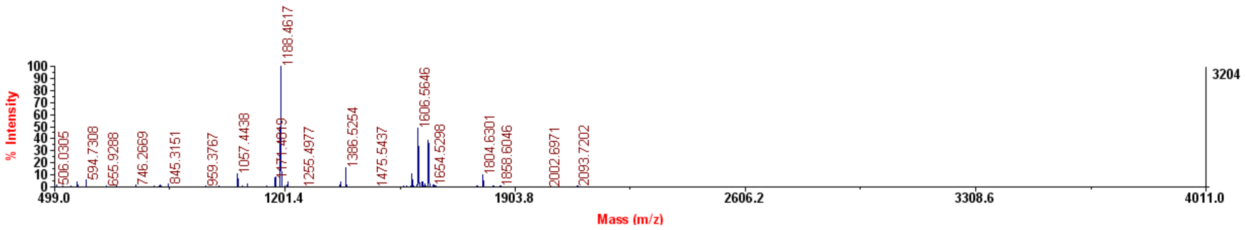

plate 2/line G/column 6

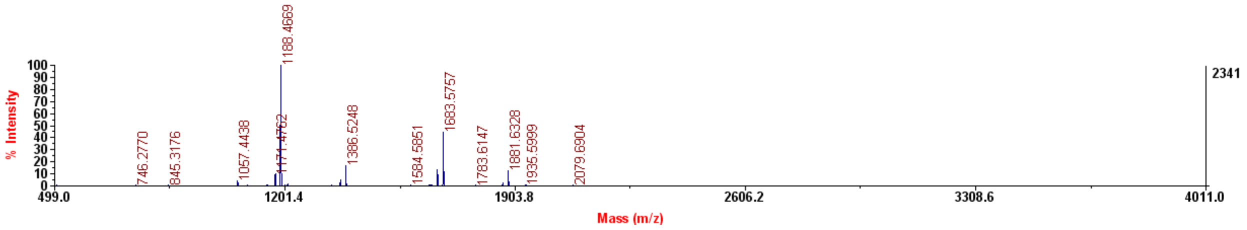

plate 2/line H/column 6

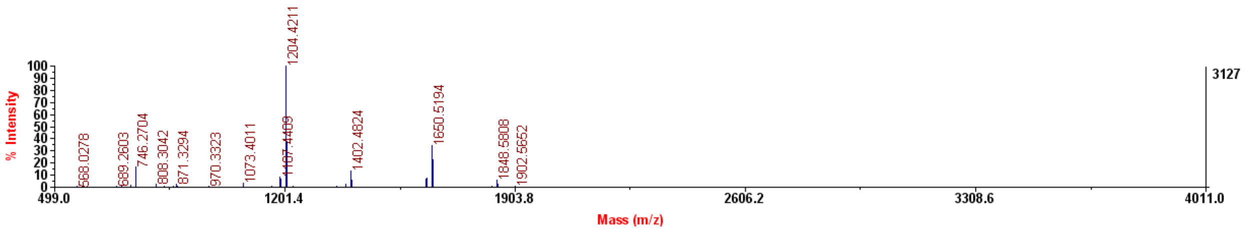

Supplementary Figure 195. MS spectra of plate 2. The data of C6–H6 are shown.

plate 2/line A/column 7

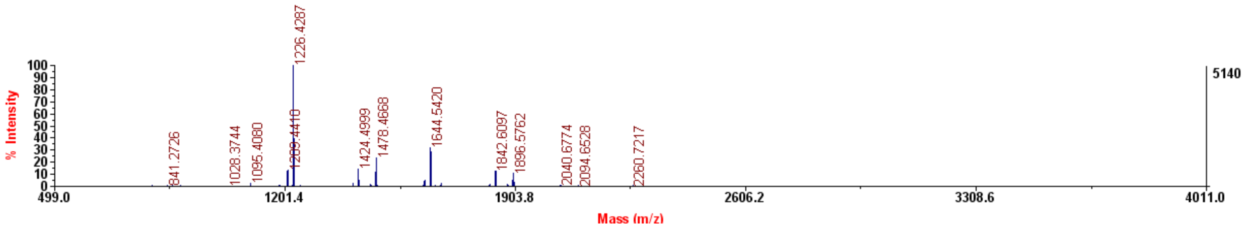

plate 2/line B/column 7

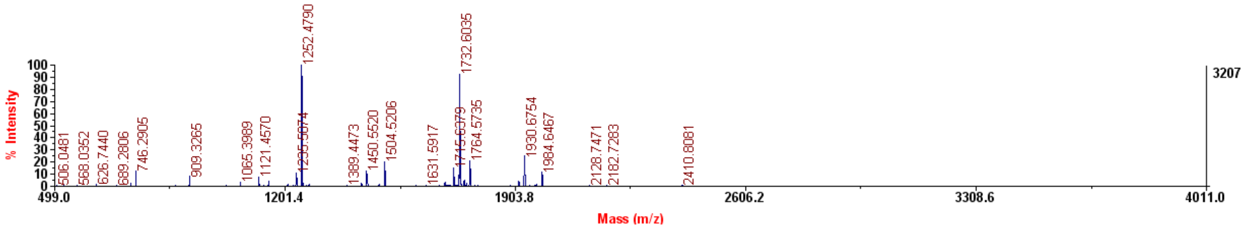

plate 2/line C/column 7

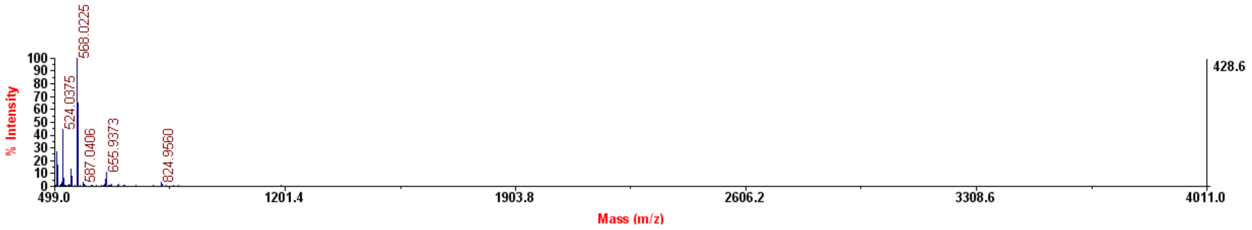

plate 2/line D/column 7

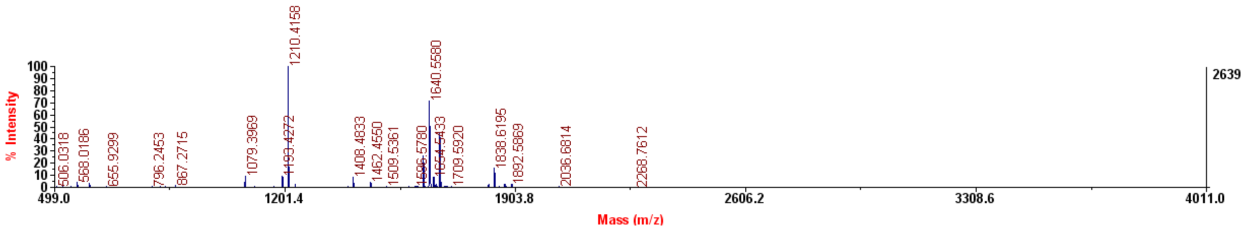

plate 2/line E/column 7

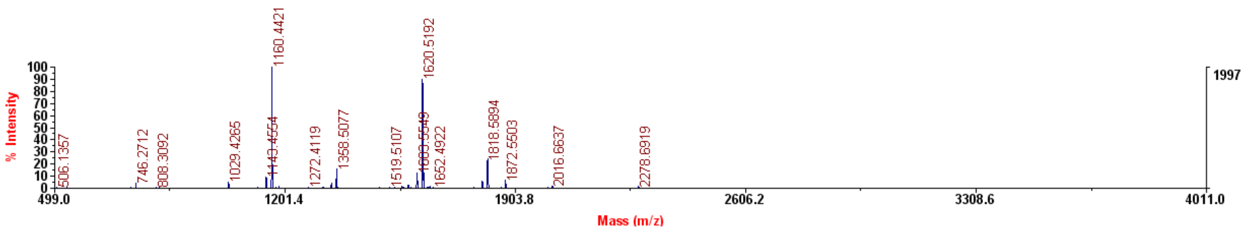

plate 2/line F/column 7

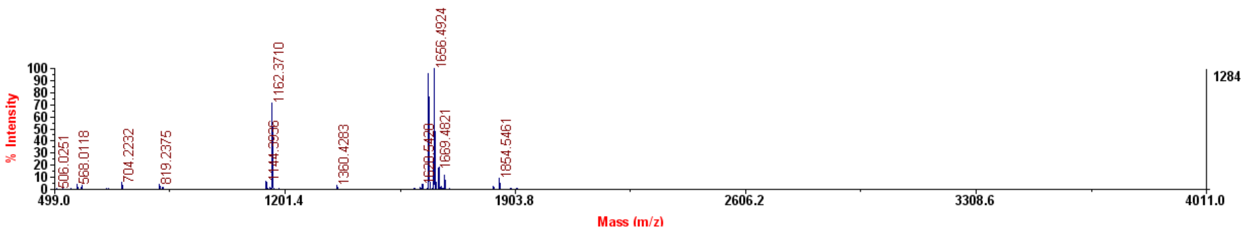

Supplementary Figure 196. MS spectra of plate 2. The data of A7–F7 are shown.

plate 2/line G/column 7

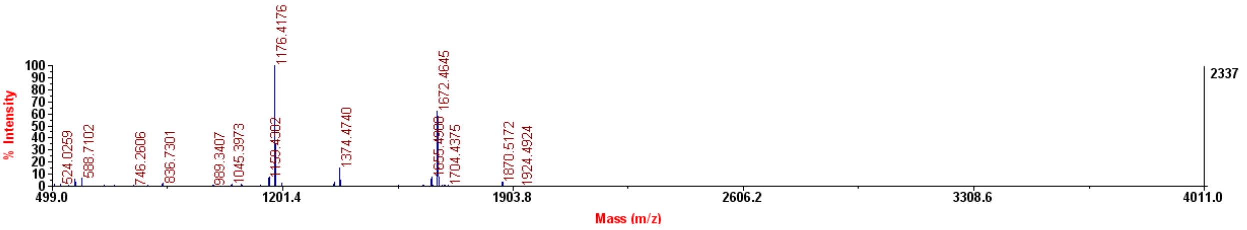

plate 2/line H/column 7

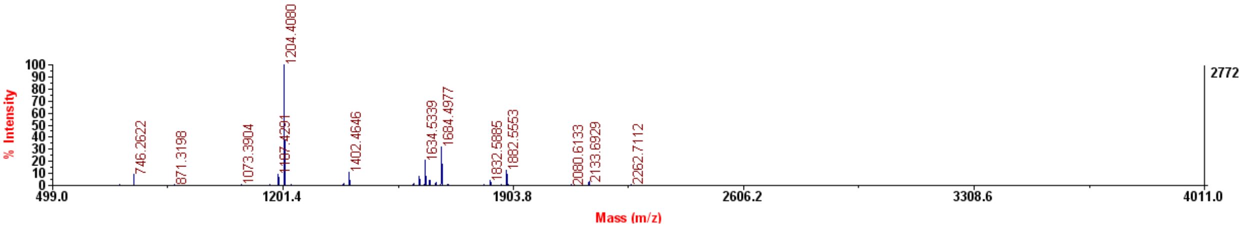

plate 2/line A/column 8

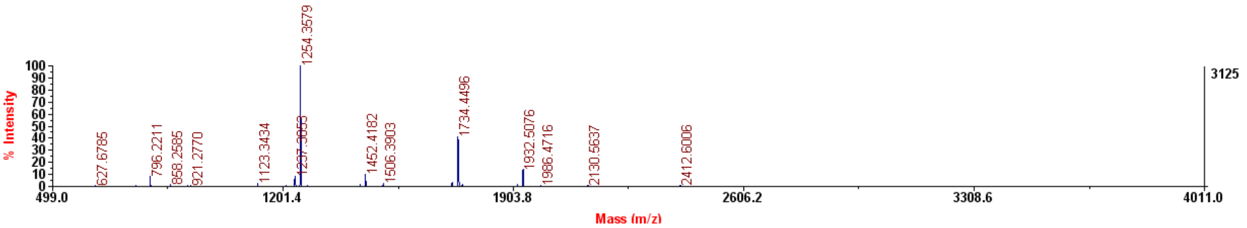

plate 2/line B/column 8

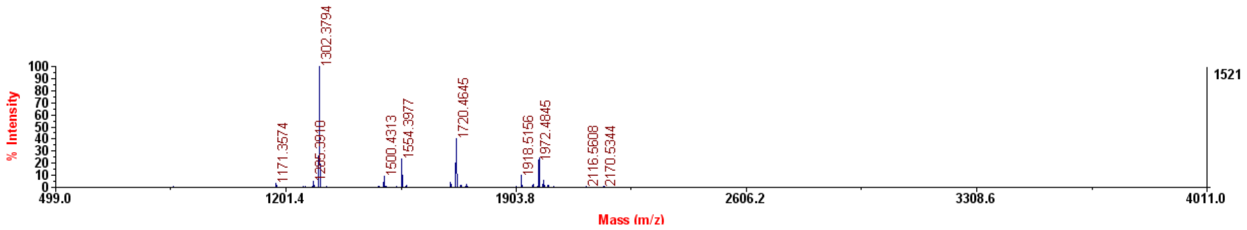

plate 2/line C/column 8

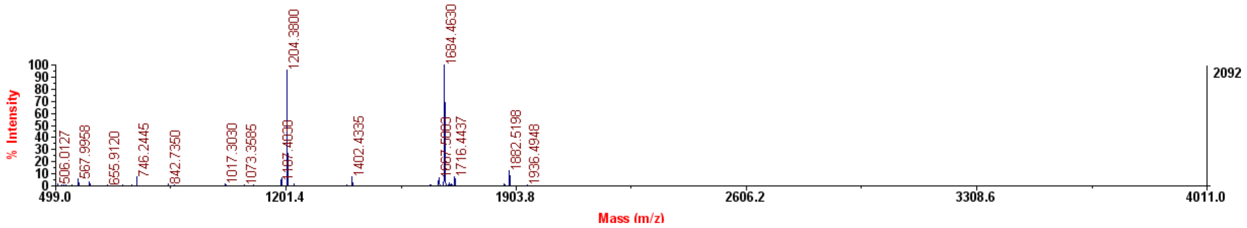

plate 2/line D/column 8

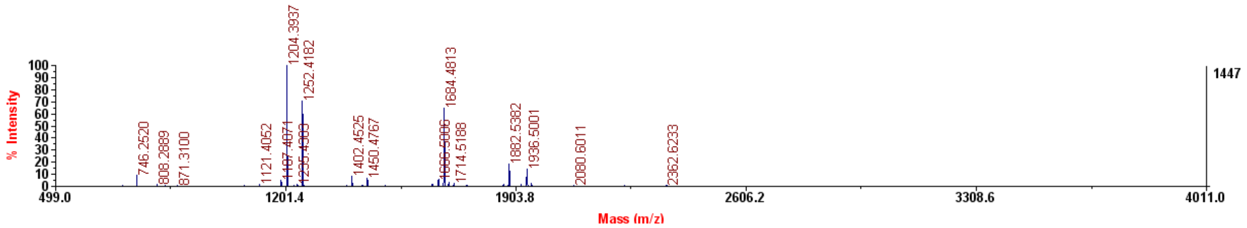

Supplementary Figure 197. MS spectra of plate 2. The data of G7, H7, and A8–D8 are shown.

plate 2/line E/column 8

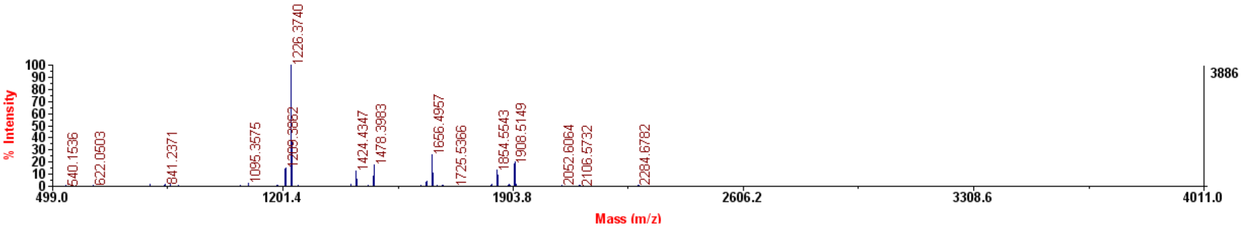

plate 2/line F/column 8

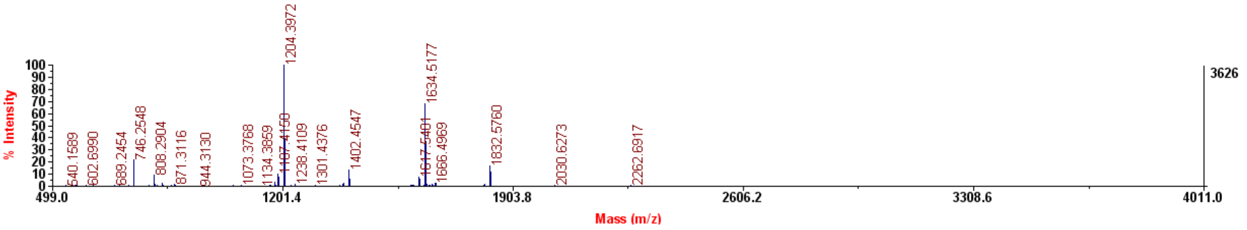

plate 2/line G/column 8

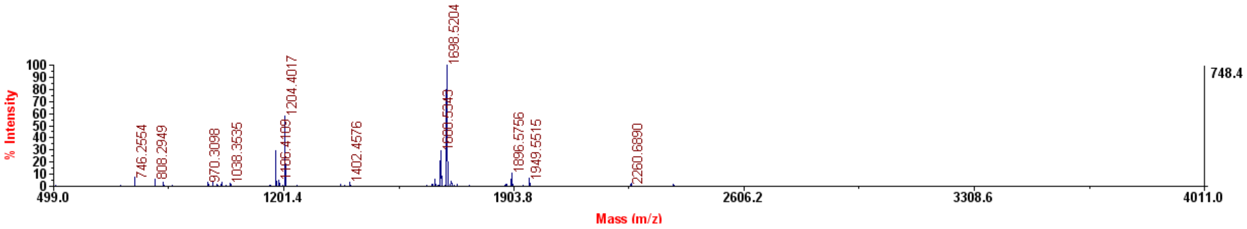

plate 2/line H/column 8

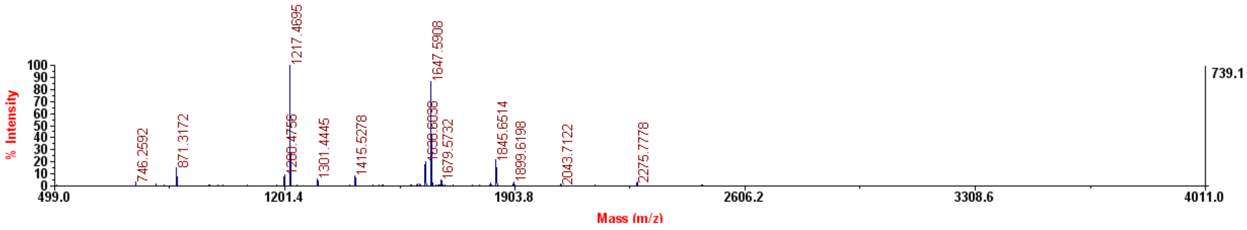

plate 2/line A/column 9

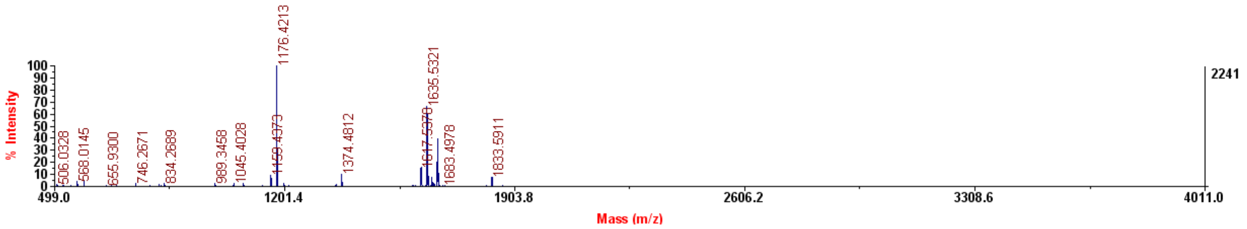

plate 2/line B/column 9

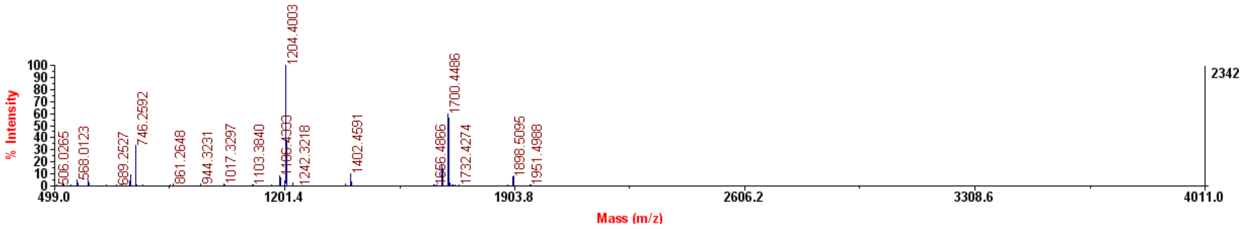

Supplementary Figure 198. MS spectra of plate 2. The data of E8–H8, A9, and B9 are shown.

plate 2/line C/column 9

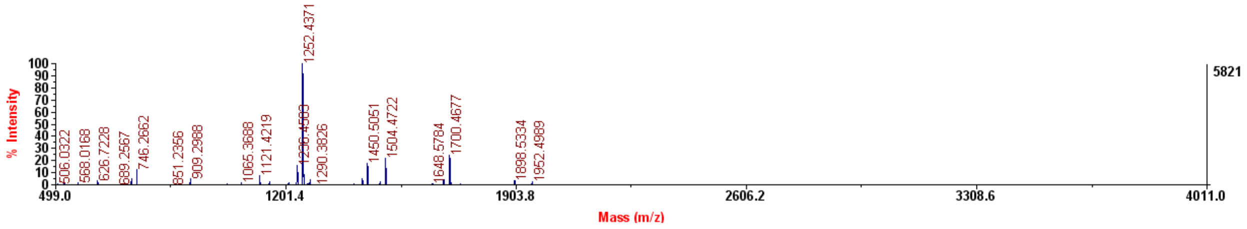

plate 2/line D/column 9

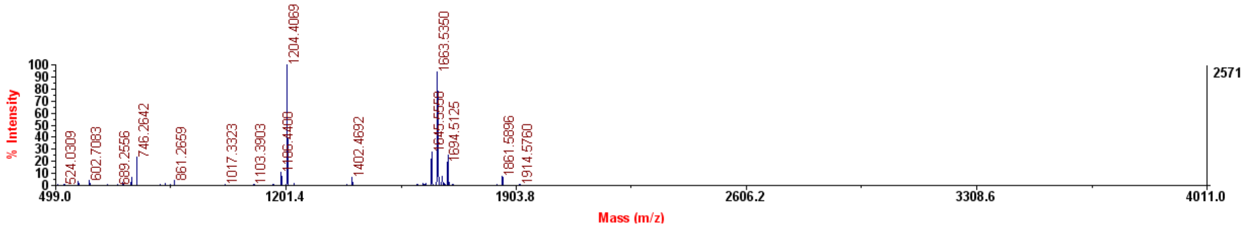

plate 2/line E/column 9

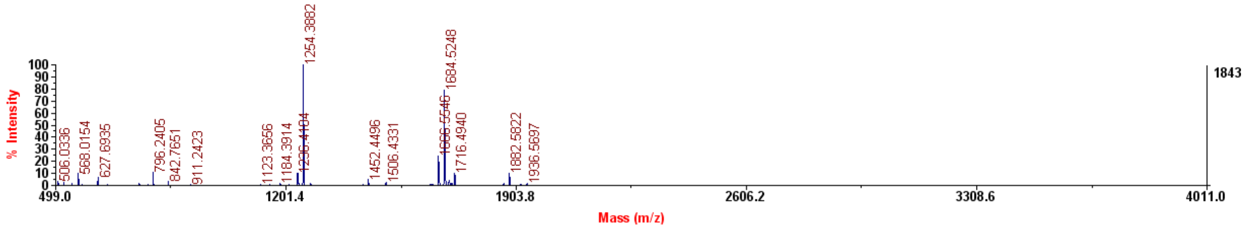

plate 2/line F/column 9

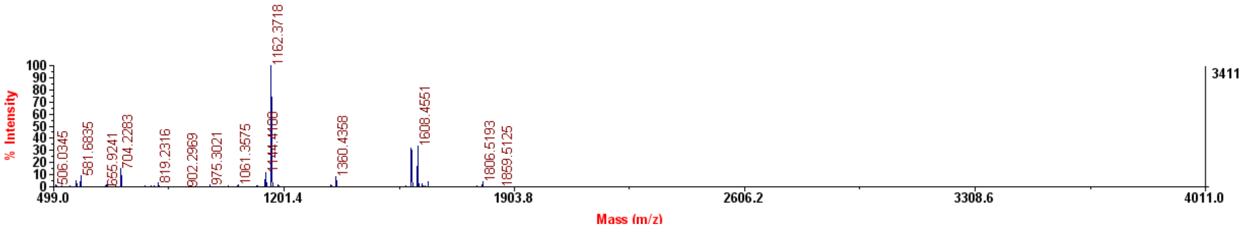

plate 2/line G/column 9

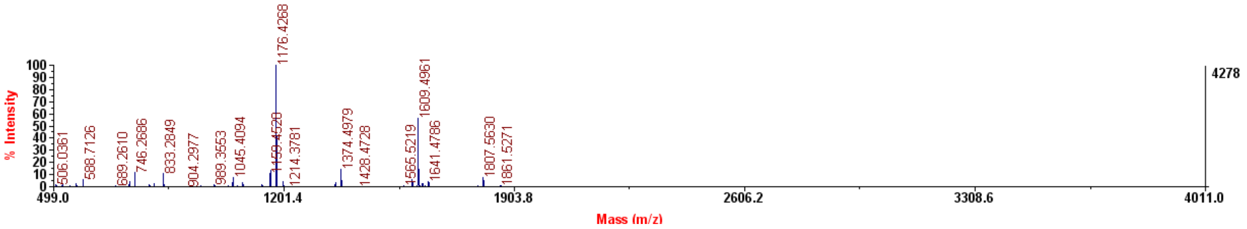

plate 2/line H/column 9

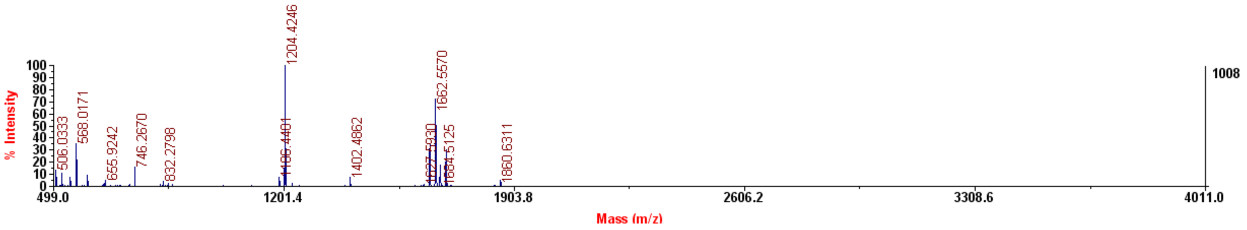

Supplementary Figure 199. MS spectra of plate 2. The data of C9–H9 are shown.

plate 2/line A/column 10

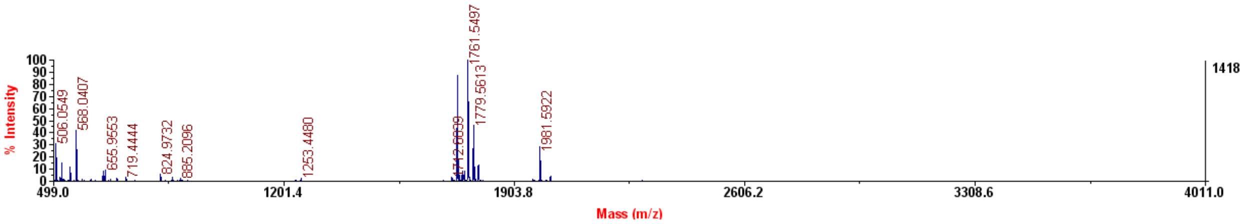

plate 2/line B/column 10

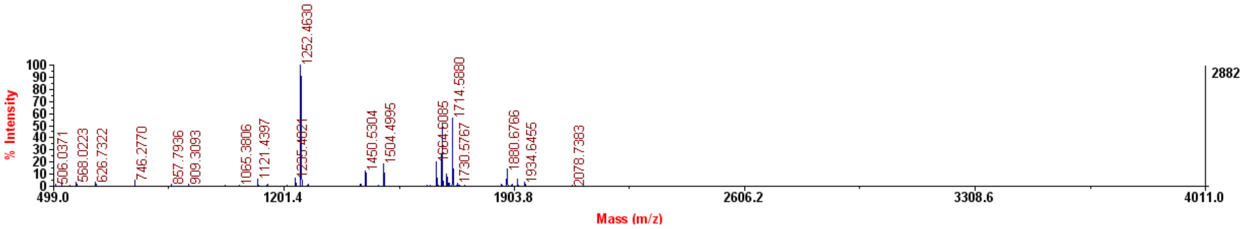

plate 2/line C/column 10

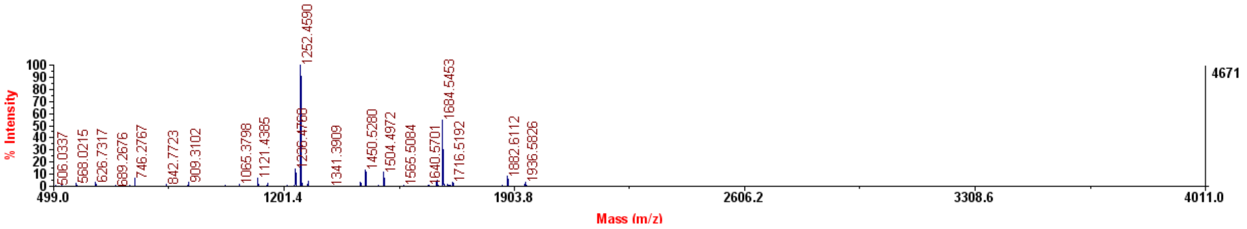

plate 2/line D/column 10

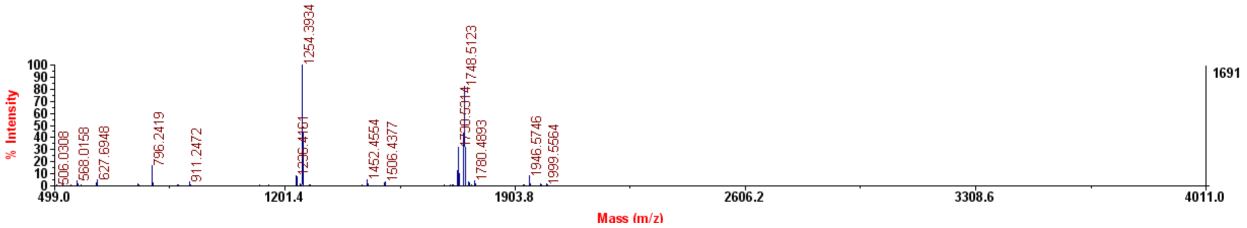

plate 2/line E/column 10

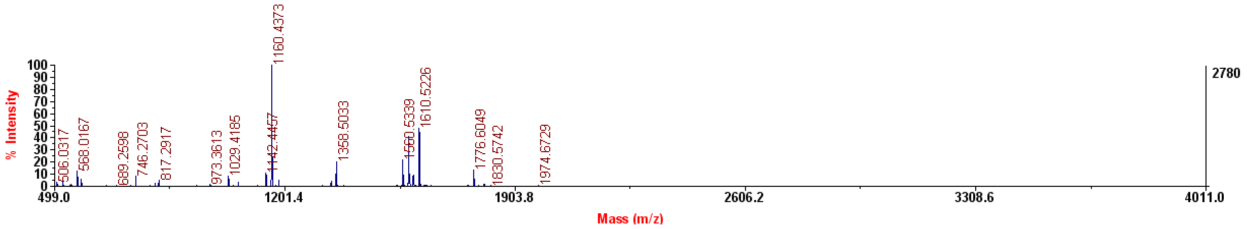

plate 2/line F/column 10

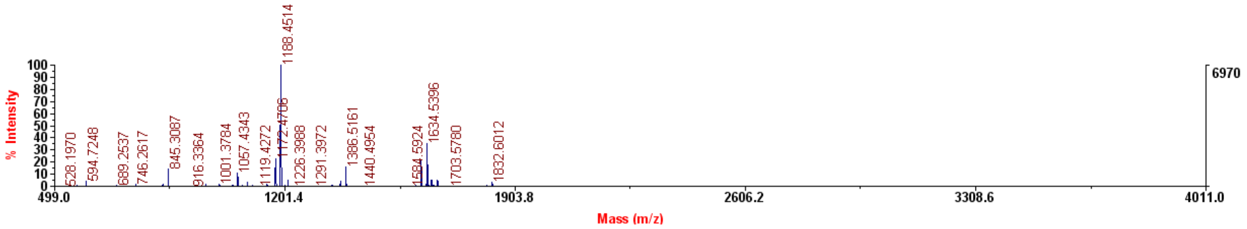

Supplementary Figure 200. MS spectra of plate 2. The data of A10–F10 are shown.

plate 2/line G/column 10

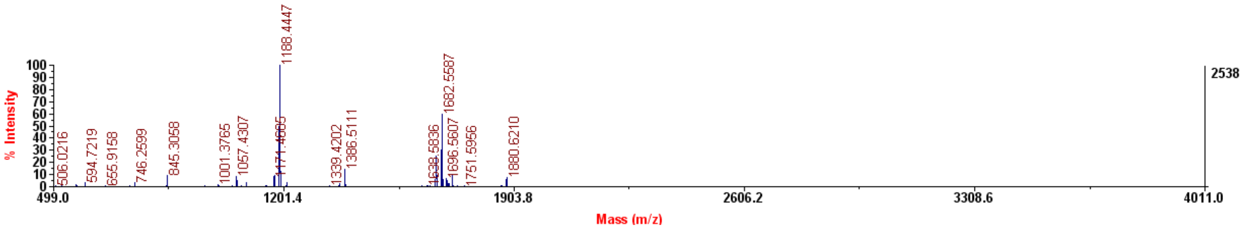

plate 2/line H/column 10

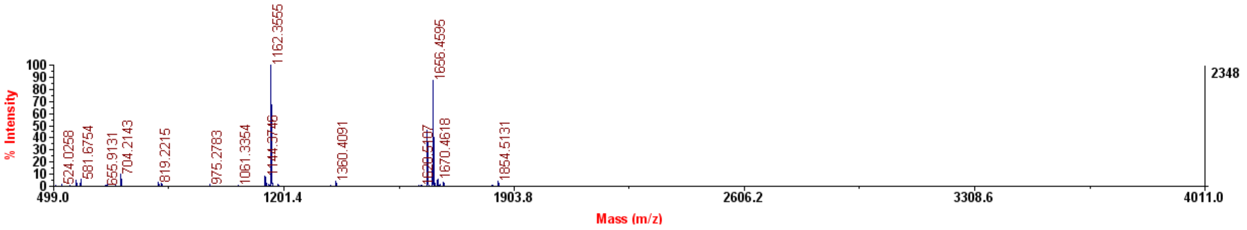

plate 2/line A/column 11

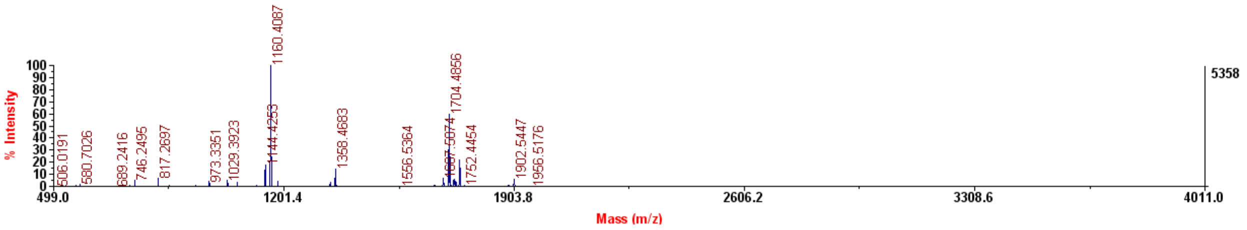

plate 2/line B/column 11

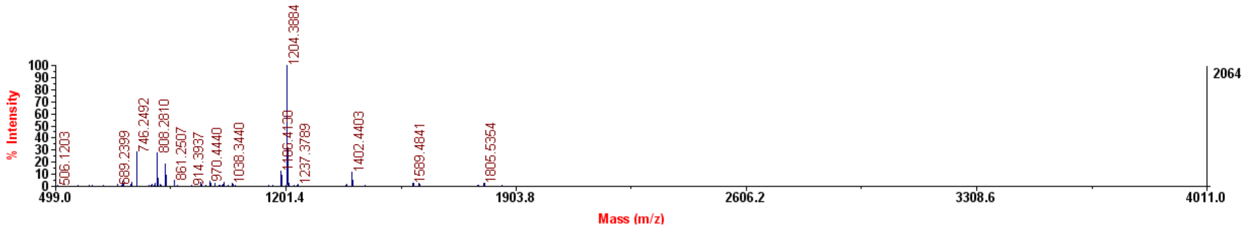

plate 2/line C/column 11

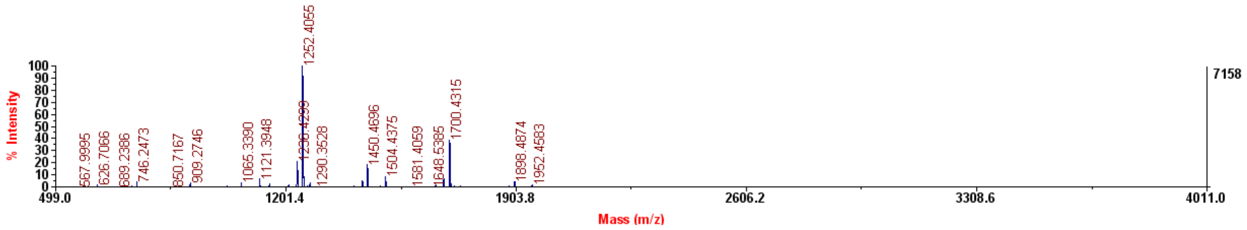

plate 2/line D/column 11

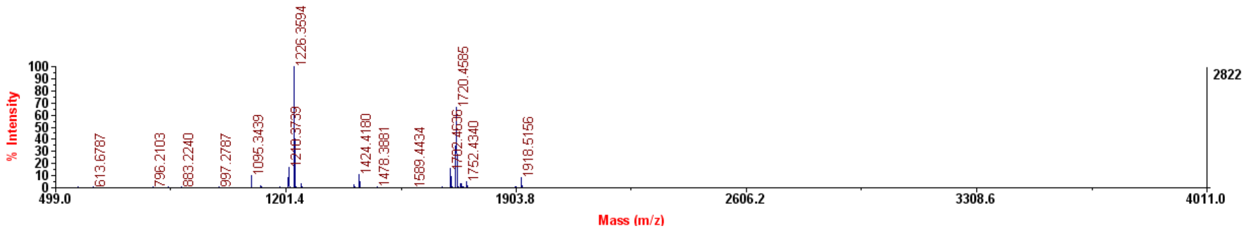

Supplementary Figure 201. MS spectra of plate 2. The data of G10, H10, and A11–D11 are shown.

plate 2/line E/column 11

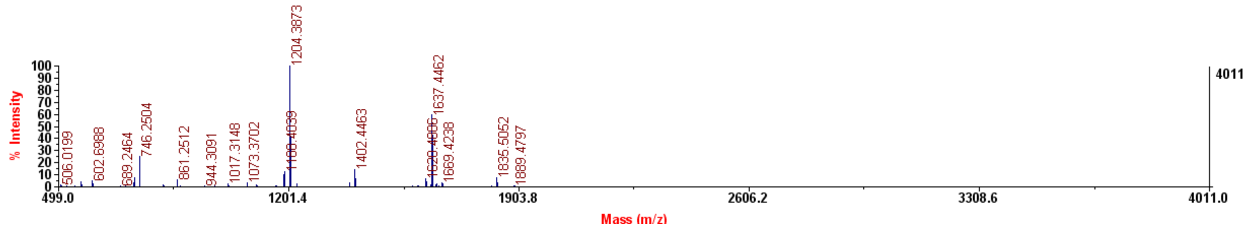

plate 2/line F/column 11

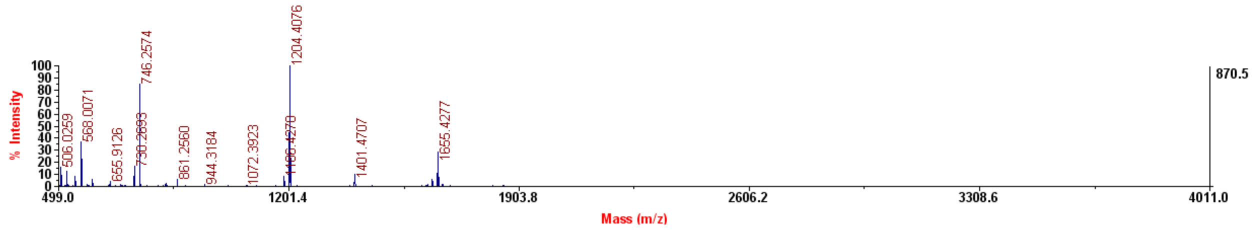

plate 2/line G/column 11

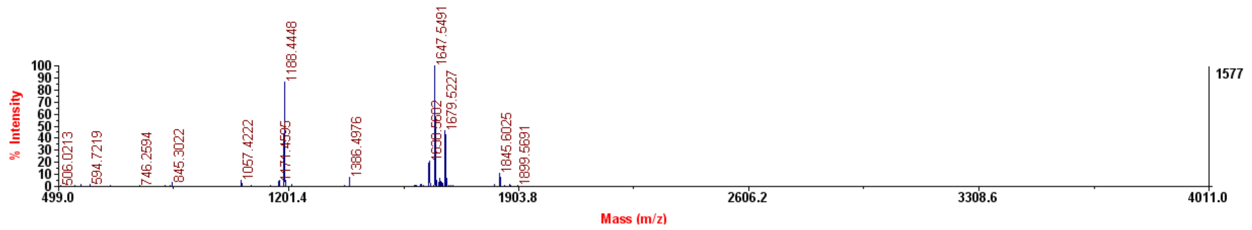

plate 2/line H/column 11

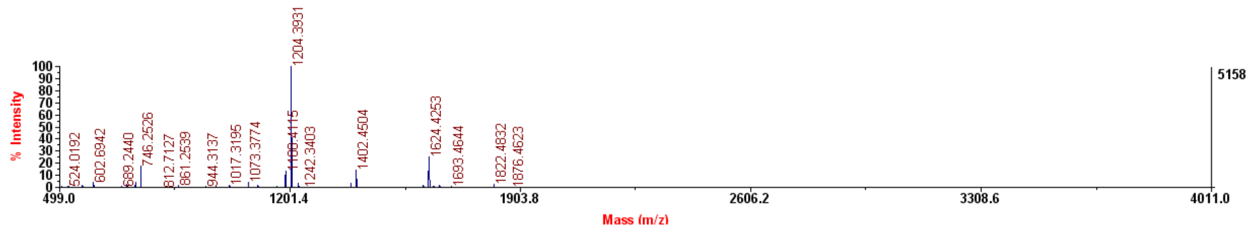

Supplementary Figure 202. MS spectra of plate 2. The data of E11–H11 are shown.

plate 3/line A/column 1

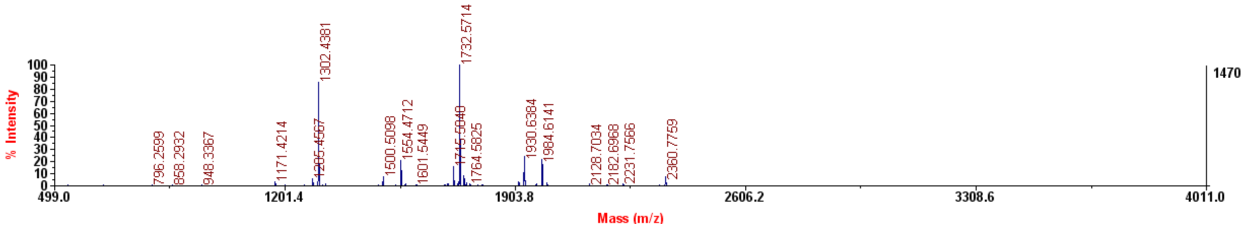

plate 3/line B/column 1

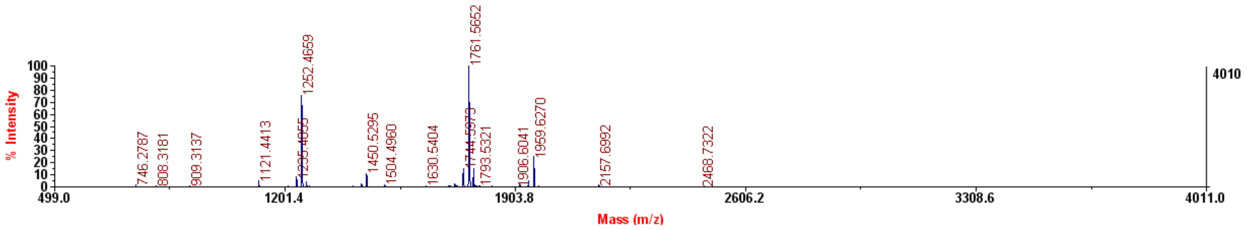

plate 3/line C/column 1

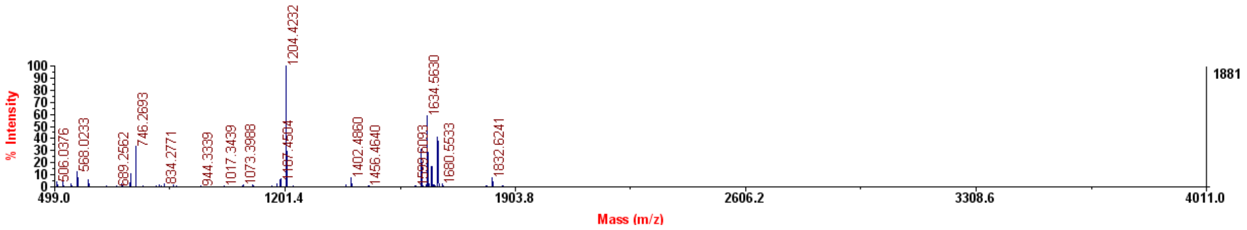

plate 3/line D/column 1

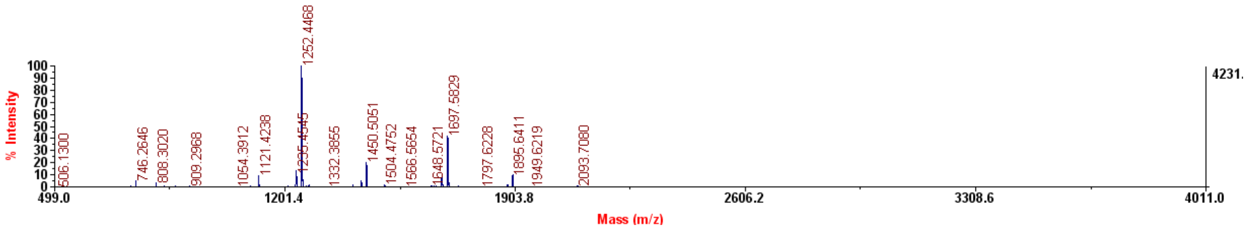

plate 3/line E/column 1

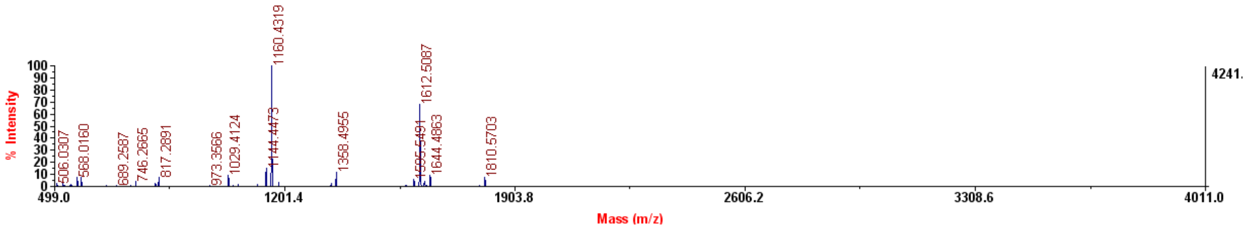

plate 3/line F/column 1

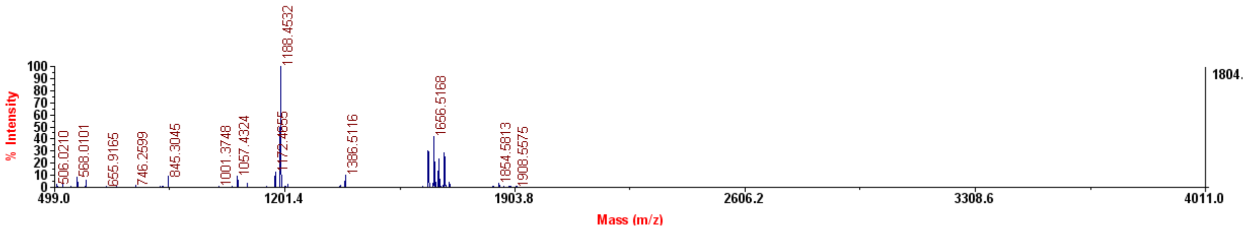

Supplementary Figure 203. MS spectra of plate 3. The data of A1–F1 are shown.

plate 3/line G/column 1

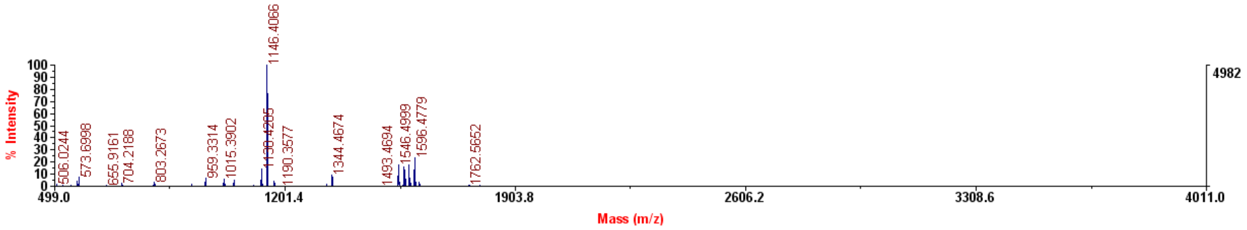

plate 3/line H/column 1

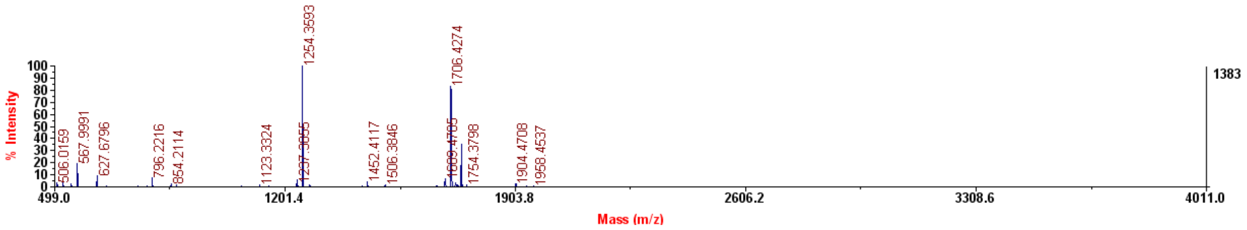

plate 3/line A/column 2

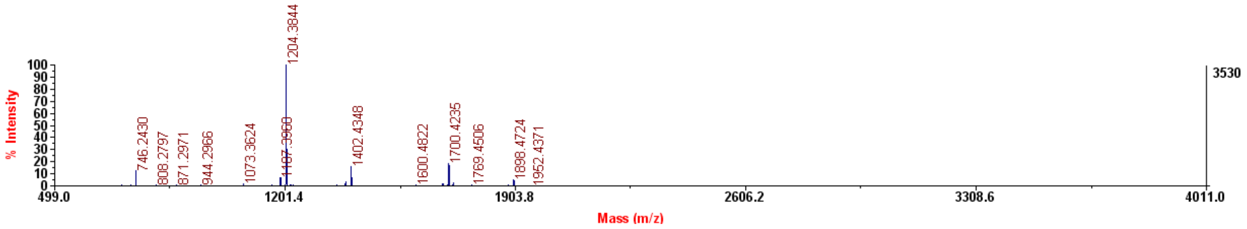

plate 3/line B/column 2

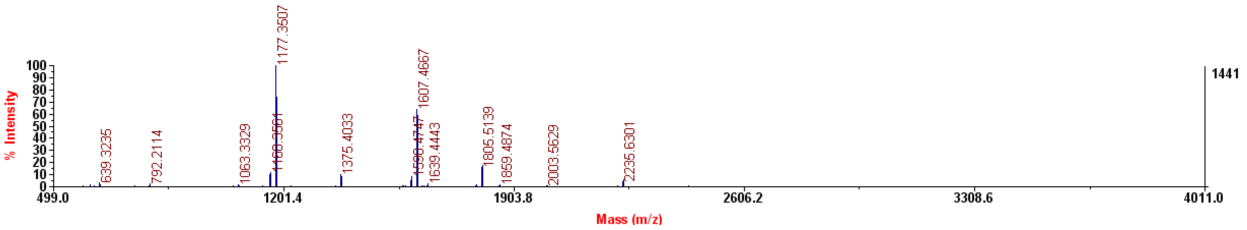

plate 3/line C/column 2

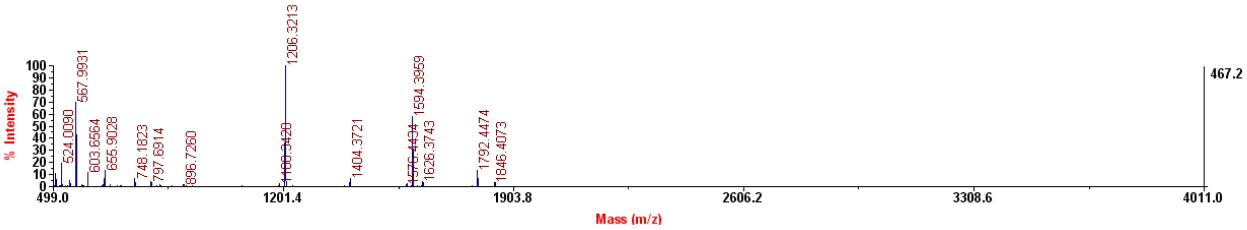

plate 3/line D/column 2

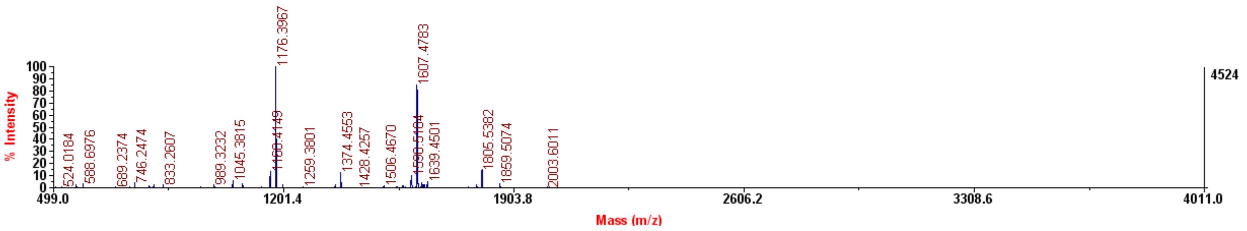

Supplementary Figure 204. MS spectra of plate 3. The data of G1, H1, and A2–D2 are shown.

plate 3/line E/column 2

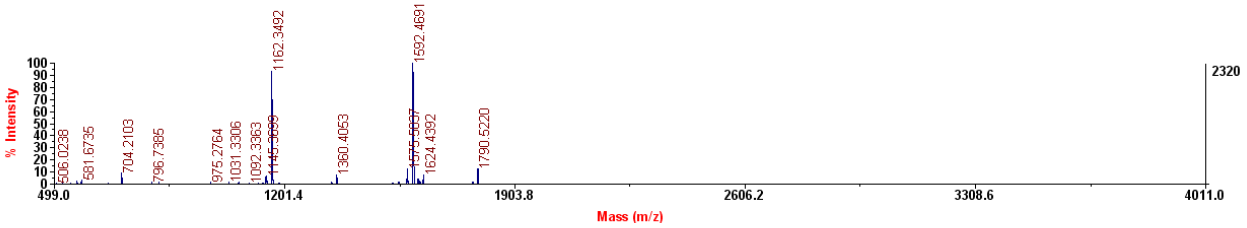

plate 3/line F/column 2

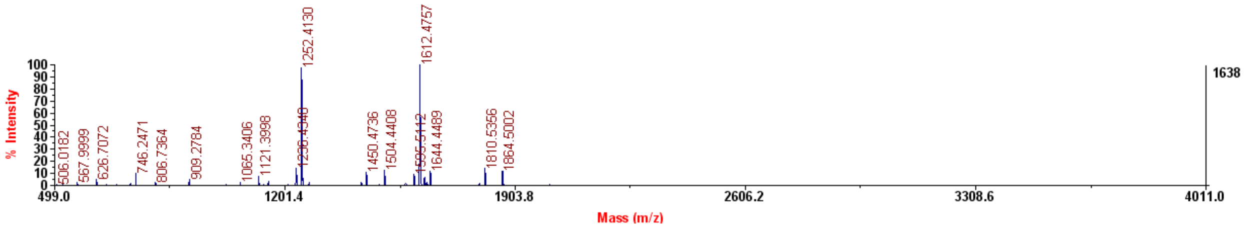

plate 3/line G/column 2

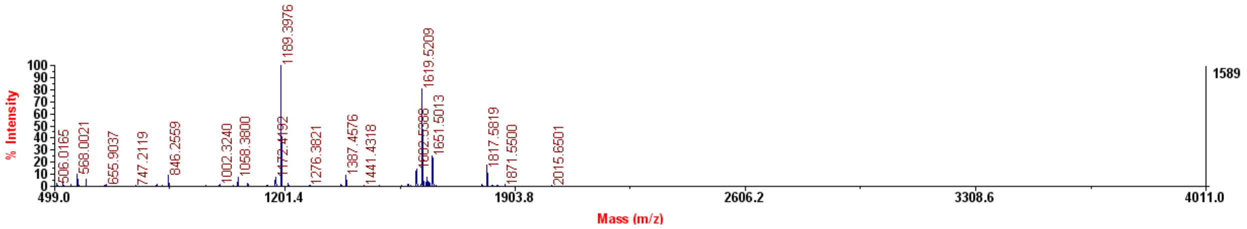

plate 3/line H/column 2

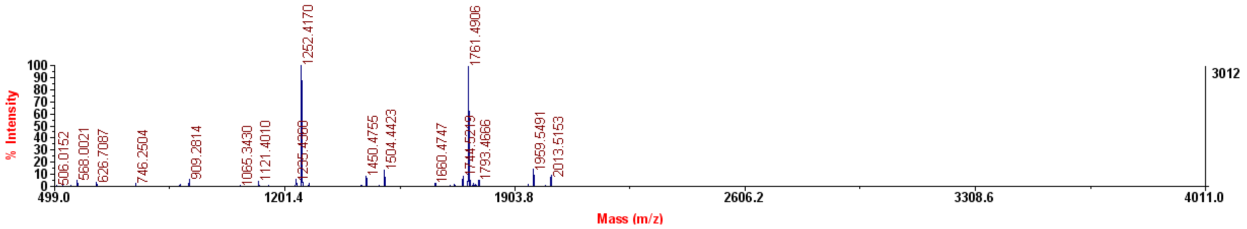

plate 3/line A/column 3

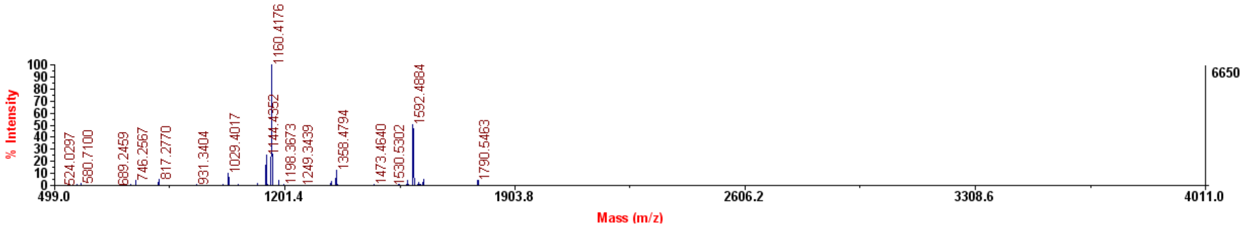

plate 3/line B/column 3

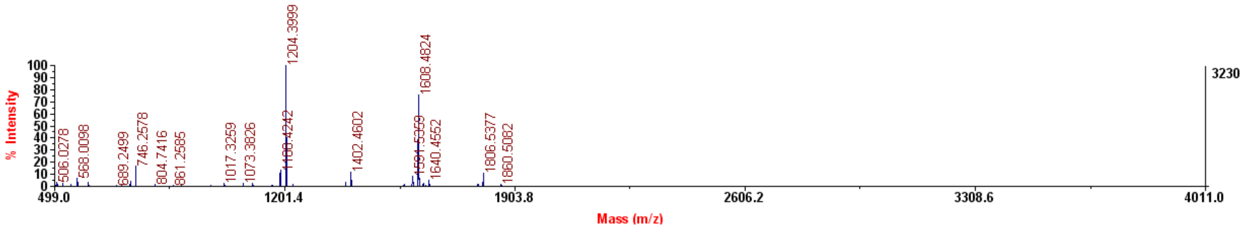

Supplementary Figure 205. MS spectra of plate 3. The data of E2–H2, A3, and B3 are shown.

plate 3/line C/column 3

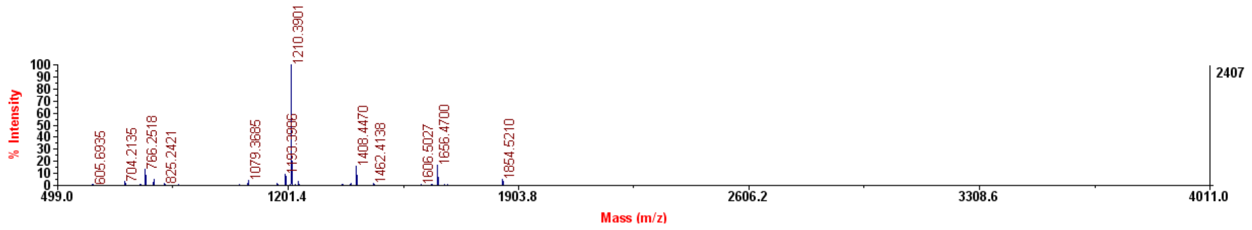

plate 3/line D/column 3

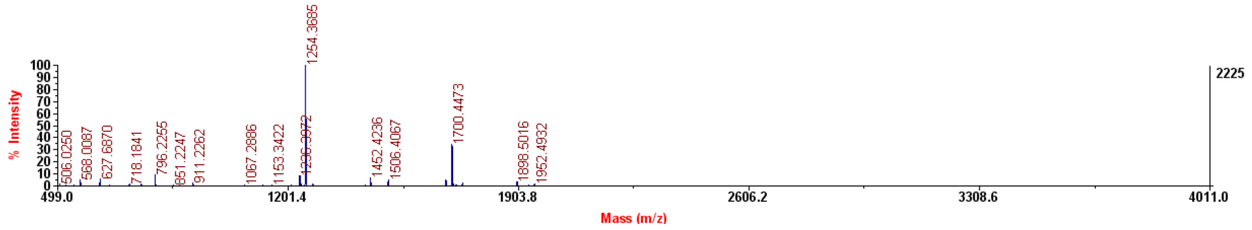

plate 3/line E/column 3

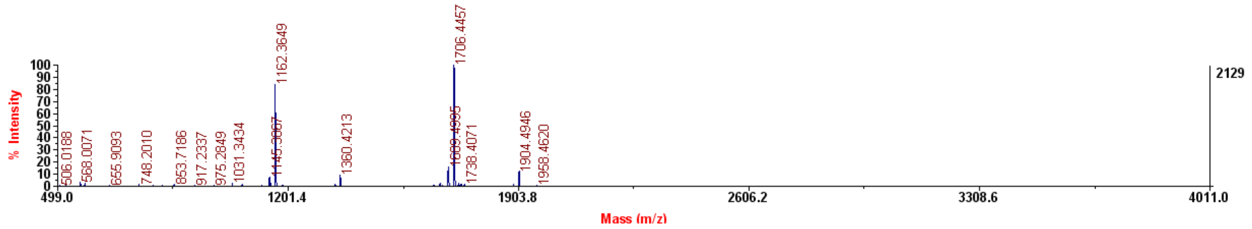

plate 3/line F/column 3

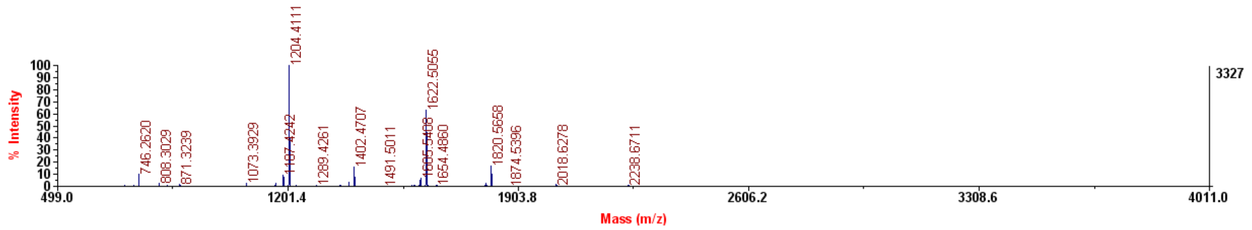

plate 3/line G/column 3

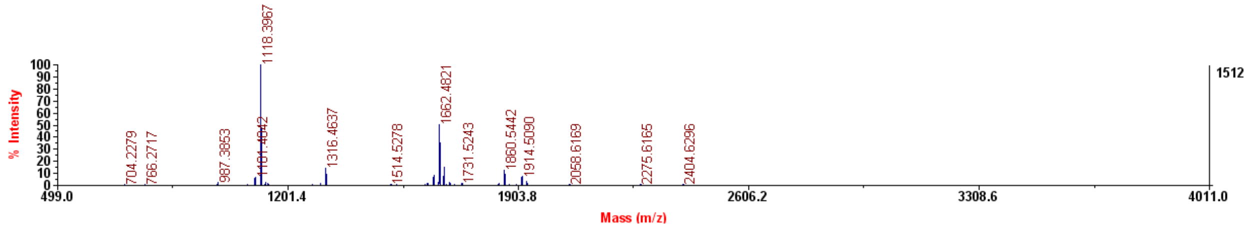

plate 3/line H/column 3

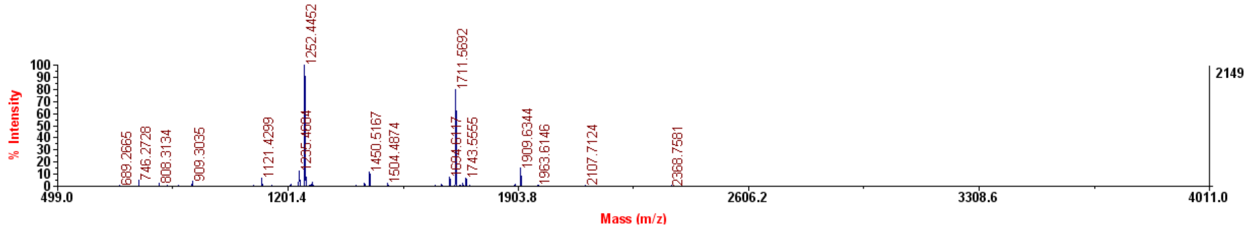

Supplementary Figure 206. MS spectra of plate 3. The data of C3–H3 are shown.

plate 3/line A/column 4

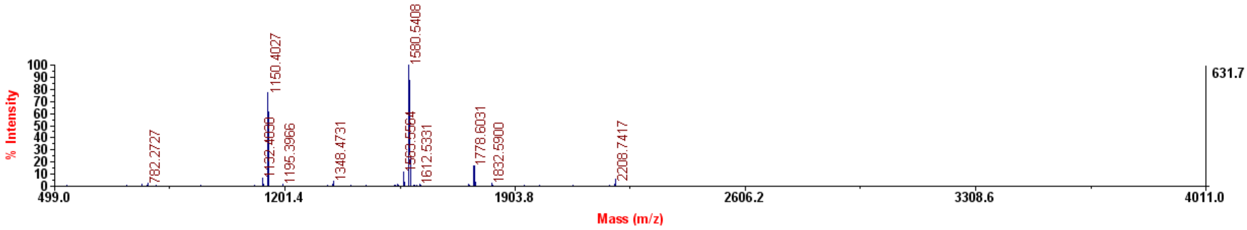

plate 3/line B/column 4

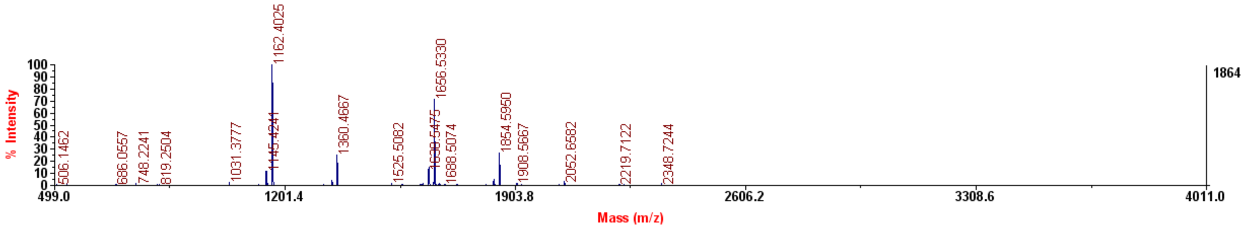

plate 3/line C/column 4

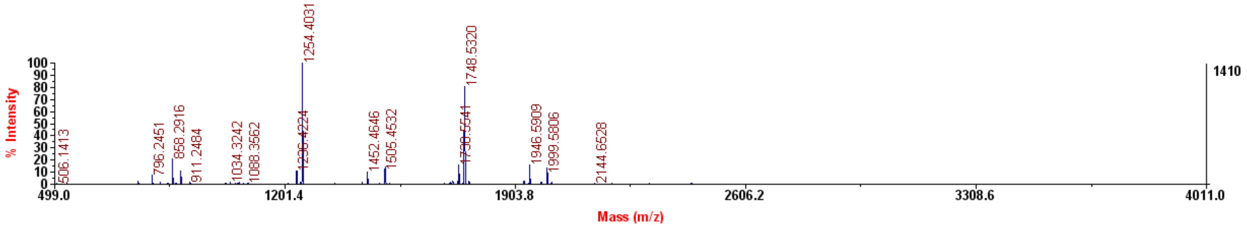

plate 3/line D/column 4

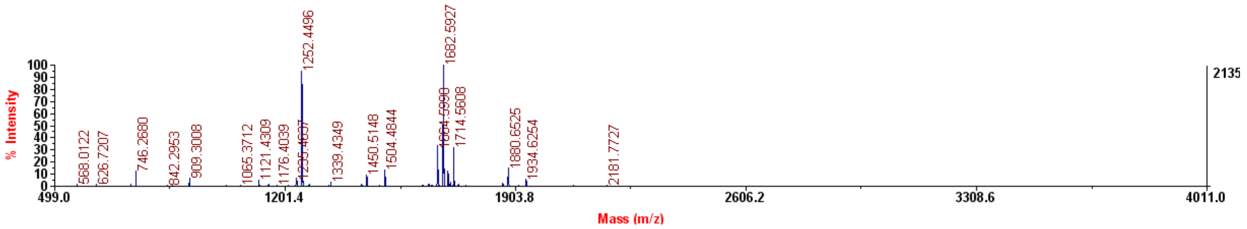

plate 3/line E/column 4

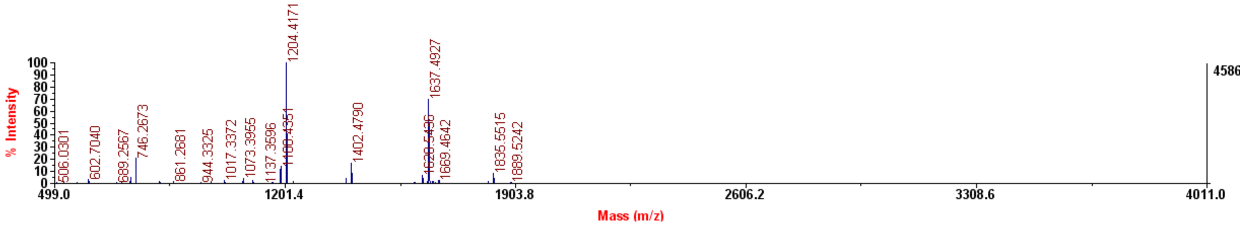

plate 3/line F/column 4

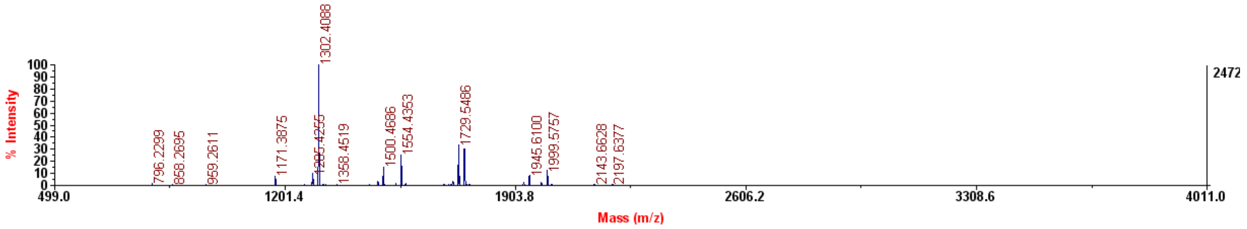

Supplementary Figure 207. MS spectra of plate 3. The data of A4–F4 are shown.

plate 3/line G/column 4

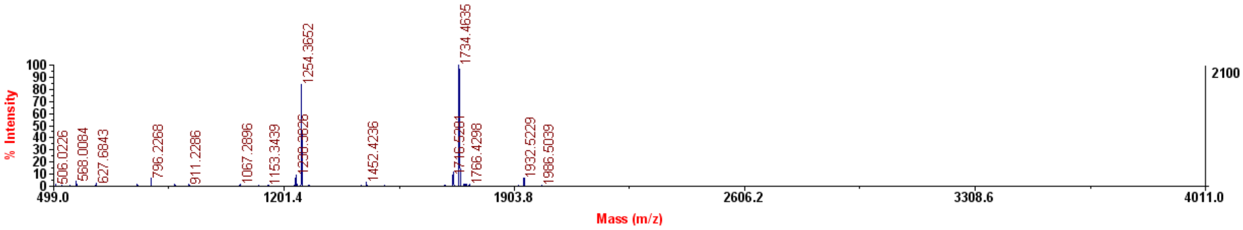

plate 3/line H/column 4

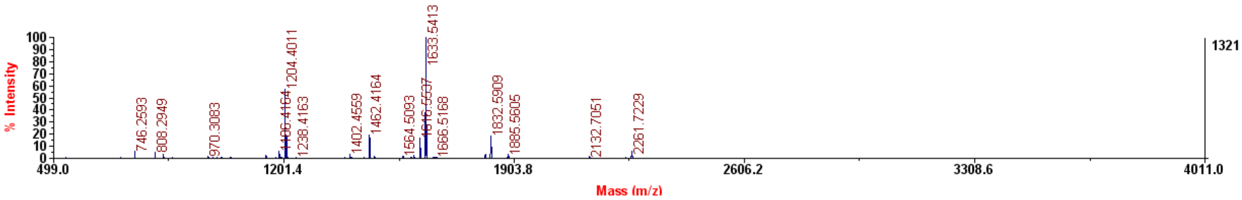

plate 3/line A/column 5

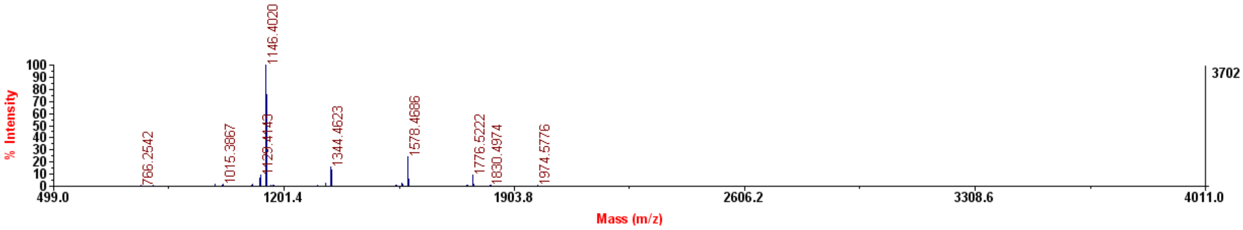

plate 3/line B/column 5

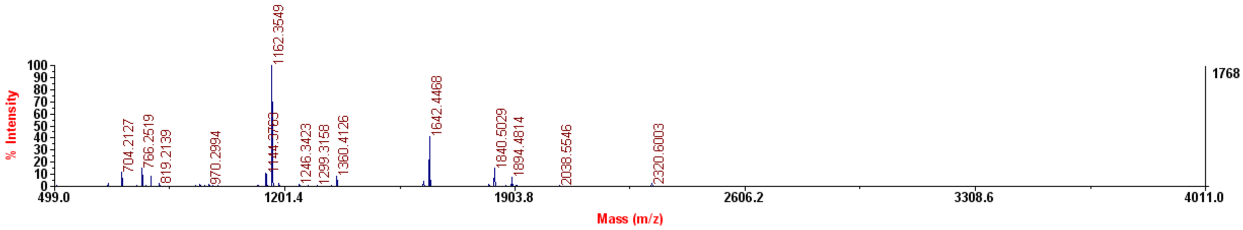

plate 3/line C/column 5

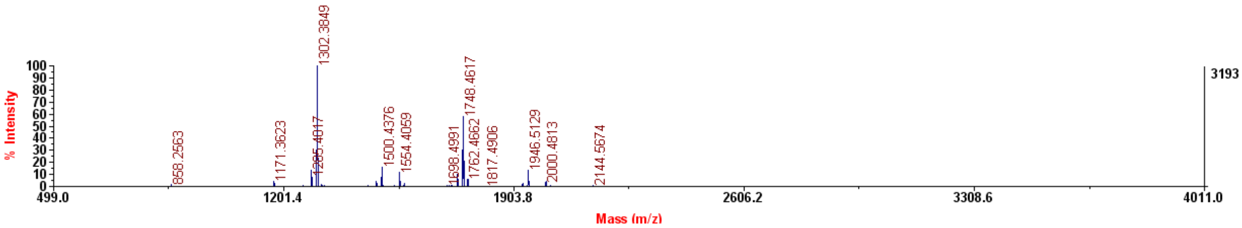

plate 3/line D/column 5

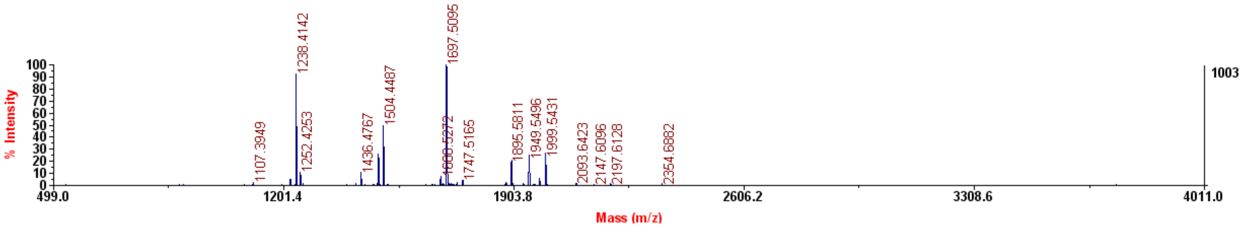

Supplementary Figure 208. MS spectra of plate 3. The data of G4, H4, and A5–D5 are shown.

plate 3/line E/column 5

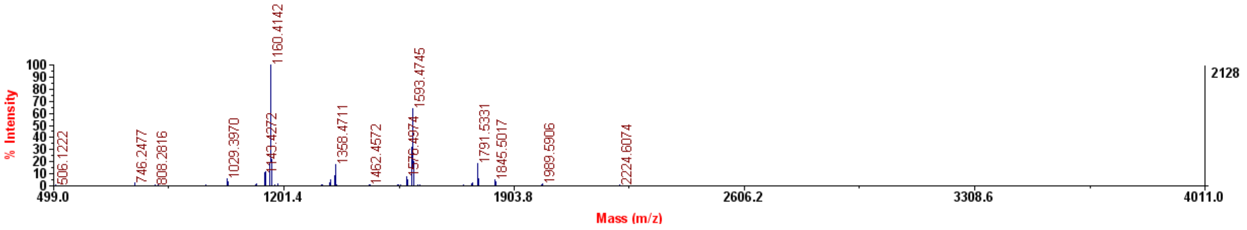

plate 3/line F/column 5

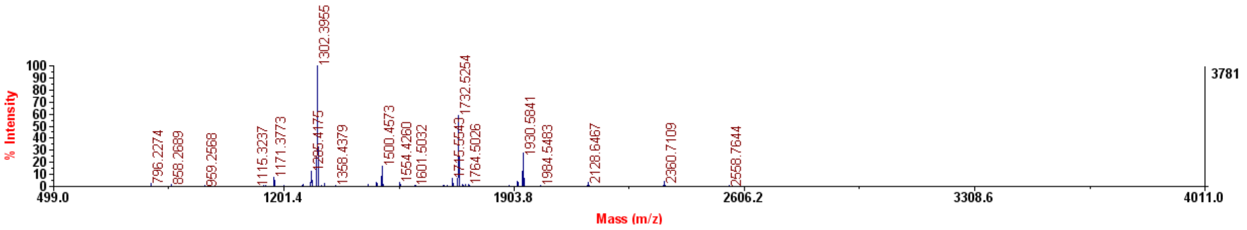

plate 3/line G/column 5

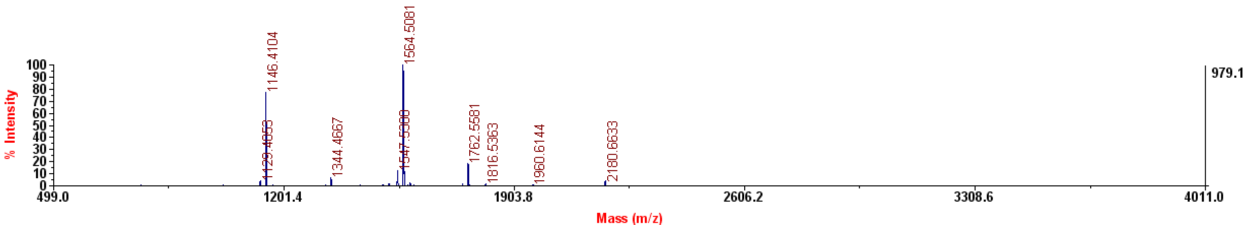

plate 3/line H/column 5

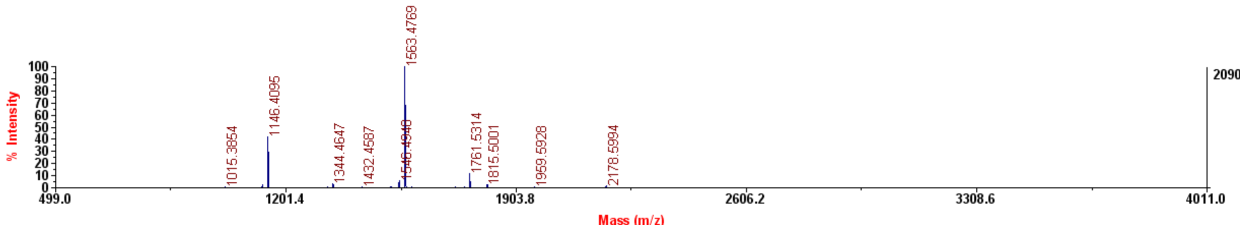

plate 3/line A/column 6

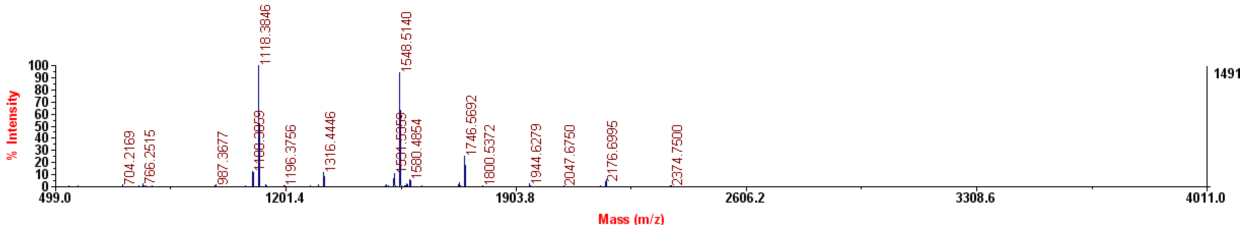

plate 3/line B/column 6

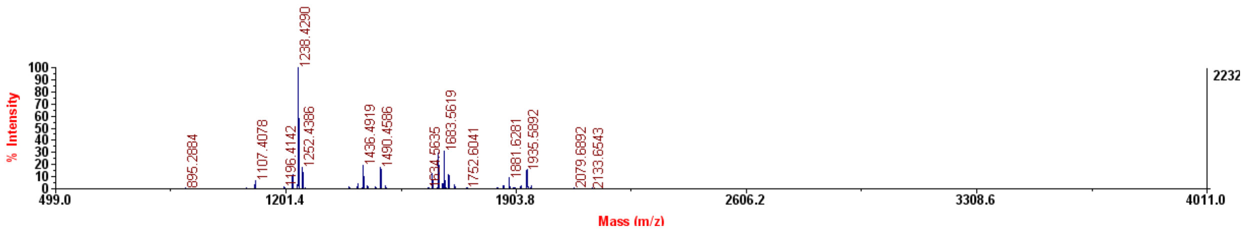

Supplementary Figure 209. MS spectra of plate 3. The data of E5–H5, A6, and B6 are shown.

plate 3/line C/column 6

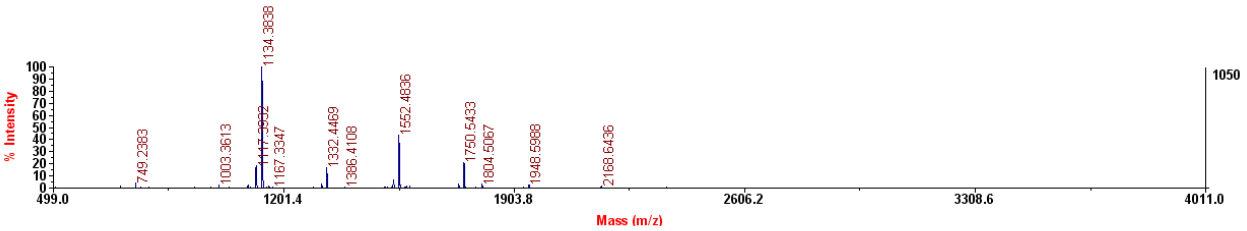

plate 3/line D/column 6

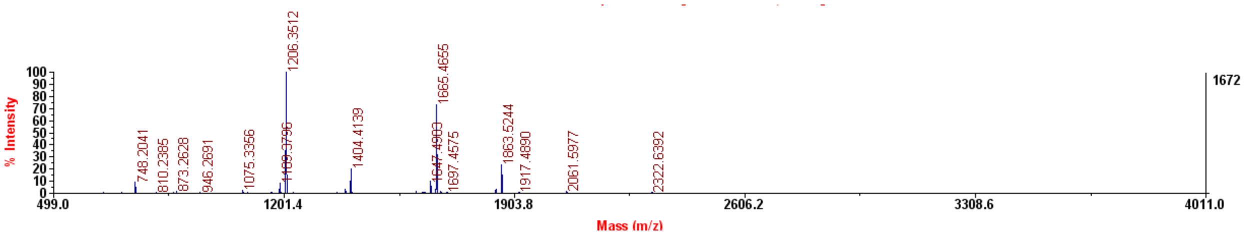

plate 3/line E/column 6

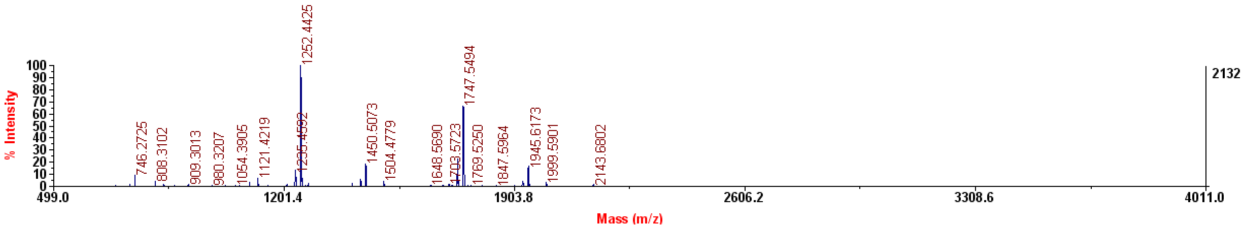

plate 3/line F/column 6

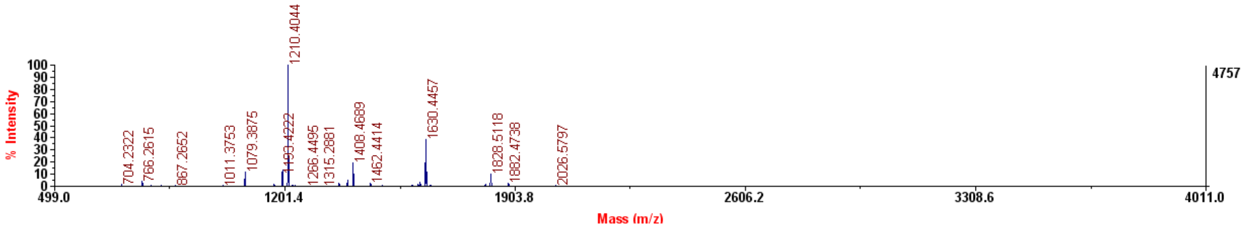

plate 3/line G/column 6

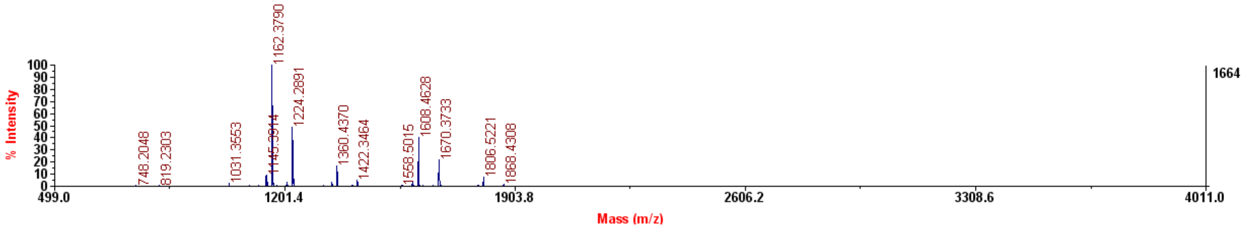

plate 3/line H/column 6

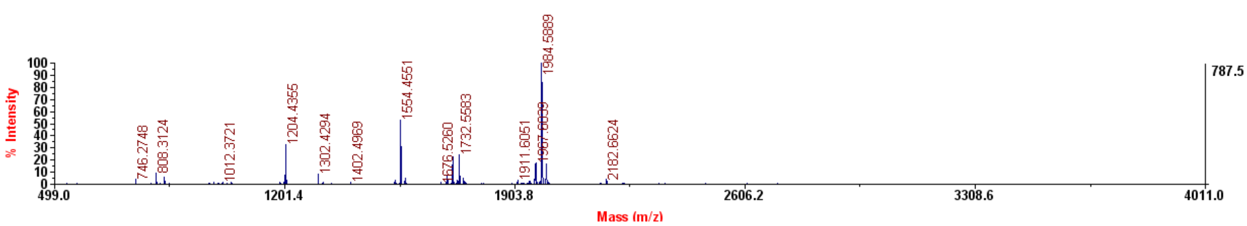

Supplementary Figure 210. MS spectra of plate 3. The data of C6–H6 are shown.

plate 3/line A/column 7

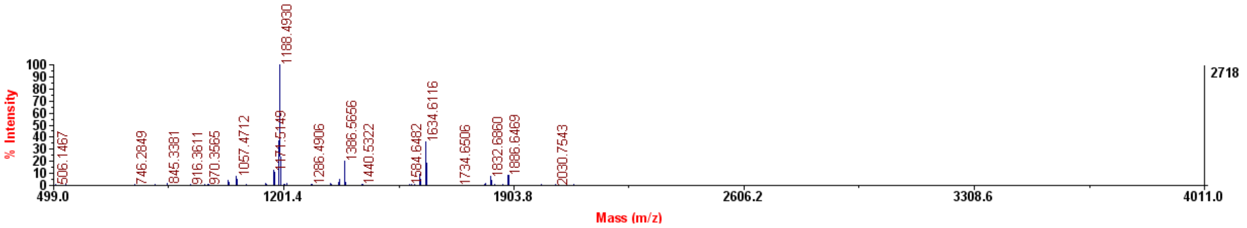

plate 3/line B/column 7

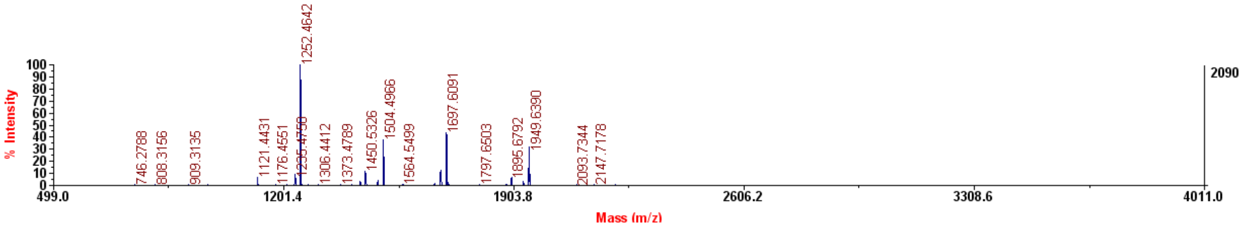

plate 3/line C/column 7

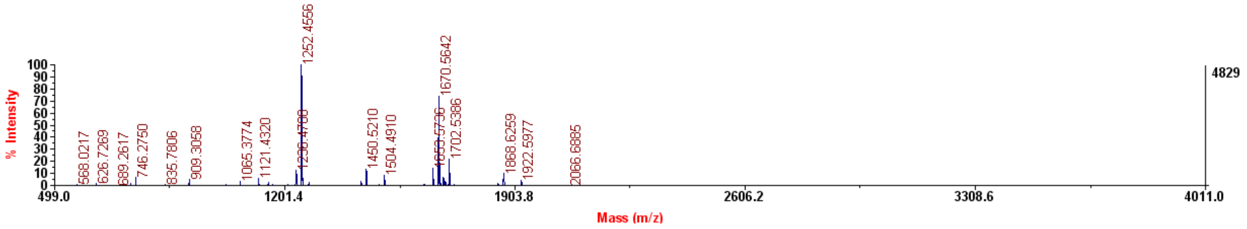

plate 3/line D/column 7

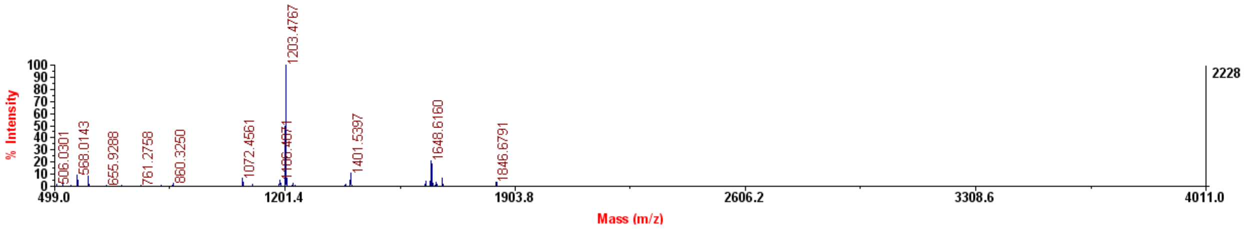

plate 3/line E/column 7

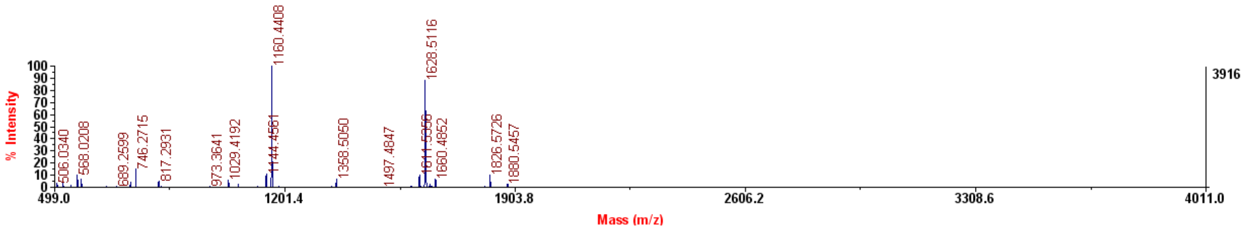

plate 3/line F/column 7

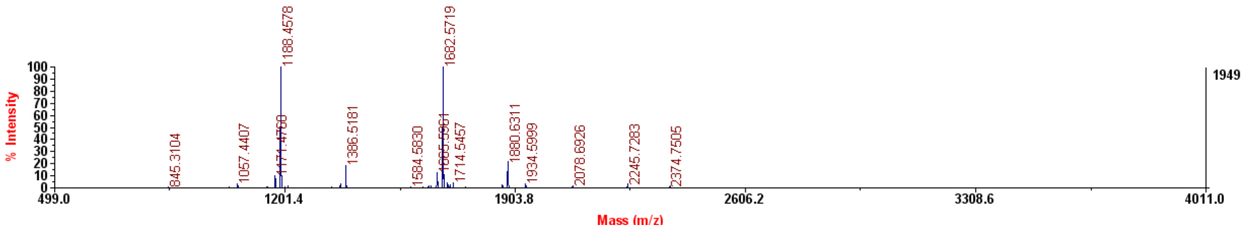

Supplementary Figure 211. MS spectra of plate 3. The data of A7–F7 are shown.

plate 3/line G/column 7

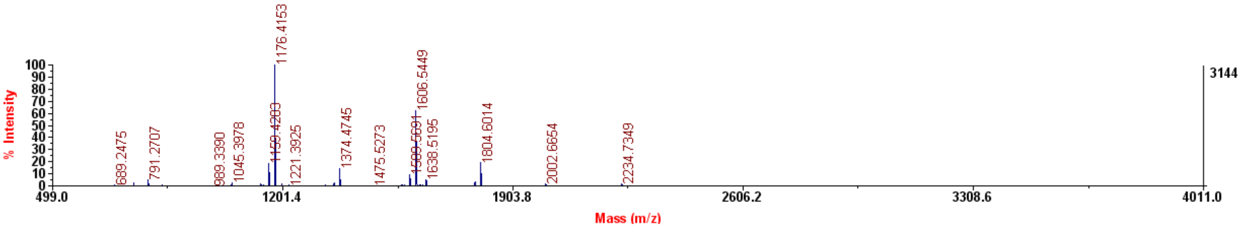

plate 3/line H/column 7

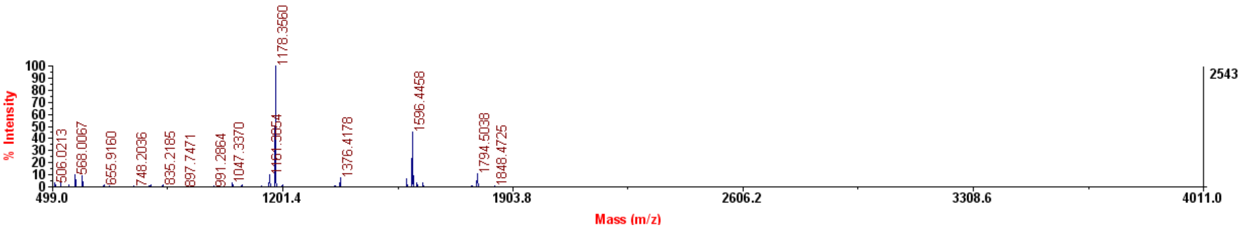

plate 3/line A/column 8

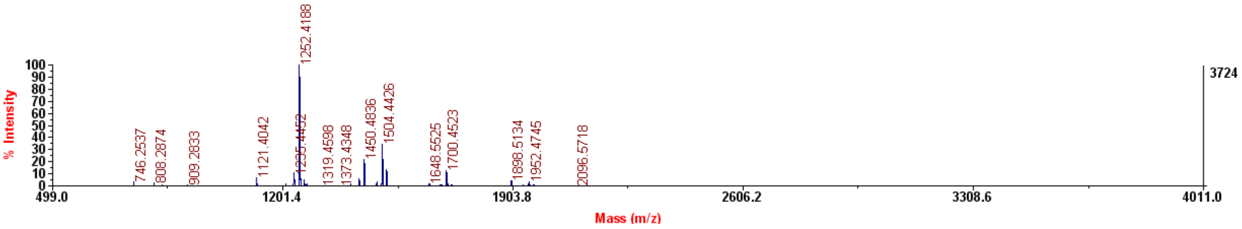

plate 3/line B/column 8

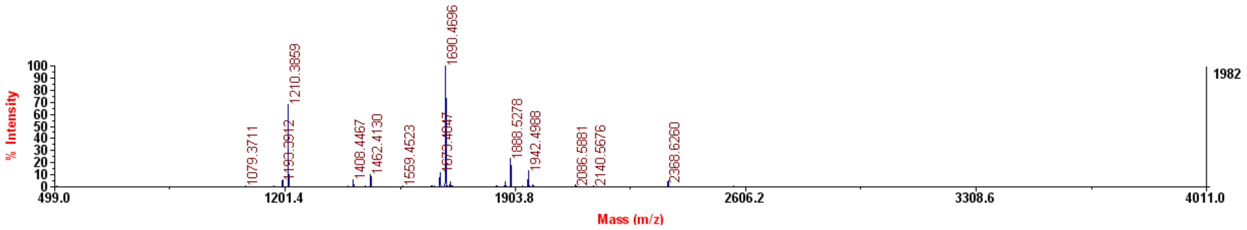

plate 3/line C/column 8

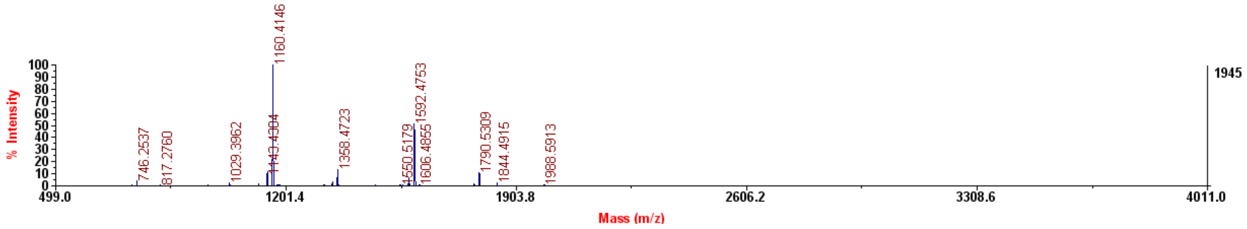

plate 3/line D/column 8

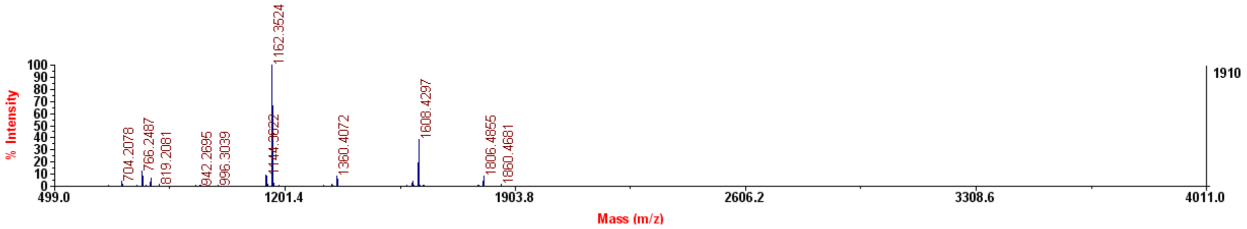

Supplementary Figure 212. MS spectra of plate 3. The data of G7, H7, and A8–D8 are shown.

plate 3/line E/column 8

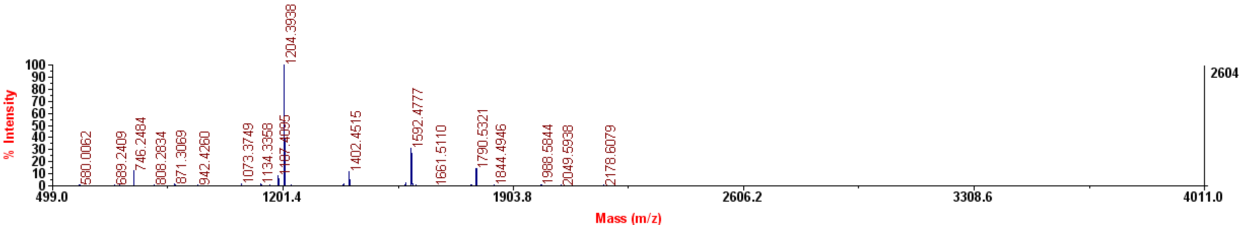

plate 3/line F/column 8

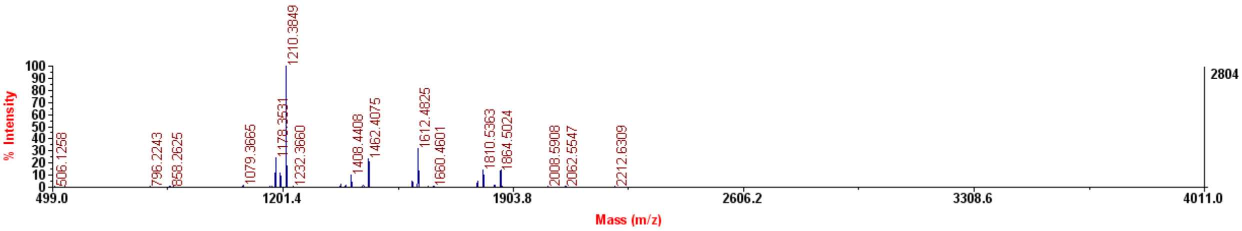

plate 3/line G/column 8

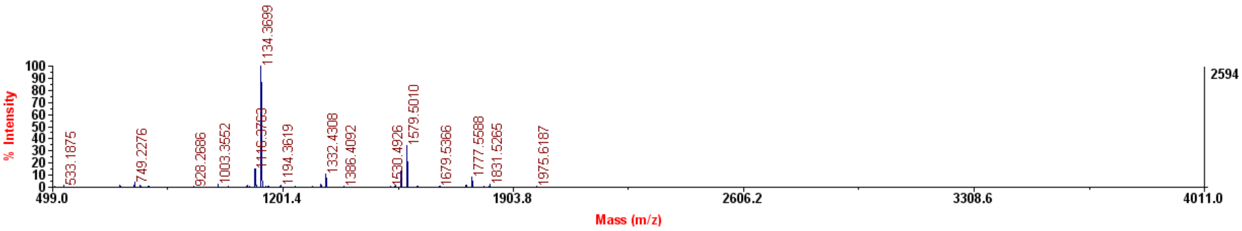

plate 3/line H/column 8

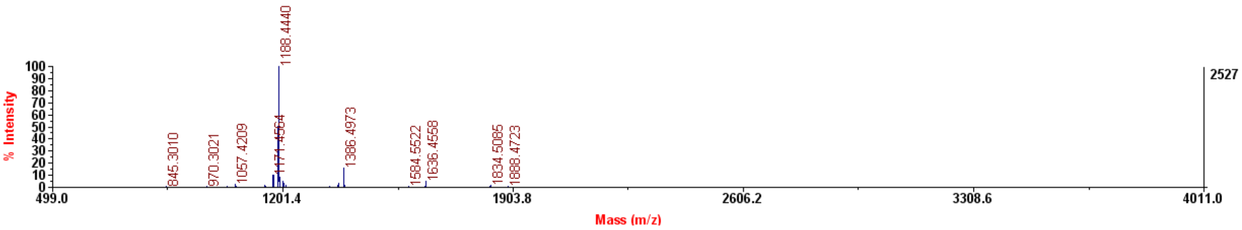

plate 3/line A/column 9

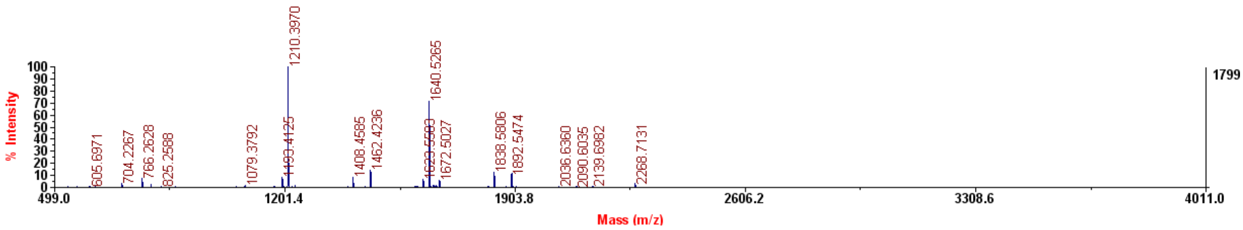

Supplementary Figure 213. MS spectra of plate 3. The data of E8–H8 and A9 are shown.

Supplementary References

1. Clinical and Laboratory Standards Institute. Methods for Dilution Antimicrobial Susceptibility Tests for Bacteria that Grow Aerobically; Approved Standard—Eighth Edition (CLSI document M07–A8). Clinical and Laboratory Standards Institute, Wayne, PA (2009).
2. R Core Team. R: A language and environment for statistical computing. R Foundation for Statistical Computing, Vienna, Austria. URL http://www.R-project.org/ (2018).
3. Ritz, C., Baty, F., Streibig, J. C. & Gerhard, D. Dose-response analysis using R. *PLOS ONE* **10,** e0146021 (2015).
