# Supplementary material for: Development of a high-throughput strategy for discovery of potent analogues of antibiotic lysocin E
Source: Nat Commun. 2019 Jul 5;10:2992. doi: 10.1038/s41467-019-10754-4 (PMC6611794; doi:10.1038/s41467-019-10754-4)
Supplement: Supplementary file 2 — Reporting Summary [file 41467_2019_10754_MOESM2_ESM.pdf]

nature research

Corresponding author(s): Masayuki Inoue

Last updated by author(s): Apr 3, 2019

Reporting Summary

Nature Research wishes to improve the reproducibility of the work that we publish. This form provides structure for consistency and transparency in reporting. For further information on Nature Research policies, see Authors & Referees and the Editorial Policy Checklist.

Statistics

For all statistical analyses, confirm that the following items are present in the figure legend, table legend, main text, or Methods section.

n/a	Confirmed	
☐	☒	The exact sample size (*n*) for each experimental group/condition, given as a discrete number and unit of measurement
☐	☒	A statement on whether measurements were taken from distinct samples or whether the same sample was measured repeatedly
☒	☐	The statistical test(s) used AND whether they are one- or two-sided<br>*Only common tests should be described solely by name; describe more complex techniques in the Methods section.*
☒	☐	A description of all covariates tested
☒	☐	A description of any assumptions or corrections, such as tests of normality and adjustment for multiple comparisons
☐	☒	A full description of the statistical parameters including central tendency (e.g. means) or other basic estimates (e.g. regression coefficient) AND variation (e.g. standard deviation) or associated estimates of uncertainty (e.g. confidence intervals)
☒	☐	For null hypothesis testing, the test statistic (e.g. *F*, *t*, *r*) with confidence intervals, effect sizes, degrees of freedom and *P* value noted<br>*Give P values as exact values whenever suitable.*
☒	☐	For Bayesian analysis, information on the choice of priors and Markov chain Monte Carlo settings
☒	☐	For hierarchical and complex designs, identification of the appropriate level for tests and full reporting of outcomes
☒	☐	Estimates of effect sizes (e.g. Cohen's *d*, Pearson's *r*), indicating how they were calculated

Our web collection on statistics for biologists contains articles on many of the points above.

Software and code

Policy information about availability of computer code

Data collection	Optical rotations were recorded on a P-2200 polarimeter (JASCO). Infrared (IR) spectra were recorded on an FT/IR-4100 spectrometer (JASCO) as a thin film on a CaF2. 1H and 13C NMR spectra were recorded on an ECX 500 (500 MHz for 1H NMR) spectrometer, an ECZ 500R (500 MHz for 1H NMR) spectrometer (JEOL), or an Avance III HD 800 MHz equipped with CryoProbe (800 MHz for 1H NMR, 200 MHz for 13C NMR) (Bruker). HRMS spectra were recorded on a MicrOTOFII (Bruker Daltonics) electrospray ionization time-of-flight (ESI-TOF) mass spectrometer. UV absorbance was measured on a UV-1800 UV-VIS spectrophotometer (Shimadzu).
Data analysis	Sigmoidal curve fittings were performed on R with drc package. The structures of the compounds were drawn with ChemBioDraw Ultra 16.0.

For manuscripts utilizing custom algorithms or software that are central to the research but not yet described in published literature, software must be made available to editors/reviewers. We strongly encourage code deposition in a community repository (e.g. GitHub). See the Nature Research guidelines for submitting code & software for further information.

Data

Policy information about availability of data

All manuscripts must include a data availability statement. This statement should provide the following information, where applicable:

- Accession codes, unique identifiers, or web links for publicly available datasets
- A list of figures that have associated raw data
- A description of any restrictions on data availability

The authors declare that the data supporting the findings of this study are available within the paper and its supplementary information files.

Field-specific reporting

Please select the one below that is the best fit for your research. If you are not sure, read the appropriate sections before making your selection.

☒ Life sciences ☐ Behavioural & social sciences ☐ Ecological, evolutionary & environmental sciences

For a reference copy of the document with all sections, see nature.com/documents/nr-reporting-summary-flat.pdf

Life sciences study design

All studies must disclose on these points even when the disclosure is negative.

Sample size	The sample size (n) of each experiment was provided in the figure/table legends in the main manuscript and in supporting information.
Data exclusions	No data were excluded from the analyses.
Replication	All the experimental findings were reliably reproduced.
Randomization	n/a
Blinding	In antimicrobial activity assay, the performers were blinded to the samples.

Reporting for specific materials, systems and methods

We require information from authors about some types of materials, experimental systems and methods used in many studies. Here, indicate whether each material, system or method listed is relevant to your study. If you are not sure if a list item applies to your research, read the appropriate section before selecting a response.

Materials & experimental systems

n/a	Involved in the study	
☒	☐	Antibodies
☒	☐	Eukaryotic cell lines
☒	☐	Palaeontology
☒	☐	Animals and other organisms
☒	☐	Human research participants
☒	☐	Clinical data

Methods

n/a	Involved in the study	
☒	☐	ChIP-seq
☒	☐	Flow cytometry
☒	☐	MRI-based neuroimaging
